# 1,2-Aminoxyalkylation of alkenes with alkyl iodides and TEMPONa through SET- and XAT-processes†

**DOI:** 10.1039/d3sc02544f

**Published:** 2023-06-23

**Authors:** Anirban Maity, Armido Studer

**Affiliations:** a Organisch-Chemisches Institut, Westfälische Wilhelms-Universität Corrensstraße 40 48149 Munster Germany studer@uni-muenster.de

## Abstract

1,2-Aminoxyalkylation of alkenes with alkyl iodides and TEMPONa in combination with an aryldiazonium salt as an XAT mediator is reported. Various primary, secondary and tertiary alkyl iodides engage as C-radical precursors in the 1,2-aminoxyalkylation with electrophilic alkenes as radical acceptors. The product alkoxyamines are readily transformed to the corresponding alcohols or ketones upon reduction or oxidation, respectively. Mechanistic investigations reveal that aryl radicals, generated through SET-reduction of the aryl diazonium salt with TEMPONa, engage in XAT from unactivated alkyl halides to give alkyl radicals that can add to alkenes. Trapping of the adduct radicals with TEMPO provides the 1,2-aminoxyalkylation products. Transition metals are not required for these transformations that are conducted under mild conditions. Perfluoroalkyl halides directly react with TEMPONa and an aryldiazonium salt as XAT-mediator is not required for alkene 1,2-aminoxyperfluoroalkylation.

## Abstract 2

1,2-Aminoxyalkylation of alkenes with alkyl iodides and TEMPONa in combination with an aryldiazonium salt as an XAT mediator is reported.

## Graphical abstract

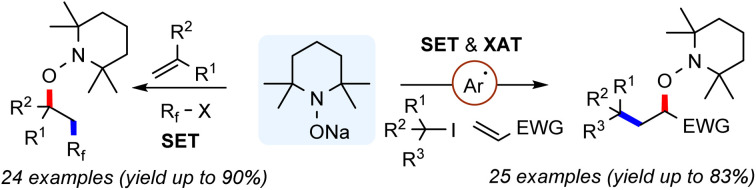

## Introduction

**Scheme 1:**
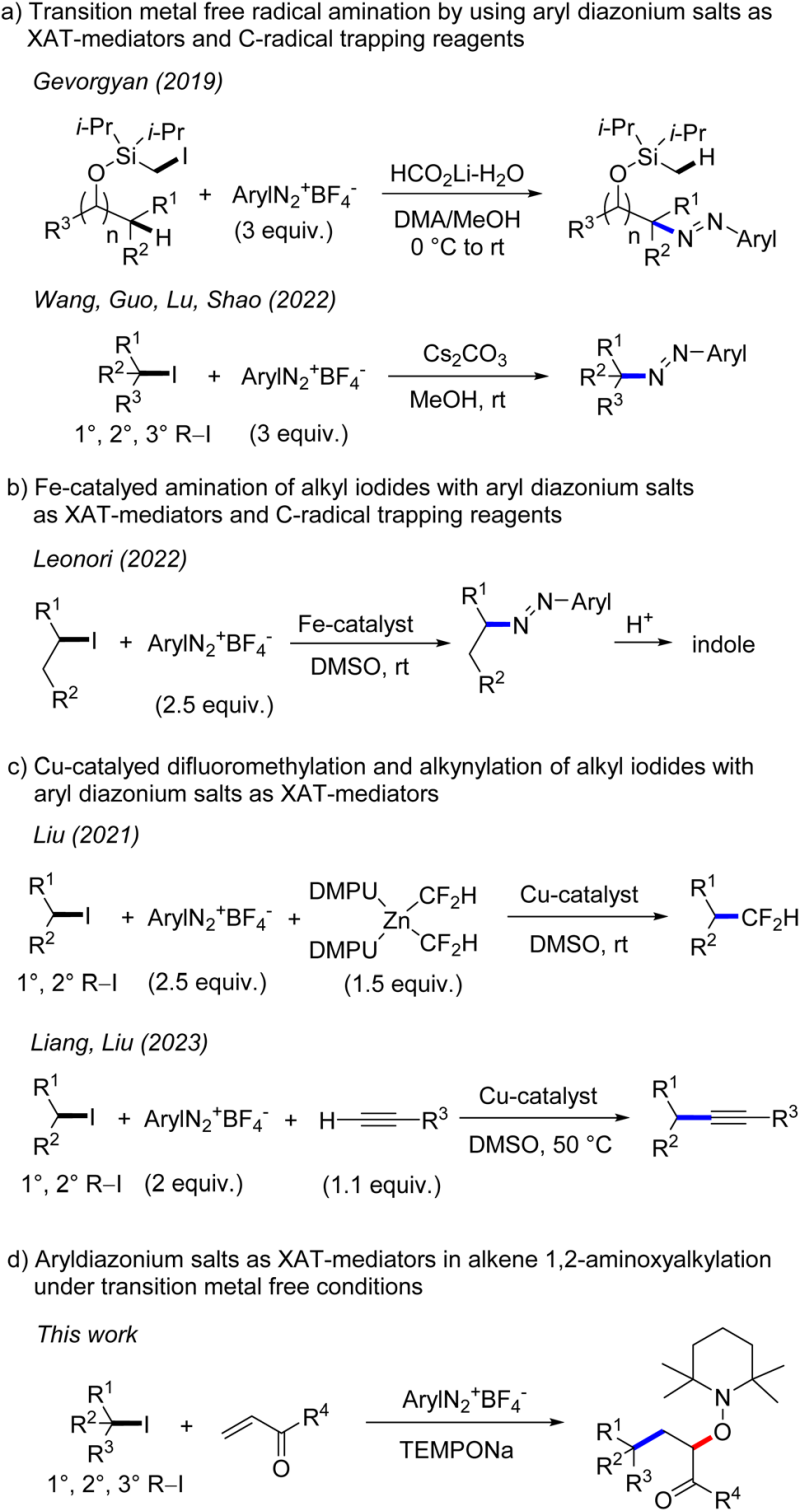
Aryldiazonium salts as XAT-mediators.

The generation of alkyl radicals from various precursors has been intensively explored in the past.^1^ It is well established that unactivated alkyl halides serve as efficient C-radical precursors in classical tin hydride mediated radical transformations.^2^ Considering the toxicity of tin hydrides,^3^ transition metal catalysis^4^ and photoredox catalysis^5^ have been successfully implemented for tin free C-radical generation from reactive alkyl halides. Despite significant advances, generation of C-radicals from unactivated alkyl halides is still challenging and in particular transition metal free processes are underdeveloped. This is mainly due to the fact that unactivated alkyl halides have highly negative reduction potentials^6^ (*E*_red_ < −2.0 V *vs.* SCE, for alkyl iodides) which render their direct SET-reduction challenging. However, it is known that the SET-reduction of aryl diazonium salts to give aryl radicals and N_2_ occurs at less negative cathodic peak potential (peak potential: *E*_p_ = −0.16 V/SCE for PhN_2_BF_4_)^7^ and consequently can easily be achieved with various mild reductants. Further, aryl radicals are known to efficiently react with alkyl iodides through iodine atom transfer (XAT), due to the thermodynamic driving force of such XATs ((BDE) of C(sp^3^)–I (BDE of primary alkyl iodide is 57 kcal mol^−1^) and C(sp^2^)–I (BDE of iodobenzene is 67 kcal mol^−1^)^8^). Therefore, aryl diazonium salts could be used as mediators for indirect C-radical generation through SET reduction and subsequent XAT in systems where the direct SET reduction of an alkyl halide is difficult or impossible to achieve. Along these lines, Gevorgyan and co-workers disclosed in 2019 transition metal free remote C–H amination of iodomethyl silyl ethers, with aryl diazonium salts involved for the generation of primary alkyl radicals through XAT (Scheme 1).^9^ Very recently, transition metal free C–N bond formation from alkyl iodides and diazonium salts was developed by Wang, Guo, Lu, Shao and co-workers using a similar strategy.^10^ An analogous Fe-catalyzed process was introduced by the Leonori group and was successfully applied for indole synthesis.^11^ In these cases, the aryl diazonium salt acts as an XAT-mediator as well as a C-radical trapping reagent and accordingly a minimum 2 fold excess of the salt is required. Liu and co-workers reported elegant copper catalyzed difluoromethylation of alkyl iodides *via* aryl radical mediated generation of alkyl radicals through XAT.^12^ Liang and Liu^13*a*^ as well as the Leonori group^13*b*^ independently reported copper catalysed Sonogashira-type coupling and C(sp^3^)–N/O/C bond formation following such an approach. In these transformations, aryl radicals acting as XAT-mediators are generated through SET-reduction of aryl diazonium salts by a Cu-catalyst.

In 2012, our group showed that aryl radicals can be cleanly formed by sodium 2,2,6,6-tetramethylpiperidin-1-olate (TEMPONa) mediated reduction of aryl diazonium salts, a reaction that was successfully used for transition metal free oxyarylation of alkenes.^14^ However, alkyl radical generation from alkyl iodides through reduction with TEMPONa was not possible, as the TEMPO sodium salt is a weak reductant.^15^ We wondered whether alkyl radicals can be indirectly generated from alkyl iodides and TEMPONa by using aryl diazonium salts as XAT-mediators. According to the reaction design, the intermediately generated aryl radicals should engage in iodine atom abstraction from unactivated alkyl iodides to form alkyl radicals which can then undergo Giese-type addition to electron deficient alkenes (primary *k* = 10^5^ M^−1^ s^−1^, secondary *k* = 10^6^ M^−1^ s^−1^, tertiary *k* = 10^6^–10^8^ M^−1^ s^−1^)^16^ followed by cross coupling of the transient adduct C-radicals with the concomitantly formed persistent 2,2,6,6-tetramethylpiperidine *N*-oxy radical (TEMPO),^17^ steered by the persistent radical effect.^18^ Overall, the cascade would represent a transition metal free alkene 1,2-aminoxyalkylation.

However, there are problems associated with such a reaction design. First, the direct trapping of an alkyl radical generated from an alkyl iodide with the persistent TEMPO (*k* = 10^8^–10^9^ M^−1^ s^−1^)^19^ must be circumvented. As TEMPO is generated *in situ* from TEMPONa, its concentration should be very low throughout the reaction, so that alkyl radical addition to the alkene should become kinetically competitive. Second, direct addition of the alkyl radical to the unreacted aryl diazonium salt (for primary C-radical *k* = 10^6^ M^−1^ s^−1^ and tertiary C-radical *k* ≥ 10^8^ M^−1^ s^−1^),^20^ which is a key step of the transformations depicted in Scheme 1a and b, must be slower than the Giese-type addition. This problem may be addressed by carefully adjusting the concentration of the reaction components and/or by varying the structure of the diazonium salt. Third, it is known^21^ that aryl radicals add efficiently to activated alkenes (*k* = 10^8^ M^−1^ s^−1^). Although fast, such a competing aryl radical addition should be suppressed in the presence of alkyl iodides, as iodine atom abstraction is known to be around one order of magnitude faster (*k* = 10^9^ M^−1^ s^−1^).^22^ Furthermore, the bulkiness of the aryl radical might influence the C–C bond formation to a larger extent than the I-abstraction reaction. Based on these analyses, we were confident that such cascades are feasible and report herein our results on transition metal free 1,2-aminoxyalkylation of alkenes with alkyl iodides by using aryl diazonium salts as stoichiometric XAT-mediators.

## Results and discussion

For reaction optimization, we selected 1-iodoadamantane (1a) as the alkyl radical precursor and methyl acrylate (2a, 5 equiv.) as the acceptor. Freshly prepared TEMPONa (0.85 M in THF, 2.5 equiv.) was added *via* syringe pump over 3 h and the aryldiazonium salt as well as the solvent were varied. Initial experiments were conducted in PhCF_3_. We were pleased to find that the cascade worked as designed and by using 2,4,6-trimethylbenzene diazonium salt 3a (2.5 equiv.), the targeted aminoxyalkylation product 4a was obtained in very good 80% yield (Table 1, entry 1). It is important to note that the aryl diazonium salts (3a–d) are not fully soluble in PhCF_3_, thereby keeping the concentration of soluble 3a–d low in the reaction mixture. Consequently, unwanted direct addition of the reactive 1-adamantyl radical to 3a is suppressed. Other XAT-mediators such as benzene diazonium tetrafluoroborate (3b, 70%), 4-methoxybenzene diazonium tetrafluoroborate (3c, 68%) and 4-(trifluoromethyl)benzene diazonium tetrafluoroborate (3d, 76%) delivered slightly lower yields of 4a under otherwise identical conditions (Table 1, entry 2–4). With CH_3_CN or THF in place of trifluorotoluene as the solvent, yield for 4a dropped to 41% and 75%, respectively (Table 1, entries 5 and 6). The slight reduction of the yield in THF might be caused by competitive HAT (*k* = 10^6^ M^−1^ s^−1^)^21^ from the solvent by the aryl radical, while the decreased yield in CH_3_CN is caused by increased solubility of 3a in CH_3_CN and accordingly alkyl azo compound by product formation (22%) through direct trapping of the 1-adamantyl radical with the aryl diazonium salt 3a.^20^ Lowering the concentration of the aryl diazonium salt and TEMPONa to 2 equiv. each or the alkene concentration to 3 equiv. led to slightly reduced yields (69% and 71%) (Table 1, entries 7 and 8). If TEMPONa was added over a period of 2 h, a significantly lower yield was noted (57%, Table 1, entry 9).

**Table 1:** Reaction optimization^a^

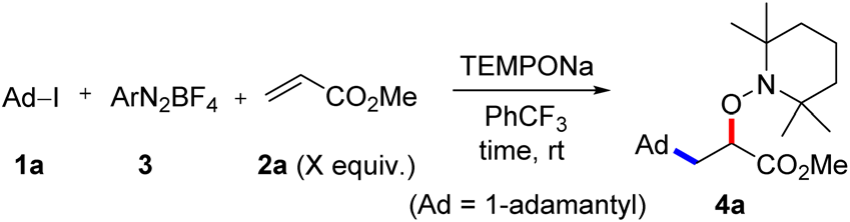						
#	2a (equiv.)	ArN_2_BF_4_	Solvent	TEMPONa (equiv.)	Time (h)	Yield^b^ (%)
**1**	5	3a	**PhCF_3_**	**2.5**	**3**	**80**
2	5	3b	PhCF_3_	2.5	3	70
3	5	3c	PhCF_3_	2.5	3	68
4	5	3d	PhCF_3_	2.5	3	76
5	5	3a	CH_3_CN	2.5	3	41
6	5	3a	THF	2.5	3	75
7	5	3a	PhCF_3_	2	3	69
8	3	3a	PhCF_3_	2.5	3	71
9	5	3a	PhCF_3_	2.5	2	57
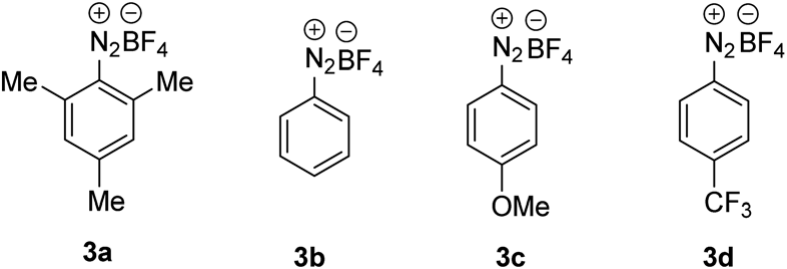						

a Reaction condition: 1a (0.2 mmol), 2a, (3a–d) and solvent (0.4 mL) under Ar, TEMPONa solution in THF was added *via* syringe pump for *t* h at rt.

b NMR yield using 1,3,5-trimethoxy benzene as internal standard.

**Scheme 2:**
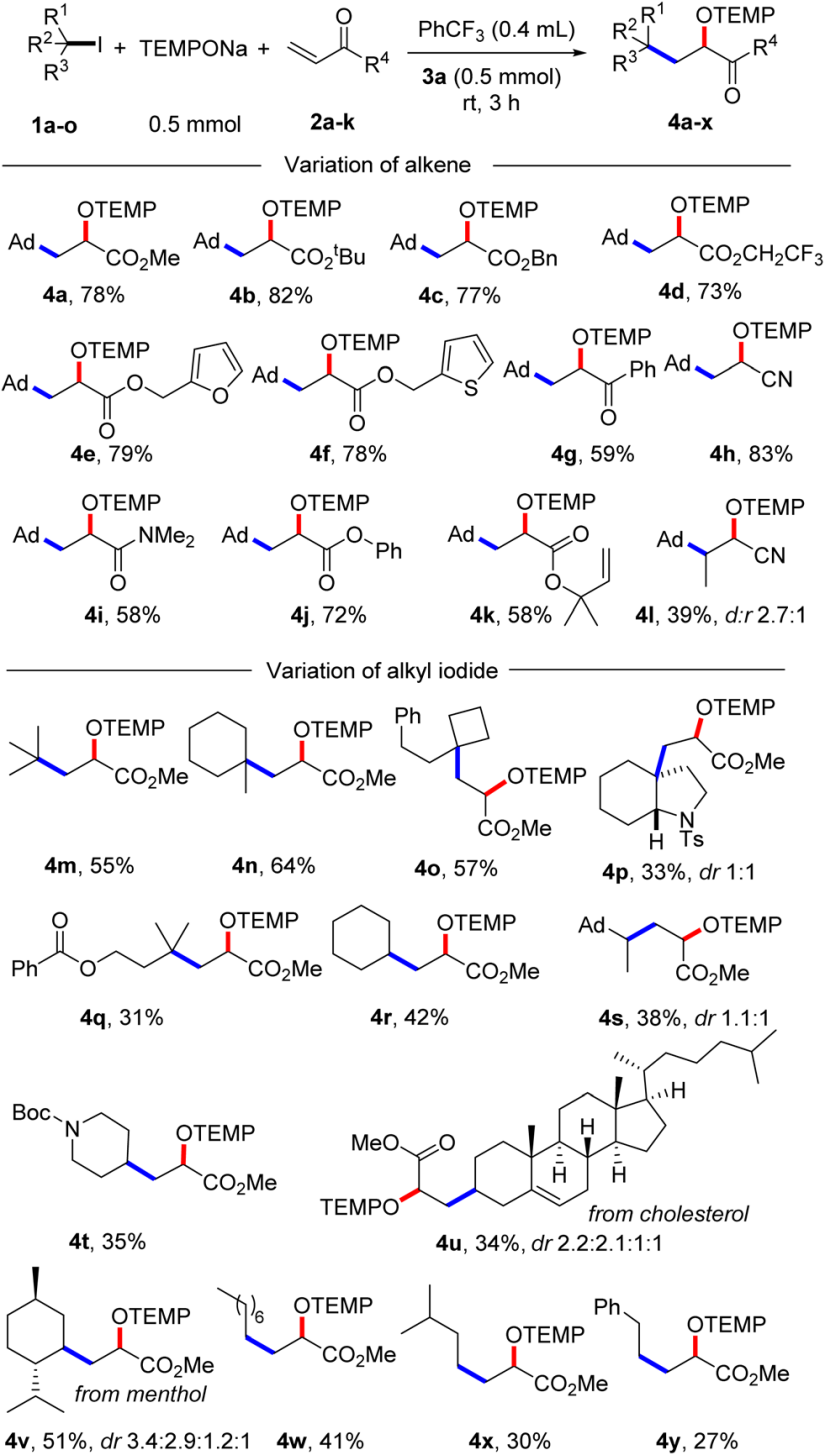
Substrate scope. Variation of alkenes: 1a (0.2 mmol) and 2a–l (5 equiv., 1 mmol). Variation of alkyl iodide: 1a–o (0.2 mmol) and 2a (10 equiv., 2 mmol).

With the optimized condition in hand, studies were continued by addressing the substrate scope. First, the alkene component was varied, keeping 1-iodoadamantane (1a) as the alkyl radical precursor (Scheme 2). Electron deficient alkenes like methyl acrylate, *tert*-butyl acrylate, benzyl acrylate and 2,2,2-trifluoroethyl acrylate all successfully reacted with 1a to give the alkoxyamines 4a–d in good yields (73–82%). Alkoxyamine 4a was successfully prepared on larger scale without compromising the yield (1 mmol, 77% yield, see the ESI†). Heterocyclic ring containing alkenes like 3-furylmethyl acrylate and 3-thienylmethyl acrylate afforded 4e and 4f in 79% and 78% yield, respectively. Other alkenes such as 1-phenylprop-2-en-1-one, acrylonitrile, *N*,*N*-dimethylacrylamide and phenyl acrylate engaged in the cascade to give products 4g–j in moderate to good yields (58–83%). As expected, acceptor 2k bearing an electron-poor as well as an electron-rich alkene moiety reacted chemoselectively with the nucleophilic^23^ 1-adamantyl radical at the electrophilic double bond to afford 4k in 58% yield. A 1,2-disubstituted alkene, but-2-enenitrile, afforded the product 4l in 39% yield with high regioselectivity but low diastereoselectivity. However, styrene and but-3-en-1-ylbenzene did not react with the C-radical generated from 1a under the standard reaction condition.

Next, the alkyl iodide component was varied using methyl acrylate (2a) as the alkyl radical acceptor. Tertiary alkyl iodides like 2-iodo-2-methylpropane and 1-iodo-1-methylcyclohexane afforded the targeted products 4m and 4n in 55% and 64% yield, respectively. Notably, TEMPONa is not basic and competing HI elimination from these tertiary alkyl iodides was not observed, documenting the mildness of the applied condition. Other tertiary alkyl iodides such as (2-(1-iodocyclobutyl)ethyl)benzene, 3a-iodo-1-tosyloctahydro-1*H*-indole and 3-iodo-3-methylbutyl benzoate were eligible C-radical precursors to provide the desired products 4o–q in 31–57% yields. Secondary alkyl iodides can also be used as C-radical precursors in the 1,2-aminoxyalkylation. As examples, cyclohexyl iodide, 1-(1-iodoethyl)adamantane and *tert*-butyl 4-iodopiperidine-1-carboxylate reacted with 2a to the corresponding alkoxyamines 4r–t. However, as compared to the reactions with *tert*-alkyl iodides, lower yields were noted in these cases (35–42%). Cholesterol- and menthol-derived alkyl iodides afforded the desired products 4u and 4v in 34% and 51% yield. We also tested the even more challenging reaction with primary alkyl iodides as C-radical precursors. For these halides, both the XAT to the aryl radical and also the C-radical addition to the acrylate are less efficient. Nevertheless, we were able to realise such transformations and 1-iodooctane, 1-iodo-3-methylbutane as well as (2-iodoethyl)benzene afforded the targeted products 4w–y in moderate yields (27–41%).

After having successfully employed the diazonium salt 3a as an XAT-mediator, we wondered whether stronger oxidizing alkyl iodides directly engage in the 1,2-aminoxyalkylation of alkenes upon reaction with TEMPONa in the absence of salt 3a. We considered electron deficient polyfluoroalkyl iodides and bromides as promising C-radical precursors, as they have significantly less negative cathodic peak potential (peak potentials: *E*_p_ = −1.32 V/SCE for C_6_F_13_I)^24^ than the alkyl iodides addressed above and accordingly might be directly reduced by TEMPONa.

**Scheme 3:**
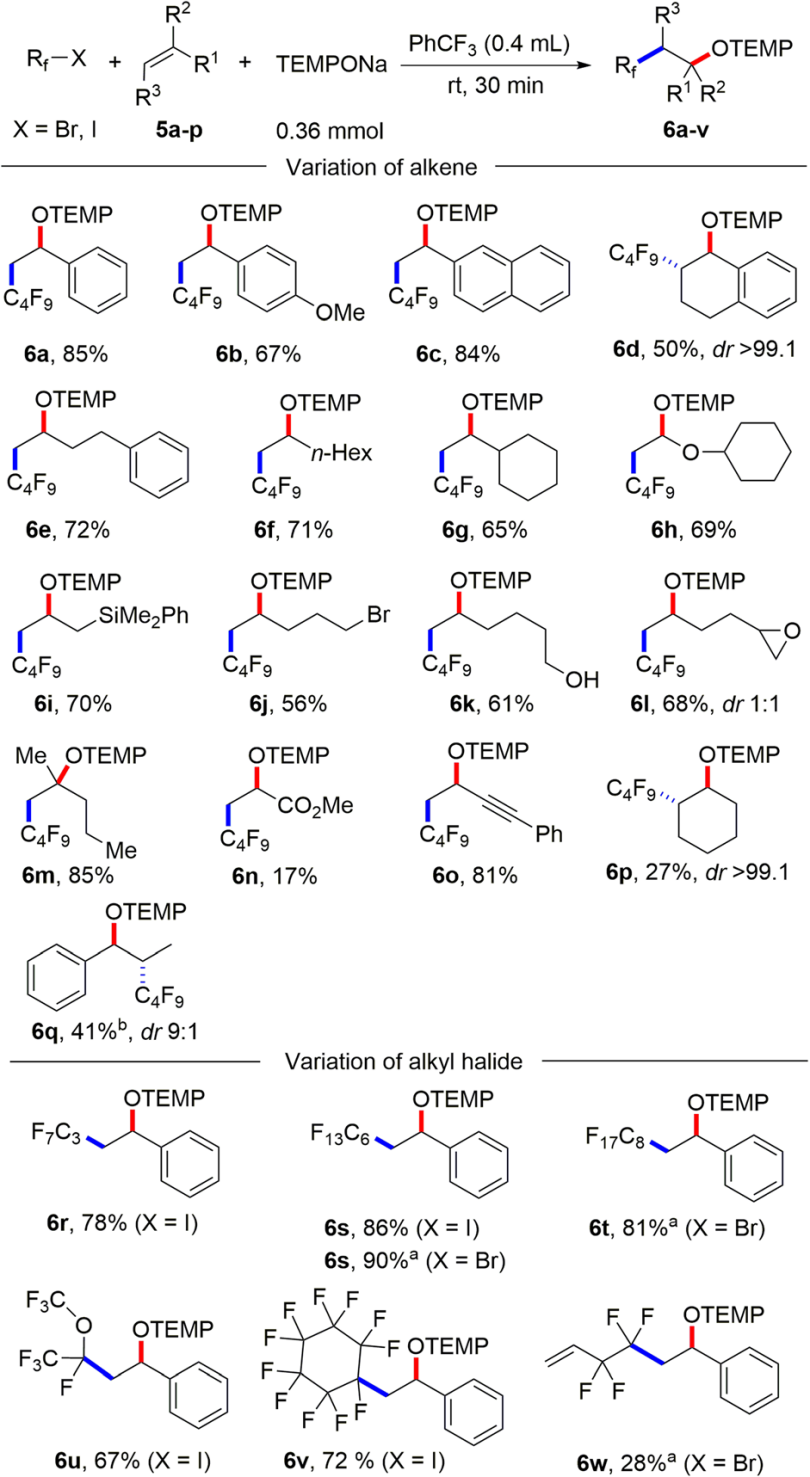
Substrate scope. Variation of alkenes: C_4_F_9_I (0.2 mmol) and styrenes (5 equiv., 1 mmol) or non-activated alkene (10 equiv., 2 mmol). Variation of perfluoroalkyl iodide: R_f_-I (0.2 mmol) and styrene (5 equiv., 1 mmol). ^*a*^R_f_-Br (0.2 mmol) and styrene (5 equiv., 1 mmol) for 18 h. ^*b*^When 10 equiv. styrene was used.

It is well known that perfluoroalkyl radicals react efficiently with styrenes, non-activated alkenes and even electron deficient Michael acceptors.^25^ Pleasingly, we found that 1,2-aminoxyperfluoroalkylation of styrene can be achieved in the absence of any XAT-mediator (Scheme 3). Thus, stirring TEMPONa (0.36 mmol), C_4_F_9_I (0.2 mmol) and styrene (1 mmol) in PhCF_3_ (0.4 mL) for 30 minutes afforded the desired alkoxyamine 6a in 85% yield (for details on reaction optimization, see ESI†). Other styrene derivatives such as 2-vinylnaphthalene, 1-methoxy-4-vinylbenzene and 1,2-dihydronaphthalene also reacted well with C_4_F_9_I, and 6b–d were obtained in moderate to good yields (50–84%). Of note, 1,2-dihydronaphthalene reacted with complete regio- and diastereoselectivity (see 6d). Various non-activated aliphatic alkenes like but-3-en-1-ylbenzene, oct-1-ene, vinylcyclohexane, (vinyloxy)cyclohexane and allyldimethyl(phenyl)silane engaged in this transition metal free alkene 1,2-difunctionalization to give the alkoxyamines 6e–i in good yields (65–72%). A free hydroxy group, a primary alkyl bromide functionality, and an epoxide are all tolerated, as documented by the successful preparation of 6j (56%), 6k (61%) and 6l (68%). 2-Methylpent-1-ene reacted with C_4_F_9_I and TEMPONa in good yield to the tertiary alkoxyamine 6m (85%).

**Scheme 4:**
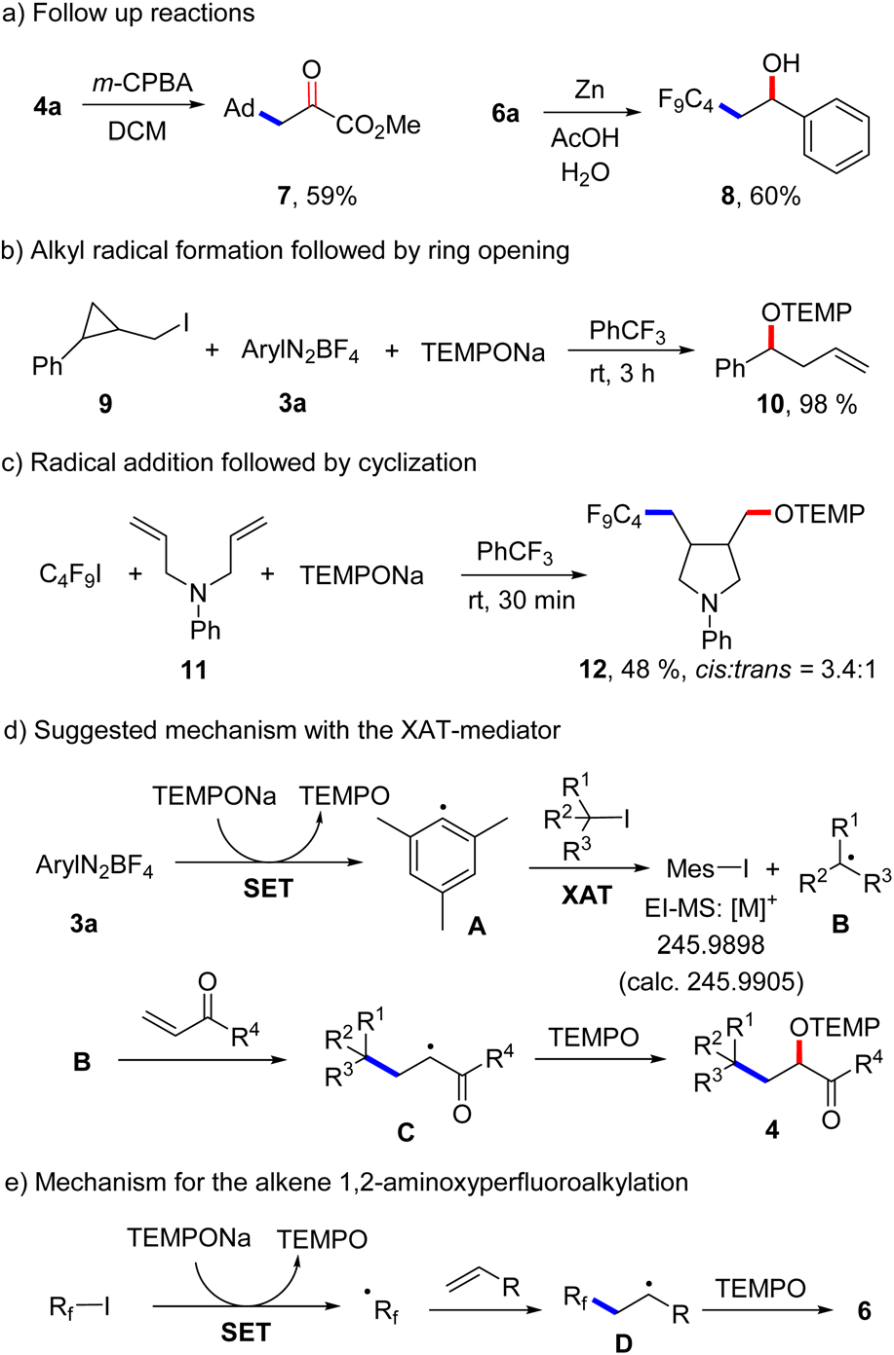
Follow-up chemistry, mechanistic studies and suggested mechanisms.

A low yield was noted for the reaction with the electron poor methyl acrylate as radical acceptor (6n, 17%). With but-3-en-1-yn-1-ylbenzene as the C_4_F_9_-radical acceptor, the 1,2-addition product 6o was formed with complete regiocontrol in 81% yield. Internal alkenes such as cyclohexene and (*Z*)-prop-1-en-1-ylbenzene afforded the desired product 6p and 6q in 27% and 41% yield, respectively. Notably, compound 6p was obtained in complete diastereoselectivity and also a good selectivity was noted for 6q. The relative configuration was assigned in analogy to a previous report.^26^ To document the practicality of our method, compound 6a was successfully prepared on a 3 mmol scale in 82% yield (1.184 g). We then tested various perfluoroalkyl iodides and bromides by using styrene as the R_f_-radical acceptor. With C_3_F_7_I and C_6_F_13_I the products 6r and 6s were obtained in good yields (78%, 86%). Perfluoroalkyl bromides can also be used as C-radical precursors, as shown by the successful transformations of C_6_F_13_Br and C_8_F_17_Br to give 6s and 6t in 90% and 81% yield. Reaction with the bromides were significantly slower and 18 h were required to get full conversion. 1,1,1,2-Tetrafluoro-2-iodo-2-(trifluoromethoxy)ethane and 1,1,2,2,3,3,4,4,5,5,6-undecafluoro-6-iodocyclohexane reacted well with styrene and TEMPONa to afford 6u (67%) and 6v (72%). 4-Bromo-3,3,4,4-tetrafluorobut-1-ene also engaged in the styrene 1,2-difunctionalization, although the desired product 6w was obtained in only 28% yield. Of note, Q. Li *et al.*^27^ and Q. Sun *et al.*^28^ demonstrated cobalt/tertiary-amine-mediated hydroxy-perfluoroalkylation of alkenes using polyfluoroalkyl bromides. In 2022, Yajima and co-workers^29^ also demonstrated light-mediated hydroxy-perfluoroalkylation of styrenes and electron deficient alkenes using enamine and DIPEA (photo-organocatalyst), while no example of a non-activated alkene as acceptor was presented in this report. Compared to previous reports, our method can be conducted under transition metal free conditions and no external reagents like enamine and DIPEA is needed to realize the alkene 1,2-aminoxyperfluoroalkylation. Thus, our method provides a valuable alternative to existing methodology to access similar β-hydroxy-perfluoroalkyl scaffolds. The synthetic value of our product alkoxyamines was documented by two follow-up reactions. The alkoxyamine function in 4a was easily converted to a ketone moiety through *m*-chloroperbenzoic acid (*m*-CPBA) oxidation and the α-keto ester 7 was isolated in 59% yield (Scheme 4a). Such α-keto esters have been used as valuable precursors for a variety of asymmetric transformations^30^ and for the synthesis of heterocycles.^31^ Reduction of the N–O bond in alkoxamine 6a with zinc in aqueous acetic acid provided the alcohol 8 in 60% yield. To support the radical nature of these 1,2-difunctionalizations, typical probe experiments were conducted. First, in order to show that alkyl radicals are generated from alkyl iodides *via* TEMPONa-mediated SET-reduction of 3a followed by XAT, (2-(iodomethyl)cyclopropyl)benzene 9 was subjected to the optimized conditions in the absence of any C-radical acceptor. The cyclopropane ring opening direct TEMPO-trapping product 10 derived from the corresponding cyclopropylmethyl radical was formed in 98% yield (Scheme 4b). Second, C_4_F_9_I was reacted with *N*,*N*-diallylaniline (11) with TEMPONa to afford the perfluoroalkyl radical addition/5-*exo trig* cyclization/TEMPO-trapping product 12 in 48% yield as a mixture of diastereoisomers (Scheme 4c). Both reactions clearly indicate the occurrence of radical intermediates. For reactions conducted with the XAT-mediator 3a, the following mechanism is suggested (Scheme 4d). TEMPONa first reduces the diazonium salt 3a through SET to generate TEMPO along with the mesityl radical A that reacts with the alkyl iodide in an XAT to give the alkyl radical B and Mes–I (detected in HRMS). C-Radical B in turn will add to the alkene acceptor to form the adduct radical C, which is eventually trapped by TEMPO to afford the final product 4. Importantly, TEMPO is present only in very small amounts as it is continuously generated and also consumed, so that radical B can add to the alkene and its direct trapping by TEMPO is not occurring. However, the adduct radical C cannot add to the alkene due to polarity mismatch and consequently selective cross coupling with TEMPO steered by the persistent radical effect^18^ is occurring. Considering the second process, the perfluoroalkyl iodide gets directly reduced by TEMPONa to give TEMPO and the corresponding perfluoroalkyl radical (Scheme 4e). The latter then adds to the alkene and the adduct radical D is finally trapped by TEMPO to give a compound of type 6.

## Conclusions

In summary, we have developed transition metal free 1,2-aminoxyalkylation of various electron-poor alkenes with primary, secondary and tertiary alkyl iodides applying a diazonium salt as a stoichiometric XAT-mediator. TEMPONa is used as mild SET-reductant able to reduce the diazonium salt but not an unactivated alkyl iodide. Interestingly, in the diazonium salt reduction the organic reductant (TEMPONa) is converted into an organic oxidant (TEMPO). 1,2-Aminoxyalkylations with perfluoroalkyl iodides and bromides proceed in the absence of any XAT-mediator, as these electrophilic halides are efficiently SET-reduced with TEMPONa. Both processes can be conducted under mild conditions and show good functional group tolerance. The alkoxyamine functional group present in the products can be easily oxidized or reduced to the corresponding ketone and alcohol functionalities.

## Data availability

The data that support the findings of this study are available in the ESI.†

## Author contributions

A. M. conducted all experiments and characterized the novel compounds. A. M. and A. S. designed the experiments and wrote the manuscript.

## Conflicts of interest

There are no conflicts to declare.

## Supplementary Material

SC-014-D3SC02544F-s001
