# Supplementary material for: 1,2-Aminoxyalkylation of alkenes with alkyl iodides and TEMPONa through SET- and XAT-processes
Source: Chem Sci. 2023 Jun 23;14(28):7675–80. doi: 10.1039/d3sc02544f (PMC10355098; doi:10.1039/d3sc02544f)

Electronic Supplementary Material (ESI) for Chemical Science.
This journal is © The Royal Society of Chemistry 2023

Supporting information

1,2-Aminoxyalkylation of Alkenes with Alkyl Iodides and TEMPONa through SET- and XAT-Processes

*Anirban Maity and Armido Studer**

1. General ………..

2. General procedure for the preparation of TEMPONa solution (GP 1) …………………………………………………………

3. General procedure for 1,2-aminoxyalkylation of alkenes by nonactivated alkyl iodides (GP 2) ……………………

4. General procedure for 1,2-aminoxyalkylation of an alkene by perfluoroalkyl halides (GP 3) ………………………

5. Procedure for large scale synthesis……

6. Substrate structure ………..

7. Optimization of the reaction condition ……………………………………………………………………………………………….

8. Mechanistic experiment ……..

9. Physical data of the products……

10. References ……….

11. NMR data …….

1. General

All reactions that are air and moisture sensitive were performed in oven-heated glassware under argon atmosphere by using Schlenk-technique. Anhydrous tetrahydrofuran (THF) was refluxed over elemental Na and freshly distilled from K metal before use. Anhydrous dichloromethane (CH_2Cl_2) was dried over P_4O_{10} and freshly distilled before use. All reagents were purchased from Sigma Aldrich, Acros Organics, ABCR, TCI, Alfa Aesar, BLDPharm and Fluorochem and were used without any further purification. Thin layer chromatography (TLC) was performed on Merck silica gel 60 F-254 plates and visualized by fluorescence quenching under UV light or staining with $KMnO_4$ (1.5 g in 400 mL H_2O, 5 g $NaHCO_3$). Solvents for column chromatography were purchased in technical grade and purified by distillation prior to use. Column chromatography was performed on Merck or VWR silica gel 60 (40- 63 µm) using a compress air pressure of 0.2 bar. ^{1}H NMR, ^{13}C NMR and ^{19}F NMR spectra were measured on DPX 300, AV 400 or 500 at 300 K and chemical shift (δ) is expressed in ppm unit. Coupling constants were reported in Hertz (Hz), singlet is defined as s; broad singlet as brs; doublet as d; triplet as t; quartet as q; doublet of doublet as dd; triplate of triplate as tt; multiplet as m. HRMS (ESI-MS) spectra were measured on a Thermo Fisher Scientific LTQ XL Orbitrap and Thermo Fisher Scientific Orbitrap Velos Pro spectrometer. Infrared spectra (IR) were measured on a Jasco FT/IR-4600 spectrometer and bands are given by wavenumber (cm^{-1}). Melting points were measured by Büchi Melting Point *M-560*.

General procedure

2. General procedure for the preparation of TEMPONa solution (GP 1):

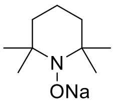

TEMPONa was prepared according to a literature known[1] procedure. A Schlenk tube was charged with freshly cleaned (with dry pentane) elemental sodium metal (0.44 g, 19 mmol, 1.4 equiv.) which was then melted to sodium mirror using a heat gun at 200 °C. After that the Schlenk tube was cooled to rt, TEMPO (2.12 g, 13.6 mmol, 1 equiv.), naphthalene (170 mg, 0.70 mmol, 0.1 equiv.) and dry THF (16 mL) were added under argon. The reaction mixture was stirred at room temperature until a blue-black colour persisted (generally 1-2 h). TEMPONa solution can be stored under argon for several days in the fridge without any observable decomposition.

3. General procedure for 1,2-aminoxyalkylation of alkenes by unactivated alkyl iodides (GP 2):

The TEMPONa-solution (0.59 mL, 2.5 equiv., 0.85M in THF) was added dropwise via syringe pump over the period of 3 h to a flame dried Schlenk-tube containing the alkyl iodide (0.2 mmol 1.0 equiv.), 2,4,6-trimethylbenzenediazonium tetrafluoroborate **3a** (117 mg, 0.5 mmol, 2.5 equiv.) and the alkene (eq. is mentioned in the text) in α,α,α-trifluorotoluene (0.4 mL) at room temperature. Then, the reaction mixture was evaporated in rotavapor and directly purified by flash column chromatography on silica gel to obtain the desired product.

4. General procedure for 1,2-aminoxyalkylation of an alkene by perfluoroalkyl iodides (GP 3):

The TEMPONa-solution (0.43 mL, 1.8 equiv., 0.85M in THF) was added dropwise to a flame dried Schlenk-tube containing the polyfluoroalkyl iodide (0.2 mmol 1.0 equiv.) and the alkene (eq. is mentioned in the text) in α,α,α-trifluorotoluene (0.4 mL). The reaction mixture was then stirred for additional 30 minutes at room temperature. Then, the reaction mixture was evaporated in rotavapor and directly purified by flash column chromatography on silica gel.

5. Procedure for large scale synthesis

5.1 Large scale synthesis of methyl 3-(adamantan-1-yl)-2-((2,2,6,6-tetramethylpiperidin-1-yl)oxy)propanoate (4a):

The TEMPONa-solution (2.95 mL, 2.5 mmol, 2.5 equiv., 0.85M in THF) was added dropwise under argon via syringe pump over the period of 3 h to a flame dried Schlenk-tube containing the 1-iodoadamantane (262.5 mg, 1 mmol, 1.0 equiv.), 2,4,6-trimethylbenzenediazonium tetrafluoroborate **3a** (585 mg, 2.5 mmol, 2.5 equiv.) and the methyl acrylate (450 µL, 5 mmol, 5 equiv.) in α,α,α-trifluorotoluene (2 mL) at room temperature. Then, water

was added to the reaction mixture and organic layer was extracted with Et_2O (3x20 mL). Organic layer then dried over $MgSO_4$ evaporated in rotavapor. Flash column chromatography (pentane/Et_2O, 100/0 to 98/2) on silica gel to obtain the desired product **4a** as a white solid (290 mg, 77%).

5.2 Large scale synthesis of 2,2,6,6-tetramethyl-1-((3,3,4,4,5,5,6,6,6-nonafluoro-1-phenylhexyl)oxy)piperidine (6a):

The TEMPONa-solution (6.45 mL, 5.4 mmol, 1.8 equiv., 0.85M in THF) was added dropwise under argon to a flame dried Schlenk-tube containing the $^nC_4F_9I$ (515 µL, 3 mmol 1.0 equiv.) and styrene (1.7 mL, 15 mmol, 5 equiv.) in α,α,α-trifluorotoluene (6 mL). The reaction mixture was then stirred for additional 30 minutes at room temperature. Then, water was added to the reaction mixture and organic layer was extracted with Et_2O (3x20 mL). Organic layer was dried over $MgSO_4$ and was evaporated in rotavapor. Flash column chromatography (pentane/Et_2O, 100/0 to 97/3) on silica gel to obtain the desired product **6a** as a colourless liquid (1.18 g, 82%).

6. Substrate structure:

Alkyl iodides:

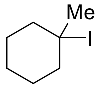
1c

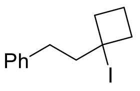
1d

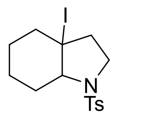
1e

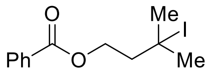
1f

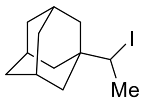
1g

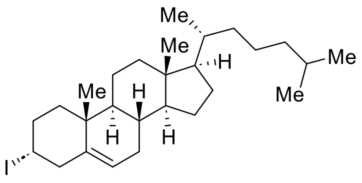
1h

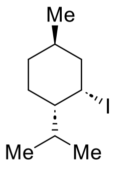
1i

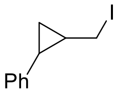
9

Alkenes:

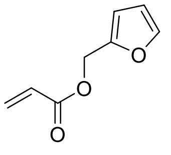
2e

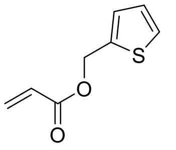
2f

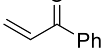
2g

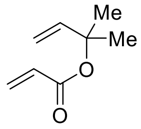
2k

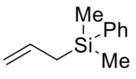
5i

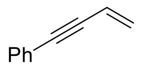
5o

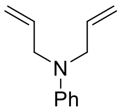
11

Aryl diazonium salts:

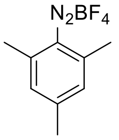
3a

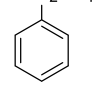
3b

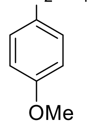
3c

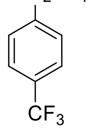
3d

Alkyl iodide **1c**, **1d**, **1e**, **1f**, **1g**, are all known compounds and were synthesized according to literatre known procedure.[2] Compounds **1h** and **1i** were prepared according to known method.[3] Alkene **9** was synthesized according to known literatute procedure.[4] Alkene **2e**[5], **2f**[6], **2g**[7], **2k**[8], **5i**[9], **5o**[10] and **11**[11] are literarure known compounds. Compound **3a** is literature reported.[12] Compounds **3b**, **3c** and **3d** were prepared by literature known[13] method.

7. Optimization of the reaction conditions for GP 3:

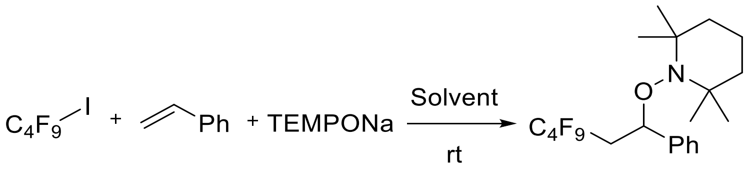

entry	alkene	TEMPONa	solvent	Time	yield (%)[a]
1	5 eq	2.2 eq	$PhCF_3$	2 h	87
2	5 eq	2.2 eq	CH_3CN	2 h	79
3	5 eq	2.2 eq	THF	2 h	83
4	**5 eq**	**1.8 eq**	**$PhCF_3$**	**30 m**	**87**
5	2 eq	1.2 eq	$PhCF_3$	2 h	68
6	3 eq	2.2 eq	$PhCF_3$	2 h	75

[a]NMR yield using 1,3,5-trimethoxy benzene as internal standard

8. Mechanistic experiment

Ring opening experiment of (2-(iodomethyl)cyclopropyl)benzene **9**:

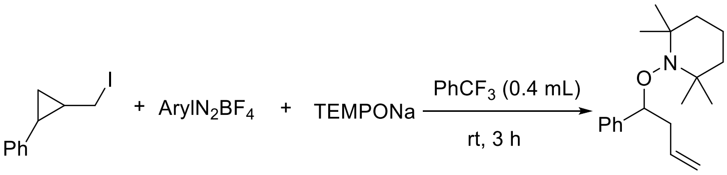

9 **3a**, 0.5 mmol 0.5 mmol **10**, 98%

According to **GP 2** TEMPONa-solution (0.59 mL, 2.5 equiv., 0.85M in THF) was added dropwise via syringe pump over the period of 3 h into a flame dried Schlenk-tube containing **9** (51.6 mg, 0.20 mmol, 1 equiv.), 2,4,6-trimethylbenzenediazonium tetrafluoroborate **3a** (117 mg, 0.50 mmol, 2.5 equiv.) in absence of alkene in α,α,α-trifluorotoluene (0.4 mL) at room temperature. Then the reaction mixture was evaporated in rotavapor and directly purified by Flash column chromatography (pentane/Et_2O, 100/0 to 200/1) afforded 2,2,6,6-tetramethyl-1-((1-phenylbut-3-en-1-yl)oxy)piperidine **10** (56.3 mg, 98%) as a colourless liquid.

2,2,6,6-Tetramethyl-1-((1-phenylbut-3-en-1-yl)oxy)piperidine (**10**)

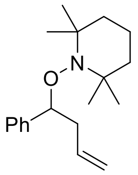

^{1}H NMR (300 MHz, $CDCl_3$) δ 7.25 – 7.12 (m, 5H), 5.65 – 5.31 (m, 1H), 4.94 – 4.78 (m, 2H), 4.60 (dd, *J* = 9.5, 4.3 Hz, 1H), 2.89 – 2.72 (m, 1H), 2.60 – 2.43 (m, 1H), 1.51 – 1.37 (m, 3H), 1.33 – 1.18 (m, 6H), 1.16 – 1.06 (m, 3H), 0.95 (s, 3H), 0.54 (s, 3H). **^{13}C NMR** (151 MHz, $CDCl_3$) δ 143.1, 134.7, 127.8, 127.7, 127.0, 116.8, 86.8, 60.0, 59.6, 40.8, 40.5, 34.4, 34.1, 20.4, 17.2. **HRMS** (**ESI**): $[M+H]^+$ Calcd. for $C_{19}H_{29}NOH$ 288.2321; Found: 288.2323. **FTIR** (neat): ν(cm^{-1}) 3064, 3003, 2972, 2928, 2871, 1640, 1493, 1453, 1375, 1360, 1302, 1258, 1241, 1207, 1182, 1132, 1074, 1006, 984, 957, 912, 877, 796, 760, 711, 697, 647, 627, 614, 578, 541, 506.

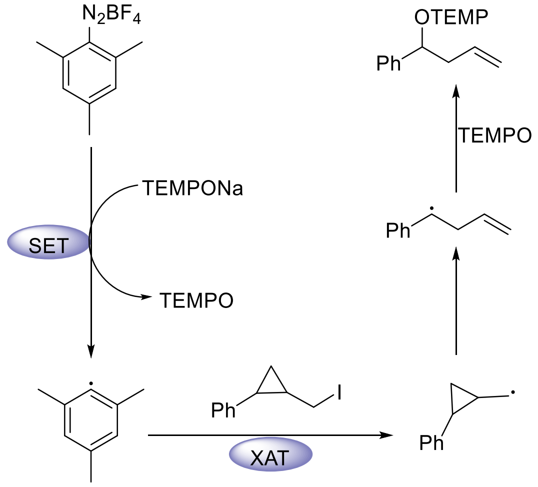

Scheme 1: Proposed reaction mechanism for ring opening experiment of **9**.

Side product **13** formation in CH_3CN:

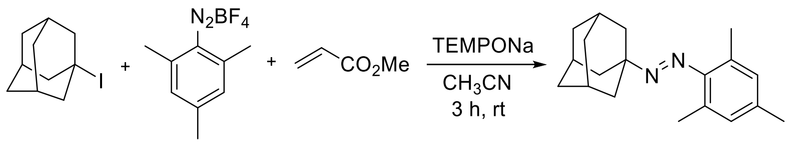

1a **3a** (0.5mmol) **2a** (1 mmol) **13**, 22%

The reaction was performed according to the **GP 2** with 1-iodoadamantane **1a** (52.5 mg, 0.20 mmol, 1 equiv.) and methyl acrylate **2a** (90 µL, 1.0 mmol, 5 equiv.) in CH_3CN. Flash column chromatography (pentane/Et_2O, 100/0 to 200/1) afforded (E)-1-(adamantan-1-yl)-2-mesityldiazene **13** (12.5 mg, 22%) as a yellow oil.

(E)-1-(Adamantan-1-yl)-2-mesityldiazene (**13**)

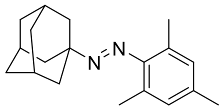

^{1}H NMR (300 MHz, CD_3COCD_3) δ 6.76 – 6.60 (m, 2H), 2.02 – 1.97 (m, 3H), 1.90 (s, 6H), 1.87 – 1.81 (m, 2H), 1.75 (d, *J* = 2.9 Hz, 6H), 1.67 – 1.50 (m, 7H). **^{13}C NMR** (126 MHz, CD_3COCD_3) δ 150.5, 137.3, 130.3, 129.7, 70.5, 41.6, 37.5, 21.1, 18.4. **HRMS** (**ESI**): $[M+Na]^+$ Calcd. for $C_{19}H_{26}N_2Na$ 305.1988; Found: 305.1987. **FTIR** (neat): ν(cm^{-1}) 2903, 2849, 1735, 1608, 1450, 1375, 1344, 1307, 1260, 1214, 1182, 1102, 1086, 1033, 974, 935, 853, 812, 732, 647, 580.

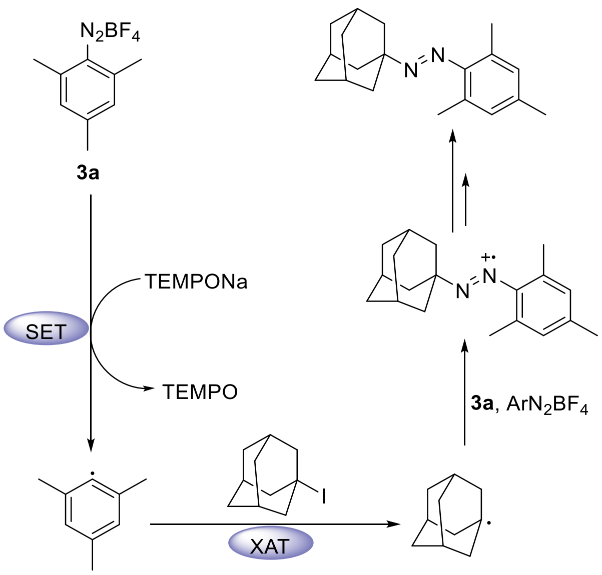

Scheme 3: Proposed reaction mechanism for formation of side product **13**

Ring cyclization experiment of N,N-diallylaniline **11**:

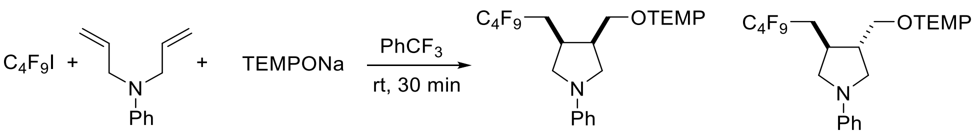

The reaction was performed according to the **GP 3** with $^{n}C_4F_9I$ **1a** (35 µL, 0.20 mmol, 1 equiv.) and N,N-diallylaniline (346 mg, 2.0 mmol, 10 equiv.). Flash column chromatography (pentane/Et_2O, 100/0 to 96/4) and (pentane/Et_2O, 100/0 to 95/5) afforded **12a (**42 mg, 37%**)** and **12b** (12 mg, 11%) both as colourless oils.

***Cis*-2,2,6,6-tetramethyl-1-((4-(2,2,3,3,4,4,5,5,5-nonafluoropentyl)-1-phenylpyrrolidin-3-yl)methoxy)piperidine** (**12a**)

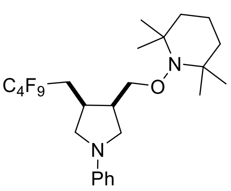

^{1}H NMR (500 MHz, $CDCl_3$) δ 7.29 – 7.21 (m, 2H), 6.71 (t, *J* = 7.3 Hz, 1H), 6.62 – 6.53 (m, 2H), 3.91 (dd, *J* = 9.4, 7.0 Hz, 1H), 3.78 (dd, *J* = 9.3, 7.1 Hz, 1H), 3.56 (dd, *J* = 9.3, 7.4 Hz, 1H), 3.45 (dd, *J* = 9.5, 6.5 Hz, 1H), 3.35 (dd, *J* = 9.5, 3.2 Hz, 1H), 3.23 (t, *J* = 8.7 Hz, 1H), 2.90 – 2.78 (m, 1H), 2.76 – 2.66 (m, 1H), 2.56 – 2.47 (m, 1H), 2.32 – 2.18 (m, 1H), 1.64 – 1.52 (m, 1H), 1.50 – 1.40 (m, 4H), 1.37 – 1.31 (m, 1H), 1.17 (s, 3H), 1.12 (d, *J* = 3.0 Hz, 6H), 1.08 (s, 3H). **^{13}C{^{19}F} NMR** (126 MHz, $CDCl_3$) δ 147.0, 128.8, 118.1, 117.0, 115.5, 111.1, 110.0, 108.4, 75.1, 59.5, 59.4, 51.5, 49.8, 40.5, 39.3, 32.8, 32.7, 32.6, 29.6, 19.7, 19.6, 16.6. **^{19}F NMR** (470 MHz, $CDCl_3$) δ -81.02 (tt, *J* = 9.7, 3.2 Hz, 3F), -111.92 – -113.68 (m, 1F), -114.18 – -115.77 (m, 1F), -123.86 – -124.96 (m, 2F), -125.28 – -126.84 (m, 2F). **HRMS** (**ESI**): $[M+H]^+$ Calcd for $C_{25}H_{33}N_{2}OF_9H$ 549.2521; Found: 549.2519. **FTIR** (neat): ν(cm^{-1}) 2974, 2930, 1685, 1599, 1507, 1483, 1374, 1359, 1299, 1218, 1184, 1131, 1046, 1022, 992, 957, 907, 879, 790, 745, 707, 690, 592, 557, 532, 510.

***Trans* -2,2,6,6-tetramethyl-1-((4-(2,2,3,3,4,4,5,5,5-nonafluoropentyl)-1-phenylpyrrolidin-3-yl)methoxy)piperidine** (**12b**)

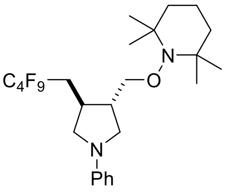

^{1}H NMR (300 MHz, $CDCl_3$) δ 7.34 – 7.27 (m, 2H), 6.75 (t, *J* = 7.3 Hz, 1H), 6.62 (d, *J* = 8.0 Hz, 2H), 4.08 – 3.88 (m, 2H), 3.78 (t, *J* = 8.9 Hz, 1H), 3.51 (t, *J* = 8.7 Hz, 1H), 3.25 – 3.06 (m, 2H), 2.98 – 2.71 (m, 1H), 2.70 – 2.52 (m, 1H), 2.47 – 2.30 (m, 1H), 2.29 – 1.98 (m, 1H), 1.61 – 1.45 (m, 5H), 1.39 (d, *J* = 14.3 Hz, 1H), 1.22 (d, *J* = 6.4 Hz, 6H), 1.14 (d, *J* = 3.8 Hz, 6H). **^{13}C{^{19}F} NMR** (126 MHz, $CDCl_3$) δ 147.3, 129.1, 118.3, 117.3, 116.1, 111.6, 110.3, 108.7, 77.8, 59.8, 54.0, 49.7, 43.5, 39.6, 35.2, 34.0, 33.1, 32.9, 29.6, 20.0, 17.0. **^{19}F NMR** (470 MHz, $CDCl_3$) δ -81.02 (tt, *J* = 9.7, 3.2 Hz, 3F), -111.19 – -113.24 (m; 1F), -113.37 – -115.45 (m, 1F), -123.58 – -125.17 (m, 2F), -125.18 – -126.66 (m, 2F). **HRMS** (**ESI**): $[M+H]^+$ Calcd for $C_{25}H_{33}N_{2}OF_9H$ 549.2521;

Found: 549.2521. **FTIR** (neat): ν(cm^{-1}) 2974, 2928, 1710, 1599, 1507, 1481, 1374, 1356, 1298, 1218, 1184, 1131, 1019, 997, 972, 956, 927, 906, 879, 861, 804, 746, 734, 712, 690, 648, 612, 591, 530, 510.

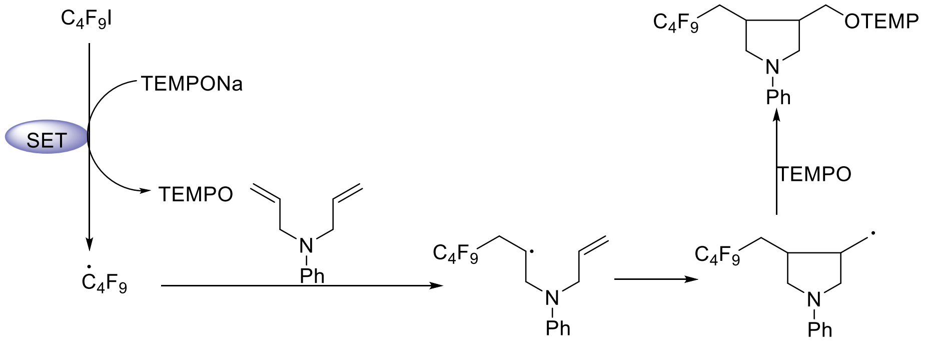

Scheme 2: Proposed reaction mechanism for radical cyclization of **11**.

9. Physical data for the products

Methyl 3-(adamantan-1-yl)-2-((2,2,6,6-tetramethylpiperidin-1-yl)oxy)propanoate (4a)

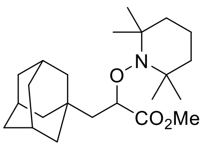

The reaction was performed according to the **GP 2** with 1-iodoadamantane (52.5 mg, 0.20 mmol, 1 equiv.) and methyl acrylate (90 µL, 1.0 mmol, 5 equiv.). Flash column chromatography (pentane/Et_2O, 100/0 to 98/2) afforded methyl 3-(adamantan-1-yl)-2-((2,2,6,6-tetramethylpiperidin-1-yl)oxy)propanoate **4a** (59 mg, 78%) as a white solid. **MP**: 67-69 °C. **^{1}H NMR** (500 MHz, $CDCl_3$) δ 4.23 (dd, *J* = 11.6, 2.6 Hz, 1H), 3.66 (s, 3H), 1.92 – 1.87 (m, 3H), 1.82 (dd, *J* = 13.8, 11.6 Hz, 1H), 1.69 – 1.62 (m, 3H), 1.61 – 1.54 (m, 4H), 1.54 – 1.50 (m, 2H), 1.50 – 1.36 (m, 9H), 1.31 – 1.23 (m, 1H), 1.18 (s, 3H), 1.09 (s, 3H), 1.04 (s, 3H), 0.96 (s, 3H). **^{13}C NMR** (126 MHz, $CDCl_3$) δ 174.6, 81.4, 60.6, 59.1, 51.2, 46.4, 42.5, 42.4, 42.2, 40.2, 40.0, 37.0, 36.8, 36.7, 33.8, 32.7, 31.4, 28.7, 28.6, 28.4, 20.3, 20.0, 17.1. **HRMS** (**ESI**): [M+Na]$^+$ Calcd. for $C_{23}H_{39}NO_3Na$ 400.2822; Found: 400.2821. **FTIR** (neat): ν(cm^{-1}) 2900, 2846, 1742, 1451, 1376, 1362, 1266, 1246, 1211, 1196, 1161, 1133, 1106, 1077, 1049, 1019, 981, 957, 920, 876, 843, 795, 728, 710, 677, 648, 629, 560, 504.

tert-Butyl 3-(adamantan-1-yl)-2-((2,2,6,6-tetramethylpiperidin-1-yl)oxy)propanoate (4b)

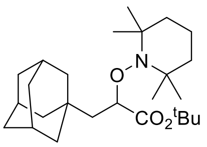

The reaction was performed according to the **GP 2** with 1-iodoadamantane (52.5 mg, 0.20 mmol, 1 equiv.) and tert-butyl acrylate (146 µL, 1.0 mmol, 5 equiv.). Flash column chromatography (pentane/Et_2O, 100/0 to 99/1) afforded *tert*-butyl 3-(adamantan-1-yl)-2-((2,2,6,6-tetramethylpiperidin-1-yl)oxy)propanoate **4b** (68.9 mg, 82%) as a white solid. **MP**: 89-91 °C. **^{1}H NMR** (300 MHz, $CDCl_3$) δ 4.16 (dd, *J* = 10.3, 2.9 Hz, 1H), 1.96 – 1.88 (m, 3H), 1.79 – 1.55 (m, 9H), 1.52 (s, 6H), 1.48 – 1.37 (m, 14H), 1.19 (s, 3H), 1.13 – 1.03 (m, 9H). **^{13}C NMR** (151 MHz, $CDCl_3$) δ 173.3, 82.3, 80.9, 60.2, 59.2, 47.2, 42.8, 40.3, 36.9, 34.2, 34.0, 31.5, 28.6, 28.1, 20.3, 20.2, 17.1. **HRMS** (**ESI**): [M+Na]$^+$ Calcd. for $C_{26}H_{45}NO_3Na$ 442.3291; Found: 442.3283. **FTIR** (neat): ν(cm^{-1}) 2976, 2903, 2848, 1739, 1452, 1364, 1147, 845, 800, 515.

Benzyl 3-(adamantan-1-yl)-2-((2,2,6,6-tetramethylpiperidin-1-yl)oxy)propanoate (4c)

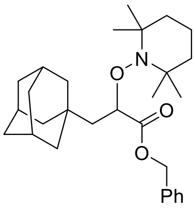

The reaction was performed according to the **GP 2** with 1-iodoadamantane (52.5 mg, 0.20 mmol, 1 equiv.) and benzyl acrylate (153 µL, 1.0 mmol, 5 equiv.). Flash column chromatography (pentane/Et_2O, 100/0 to 98/2) afforded benzyl 3-(adamantan-1-yl)-2-((2,2,6,6-tetramethylpiperidin-1-yl)oxy)propanoate **4c** (70 mg, 77%) as a white solid. **MP**: 85-87 °C. **^{1}H NMR** (599 MHz, $CDCl_3$) δ 7.43 – 7.39 (m, 2H), 7.38 – 7.30 (m, 3H), 5.17 (d, *J* = 12.2 Hz, 1H), 5.05 (d, *J* = 12.3 Hz, 1H), 4.28 (dd, *J* = 11.4, 2.7 Hz, 1H), 1.87 – 1.79 (m, 4H), 1.66 – 1.57 (m, 4H), 1.56 – 1.49 (m, 4H), 1.49 – 1.34 (m, 10H), 1.32 – 1.25 (m, 1H), 1.19 (s, 3H), 1.11 (s, 3H), 1.06 (s, 3H), 1.01 (s, 3H). **^{13}C NMR** (151 MHz, $CDCl_3$) δ 173.9, 135.4, 128.9, 128.4, 128.2, 81.6, 66.2, 60.5, 59.1, 46.5, 42.4, 40.3, 40.1, 36.8, 33.8, 33.0, 31.3, 28.5, 20.3, 20.1, 17.1. **HRMS** (**ESI**): [M+Na]$^+$ Calcd. for

$C_{29}H_{43}NO_3Na$ 476.3135; Found: 476.3129. **FTIR** (neat): ν(cm^{-1}) 2900, 2846, 1743, 1497, 1453, 1375, 1362, 1300, 1260, 1243, 1210, 1175, 1153, 1134, 1106, 1076, 1048, 1016, 974, 957, 918, 876, 794, 778, 750, 731, 696, 630, 600, 506.

2,2,2-Trifluoroethyl 3-(adamantan-1-yl)-2-((2,2,6,6-tetramethylpiperidin-1-yl)oxy)propanoate (**4d**)

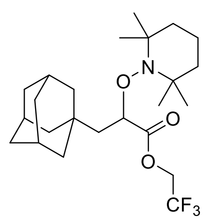

The reaction was performed according to the **GP 2** with 1-iodoadamantane (52.5 mg, 0.20 mmol, 1 equiv.) and 2,2,2-trifluoroethyl acrylate (126 µL, 1.0 mmol, 5 equiv.). Flash column chromatography (pentane/Et$_2$O, 100/0 to 98/2) afforded 2,2,2-trifluoroethyl 3-(adamantan-1-yl)-2-((2,2,6,6-tetramethylpiperidin-1-yl)oxy)propanoate **4d** (65 mg, 73%) as a white solid. **MP**: 104-106 °C. **^{1}H NMR** (500 MHz, CDCl$_3$) δ 4.51 – 4.31 (m, 3H), 1.95 – 1.88 (m, 3H), 1.82 (dd, *J* = 14.0, 11.1 Hz, 1H), 1.70 – 1.57 (m, 7H), 1.56 – 1.34 (m, 11H), 1.33 – 1.24 (m, 1H), 1.20 (s, 3H), 1.11 (s, 3H), 1.05 (s, 3H), 0.95 (s, 3H). **^{13}C NMR** (76 MHz, CDCl$_3$) δ 172.6, 123.1 (q, *J* = 275 Hz), 80.9, 60.6, 60.5 (q, *J* = 36 Hz), 59.2, 46.5, 42.4, 40.2, 40.1, 36.8, 33.9, 32.8, 31.4, 28.5, 20.2, 20.1, 17.0. **^{19}F NMR** (282 MHz, CDCl$_3$) δ -72.94 (s, 3F). **HRMS** (**ESI**): [M+Na]$^+$ Calcd. for $C_{24}H_{38}NO_3F_3Na$ 468.2696; Found: 468.2692. **FTIR** (neat): ν(cm^{-1}) 2975, 2906, 2847, 1760, 1452, 1415, 1375, 1362, 1279, 1241, 1209, 1171, 1135, 1108, 1078, 1051, 1019, 974, 957, 909, 876, 843, 795, 778, 763, 729, 714, 687, 638, 570, 556, 505.

Furan-2-ylmethyl 3-(adamantan-1-yl)-2-((2,2,6,6-tetramethylpiperidin-1-yl)oxy)propanoate (**4e**)

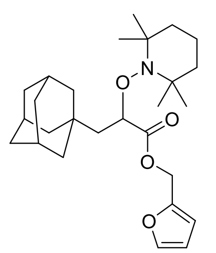

The reaction was performed according to the **GP 2** with 1-iodoadamantane (52.5 mg, 0.20 mmol, 1 equiv.) and furan-2-ylmethyl acrylate (152 mg, 1.0 mmol, 5 equiv.). Flash column chromatography (pentane/Et$_2$O, 100/0 to 98/2) afforded furan-2-ylmethyl 3-(adamantan-1-yl)-2-((2,2,6,6-tetramethylpiperidin-1-yl)oxy)propanoate **4e** (70 mg, 79%) as a white solid. **MP**: 99-100 °C. **^{1}H NMR** (300 MHz, CDCl$_3$) δ 7.39 (dd, *J* = 1.8, 0.9 Hz, 1H), 6.44 – 6.37 (m, 1H), 6.33 (dd, *J* = 3.3, 1.8 Hz, 1H), 5.14 (d, *J* = 13.1 Hz, 1H), 4.98 (d, *J* = 13.1 Hz, 1H), 4.23 (dd, *J* = 11.5, 2.7 Hz, 1H), 1.89 – 1.75 (m, 4H), 1.68 – 1.49 (m, 8H), 1.48 – 1.31 (m, 10H), 1.30 – 1.23 (m, 1H), 1.17 (s, 3H), 1.09 (s, 3H), 1.03 (s, 3H), 0.97 (s, 3H). **^{13}C NMR** (126 MHz, CDCl$_3$) δ 173.7, 149.1, 143.0, 111.1, 110.5, 81.5, 60.5, 59.0, 57.7, 46.6, 42.3, 40.2, 40.1, 36.8, 33.8, 32.8, 31.3, 28.6, 20.2, 20.0, 17.1. **HRMS** (**ESI**): [M+Na]$^+$ Calcd. for $C_{27}H_{41}NO_4Na$ 466.2927; Found: 466.2927. **FTIR** (neat): ν(cm^{-1}) 2900, 2846, 1742, 1501, 1451, 1376, 1362, 1349, 1275, 1260, 1243, 1210, 1174, 1153, 1106, 1078, 1047, 1016, 1002, 973, 957, 916, 885, 798, 748, 708, 672, 647, 623, 599, 561, 529.

Thiophen-2-ylmethyl 3-(adamantan-1-yl)-2-((2,2,6,6-tetramethylpiperidin-1-yl)oxy)propanoate (**4f**)

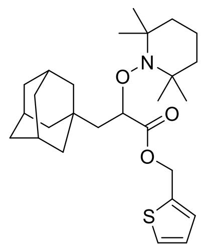

The reaction was performed according to the **GP 2** with 1-iodoadamantane (52.5 mg, 0.20 mmol, 1 equiv.) and thiophen-2-ylmethyl acrylate (168 mg, 1.0 mmol, 5 equiv.). Flash column chromatography (pentane/Et$_2$O, 100/0 to 98/2) afforded thiophen-2-ylmethyl 3-(adamantan-1-yl)-2-((2,2,6,6-tetramethylpiperidin-1-yl)oxy)propanoate **4f** (71.7 mg, 78%) as a white solid. **MP**: 112-114 °C. **^{1}H NMR** (500 MHz, CDCl$_3$) δ 7.30 (dd, *J* = 5.1, 1.2 Hz, 1H), 7.11 (dd, *J* = 3.5, 1.2 Hz, 1H), 6.97 (dd, *J* = 5.1, 3.5 Hz, 1H), 5.35 (dd, *J* = 12.8, 0.6 Hz, 1H), 5.17 (dd, *J* = 12.8, 0.6 Hz, 1H), 4.25 (dd, *J* = 11.4, 2.6 Hz, 1H), 1.87 – 1.77 (m, 4H), 1.65 – 1.50 (m, 8H), 1.49 – 1.31 (m, 10H), 1.31 – 1.25 (m, 1H), 1.18 (s, 3H), 1.10 (s, 3H), 1.05 (s, 3H), 0.99 (s, 3H). **^{13}C NMR** (126 MHz, CDCl$_3$) δ 173.7, 137.2, 128.8, 126.8, 126.7, 81.5, 60.5, 60.1, 59.1, 46.5, 42.4, 40.2, 40.1, 36.8, 33.8, 32.9, 31.3, 28.5, 20.3, 20.0, 17.1. **HRMS** (**ESI**): [M+Na]$^+$ Calcd. for $C_{27}H_{41}NO_3SNa$ 482.2699; Found: 482.2696. **FTIR** (neat): ν(cm^{-1}) 2900, 2846, 1742, 1451, 1375, 1362, 1348, 1258, 1242, 1209, 1175, 1150, 1106, 1075, 1046, 1016, 1001, 973, 957, 942, 910, 876, 853, 830, 796, 780, 730, 700, 647, 617, 562, 524, 505.

3-(Adamantan-1-yl)-1-phenyl-2-((2,2,6,6-tetramethylpiperidin-1-yl)oxy)propan-1-one (**4g**)

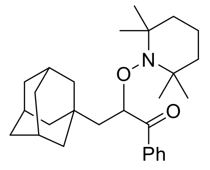

The reaction was performed according to the **GP 2** with 1-iodoadamantane (52.5 mg, 0.20 mmol, 1 equiv.) and 1-phenylprop-2-en-1-one (132 mg, 1.0 mmol, 5 equiv.). Flash column chromatography (pentane/Et$_2$O, 100/0 to 98/2) afforded 3-(adamantan-1-yl)-1-phenyl-2-((2,2,6,6-tetramethylpiperidin-1-yl)oxy)propan-1-one **4g** (50 mg, 59%) as a colourless oil. **^{1}H NMR** (300 MHz, CDCl$_3$) δ 8.19 – 8.10 (m, 2H), 7.60 – 7.40 (m, 3H), 4.90 (dd, *J* = 11.6, 2.3 Hz, 1H), 2.04 (dd, *J* = 13.9, 11.6 Hz, 1H), 1.90 – 1.76 (m, 4H), 1.64 – 1.55 (m, 3H), 1.54 – 1.43 (m, 6H), 1.43 – 1.23 (m, 12H), 1.16 (s, 3H), 0.99 (s, 3H), 0.75 (s, 3H). **^{13}C NMR** (126 MHz, CDCl$_3$) δ 201.6, 136.3, 132.2, 129.3, 127.9, 83.6, 60.0, 58.8, 47.0, 42.4, 39.9, 36.3, 33.9, 33.4, 31.4, 28.1, 19.9, 19.8, 16.6. **HRMS** (**ESI**): [M+Na]$^+$ Calcd. for $C_{28}H_{41}NO_2Na$ 446.3029; Found: 446.3022. **FTIR** (neat): ν(cm^{-1}) 2972, 2899, 2846, 1682, 1597, 1579,

1448, 1376, 1362, 1348, 1258, 1223, 1180, 1158, 1132, 1105, 1044, 1011, 974, 956, 909, 876, 864, 789, 775, 730, 698, 647, 621, 594, 529.

3-(Adamantan-1-yl)-2-((2,2,6,6-tetramethylpiperidin-1-yl)oxy)propanenitrile (**4h**)

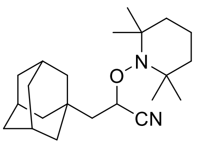

The reaction was performed according to the **GP 2** with 1-iodoadamantane (52.5 mg, 0.20 mmol, 1 equiv.) and acrylonitrile (65 µL, 1.0 mmol, 5 equiv.). Flash column chromatography (pentane/Et_2O, 100/0 to 98/2) afforded 3-(adamantan-1-yl)-2-((2,2,6,6-tetramethylpiperidin-1-yl)oxy)propanenitrile **4h** (57.2 mg, 83%) as a colourless liquid. **^{1}H NMR** (300 MHz, $CDCl_3$) δ 4.74 (t, *J* = 6.4 Hz, 1H), 1.98 (p, *J* = 3.2 Hz, 3H), 1.78 – 1.67 (m, 5H), 1.65 (q, *J* = 2.2 Hz, 3H), 1.63 – 1.39 (m, 12H), 1.31 (s, 3H), 1.21 (s, 3H), 1.12 (s, 3H), 1.06 (s, 3H). **^{13}C NMR** (126 MHz, $CDCl_3$) δ 120.7, 70.5, 60.7, 59.7, 47.5, 42.8, 42.6, 42.5, 39.8, 39.7, 36.9, 36.7, 36.6, 33.9, 33.7, 32.0, 28.6, 28.5, 28.3, 20.7, 20.6, 17.0. **HRMS** (**ESI**): $[M+Na]^+$ Calcd. for $C_{22}H_{36}N_2ONa$ 367.2719; Found: 367.2716. **FTIR** (neat): ν(cm^{-1}) 2973, 2899, 2847, 2676, 1737, 1451, 1377, 1362, 1349, 1316, 1259, 1243, 1209, 1181, 1132, 1106, 1063, 1019, 974, 957, 929, 907, 876, 797, 714, 597, 562, 517.

3-(Adamantan-1-yl)-N,N-dimethyl-2-((2,2,6,6-tetramethylpiperidin-1-yl)oxy)propanamide (**4i**)

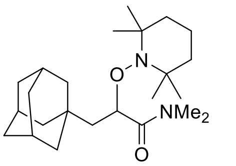

The reaction was performed according to the **GP 2** with 1-iodoadamantane (52.5 mg, 0.20 mmol, 1 equiv.) and N,N-dimethylacrylamide (102 µL, 1.0 mmol, 5 equiv.). Flash column chromatography (pentane/EtOAc, 100/0 to 85/15) afforded 3-(adamantan-1-yl)-N,N-dimethyl-2-((2,2,6,6-tetramethylpiperidin-1-yl)oxy)propanamide **4i** (45.2 mg, 58%) as a white solid. **MP**: 134-136 °C. **^{1}H NMR** (500 MHz, $CDCl_3$) δ 4.58 (dd, *J* = 11.6, 2.1 Hz, 1H), 3.24 (s, 3H), 2.88 (s, 3H), 2.04 – 1.96 (m, 1H), 1.91 – 1.86 (m, 3H), 1.68 – 1.62 (m, 3H), 1.61 – 1.55 (m, 3H), 1.54 – 1.45 (m, 5H), 1.45 – 1.30 (m, 7H), 1.29 – 1.23 (m, 1H), 1.20 (s, 3H), 1.09 (s, 3H), 1.02 (s, 3H), 0.93 (s, 3H). **^{13}C NMR** (126 MHz, $CDCl_3$) δ 173.5, 60.5, 59.1, 46.1, 42.8, 40.5, 40.2, 37.7, 37.0, 36.0, 33.8, 32.5, 31.5, 28.6, 20.4, 20.0, 17.1. **HRMS** (**ESI**): $[M+H]^+$ Calcd. for $C_{24}H_{42}N_2O_2H$ 391.3319; Found: 391.3308. **FTIR** (neat): ν(cm^{-1}) 2899, 2845, 2234, 1645, 1451, 1399, 1375, 1362, 1347, 1335, 1259, 1209, 1182, 1133, 1104, 1078, 1047, 1013, 982, 957, 921, 876, 818, 798, 728, 705, 644, 613, 559, 520, 507.

Phenyl 3-(adamantan-1-yl)-2-((2,2,6,6-tetramethylpiperidin-1-yl)oxy)propanoate (**4j**)

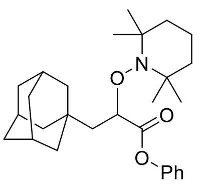

The reaction was performed according to the **GP 2** with 1-iodoadamantane (52.5 mg, 0.20 mmol, 1 equiv.) and phenyl acrylate (137 µL, 1.0 mmol, 5 equiv.). Flash column chromatography (pentane/Et_2O, 100/0 to 98/2) afforded phenyl 3-(adamantan-1-yl)-2-((2,2,6,6-tetramethylpiperidin-1-yl)oxy)propanoate **4j** (63.3 mg, 72%) as a white solid. **MP**: 138-139 °C. **^{1}H NMR** (500 MHz, $CDCl_3$) δ 7.42 – 7.34 (m, 2H), 7.25 – 7.20 (m, 1H), 7.19 – 7.13 (m, 2H), 4.50 (dd, *J* = 11.2, 2.7 Hz, 1H), 2.03 – 1.93 (m, 4H), 1.77 – 1.67 (m, 4H), 1.67 – 1.61 (m, 6H), 1.61 – 1.41 (m, 8H), 1.36 – 1.30 (m, 1H), 1.27 (s, 3H), 1.18 (d, *J* = 12.6 Hz, 6H), 1.12 (s, 3H). **^{13}C NMR** (126 MHz, $CDCl_3$) δ 172.2, 150.7, 129.3, 125.6, 121.3, 81.8, 60.6, 59.3, 47.0, 42.7, 40.3, 40.2, 36.9, 34.0, 33.7, 31.6, 28.6, 20.3, 20.1, 17.1. **HRMS** (**ESI**): $[M+Na]^+$ Calcd. for $C_{28}H_{41}NO_3Na$ 462.2978; Found: 462.2977. **FTIR** (neat): ν(cm^{-1}) 2973, 2899, 2846, 1765, 1592, 1492, 1453, 1376, 1362, 1297, 1258, 1234, 1193, 1162, 1130, 1104, 1070, 1047, 1018, 985, 955, 906, 877, 856, 798, 732, 713, 688, 648, 632, 558.

2-Methylbut-3-en-2-yl 3-(adamantan-1-yl)-2-((2,2,6,6-tetramethylpiperidin-1-yl)oxy)propanoate (**4k**)

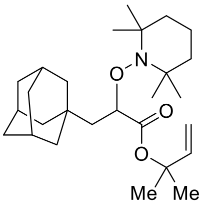

The reaction was performed according to the **GP 2** with 1-iodoadamantane (52.5 mg, 0.20 mmol, 1 equiv.) and 2-methylbut-3-en-2-yl acrylate (140 mg, 1.0 mmol, 5 equiv.). Flash column chromatography (pentane/Et_2O, 100/0 to 98/2) afforded 2-methylbut-3-en-2-yl 3-(adamantan-1-yl)-2-((2,2,6,6-tetramethylpiperidin-1-yl)oxy)propanoate **4k** (50 mg, 58%) as a white solid. **MP**: 61-62 °C. **^{1}H NMR** (500 MHz, $CDCl_3$) δ 6.16 (dd, *J* = 17.5, 10.9 Hz, 1H), 5.22 (dd, *J* = 17.5, 0.9 Hz, 1H), 5.06 (dd, *J* = 11.0, 1.1 Hz, 1H), 4.22 (dd, *J* = 10.1, 3.1 Hz, 1H), 1.98 – 1.86 (m, 3H), 1.77 – 1.57 (m, 9H), 1.55 (s, 3H), 1.54 (s, 3H), 1.54 – 1.38 (m, 10H), 1.38 – 1.16 (m, 4H), 1.16 – 0.99 (m, 9H). **^{13}C NMR** (126 MHz, $CDCl_3$) δ 172.8, 142.3, 112.8, 82.0, 81.3, 60.3, 59.3, 47.1, 42.8, 40.3, 36.9, 34.3, 34.1, 31.5, 28.6, 26.2, 26.1, 20.3, 17.1. **HRMS** (**ESI**): $[M+H]^+$ Calcd. for $C_{27}H_{45}NO_3H$ 432.3472; Found: 432.3463. **FTIR** (neat): ν(cm^{-1}) 2975, 2901, 2847, 1741, 1452, 1415, 1377, 1362, 1259, 1210, 1193, 1156, 1125, 1048, 1015, 985, 957, 923, 838, 799, 738, 703.

3-(Adamantan-1-yl)-2-((2,2,6,6-tetramethylpiperidin-1-yl)oxy)butanenitrile (4l)

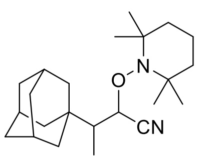

The reaction was performed according to the **GP 2** with 1-iodoadamantane (52.5 mg, 0.20 mmol, 1 equiv.) and but-2-enenitrile (80 µL, 1.0 mmol, 5 equiv.). Flash column chromatography (pentane/Et_2O, 100/0 to 98/2) afforded 3-(Adamantan-1-yl)-2-((2,2,6,6-tetramethylpiperidin-1-yl)oxy)butanenitrile **4l** (28 mg, 39%, dr 2.7:1) as a colourless oil. **^{1}H NMR** (300 MHz, $CDCl_3$) δ 5.12 – 4.62 (m, 1H), 1.99 (s, 3H), 1.79 – 1.41 (m, 18H), 1.37 – 1.24 (m, 5H), 1.22 – 1.06 (m, 11H). **^{13}C NMR** (76 MHz, $CDCl_3$) δ 121.7, 119.9, 77.4, 77.0, 76.6, 74.9, 74.5, 60.9, 60.7, 60.4, 59.8, 49.0, 46.7, 40.5, 40.4, 40.1, 40.03, 39.95, 37.0, 36.9, 35.1, 34.4, 34.2, 34.0, 33.4, 28.7, 28.5, 21.0, 20.9, 20.5, 20.4, 17.0, 16.9, 9.7, 9.5. **HRMS** (**ESI**): $[M+Na]^+$ Calcd. for $C_{23}H_{38}N_2ONa$ 381.2876; Found: 381.2878. **FTIR** (neat): ν(cm^{-1}) 2974, 2905, 2848, 1452, 1379, 1362, 1259, 1183, 1132, 1008, 986, 957, 720, 568.

Methyl 4,4-dimethyl-2-((2,2,6,6-tetramethylpiperidin-1-yl)oxy)pentanoate (4m)

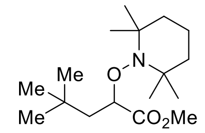

The reaction was performed according to the **GP 2** with 2-iodo-2-methylpropane **1b** (24 µL, 0.20 mmol, 1 equiv.) and methyl acrylate (180 µL, 2.0 mmol, 10 equiv.). Flash column chromatography (pentane/Et_2O, 100/0 to 99/1) afforded methyl 4,4-dimethyl-2-((2,2,6,6-tetramethylpiperidin-1-yl)oxy)pentanoate **4m** (33 mg, 55%) as a colourless oil. **^{1}H NMR** (300 MHz, $CDCl_3$) δ 4.20 (dd, *J* = 11.5, 2.8 Hz, 1H), 3.67 (s, 3H), 1.92 (dd, *J* = 13.6, 11.5 Hz, 1H), 1.73 (dd, *J* = 13.6, 2.9 Hz, 1H), 1.60 – 1.37 (m, 5H), 1.36 – 1.26 (m, 1H), 1.18 (s, 3H), 1.10 (s, 3H), 1.05 (s, 3H), 0.98 (s, 3H), 0.88 (s, 9H). **^{13}C NMR** (151 MHz, $CDCl_3$) δ 174.5, 82.9, 60.6, 59.1, 51.2, 45.5, 40.3, 40.1, 33.7, 32.8, 29.7, 29.3, 20.3, 20.0, 17.1. **HRMS** (**ESI**): $[M+Na]^+$ Calcd. for $C_{17}H_{33}NO_3Na$ 322.2352; Found: 322.2353. **FTIR** (neat): ν(cm^{-1}) 2933, 1746, 1469, 1364, 1259, 1196, 1159, 1134, 1083, 1049, 1013, 990, 957, 922, 844, 796, 715, 680.

Methyl 3-(1-methylcyclohexyl)-2-((2,2,6,6-tetramethylpiperidin-1-yl)oxy)propanoate (4n)

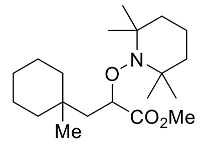

The reaction was performed according to the **GP 2** with 1-iodo-1-methylcyclohexane **1c** (45 mg, 0.20 mmol, 1 equiv.) and and methyl acrylate (180 µL, 2.0 mmol, 10 equiv.). Flash column chromatography (pentane/Et_2O, 100/0 to 99/1) afforded methyl 3-(1-methylcyclohexyl)-2-((2,2,6,6-tetramethylpiperidin-1-yl)oxy)propanoate **4n** (43.4 mg, 64%) as a colourless oil. **^{1}H NMR** (300 MHz, $CDCl_3$) δ 4.23 (dd, *J* = 11.5, 2.9 Hz, 1H), 3.66 (s, 3H), 1.98 (dd, *J* = 13.7, 11.5 Hz, 1H), 1.70 (dd, *J* = 13.7, 2.9 Hz, 1H), 1.56 – 1.33 (m, 10H), 1.32 – 1.22 (m, 4H), 1.22 – 1.13 (m, 5H), 1.08 (d, *J* = 16.2 Hz, 6H), 0.97 (s, 3H), 0.85 (s, 3H). **^{13}C NMR** (151 MHz, $CDCl_3$) δ 174.6, 82.2, 60.6, 59.1, 51.2, 44.3, 40.3, 40.1, 38.7, 37.7, 33.9, 32.8, 31.8, 26.2, 24.4, 21.9, 21.8, 20.3, 20.1, 17.1. **HRMS** (**ESI**): $[M+Na]^+$ Calcd. for $C_{20}H_{37}NO_3Na$ 362.2665; Found: 362.2661. **FTIR** (neat): ν(cm^{-1}) 2925, 2850, 1743, 1454, 1376, 1362, 1259, 1193, 1167, 1133, 1018, 988, 957, 922, 834, 794, 708, 679.

Methyl 3-(1-phenethylcyclobutyl)-2-((2,2,6,6-tetramethylpiperidin-1-yl)oxy)propanoate (4o)

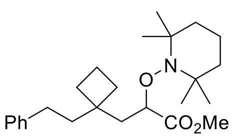

The reaction was performed according to the **GP 2** with (2-(1-iodocyclobutyl)ethyl)benzene **1d** (57.3 mg, 0.20 mmol, 1 equiv.) and methyl acrylate (180 µL, 2.0 mmol, 10 equiv.). Flash column chromatography (pentane/Et_2O, 100/0 to 98/2) afforded methyl 3-(1-phenethylcyclobutyl)-2-((2,2,6,6-tetramethylpiperidin-1-yl)oxy)propanoate **4o** (45.7 mg, 57%) as a white solid. **MP**: 85-86 °C. **^{1}H NMR** (300 MHz, $CDCl_3$) δ 7.33 – 7.25 (m, 2H), 7.24 – 7.15 (m, 3H), 4.25 (dd, *J* = 8.8, 6.8 Hz, 1H), 3.65 (s, 3H), 2.64 – 2.45 (m, 2H), 2.18 – 2.04 (m, 2H), 1.94 – 1.69 (m, 7H), 1.67 – 1.40 (m, 6H), 1.34 – 1.21 (m, 4H), 1.12 (d, *J* = 12.0 Hz, 6H), 1.02 (s, 3H). **^{13}C NMR** (126 MHz, $CDCl_3$) δ 174.1, 142.9, 128.32, 128.29, 125.6, 82.6, 60.5, 59.2, 51.3, 40.6, 40.4, 40.1, 39.6, 39.5, 34.0, 33.0, 32.0, 30.3, 20.3, 20.1, 17.1, 15.6. **HRMS** (**ESI**): $[M+Na]^+$ Calcd. for $C_{25}H_{39}NO_3Na$ 424.2822; Found: 424.2822. **FTIR** (neat): ν(cm^{-1}) 2973, 2929, 2869, 1741, 1604, 1496, 1454, 1375, 1362, 1298, 1257, 1196, 1160, 1133, 1058, 1019, 991, 974, 957, 915, 877, 842, 795, 750, 731, 698, 680, 596, 569, 507.

Methyl 2-((2,2,6,6-tetramethylpiperidin-1-yl)oxy)-3-(1-tosyloctahydro-3aH-indol-3a-yl)propanoate (4p)

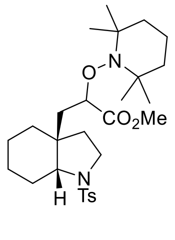

The reaction was performed according to the **GP 2** with 3a-iodo-1-tosyloctahydro-1H-indole **1e** (81 mg, 0.20 mmol, 1 equiv.) and methyl acrylate (180 µL, 2.0 mmol, 10 equiv.). Flash column chromatography (pentane/EtOAc, 100/0 to 94/6) afforded methyl 2-((2,2,6,6-tetramethylpiperidin-1-yl)oxy)-3-(1-tosyloctahydro-3aH-indol-3a-yl)propanoate **4p** (34.3 mg, 33%, dr 1:1) as a colourless liquid. **^{1}H NMR** (300 MHz, $CDCl_3$, isomers) δ 7.88 – 7.54 (m, 2H), 7.31 (d, *J* = 7.9 Hz, 2H), 4.24 – 3.94 (m, 1H), 3.61 (s, 3H), 3.53 – 3.38 (m, 1H), 3.37 – 3.18 (m, 1H), 3.13 – 2.75 (m, 1H), 2.42 (s, 3H), 2.26 – 1.61 (m, 4H), 1.56 – 1.22 (m, 14H), 1.11 (d, *J* =

26.3 Hz, 3H), 1.04 – 0.94 (m, 6H), 0.91 (s, 3H). **^{13}C NMR** (126 MHz, $CDCl_3$, isomers) δ 173.9, 173.6, 143.3, 143.1, 135.2, 134.2, 129.63, 129.60, 127.4, 127.3, 81.8, 65.52, 65.49, 60.6, 59.2, 51.4, 46.8, 45.8, 42.4, 41.8, 40.2, 40.0, 39.2, 38.2, 33.8, 32.9, 32.8, 32.1, 30.2, 29.7, 29.1, 28.5, 27.1, 21.5, 21.4, 21.3, 21.1, 20.8, 20.2, 20.1, 20.0, 17.0. **HRMS** (**ESI**): $[M+Na]^+$ Calcd. for $C_{28}H_{44}N_2O_5SNa$ 543.2863; Found: 543.2857. **FTIR** (neat): ν(cm^{-1})

6-Methoxy-3,3-dimethyl-6-oxo-5-((2,2,6,6-tetramethylpiperidin-1-yl)oxy)hexyl benzoate (**4q**)

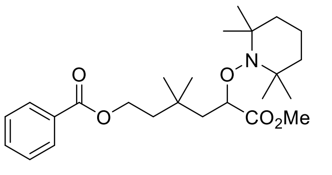

The reaction was performed according to the **GP 2** with 3-iodo-3-methylbutyl benzoate **1f** (63.6 mg, 0.20 mmol, 1 equiv.) and methyl acrylate (180 µL, 2.0 mmol, 10 equiv.). Flash column chromatography (pentane/Et_2O, 100/0 to 96/4) afforded 6-methoxy-3,3-dimethyl-6-oxo-5-((2,2,6,6-tetramethylpiperidin-1-yl)oxy)hexyl benzoate **4q** (27 mg, 31%) as a colourless oil. **^{1}H NMR** (300 MHz, $CDCl_3$) δ 8.09 – 7.98 (m, 2H), 7.60 – 7.37 (m, 3H), 4.37 (t, *J* = 7.2 Hz, 2H), 4.28 (dd, *J* = 11.2, 3.1 Hz, 1H), 3.69 (s, 3H), 2.03 (dd, *J* = 13.8, 11.2 Hz, 1H), 1.84 (dd, *J* = 13.8, 3.1 Hz, 1H), 1.76 – 1.65 (m, 2H), 1.59 – 1.51 (m, 1H), 1.50 – 1.38 (m, 4H), 1.34 – 1.26 (m, 1H), 1.20 (s, 3H), 1.08 (d, *J* = 13.8 Hz, 6H), 1.03 – 0.96 (m, 6H), 0.94 (s, 3H). **^{13}C NMR** (126 MHz, $CDCl_3$) δ 174.2, 166.5, 132.7, 130.3, 129.4, 128.2, 82.3, 61.9, 60.5, 59.1, 51.3, 44.0, 40.9, 40.2, 40.0, 33.8, 32.7, 31.3, 27.0, 20.2, 20.0, 17.0. **HRMS** (**ESI**): $[M+Na]^+$ Calcd. for $C_{25}H_{39}NO_5Na$ 456.2720; Found: 456.2712. **FTIR** (neat): ν(cm^{-1}) 2932, 1743, 1720, 1603, 1452, 1375, 1315, 1273, 1198, 1173, 1133, 1114, 1069, 1026, 990, 975, 957, 925, 843, 795, 712, 688, 598, 572, 505.

Methyl 3-cyclohexyl-2-((2,2,6,6-tetramethylpiperidin-1-yl)oxy)propanoate (**4r**)

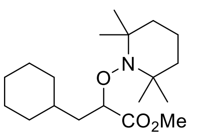

The reaction was performed according to the **GP 2** with iodocyclohexane **1g** (26 µL, 0.20 mmol, 1 equiv.) and methyl acrylate (180 µL, 2.0 mmol, 10 equiv.). Flash column chromatography (pentane/Et_2O, 100/0 to 98/2) afforded methyl 3-cyclohexyl-2-((2,2,6,6-tetramethylpiperidin-1-yl)oxy)propanoate **4r** (27.4 mg, 42%) as a colourless oil. **^{1}H NMR** (500 MHz, $CDCl_3$) δ 4.28 (dd, *J* = 10.8, 4.8 Hz, 1H), 3.69 (s, 3H), 1.82 – 1.59 (m, 7H), 1.57 – 1.38 (m, 5H), 1.32 – 1.26 (m, 1H), 1.22 – 1.05 (m, 13H), 1.03 – 0.81 (m, 5H). **^{13}C NMR** (126 MHz, $CDCl_3$) δ 174.2, 84.2, 60.4, 59.2, 51.2, 40.3, 40.2, 39.6, 34.2, 33.9, 33.5, 32.8, 32.4, 26.4, 26.2, 26.0, 20.2, 20.0, 17.1. **HRMS** (**ESI**): $[M+Na]^+$ Calcd. for $C_{19}H_{35}NO_3Na$ 348.2509; Found: 348.2507. **FTIR** (neat): ν(cm^{-1}) 2924, 2851, 1742, 1449, 1375, 1362, 1258, 1193, 1167, 1133, 1036, 992, 916, 879, 830, 795, 720.

Methyl 4-(adamantan-1-yl)-2-((2,2,6,6-tetramethylpiperidin-1-yl)oxy)pentanoate (**4s**)

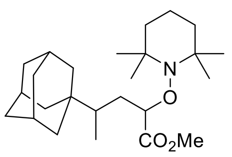

The reaction was performed according to the **GP 2** with (3r,5r,7r)-1-(1-iodoethyl)adamantane **1h** (58 mg, 0.20 mmol, 1 equiv.) and methyl acrylate (180 µL, 2.0 mmol, 10 equiv.). Flash column chromatography (pentane/Et_2O, 100/0 to 98/2) afforded methyl 4-(adamantan-1-yl)-2-((2,2,6,6-tetramethylpiperidin-1-yl)oxy)pentanoate **4s** (30.7 mg, 38%, dr 1.1:1) as a colourless oil. **^{1}H NMR** (300 MHz, $CDCl_3$, isomers) δ 4.29 – 4.18 (m, 1H), 3.68 (s, 3H), 2.15 – 1.99 (m, 1H), 1.98 – 1.90 (m, 3H), 1.72 – 1.34 (m, 18H), 1.30 – 1.06 (m, 11H), 1.00 (s, 3H), 0.80 (dd, *J* = 13.0, 6.8 Hz, 3H). **^{13}C NMR** (151 MHz, $CDCl_3$, isomers) δ 174.1, 173.9, 86.0, 85.3, 60.6, 60.4, 59.3, 59.2, 51.20, 51.18, 40.4, 40.3, 40.1, 39.5, 39.2, 39.1, 38.8, 37.31, 37.29, 35.0, 34.5, 33.8, 33.5, 33.0, 32.9, 32.8, 28.69, 28.68, 20.3, 20.2, 20.0, 17.11, 17.10, 14.0, 12.9. **HRMS** (**ESI**): $[M+Na]^+$ Calcd. for $C_{25}H_{43}NO_3Na$ 428.3135; Found: 428.3134. **FTIR** (neat): ν(cm^{-1}) 2904, 2848, 1743, 1450, 1375, 1362, 1260, 1194, 1170, 1133, 1091, 1039, 1011, 922, 792, 720.

***tert*-Butyl 4-(3-methoxy-3-oxo-2-((2,2,6,6-tetramethylpiperidin-1-yl)oxy)propyl)piperidine-1-carboxylate** (**4t**)

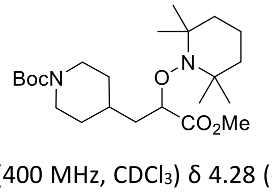

The reaction was performed according to the **GP 2** with tert-butyl 4-iodopiperidine-1-carboxylate **1i** (62.2 mg, 0.20 mmol, 1 equiv.) and methyl acrylate (180 µL, 2.0 mmol, 10 equiv.). Flash column chromatography (pentane/EtOAc, 100/0 to 94/6) afforded *tert*-butyl 4-(3-methoxy-3-oxo-2-((2,2,6,6-tetramethylpiperidin-1-yl)oxy)propyl)piperidine-1-carboxylate **4t** (30 mg, 35%) as a colourless oil. **^{1}H NMR** (400 MHz, $CDCl_3$) δ 4.28 (dd, *J* = 10.8, 4.4 Hz, 1H), 4.03 (s, 2H), 3.70 (s, 3H), 2.78 – 2.51 (m, 2H), 1.87 – 1.77 (m, 1H), 1.76 – 1.67 (m, 2H), 1.63 – 1.57 (m, 1H), 1.56 – 1.34 (m, 14H), 1.33 – 1.18 (m, 3H), 1.17 – 1.03 (m, 10H), 0.99 (s, 3H). **^{13}C NMR** (126 MHz, $CDCl_3$) δ 173.9, 154.8, 83.8, 79.2, 60.4, 59.3, 51.4, 40.3, 40.1, 38.7, 33.6, 32.9, 32.8, 32.3, 31.4, 28.4, 20.2, 20.0, 17.1. **HRMS** (**ESI**): $[M+Na]^+$ Calcd. for $C_{23}H_{42}N_2O_5Na$ 449.2985; Found: 449.2972. **FTIR** (neat): ν(cm^{-1}) 2974, 2929, 2870, 1740, 1693, 1449, 1422, 1364, 1317, 1279, 1244, 1164, 1131, 1083, 1015, 993, 973, 916, 869, 831, 788, 768, 720, 681, 616, 570, 529, 505.

Methyl 3-((8S,9S,10R,13R,14S,17R)-10,13-dimethyl-17-((R)-6-methylheptan-2-yl)-2,3,4,7,8,9,10,11,12,13,14,15,16,17-tetradecahydro-1H-cyclopenta[a]phenanthren-3-yl)-2-((2,2,6,6-tetramethylpiperidin-1-yl)oxy)propanoate (**4u**)

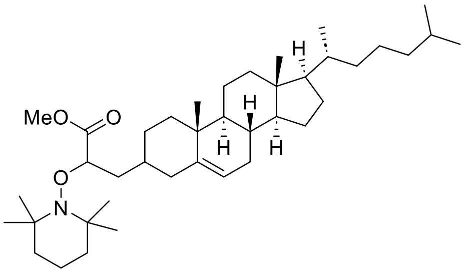

The reaction was performed according to the **GP 2** with (3R,8S,9S,10R,13R,14S,17R)-3-iodo-10,13-dimethyl-17-((R)-6-methylheptan-2-yl)-2,3,4,7,8,9,10,11,12,13,14,15,16,17-tetradecahydro-1H-cyclopenta[a]phenanthrene **1j** (99.3 mg, 0.20 mmol, 1 equiv.) and methyl acrylate (180 µL, 2.0 mmol, 10 equiv.). Flash column chromatography (pentane/Et_2O, 100/0 to 99/1) afforded methyl 3-((8S,9S,10R,13R,14S,17R)-10,13-dimethyl-17-((R)-6-methylheptan-2-yl)-2,3,4,7,8,9,10,11,12,13,14,15,16,17-tetradecahydro-1H-cyclopenta[a]phenanthren-3-yl)-2-((2,2,6,6-tetramethylpiperidin-1-yl)oxy)propanoate **4u** (41.7 mg, 34%, dr 2.2:2.1:1:1) as a white solid. **MP**: 117-119 °C. **^{1}H NMR** (300 MHz, $CDCl_3$, isomers) δ 5.19 (s, 1H), 4.30 – 4.03 (m, 1H), 3.62 (s, 3H), 2.46 – 1.63 (m, 7H), 1.62 – 1.30 (m, 14H), 1.23 (dd, *J* = 22.4, 6.5 Hz, 8H), 1.14 – 0.98 (m, 14H), 0.92 (q, *J* = 8.5 Hz, 8H), 0.87 – 0.73 (m, 10H), 0.60 (s, 3H). **^{13}C NMR** (126 MHz, $CDCl_3$, isomers) δ 174.2, 174.11, 174.07, 174.0, 142.7, 142.5, 139.9, 139.7, 122.3, 121.7, 119.9, 119.7, 84.9, 84.8, 84.1, 84.0, 60.4, 59.2, 56.8, 56.2, 51.3, 51.21, 51.20, 50.63, 50.59, 50.4, 42.29, 40.31, 40.22, 40.15, 39.9, 39.8, 39.5, 39.4, 39.3, 38.8, 38.2, 37.3, 37.2, 37.04, 37.01, 36.2, 35.8, 35.6, 35.2, 34.3, 33.9, 33.6, 33.4, 33.2, 33.1, 32.8, 31.9, 31.8, 30.5, 30.4, 29.9, 29.7, 29.4, 28.3, 28.2, 28.1, 28.00, 24.9, 24.3, 23.8, 22.8, 22.7, 22.6, 20.89, 20.85, 20.8, 20.7, 20.3, 20.1, 20.0, 19.5, 19.4, 18.7, 17.12, 17.09, 14.1, 11.8. **HRMS** (**ESI**): $[M+Na]^+$ Calcd. for $C_{40}H_{69}NO_3Na$ 634.5169; Found: 634.5170. **FTIR** (neat): ν(cm^{-1}) 2928, 2868, 1741, 1464, 1375, 1259, 1203, 1165, 1134, 1029, 991, 958, 916, 877, 800, 733, 648, 608, 588, 539, 514.

Methyl 3-((2S,5R)-2-isopropyl-5-methylcyclohexyl)-2-((2,2,6,6-tetramethylpiperidin-1-yl)oxy)propanoate (**4v**)

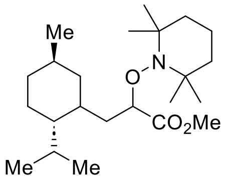

The reaction was performed according to the **GP 2** with (1S,2S,4R)-2-iodo-1-isopropyl-4-methylcyclohexane **1k** (53.2 mg, 0.20 mmol, 1 equiv.) and methyl acrylate (180 µL, 2.0 mmol, 10 equiv.). Flash column chromatography (pentane/Et_2O, 100/0 to 98/2) afforded methyl 3-((2S,5R)-2-isopropyl-5-methylcyclohexyl)-2-((2,2,6,6-tetramethylpiperidin-1-yl)oxy)propanoate **4v** (39 mg, 51%, dr 3.4:2.9:1.2:1) as a colourless liquid. **^{1}H NMR** (300 MHz, $CDCl_3$, isomers) δ 4.40 – 4.20 (m, 1H), 3.68 (s, 3H), 2.17 – 1.82 (m, 2H), 1.79 – 1.34 (m, 10H), 1.34 – 1.14 (m, 6H), 1.13 – 1.04 (m, 6H), 0.99 (s, 3H), 0.94 – 0.70 (m, 11H), 0.63 (d, *J* = 6.8 Hz, 1H). **^{13}C NMR** (126 MHz, $CDCl_3$, isomers) δ 174.00, 173.95, 173.83, 173.80, 85.2, 84.84, 84.80, 83.9, 60.5, 60.4, 59.2, 59.1, 51.14, 51.11, 51.07, 51.05, 48.7, 48.1, 47.5, 47.2, 42.7, 41.0, 40.3, 40.1, 39.5, 37.8, 36.03, 35.96, 35.8, 35.7, 35.4, 35.2, 35.12, 35.07, 33.6, 33.4, 33.1, 32.9, 32.7, 32.6, 32.4, 31.4, 30.9, 29.2, 29.0, 28.9, 27.3, 26.5, 26.24, 26.22, 26.1, 25.10, 25.08, 24.3, 24.1, 22.7, 22.62, 22.61, 22.5, 21.63, 21.62, 21.60, 21.5, 20.8, 20.24, 20.21, 20.1, 20.0, 19.9, 17.08, 17.06, 15.1, 14.8. **HRMS** (**ESI**): $[M+Na]^+$ Calcd. for $C_{23}H_{43}NO_3Na$ 404.3135; Found: 404.3133. **FTIR** (neat): ν(cm^{-1}) 2927, 2871, 1744, 1455, 1375, 1362, 1259, 1245, 1171, 1134, 1027, 990, 957, 924, 877, 792, 719, 578, 545.

Methyl 2-((2,2,6,6-tetramethylpiperidin-1-yl)oxy)undecanoate (**4w**)

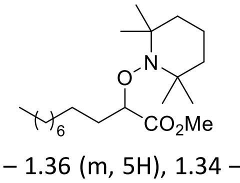

The reaction was performed according to the **GP 2** with 1-Iodooctane **1l** (36 µL, 0.20 mmol, 1 equiv.) and methyl acrylate (180 µL, 2.0 mmol, 10 equiv.). Flash column chromatography (pentane/Et_2O, 100/0 to 99/1) afforded methyl 2-((2,2,6,6-tetramethylpiperidin-1-yl)oxy)undecanoate **4w** (29 mg, 41%) as a colourless liquid. **^{1}H NMR** (300 MHz, $CDCl_3$) δ 4.22 (dd, *J* = 7.9, 6.5 Hz, 1H), 3.69 (s, 3H), 1.90 – 1.72 (m, 2H), 1.55 – 1.36 (m, 5H), 1.34 – 1.20 (m, 14H), 1.17 (s, 4H), 1.10 (d, *J* = 5.6 Hz, 6H), 1.00 (s, 3H), 0.90 – 0.82 (m, 3H). **^{13}C NMR** (151 MHz, $CDCl_3$) δ 174.1, 85.7, 60.3, 59.4, 51.3, 40.3, 40.2, 33.5, 32.8, 32.0, 31.9, 29.5, 29.4, 29.3, 24.6, 22.7, 20.2, 20.1, 17.1, 14.1. **HRMS** (**ESI**): $[M+Na]^+$ Calcd. for $C_{21}H_{41}NO_3Na$ 378.2978; Found: 378.2976. **FTIR** (neat): ν(cm^{-1}) 2925, 2854, 1743, 1465, 1375, 1362, 1260, 1243, 1195, 1166, 1133, 1023, 992, 975, 957, 917, 876, 792, 720, 675, 606, 512.

Methyl 6-methyl-2-((2,2,6,6-tetramethylpiperidin-1-yl)oxy)heptanoate (4x)

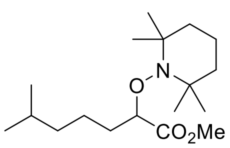

The reaction was performed according to the **GP 2** with 1-iodo-3-methylbutane **1m** (27 µL, 0.20 mmol, 1 equiv.) and methyl acrylate (180 µL, 2.0 mmol, 10 equiv.). Flash column chromatography (pentane/Et_2O, 100/0 to 99/1) afforded methyl 6-methyl-2-((2,2,6,6-tetramethylpiperidin-1-yl)oxy)heptanoate **4x** (18.8 mg, 30%) as a colourless liquid. **^{1}H NMR** (300 MHz, $CDCl_3$) δ 4.22 (t, *J* = 7.2 Hz, 1H), 3.70 (s, 3H), 1.86 – 1.71 (m, 2H), 1.61 – 1.38 (m, 6H), 1.33 – 1.24 (m, 2H), 1.23 – 1.15 (m, 6H), 1.11 (d, *J* = 5.2 Hz, 6H), 1.00 (s, 3H), 0.85 (dd, *J* = 6.6, 1.9 Hz, 6H). **^{13}C NMR** (126 MHz, $CDCl_3$) δ 174.0, 85.6, 60.3, 59.4, 51.2, 40.3, 40.2, 38.7, 33.5, 32.8, 32.1, 27.7, 22.6, 22.4, 22.3, 20.2, 20.0, 17.1. **HRMS** (**ESI**): $[M+Na]^+$ Calcd. for $C_{18}H_{35}NO_3Na$ 336.2509; Found: 336.2509. **FTIR** (neat): ν(cm^{-1}) 2929, 2870, 1744, 1465, 1375, 1362, 1260, 1170, 1134, 1035, 991, 958, 917, 792, 742.

Methyl 5-phenyl-2-((2,2,6,6-tetramethylpiperidin-1-yl)oxy)pentanoate (4y)

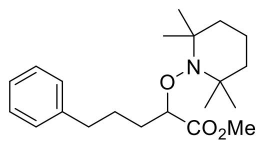

The reaction was performed according to the **GP 2** with (2-iodoethyl)benzene **1n** (29 µL, 0.20 mmol, 1 equiv.) and methyl acrylate (180 µL, 2.0 mmol, 10 equiv.). Flash column chromatography (pentane/Et_2O, 100/0 to 97/3) afforded methyl 5-phenyl-2-((2,2,6,6-tetramethylpiperidin-1-yl)oxy)pentanoate **4y** (18.7 mg, 27%) as a colourless liquid. **^{1}H NMR** (599 MHz, $CDCl_3$) δ 7.28 – 7.25 (m, 2H), 7.20 – 7.14 (m, 3H), 4.27 (dd, *J* = 7.7, 6.5 Hz, 1H), 3.69 (s, 3H), 2.75 – 2.51 (m, 2H), 1.95 – 1.81 (m, 2H), 1.63 – 1.51 (m, 3H), 1.49 – 1.39 (m, 4H), 1.33 – 1.27 (m, 1H), 1.16 – 1.07 (m, 9H), 1.01 (s, 3H). **^{13}C NMR** (151 MHz, $CDCl_3$) δ 173.8, 141.9, 128.4, 128.3, 125.8, 85.4, 60.3, 59.5, 51.3, 40.3, 40.2, 35.6, 33.6, 32.9, 31.6, 26.3, 20.2, 20.1, 17.1. **HRMS** (**ESI**): $[M+Na]^+$ Calcd. for $C_{21}H_{33}NO_3Na$ 370.2352; Found: 370.2345. **FTIR** (neat): ν(cm^{-1}) 2930, 2870, 1742, 1604, 1496, 1454, 1375, 1362, 1259, 1196, 1160, 1133, 1090, 1027, 991, 957, 916, 876, 792, 746, 698, 610, 571, 533, 506.

2,2,6,6-Tetramethyl-1-((3,3,4,4,5,5,6,6,6-nonafluoro-1-phenylhexyl)oxy)piperidine (6a)

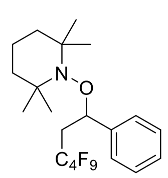

The reaction was performed according to the **GP 3** with $^{n}C_4F_9I$ (35 µL, 0.20 mmol, 1 equiv.) and styrene (115 µL, 1.0 mmol, 5 equiv.). Flash column chromatography (pentane/Et_2O, 100/0 to 97/3) afforded 2,2,6,6-tetramethyl-1-((3,3,4,4,5,5,6,6,6-nonafluoro-1-phenylhexyl)oxy)piperidine **6a** (81.4 mg, 85%) as a colourless oil. **^{1}H NMR** (500 MHz, $CDCl_3$) δ 7.37 – 7.28 (m, 5H), 5.10 (dd, *J* = 10.1, 3.3 Hz, 1H), 3.36 – 3.13 (m, 1H), 2.67 – 2.43 (m, 1H), 1.61 – 1.22 (m, 9H), 1.15 (s, 3H), 1.02 (s, 3H), 0.68 (s, 3H). **^{13}C{^{19}F} NMR** (126 MHz, $CDCl_3$) δ 141.5, 128.13, 128.07, 128.0, 117.6, 117.4, 110.3, 108.8, 79.5, 60.1, 59.9, 40.4, 36.2, 34.13, 34.05, 20.4, 20.3, 17.1. **^{19}F NMR** (470 MHz, $CDCl_3$) δ -81.12 (tt, *J* = 9.3, 3.1 Hz, 3F), -110.61 – -111.47 (m, 1F), -113.06 – -114.02 (m, 1F), -124.42 – -124.91 (m, 2F), -125.67 – -126.26 (m, 2F). **HRMS** (**ESI**): $[M+H]^+$ Calcd. for $C_{21}H_{26}F_9NOH$ 480.1943; Found: 480.1941. **FTIR** (neat): ν(cm^{-1}) 2976, 2936, 1456, 1377, 1352, 1231, 1216, 1182, 1131, 1073, 1014, 975, 957, 915, 880, 857, 807, 758, 737, 722, 696, 576, 563, 530, 499, 484, 474, 465.

2,2,6,6-Tetramethyl-1-((3,3,4,4,5,5,6,6,6-nonafluoro-1-(4-methoxyphenyl)hexyl)oxy)piperidine (6b)

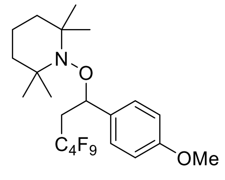

The reaction was performed according to the **GP 3** with $^{n}C_4F_9I$ (35 µL, 0.20 mmol, 1 equiv.) and 4-vinylanisole (133 µL, 1.0 mmol, 5 equiv.). Flash column chromatography (pentane/Et_2O, 100/0 to 94/6) afforded 2,2,6,6-tetramethyl-1-((3,3,4,4,5,5,6,6,6-nonafluoro-1-(4-methoxyphenyl)hexyl)oxy)piperidine **6b** (66 mg, 67%) as a colourless oil. **^{1}H NMR** (300 MHz, $CDCl_3$) δ 7.29 – 7.18 (m, 2H), 6.90 – 6.79 (m, 2H), 5.03 (dd, *J* = 10.0, 3.4 Hz, 1H), 3.78 (s, 3H), 3.32 – 3.00 (m, 1H), 2.61 – 2.26 (m, 1H), 1.61 – 1.18 (m, 9H), 1.11 (s, 3H), 0.99 (s, 3H), 0.70 – 0.63 (m, 3H). **^{13}C{^{19}F} NMR** (151 MHz, $CDCl_3$) δ 159.4, 133.7, 129.1, 117.7, 117.4, 113.5, 110.3, 108.7, 78.8, 60.1, 59.8, 55.1, 40.4, 35.9, 34.1, 20.4, 20.3, 17.1. **^{19}F NMR** (564 MHz, $CDCl_3$) δ -81.14 (tt, *J* = 9.7, 3.1 Hz, 3F), -110.64 – -111.87 (m, 1F), -113.04 – -114.20 (m, 1F), -124.42 – -125.03 (m, 2F), -125.78 – -126.26 (m, 2F). **HRMS** (**ESI**): $[M+H]^+$ Calcd. for $C_{22}H_{28}NO_2F_9H$ 510.2049; Found: 510.2045. **FTIR** (neat): ν(cm^{-1}) 2931, 1613, 1515, 1466, 1352, 1231, 1177, 1132, 1093, 1038, 975, 880, 858, 831, 720, 698, 566, 472, 459, 440, 430, 418, 406.

2,2,6,6-Tetramethyl-1-((3,3,4,4,5,5,6,6,6-nonafluoro-1-(naphthalen-2-yl)hexyl)oxy)piperidine (6c)

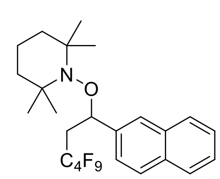

The reaction was performed according to the **GP 3** with $^{n}C_4F_9I$ (35 µL, 0.20 mmol, 1 equiv.) and 2-vinylnaphthalene (155 mg, 1.0 mmol, 5 equiv.). Flash column chromatography (pentane/Et_2O, 100/0 to 94/6) afforded 2,2,6,6-tetramethyl-1-((3,3,4,4,5,5,6,6,6-nonafluoro-1-(naphthalen-2-yl)hexyl)oxy)piperidine **6c** (89 mg, 84%) as a white solid. **MP**: 53-54 °C. **^{1}H NMR** (300 MHz, $CDCl_3$) δ 7.94 – 7.71 (m, 4H), 7.56 – 7.43 (m, 3H), 5.26 (dd, *J* = 10.2, 3.1 Hz, 1H), 3.43 – 3.15 (m, 1H), 2.80 – 2.53 (m, 1H), 1.65 – 1.24 (m, 9H), 1.18 (s, 3H), 1.02 (s, 3H), 0.65 (s, 3H). **^{13}C{^{19}F} NMR** (126 MHz, $CDCl_3$) δ 138.8, 133.2, 133.1, 128.2, 128.1, 127.7, 127.3, 126.0, 125.3, 117.6, 117.3, 110.3, 108.7, 79.8, 60.2, 59.9, 40.4, 36.2, 34.2, 20.4, 17.1. **^{19}F NMR** (470 MHz, $CDCl_3$) δ -81.10 (tt, *J* = 9.4, 3.2 Hz, 3F), -110.49 – -111.40 (m, 1F), -113.13 – -114.00 (m, 1F), -124.41 – -124.88 (m, 2F), -125.68 – -126.26 (m, 2F). **HRMS** (**ESI**): $[M+H]^+$ Calcd. for $C_{25}H_{28}NOF_9H$ 530.2099; Found: 530.2098. **FTIR** (neat): ν(cm^{-1}) 2971, 2931, 1456, 1377, 1352, 1217, 1182, 1130, 1098, 1067, 1013, 975, 955, 880, 854, 817, 775, 740, 723, 696, 660, 532, 476.

2,2,6,6-Tetramethyl-1-((2-(perfluorobutyl)-1,2,3,4-tetrahydronaphthalen-1-yl)oxy)piperidine (6d)

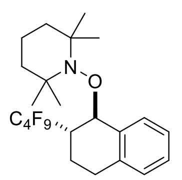

The reaction was performed according to the **GP 3** with $^{n}C_4F_9I$ (35 µL, 0.20 mmol, 1 equiv.) and 1,2-dihydronaphthalen (130 µL, 1.0 mmol, 5 equiv.). Flash column chromatography (pentane/Et_2O, 100/0 to 97/3) afforded 2,2,6,6-tetramethyl-1-((2-(perfluorobutyl)-1,2,3,4-tetrahydronaphthalen-1-yl)oxy)piperidine **6d** (50.3 mg, 50%) as a colourless oil. **^{1}H NMR** (600 MHz, $CDCl_3$) δ 7.44 (d, *J* = 7.5 Hz, 1H), 7.28 – 7.22 (m, 1H), 7.20 – 7.15 (m, 1H), 7.12 (d, *J* = 7.4 Hz, 1H), 5.17 (d, *J* = 1.8 Hz, 1H), 3.20 – 3.09 (m, 1H), 2.90 – 2.77 (m, 2H), 2.41 – 2.32 (m, 1H), 1.95 – 1.87 (m, 1H), 1.60 – 1.46 (m, 3H), 1.38 (s, 3H), 1.33 – 1.26 (m, 3H), 1.19 (s, 3H), 0.97 (s, 3H), 0.16 (s, 3H). **^{13}C{^{19}F} NMR** (151 MHz, $CDCl_3$) δ 138.5, 134.3, 131.9, 127.9, 127.2, 124.8, 118.1, 117.1, 111.0, 108.5, 75.8, 60.4, 58.8, 41.1, 40.1, 39.8, 33.9, 32.0, 26.0, 20.3, 20.1, 19.6, 16.9. **^{19}F NMR** (564 MHz, $CDCl_3$) δ -80.96 (tt, *J* = 9.5, 3.3 Hz, 3F), -110.40 – -111.67 (m, 1F), -115.93 – -117.31 (m, 1F), -121.11 – -121.85 (m, 1F), -121.87 – -122.61 (m, 1F), -125.04 – -125.72 (m, 1F), -126.30 – -126.98 (m, 1F). **HRMS** (**ESI**): $[M+H]^+$ Calcd. for $C_{23}H_{28}NOF_9H$ 506.2099; Found: 506.2100. **FTIR** (neat): ν(cm^{-1}) 2931, 1735, 1459, 1361, 1235, 1134, 913, 745, 545.

2,2,6,6-Tetramethyl-1-((5,5,6,6,7,7,8,8,8-nonafluoro-1-phenyloctan-3-yl)oxy)piperidine (6e)

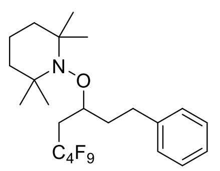

The reaction was performed according to the **GP 3** with $^{n}C_4F_9I$ (35 µL, 0.20 mmol, 1 equiv.) and 4-phenyl-1-butene (300 µL, 2.0 mmol, 10 equiv.). Flash column chromatography (pentane/Et_2O, 100/0 to 97/3) afforded 2,2,6,6-tetramethyl-1-((5,5,6,6,7,7,8,8,8-nonafluoro-1-phenyloctan-3-yl)oxy)piperidine **6e** (73 mg, 72%) as a colourless oil. **^{1}H NMR** (500 MHz, $CDCl_3$) δ 7.33 – 7.27 (m, 2H), 7.25 – 7.18 (m, 3H), 4.38 – 4.26 (m, 1H), 3.26 – 3.07 (m, 1H), 2.95 – 2.83 (m, 1H), 2.80 – 2.65 (m, 1H), 2.16 – 1.90 (m, 3H), 1.64 – 1.40 (m, 5H), 1.38 – 1.29 (m, 1H), 1.18 (s, 3H), 1.12 (d, *J* = 8.4 Hz, 6H), 1.04 (s, 3H). **^{13}C{^{19}F} NMR** (126 MHz, $CDCl_3$) δ 141.9, 128.4, 125.9, 118.3, 117.4, 110.4, 108.8, 74.2, 60.3, 59.5, 40.4, 40.1, 36.5, 34.2, 33.8, 33.1, 31.7, 20.8, 20.5, 17.2. **^{19}F NMR** (470 MHz, $CDCl_3$) δ -81.07 (tt, *J* = 9.6, 3.2 Hz, 3F), -111.06 – -112.09 (m, 1F), -113.41 – -114.35 (m, 1F), -124.61 – -124.92 (m, 2F), -125.78 – -126.00 (m, 2F). **HRMS** (**ESI**): $[M+H]^+$ Calcd. for $C_{23}H_{30}NOF_9H$ 508.2256; Found: 508.2254. **FTIR** (neat): ν(cm^{-1}) 2930, 1455, 1361, 1217, 1131, 1046, 1013, 957, 880, 794, 739, 712, 697, 508, 479, 468, 456.

2,2,6,6-Tetramethyl-1-((1,1,1,2,2,3,3,4,4-nonafluorododecan-6-yl)oxy)piperidine (6f)

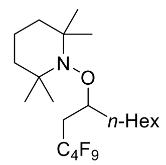

The reaction was performed according to the **GP 3** with $^{n}C_4F_9I$ (35 µL, 0.20 mmol, 1 equiv.) and 1-octane (314 µL, 2.0 mmol, 10 equiv.). Flash column chromatography (pentane/Et_2O, 100/0 to 99/1) afforded 2,2,6,6-tetramethyl-1-((1,1,1,2,2,3,3,4,4-nonafluorododecan-6-yl)oxy)piperidine **6f** (69.1 mg, 71%) as a colourless oil. **^{1}H NMR** (500 MHz, $CDCl_3$) δ 4.28 – 4.22 (m, 1H), 3.15 – 2.92 (m, 1H), 2.04 – 1.88 (m, 1H), 1.82 – 1.70 (m, 1H), 1.63 – 1.53 (m, 2H), 1.51 – 1.25 (m, 13H), 1.21 – 1.17 (m, 1H), 1.13 (d, *J* = 6.5 Hz, 5H), 1.09 (s, 3H), 1.05 (s, 3H), 0.92 – 0.87 (m, 3H). **^{13}C{^{19}F} NMR** (126 MHz, $CDCl_3$) δ 118.4, 117.5, 110.4, 108.9, 74.8, 60.2, 59.5, 40.5, 40.4, 40.2, 34.7, 34.3, 33.8, 33.3, 31.8, 29.4, 25.4, 22.6, 20.73, 20.67, 20.5, 17.3, 14.1. **^{19}F NMR** (470 MHz, $CDCl_3$) δ -80.95 – -81.23 (m, 3F), -111.30 – -112.26 (m, 1F), -113.30 – -114.17 (m, 1F), -124.47 – -124.85 (m, 2F), -125.80 – -126.12 (m, 2F). **HRMS** (**ESI**): $[M+H]^+$ Calcd. for $C_{21}H_{34}NOF_9H$ 488.2569; Found: 488.2569. **FTIR** (neat): ν(cm^{-1}) 2925, 1469, 1361, 1232, 1131, 1083, 1015, 958, 881, 739, 712, 588, 501, 483, 472.

1-((1-Cyclohexyl-3,3,4,4,5,5,6,6,6-nonafluorohexyl)oxy)-2,2,6,6-tetramethylpiperidine (6g)

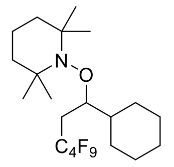

The reaction was performed according to the **GP 3** with C_4F_9I (35 µL, 0.20 mmol, 1 equiv.) and vinylcyclohexane (270 µL, 2.0 mmol, 10 equiv). Flash column chromatography (pentane) afforded 1-((1-cyclohexyl-3,3,4,4,5,5,6,6,6-nonafluorohexyl)oxy)-2,2,6,6-tetramethylpiperidine **6g** (63.1 mg, 65%) as a colourless oil. **^{1}H NMR** (500 MHz, $CDCl_3$) δ 4.33 – 4.12 (m, 1H), 3.33 – 3.09 (m, 1H), 2.20 – 2.03 (m, 1H), 1.86 – 1.75 (m, 2H), 1.74 – 1.36 (m, 10H), 1.36 – 1.20 (m, 5H), 1.19 (s, 3H), 1.16 (s, 3H), 1.09 (s, 3H), 1.00 (s, 3H). **^{13}C{^{19}F} NMR** (126 MHz, $CDCl_3$) δ 118.7, 117.5, 110.5, 108.9, 77.8, 60.9, 59.6, 42.3, 40.7, 40.3, 34.5, 33.5, 31.0, 29.1, 27.0, 26.70, 26.65, 25.3, 21.1, 20.8, 17.2. **^{19}F NMR** (470 MHz, $CDCl_3$) δ -81.11 (tt, *J* = 9.7, 3.3 Hz, 3F), -110.25 – -112.17 (m, 1F), -113.71 – -115.83 (m, 1F), -124.59 – -124.95 (m, 2F), -125.69 – -126.05 (m, 2F). **HRMS** (**ESI**): $[M+H]^+$ Calcd. for $C_{21}H_{32}NOF_9H$ 486.2412; Found: 486.2408. **FTIR** (neat): ν(cm^{-1}) 2930, 2856, 1453, 1397, 1376, 1361, 1323, 1231, 1218, 1132, 1084, 1066, 1046, 1021, 973, 922, 902, 881, 855, 798, 776, 740, 719, 647, 597, 562, 534.

1-((1-(Cyclohexyloxy)-3,3,4,4,5,5,6,6,6-nonafluorohexyl)oxy)-2,2,6,6-tetramethylpiperidine (6h)

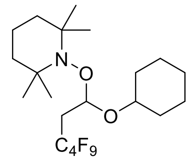

The reaction was performed according to the **GP 3** with $^nC_4F_9I$ (35 µL, 0.20 mmol, 1 equiv.) and cyclohexyl vinyl ether (280 µL, 2.0 mmol, 10 equiv.). Flash column chromatography (pentane/Et_2O, 200/1) afforded 1-((1-(cyclohexyloxy)-3,3,4,4,5,5,6,6,6-nonafluorohexyl)oxy)-2,2,6,6-tetramethylpiperidine **6h** (69.3 mg, 69%) as a colourless oil. **^{1}H NMR** (500 MHz, $CDCl_3$) δ 5.41 (dd, *J* = 5.5, 4.3 Hz, 1H), 3.92 – 3.73 (m, 1H), 2.79 – 2.55 (m, 1H), 2.38 – 2.17 (m, 1H), 2.05 – 1.95 (m, 1H), 1.91 (dd, *J* = 10.3, 4.6 Hz, 1H), 1.80 – 1.67 (m, 2H), 1.65 – 1.42 (m, 6H), 1.41 – 1.20 (m, 6H), 1.18 (s, 3H), 1.16 (s, 3H), 1.11 (s, 3H), 1.09 (s, 3H). **^{13}C{^{19}F} NMR** (126 MHz, $CDCl_3$) δ 117.43, 117.39, 110.3, 108.8, 97.9, 78.0, 60.3, 59.7, 40.33, 40.30, 36.1, 33.9, 33.7, 33.1, 32.2, 25.7, 24.3, 24.2, 20.6, 20.1, 17.2. **^{19}F NMR** (470 MHz, $CDCl_3$) δ -81.12 (tt, *J* = 9.3, 3.2 Hz, 3F), -111.81 – -112.57 (m, 1F), -112.58 – -113.33 (m, 1F), -124.56 – -124.86 (m, 2F), -125.80 – -126.09 (m, 2F). **HRMS** (**ESI**): $[M+H]^+$ Calcd. for $C_{21}H_{32}NO_2F_9H$ 502.2362; Found: 502.2359. **FTIR** (neat): ν(cm^{-1}) 2933, 2858, 1736, 1656, 1453, 1377, 1353, 1300, 1231, 1219, 1200, 1184, 1167, 1131, 1079, 1022, 956, 929, 880, 797, 769, 739, 712, 691, 642, 585, 562, 532.

1-((1-(Dimethyl(phenyl)silyl)-4,4,5,5,6,6,7,7,7-nonafluoroheptan-2-yl)oxy)-2,2,6,6-tetramethylpiperidine (6i)

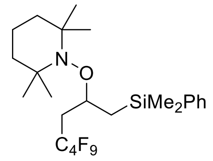

The reaction was performed according to the **GP 3** with $^nC_4F_9I$ (35 µL, 0.20 mmol, 1 equiv.) and allyldimethyl(phenyl)silane (353 mg, 2.0 mmol, 10 equiv.). Flash column chromatography (pentane) afforded 1-((1-(dimethyl(phenyl)silyl)-4,4,5,5,6,6,7,7,7-nonafluoroheptan-2-yl)oxy)-2,2,6,6-tetramethylpiperidine **6i** (77.3 mg, 70%) as a colourless oil. **^{1}H NMR** (300 MHz, $CDCl_3$) δ 7.61 – 7.48 (m, 2H), 7.42 – 7.31 (m, 3H), 4.51 – 4.33 (m, 1H), 3.40 – 2.98 (m, 1H), 2.04 – 1.77 (m, 1H), 1.68 – 1.38 (m, 6H), 1.35 – 1.27 (m, 1H), 1.19 (dd, *J* = 14.7, 5.7 Hz, 1H), 1.09 (s, 3H), 1.04 (s, 3H), 1.00 (s, 6H), 0.41 (s, 3H), 0.39 (s, 3H). **^{13}C{^{19}F} NMR** (126 MHz, $CDCl_3$) δ 139.0, 133.5, 129.0, 127.84, 127.81, 118.4, 117.5, 110.3, 108.8, 73.2, 59.9, 59.4, 40.4, 40.2, 35.8, 33.8, 33.6, 23.3, 21.0, 20.8, 17.2, -1.4, -1.5, -2.4, -2.5. **^{19}F NMR** (470 MHz, $CDCl_3$) δ -81.10 (tt, *J* = 9.7, 3.2 Hz, 3F), -111.19 – -112.53 (m, 1F), -112.74 – -114.23 (m, 1F), -124.50 – -125.27 (m, 2F), -125.63 – -126.30 (m, 2F). **HRMS** (**ESI**): $[M+H]^+$ Calcd. for $C_{24}H_{34}NOF_9SiH$ 552.2338; Found: 552.2339. **FTIR** (neat): ν(cm^{-1}) 2934, 2873, 1454, 1377, 1352, 1231, 1213, 1164, 1132, 1067, 1044, 1021, 964, 909, 879, 843, 822, 787, 724, 709, 693, 535, 506, 489, 481, 471, 462, 455, 447, 438, 430, 422, 416, 407.

1-((1-Bromo-6,6,7,7,8,8,9,9,9-nonafluorononan-4-yl)oxy)-2,2,6,6-tetramethylpiperidine (6j)

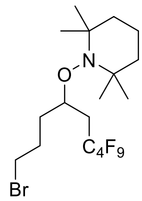

The reaction was performed according to the **GP 3** with $^nC_4F_9I$ (35 µL, 0.20 mmol, 1 equiv.), and 5-bromopent-1-ene (235 µL, 2.0 mmol, 10 equiv.). Flash column chromatography (pentane) afforded 1-((1-bromo-6,6,7,7,8,8,9,9,9-nonafluorononan-4-yl)oxy)-2,2,6,6-tetramethylpiperidine **6j** (59 mg, 56%) as a colourless oil. **^{1}H NMR** (500 MHz, $CDCl_3$) δ 4.34 – 4.22 (m, 1H), 3.46 (t, *J* = 6.8 Hz, 2H), 3.20 (dd, *J* = 14.7, 3.0 Hz, 1H), 2.18 – 2.07 (m, 1H), 2.03 – 1.89 (m, 2H), 1.87 – 1.79 (m, 2H), 1.60 – 1.40 (m, 5H), 1.37 – 1.27 (m, 1H), 1.14 (d, *J* = 7.5 Hz, 6H), 1.08 (s, 3H), 1.04 (s, 3H). **^{13}C{^{19}F} NMR** (126 MHz, $CDCl_3$) δ 118.3, 117.4, 110.3, 108.8, 74.2, 60.4, 59.5, 40.4, 40.1, 34.3, 33.8, 33.61, 33.59, 33.1, 29.0, 20.9, 20.6, 17.2. **^{19}F NMR** (470 MHz, $CDCl_3$) δ -81.10 (tt, *J* = 9.5, 3.3 Hz, 3F), -111.14 – -111.96 (m, 1F), -113.74 – -114.59 (m, 1F), -124.50 – -124.94 (m, 2F), -125.71 – -126.10 (m, 2F). **HRMS** (**ESI**): $[M+H]^+$ Calcd. for $C_{18}H_{27}NOBrF_9H$ 524.1205; Found: 524.1205. **FTIR** (neat): ν(cm^{-1}) 2932, 1456, 1441, 1378, 1361, 1322, 1218, 1183, 1131, 1045, 1014, 957, 930, 880, 782, 739, 713, 691, 652, 586, 565, 533, 510.

7,7,8,8,9,9,10,10,10-Nonafluoro-5-((2,2,6,6-tetramethylpiperidin-1-yl)oxy)decan-1-ol (6k)

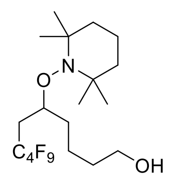

The reaction was performed according to the **GP 3** with $^{n}C_4F_9I$ (35 µL, 0.20 mmol, 1 equiv.) and 5-hexene-1-ol (240 µL, 2.0 mmol, 10 equiv.). Flash column chromatography (pentane/Et_2O, 100/0 to 60/40) afforded 7,7,8,8,9,9,10,10,10-nonafluoro-5-((2,2,6,6-tetramethylpiperidin-1-yl)oxy)decan-1-ol **6k** (58 mg, 61%) as a colourless oil. **^{1}H NMR** (500 MHz, $CDCl_3$) δ 4.37 – 4.16 (m, 1H), 3.67 (t, *J* = 6.4 Hz, 2H), 3.08 (dd, *J* = 15.5, 3.5 Hz, 1H), 2.01 – 1.91 (m, 1H), 1.85 – 1.73 (m, 1H), 1.68 – 1.53 (m, 5H), 1.51 – 1.37 (m, 6H), 1.35 – 1.28 (m, 1H), 1.13 (d, *J* = 6.6 Hz, 6H), 1.08 (s, 3H), 1.04 (s, 3H). **^{13}C{^{19}F} NMR** (126 MHz, $CDCl_3$) δ 118.3, 117.4, 110.4, 108.8, 74.6, 62.8, 60.2, 59.5, 40.3, 40.1, 34.5, 34.3, 33.8, 33.2, 32.8, 21.6, 20.8, 20.5, 17.2. **^{19}F NMR** (470 MHz, $CDCl_3$) δ -81.12 (tt, *J* = 9.4, 3.2 Hz, 3F), -111.35 – -112.14 (m, 1F), -113.46 – -114.27 (m, 1F), -124.61 – -124.89 (m, 2F), -125.92 (tt, *J* = 12.6, 4.0 Hz, 2F). **HRMS** (**ESI**): $[M+H]^+$ Calcd. for $C_{19}H_{30}NO_2F_9H$ 476.2205; Found: 476.2204. **FTIR** (neat): ν(cm^{-1}) 3317, 2933, 2872, 1736, 1456, 1377, 1361, 1322, 1299, 1218, 1183, 1131, 1060, 1046, 974, 957, 929, 880, 769, 739, 712, 690, 653, 588, 533, 514.

2,2,6,6-Tetramethyl-1-((5,5,6,6,7,7,8,8,8-nonafluoro-1-(oxiran-2-yl)octan-3-yl)oxy)piperidine (6l)

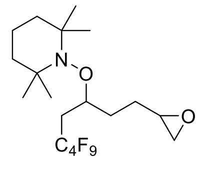

The reaction was performed according to the **GP 3** with $^{n}C_4F_9I$ (35 µL, 0.20 mmol, 1 equiv.) and 2-(but-3-en-1-yl)oxirane (230 µL, 2.0 mmol, 10 equiv.). Flash column chromatography (pentane/Et_2O, 100/0 to 97/3) afforded 2,2,6,6-tetramethyl-1-((5,5,6,6,7,7,8,8,8-nonafluoro-1-(oxiran-2-yl)octan-3-yl)oxy)piperidine **6l** (64.2 mg, 68%, d:r 1:1) as a colourless oil. **^{1}H NMR** (300 MHz, $CDCl_3$, isomers) δ 4.51 – 4.14 (m, 1H), 3.29 – 3.02 (m, 1H), 3.00 – 2.89 (m, 1H), 2.77 (t, *J* = 4.5 Hz, 1H), 2.60 – 2.45 (m, 1H), 2.06 – 1.72 (m, 4H), 1.72 – 1.37 (m, 6H), 1.37 – 1.27 (m, 1H), 1.13 (s, 6H), 1.07 (s, 3H), 1.03 (s, 3H). **^{13}C{^{19}F} NMR** (126 MHz, $CDCl_3$, isomers) δ 118.3, 117.4, 110.3, 108.8, 74.34, 74.31, 60.4, 59.5, 52.2, 52.1, 47.1, 47.0, 40.3, 40.1, 34.2, 33.8, 33.12, 33.08, 31.1, 31.0, 28.6, 28.4, 20.8, 20.5, 17.2. **^{19}F NMR** (470 MHz, $CDCl_3$, isomers) δ -81.13 (tt, *J* = 9.5, 3.2 Hz, 3F), -111.07 – -112.11 (m, 1F), -113.52 – -114.55 (m, 1F), -124.51 – -125.03 (m, 2F), -125.73 – -126.11 (m, 2F). **HRMS** (**ESI**): $[M+H]^+$ Calcd. for $C_{19}H_{28}NO_2F_9H$ 474.2049; Found: 474.2049. **FTIR** (neat): ν(cm^{-1}) 2930, 1455, 1377, 1361, 1321, 1217, 1182, 1131, 1070, 1045, 1014, 957, 927, 880, 838, 770, 739, 712, 691, 588.

2,2,6,6-Tetramethyl-1-((6,6,7,7,8,8,9,9,9-nonafluoro-4-methylnonan-4-yl)oxy)piperidine (6m)

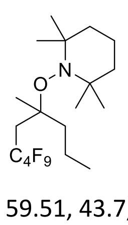

The reaction was performed according to the **GP 3** with $^{n}C_4F_9I$ (35 µL, 0.20 mmol, 1 equiv.) and 2-methyl-1-pentene (246 µL, 2.0 mmol, 10 equiv.). Flash column chromatography (pentane) afforded 2,2,6,6-tetramethyl-1-((6,6,7,7,8,8,9,9,9-nonafluoro-4-methylnonan-4-yl)oxy)piperidine **6m** (78.1 mg, 85%) as a colourless oil. **^{1}H NMR** (500 MHz, $CDCl_3$) δ 2.71 (dd, *J* = 16.2, 9.9 Hz, 1H), 2.48 (dd, *J* = 15.9, 9.4 Hz, 1H), 1.81 – 1.73 (m, 2H), 1.61 – 1.41 (m, 10H), 1.33 – 1.21 (m, 1H), 1.14 – 1.07 (m, 12H), 0.94 (t, *J* = 7.3 Hz, 3H). **^{13}C{^{19}F} NMR** (126 MHz, $CDCl_3$) δ 118.5, 117.4, 110.3, 108.8, 79.3, 59.52, 59.51, 43.7, 40.9, 37.1, 34.6, 34.5, 25.3, 20.72, 20.70, 16.9, 16.8, 14.4. **^{19}F NMR** (470 MHz, $CDCl_3$) δ -81.15 (tt, *J* = 9.8, 3.3 Hz, 3F), -111.11 – -111.87 (m, 1F), -111.94 – -112.71 (m, 1F), -124.45 – -124.85 (m, 2F), -125.61 – -125.90 (m, 2F). **HRMS** (**ESI**): $[M+H]^+$ Calcd. for $C_{19}H_{30}NOF_9H$ 460.2256; Found: 460.2256. **FTIR** (neat): ν(cm^{-1}) 2970, 2935, 2875, 1466, 1377, 1350, 1219, 1130, 1089, 1072, 1040, 976, 957, 918, 879, 778, 739, 723, 710, 688, 584, 552, 530, 502.

Methyl 4,4,5,5,6,6,7,7,7-nonafluoro-2-((2,2,6,6-tetramethylpiperidin-1-yl)oxy)heptanoate (6n)

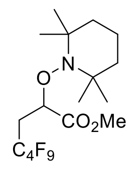

The reaction was performed according to the **GP 3** with $^{n}C_4F_9I$ (35 µL, 0.20 mmol, 1 equiv.) and methyl acrylate (180 µL, 2.0 mmol, 10 equiv.). Flash column chromatography (pentane/Et_2O, 100/0 to 97/3) afforded methyl 4,4,5,5,6,6,7,7,7-nonafluoro-2-((2,2,6,6-tetramethylpiperidin-1-yl)oxy)heptanoate **6n** (15.7 mg, 17%) as a colourless oil. **^{1}H NMR** (500 MHz, $CDCl_3$) δ 4.61 (dd, *J* = 10.3, 3.2 Hz, 1H), 3.74 (s, 3H), 2.91 – 2.71 (m, 1H), 2.69 – 2.58 (m, 1H), 1.61 – 1.42 (m, 5H), 1.35 – 1.28 (m, 1H), 1.18 (s, 3H), 1.10 (d, *J* = 15.8 Hz, 6H), 1.02 (s, 3H). **^{13}C{^{19}F} NMR** (126 MHz, $CDCl_3$) δ 171.7, 117.3, 117.0, 110.1, 108.7, 77.8, 60.7, 59.8, 52.0, 40.2, 40.1, 33.5, 33.4, 32.7, 20.2, 20.0, 17.0. **^{19}F NMR** (470 MHz, $CDCl_3$) δ -81.08 (tt, *J* = 9.7, 3.2 Hz, 3F), -112.56 – -113.09 (m, 2F), -123.92 – -125.07 (m, 2F), -125.22 – -126.52 (m, 2F). **HRMS** (**ESI**): $[M+Na]^+$ Calcd. for $C_{17}H_{24}NO_3F_9Na$ 484.1504; Found: 484.1501. **FTIR** (neat): ν(cm^{-1}) 2933, 1753, 1439, 1353, 1218, 1132, 1083, 1020, 992, 976, 957, 918, 880, 860, 798, 735, 715, 679, 616, 529, 499, 481, 471, 461, 451, 430.

2,2,6,6-Tetramethyl-1-((5,5,6,6,7,7,8,8,8-nonafluoro-1-phenyloct-1-yn-3-yl)oxy)piperidine (6o)

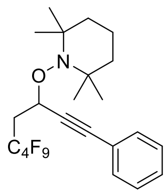

The reaction was performed according to the **GP 3** with $^nC_4F_9I$ (35 µL, 0.20 mmol, 1 equiv.) and but-3-en-1-yn-1-ylbenzene (256 mg, 2.0 mmol, 10 equiv.). Flash column chromatography (pentane/Et_2O, 100/0 to 99/1) afforded 2,2,6,6-tetramethyl-1-((5,5,6,6,7,7,8,8,8-nonafluoro-1-phenyloct-1-yn-3-yl)oxy)piperidine **6o** (82 mg, 81%) as a colourless oil. **^{1}H NMR** (300 MHz, $CDCl_3$) δ 7.47 – 7.40 (m, 2H), 7.36 – 7.29 (m, 3H), 5.12 (t, *J* = 6.3 Hz, 1H), 2.86 – 2.51 (m, 2H), 1.63 – 1.29 (m, 9H), 1.24 (s, 3H), 1.14 (d, *J* = 10.2 Hz, 6H). **^{13}C{^{19}F} NMR** (151 MHz, $CDCl_3$) δ 132.9, 130.8, 128.2, 127.8, 127.7, 122.3, 116.8, 116.4, 109.7, 108.2, 87.9, 86.7, 67.6, 60.0, 59.0, 39.6, 36.2, 34.0, 33.2, 19.9, 19.6, 16.6. **^{19}F NMR** (564 MHz, $CDCl_3$) δ -80.52 – -81.17 (m, 3F), -111.60 – -113.77 (m, 2F), -123.33 – -125.05 (m, 2F), -125.58 – -127.09 (m, 2F). **HRMS** (**ESI**): $[M+H]^+$ Calcd. for $C_{23}H_{26}NOF_9H$ 504.1943; Found: 504.1942. **FTIR** (neat): ν(cm^{-1}) 2976, 2934, 2208, 1748, 1687, 1638, 1599, 1491, 1452, 1379, 1353, 1219, 1131, 1079, 1025, 960, 926, 882, 793, 756, 742, 710, 689, 651, 592, 568, 528.

2,2,6,6-Tetramethyl-1-((2-(perfluorobutyl)cyclohexyl)oxy)piperidine (6p)

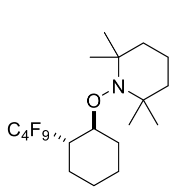

The reaction was performed according to the **GP 3**, with $^nC_4F_9I$ (35 µL, 0.20 mmol, 1 equiv.) and cyclohexene (200 µL, 2.0 mmol, 10 equiv.). Flash column chromatography (pentane) afforded 2,2,6,6-tetramethyl-1-((2-(perfluorobutyl)cyclohexyl)oxy)piperidine **6p** (24.7 mg, 27%) as a colourless oil. **^{1}H NMR** (500 MHz, $CDCl_3$) δ 4.20 – 3.97 (m, 1H), 2.58 – 2.39 (m, 1H), 2.30 – 2.17 (m, 1H), 2.01 – 1.87 (m, 1H), 1.77 – 1.61 (m, 2H), 1.60 – 1.50 (m, 3H), 1.49 – 1.38 (m, 4H), 1.37 – 1.24 (m, 3H), 1.18 – 1.05 (m, 12H). **^{13}C{^{19}F} NMR** (126 MHz, $CDCl_3$) δ 119.03, 117.61, 111.68, 108.99, 77.67, 77.25, 77.00, 76.75, 60.32, 59.35, 43.60, 40.63, 40.36, 34.35, 33.98, 29.71, 23.85, 23.14, 22.53, 20.32, 17.26. **^{19}F NMR** (470 MHz, $CDCl_3$) δ -80.86 – -81.04 (m, 3F), -106.81 (d, *J* = 281.6 Hz, 1F), -113.38 (d, *J* = 281.7 Hz, 1F), -119.56 – -122.99 (m, 2F), -124.47 – -127.35 (m, 2F). HRMS (ESI): $[M + H]^+$ Calcd for $C_{19}H_{28}NOF_9H$ 458.2099; Found: 458.2097. **FTIR** (neat): ν(cm^{-1}) 2934, 2873, 1454, 1377, 1352, 1231, 1213, 1164, 1132, 1067, 1044, 1021, 964, 909, 879, 843, 822, 787, 724, 709, 693, 535, 506, 489, 481, 471, 462, 455, 447, 438, 430, 422, 416, 407.

2,2,6,6-Tetramethyl-1-((3,3,4,4,5,5,6,6,6-nonafluoro-2-methyl-1-phenylhexyl)oxy)piperidine (6q)

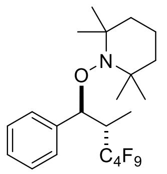

The reaction was performed according to the **GP 3**, with $^nC_4F_9I$ (35 µL, 0.20 mmol, 1 equiv.) and (Z)-prop-1-en-1-ylbenzene (260 µL, 2.0 mmol, 10 equiv.). Flash column chromatography (pentane/Et_2O, 100/0 to 97/3) afforded 2,2,6,6-tetramethyl-1-((3,3,4,4,5,5,6,6,6-nonafluoro-2-methyl-1-phenylhexyl)oxy)piperidine **6q** (40.4 mg, 41%, dr 9:1) as a colourless oil. **^{1}H NMR** (500 MHz, $CDCl_3$) δ 7.45 – 7.37 (m, 2H), 7.35 – 7.27 (m, 3H), 4.96 (d, *J* = 4.0 Hz, 1H), 3.47 – 3.34 (m, 1H), 1.71 – 0.75 (m, 21H). **^{13}C{^{19}F} NMR** (126 MHz, $CDCl_3$) δ 140.02, 138.83, 130.66, 129.08, 128.32, 127.47, 127.42, 127.28, 127.16, 126.16, 119.29, 118.63, 117.39, 111.26, 108.79, 85.49, 82.64, 77.25, 77.00, 76.75, 60.26, 59.76, 40.67, 40.40, 40.02, 38.88, 34.58, 33.35, 29.61, 20.35, 16.99, 16.95, 11.27, 7.39. **^{19}F NMR** (470 MHz, $CDCl_3$) δ -80.79 – -81.34 (m, 3F), -110.50 – -112.45 (m, 1F), -115.83 – -118.10 (m, 1F), -119.90 – -122.56 (m, 2F), -123.88 – -125.85 (m, 1F), -126.33 – -127.92 (m, 1F). HRMS (ESI): $[M + H]^+$ Calcd for $C_{22}H_{28}NOF_9H$ 494.2099; Found: 494.2100. **FTIR** (neat): ν(cm^{-1}) 2932, 1457, 1377, 1352, 1294, 1232, 1217, 1132, 1103, 1016, 975, 958, 907, 875, 826, 789, 754, 700, 585.

1-((3,3,4,4,5,5,5-Heptafluoro-1-phenylpentyl)oxy)-2,2,6,6-tetramethylpiperidine (6r)

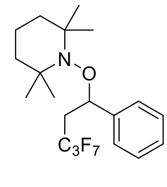

The reaction was performed according to the **GP 3** with $^nC_3F_7I$ (29 µL, 0.20 mmol, 1 equiv.) and styrene (115 µL, 1.0 mmol, 5 equiv.). Flash column chromatography (pentane/Et_2O, 100/0 to 97/3) afforded 1-((3,3,4,4,5,5,5-heptafluoro-1-phenylpentyl)oxy)-2,2,6,6-tetramethylpiperidine **6r** (66.7 mg, 78%) as a colourless oil. **^{1}H NMR** (500 MHz, $CDCl_3$) δ 7.37 – 7.28 (m, 5H), 5.10 (dd, *J* = 10.0, 3.4 Hz, 1H), 3.27 – 3.19 (m, 1H), 2.59 – 2.50 (m, 1H), 1.61 – 1.23 (m, 9H), 1.16 (s, 3H), 1.02 (s, 3H), 0.67 (s, 3H). **^{13}C{^{19}F} NMR** (151 MHz, $CDCl_3$) δ 140.8, 127.5, 127.4, 127.3, 117.2, 116.4, 108.0, 78.9, 59.4, 59.2, 39.7, 35.3, 33.5, 33.4, 19.7, 19.6, 16.4. **^{19}F NMR** (470 MHz, $CDCl_3$) δ -80.40 (t, *J* = 9.7 Hz, 3F), -111.30 – -112.22 (m, 1F), -113.89 – -114.77 (m, 1F), -127.96 (dd, *J* = 32.1, 5.9 Hz, 2F). **HRMS** (**ESI**): $[M+H]^+$ Calcd. for $C_{20}H_{26}NOF_7H$ 430.1975; Found: 430.1974. **FTIR** (neat): ν(cm^{-1}) 2935, 1457, 1354, 1222, 1173, 1117, 956, 918, 810, 759, 697, 683, 661, 608, 585, 554.

2,2,6,6-Tetramethyl-1-((3,3,4,4,5,5,6,6,7,7,8,8,8-tridecafluoro-1-phenyloctyl)oxy)piperidine (**6s**)

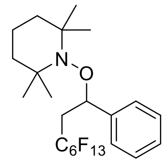

The reaction was performed according to the **GP 3** with $^{n}C_6F_{13}I$ (44 µL, 0.20 mmol, 1 equiv.) and styrene (115 µL, 1.0 mmol, 5 equiv.). For $^{n}C_6F_{13}Br$ (80 mg, 0.20 mmol, 1 equiv.) similar condition like $^{n}C_6F_{13}I$ but the reaction was stirred for 18 h. Flash column chromatography (pentane/Et_2O, 100/0 to 97/3) afforded 2,2,6,6-tetramethyl-1-((3,3,4,4,5,5,6,6,7,7,8,8,8-tridecafluoro-1-phenyloctyl)oxy)piperidine **6s** (X = I, 99.2 mg, 86%; X = Br 104 mg, 90%) as a colourless oil. **^{1}H NMR** (500 MHz, $CDCl_3$) δ 7.37 – 7.28 (m, 5H), 5.10 (dd, *J* = 10.0, 3.3 Hz, 1H), 3.33 – 3.15 (m, 1H), 2.64 – 2.42 (m, 1H), 1.60 – 1.22 (m, 9H), 1.15 (s, 3H), 1.02 (s, 3H), 0.68 (s, 3H). **^{13}C{^{19}F} NMR** (126 MHz, $CDCl_3$) δ 141.5, 128.2, 128.1, 128.0, 117.8, 117.2, 111.1, 110.8, 110.2, 108.5, 79.6, 60.1, 59.9, 40.4, 36.3, 34.2, 20.4, 17.1. **^{19}F NMR** (470 MHz, $CDCl_3$) δ -80.87 (tt, *J* = 10.0, 2.5 Hz, 3F), -110.41 – -111.22 (m, 1F), -112.83 – -113.64 (m, 1F), -121.71 – -121.98 (m, 2F), -122.74 – -123.09 (m, 2F), -123.56 – -123.86 (m, 2F), -126.03 – -126.33 (m, 2F). **HRMS** (**ESI**): $[M+H]^+$ Calcd. for $C_{23}H_{26}NOF_{13}H$ 580.1879; Found: 580.1880. **FTIR** (neat): ν(cm^{-1}) 2976, 2933, 2359, 1496, 1457, 1377, 1362, 1319, 1235, 1191, 1143, 1121, 1070, 1019, 1005, 975, 957, 916, 877, 847, 809, 760, 747, 732, 708, 695, 657, 616, 553.

1-((3,3,4,4,5,5,6,6,7,7,8,8,9,9,10,10,10-Heptadecafluoro-1-phenyldecyl)oxy)-2,2,6,6-tetramethylpiperidine (**6t**)

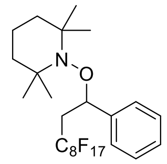

The reaction was performed according to the **GP 3** with $^{n}C_8F_{17}Br$ (99.8 mg, 0.20 mmol, 1 equiv.) and styrene (115 µL, 1.0 mmol, 5 equiv.) and the reaction mixture was stirred for 18 h. Flash column chromatography (pentane/Et_2O, 100/0 to 97/3) afforded 1-((3,3,4,4,5,5,6,6,7,7,8,8,9,9,10,10,10-heptadecafluoro-1-phenyldecyl)oxy)-2,2,6,6-tetramethylpiperidine **6t** (110 mg, 81%) as a white solid. **MP**: 49-51 °C. **^{1}H NMR** (500 MHz, $CDCl_3$) δ 7.39 – 7.28 (m, 5H), 5.17 – 5.04 (m, 1H), 3.45 – 3.06 (m, 1H), 2.75 – 2.40 (m, 1H), 1.64 – 1.24 (m, 9H), 1.17 (s, 3H), 1.03 (s, 3H), 0.69 (s, 3H). **^{13}C{^{19}F} NMR** (126 MHz, $CDCl_3$) δ 141.5, 128.1, 128.0, 127.9, 117.8, 117.1, 111.2, 111.1, 110.8, 110.72, 110.68, 110.2, 108.4, 79.5, 60.1, 59.8, 40.3, 36.2, 34.0, 20.4, 20.2, 17.1. **^{19}F NMR** (470 MHz, $CDCl_3$) δ -80.82 – -81.02 (m, 3F), -110.34 – -111.39 (m, 1F), -112.84 – -113.79 (m, 1F), -121.50 – -121.80 (m, 2F), -121.84 – -122.16 (m, 4F), -122.62 – -122.95 (m, 2F), -123.56 – -123.86 (m, 2F), -126.11 – -126.35 (m, 2F). **HRMS** (**ESI**): $[M+H]^+$ Calcd. for $C_{25}H_{26}NOF_{17}H$ 680.1815; Found: 680.1808. **FTIR** (neat): ν(cm^{-1}) 2933, 1456, 1377, 1362, 1280, 1239, 1203, 1145, 1134, 1115, 1073, 1006, 957, 916, 877, 760, 697, 655, 552, 529, 490, 480, 468, 455, 429, 417.

2,2,6,6-Tetramethyl-1-(3,4,4,4-tetrafluoro-1-phenyl-3-(trifluoromethoxy)butoxy)piperidine (**6u**)

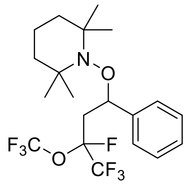

The reaction was performed according to the **GP 3** with 1,1,1,2-tetrafluoro-2-iodo-2-(trifluoromethoxy)ethane(39 µL, 0.20 mmol, 1 equiv.) and styrene (115 µL, 1.0 mmol, 5 equiv.). Flash column chromatography (pentane/Et_2O, 100/0 to 99/1) afforded 2,2,6,6-tetramethyl-1-(3,4,4,4-tetrafluoro-1-phenyl-3-(trifluoromethoxy)butoxy)piperidine **6u** (59.6 mg, 67%, d:r 1:1) as a colourless oil. **^{1}H NMR** (300 MHz, $CDCl_3$, isomers) δ 7.47 – 7.27 (m, 5H), 5.12 – 4.95 (m, 1H), 3.36 – 3.12 (m, 1H), 2.87 – 2.53 (m, 1H), 1.64 – 1.21 (m, 9H), 1.14 (s, 3H), 1.01 (s, 3H), 0.67 (d, *J* = 17.7 Hz, 3H). **^{13}C{^{19}F} NMR** (126 MHz, $CDCl_3$, isomers) δ 139.5, 137.5, 127.7, 127.6, 126.8, 126.70, 126.66, 125.5, 109.4, 109.2, 108.6, 108.4, 106.6, 90.5, 88.9, 78.8, 58.8, 58.4, 39.0, 32.7, 29.0, 28.9, 19.0, 15.7.**^{19}F NMR** (564 MHz, $CDCl_3$, isomers) δ -53.02 – -53.41 (m, 3F), -82.14 – -83.17 (m, 3F), -129.30 – -131.99 (m, 1F).**HRMS** (**ESI**): $[M+H]^+$ Calcd. for $C_{20}H_{26}NO_2F_7H$ 446.1924; Found: 446.1923. **FTIR** (neat): ν(cm^{-1}) 2975, 2933, 1457, 1378, 1362, 1327, 1232, 1180, 1133, 1100, 1080, 1064, 1027, 1003, 975, 957, 916, 876, 807, 759, 697, 652, 605, 548, 530.

2,2,6,6-Tetramethyl-1-(2-(perfluorocyclohexyl)-1-phenylethoxy)piperidine (6v)

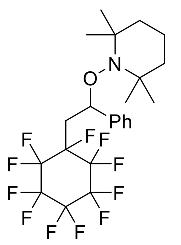

The reaction was performed according to the **GP 3** with 1,1,2,2,3,3,4,4,5,5,6-undecafluoro-6-iodocyclohexane (38 µL, 0.20 mmol, 1 equiv.) and styrene (115 µL, 1.0 mmol, 5 equiv.). Flash column chromatography (pentane/Et_2O, 100/0 to 99/1) afforded 2,2,6,6-tetramethyl-1-(2-(perfluorocyclohexyl)-1-phenylethoxy)piperidine **6v** (77.2 mg, 72%) as a white solid. **MP**: 69-71 °C. **^{1}H NMR** (300 MHz, $CDCl_3$) δ 7.40 – 7.27 (m, 5H), 5.21 – 5.09 (m, 1H), 3.43 – 3.20 (m, 1H), 2.89 – 2.69 (m, 1H), 1.64 – 1.45 (m, 3H), 1.43 – 1.24 (m, 6H), 1.14 (s, 3H), 1.01 (s, 3H), 0.65 (s, 3H). **^{13}C{^{19}F} NMR** s(126 MHz, $CDCl_3$) δ 138.9, 136.9, 127.1, 127.02, 126.95, 126.13, 126.06, 126.0, 125.5, 124.9, 108.8, 108.5, 107.9, 107.7, 106.0, 105.3, 89.9, 88.2, 78.1, 58.1, 57.8, 38.3, 32.1, 28.4, 28.2, 18.3, 15.1. **^{19}F NMR** (470 MHz, $CDCl_3$) δ -117.68 (t, *J* = 318.4 Hz, 2F), -120.49 – -125.03 (m, 3F), -127.77 – -135.75 (m, 1F), -140.57 (dd, *J* = 1444.0, 284.9 Hz, 3F), -180.24 – -187.48 (m, 2F). **HRMS** (**ESI**): $[M+H]^+$ Calcd. for $C_{23}H_{26}NOF_{11}H$ 542.1911; Found: 542.1913. **FTIR** (neat): ν(cm^{-1}) 2975, 2935, 1736, 1456, 1378, 1362, 1315, 1259, 1232, 1208, 1178, 1125, 1086, 1033, 1005, 975, 933, 876, 799, 758, 698, 633, 620, 582, 548.

2,2,6,6-Tetramethyl-1-((3,3,4,4-tetrafluoro-1-phenylhex-5-en-1-yl)oxy)piperidine (6w)

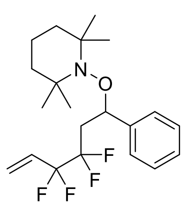

The reaction was performed according to the **GP 3** with 4-bromo-3,3,4,4-tetrafluorobut-1-ene (29 µL, 0.20 mmol, 1 equiv.) and styrene (115 µL, 1.0 mmol, 5 equiv.) and the reaction was stirred for 18 h. Flash column chromatography (pentane/Et_2O, 100/0 to 97/3) afforded 2,2,6,6-tetramethyl-1-((3,3,4,4-tetrafluoro-1-phenylhex-5-en-1-yl)oxy)piperidine **6w** (21.6 mg, 28%) as a colourless oil. **^{1}H NMR** (500 MHz, $CDCl_3$) δ 7.38 – 7.26 (m, 5H), 5.91 (dd, *J* = 17.4, 10.8 Hz, 1H), 5.81 (dd, *J* = 17.3, 1.0 Hz, 1H), 5.65 (dd, *J* = 10.8, 1.0 Hz, 1H), 5.06 (dd, *J* = 10.2, 3.3 Hz, 1H), 3.24 – 2.92 (m, 1H), 2.64 – 2.35 (m, 1H), 1.65 – 1.23 (m, 9H), 1.16 (s, 3H), 1.01 (s, 3H), 0.61 (s, 3H). **^{13}C{^{19}F} NMR** (126 MHz, $CDCl_3$) δ 142.3, 128.1, 127.9, 127.7, 126.4, 123.8, 118.0, 115.0, 80.2, 60.1, 59.6, 40.4, 35.9, 34.2, 34.0, 20.4, 17.1. **^{19}F NMR** (470 MHz, $CDCl_3$) δ -111.49 (dd, *J* = 264.0, 4.9 Hz, 1F), -114.48 (dd, *J* = 264.1, 4.3 Hz, 1F), -115.42 (dd, *J* = 13.1, 4.4 Hz, 2F). **HRMS** (**ESI**): $[M+H]^+$ Calcd. for $C_{21}H_{29}NOF_4H$ 388.2258; Found: 388.2257. **FTIR** (neat): ν(cm^{-1}) 2973, 2931, 1653, 1495, 1456, 1420, 1376, 1362, 1257, 1239, 1187, 1112, 1069, 1047, 1026, 1008, 980, 955, 920, 877, 809, 759, 726, 696, 626, 616, 574, 551, 529, 505.

Methyl 3-(adamantan-1-yl)-2-oxopropanoate (7)

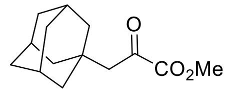

In a reaction vessel containing compound **4a** (57 mg, 0.15 mmol, 1 equiv.) in CH_2Cl_2 (6.5 mL) was added *m*-CPBA (44 mg, 0.19 mmol, 1.3 equiv., 70-75% in water) and then the reaction mixture was stirred 1 h at room temperature. Solvent was then evaporated and Flash column chromatography (pentane/Et_2O, 100/0 to 97/3) afforded methyl 3-(adamantan-1-yl)-2-oxopropanoate **7** (21 mg, 59%) as a white solid. **MP**: 46-47 °C. **^{1}H NMR** (300 MHz, $CDCl_3$) δ 3.84 (s, 3H), 2.60 (s, 2H), 2.00 – 1.91 (m, 3H), 1.72 – 1.58 (m, 12H). **^{13}C NMR** (76 MHz, $CDCl_3$) δ 194.3, 162.1, 52.9, 51.4, 42.3, 36.6, 34.4, 28.5. **HRMS** (**ESI**): $[M+Na]^+$ Calcd. for $C_{14}H_{20}O_3Na$ 259.1304; Found: 259.1301. **FTIR** (neat): ν(cm^{-1}) 2899, 2847, 1724, 1451, 1279, 1241, 1208, 1168, 1107, 1078, 486, 426.

3,3,4,4,5,5,6,6,6-Nonafluoro-1-phenylhexan-1-ol (8)

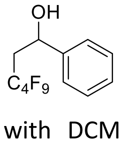

In a reaction vessel containing compound **6a** (0. 2 mmol, 96 mg), 0.6 mL of water, 2 mL of acetic acid was added Zn dust (70 mg, 1 mmol, 5 equiv.) in two portions with the time interval of 3 h and the reaction mixture was vigorously stirred for overnight at room temperature. Then 2 (M) aqueous NaOH solution was added into the reaction mixture. The resulting reaction mixture was extracted with DCM and organic layer was dried over $MgSO_4$. Solvent was then evaporated and flash column chromatography (pentane/EtOAc, 100/0 to 90/10) afforded 3,3,4,4,5,5,6,6,6-nonafluoro-1-phenylhexan-1-ol **8** (41 mg, 60%) as a colourless oil. **^{1}H NMR** (300 MHz, $CDCl_3$) δ 7.54 – 7.28 (m, 5H), 5.30 – 5.12 (m, 1H), 2.75 – 2.29 (m, 2H), 2.23 (s, 1H). **^{13}C{^{19}F} NMR** (126 MHz, $CDCl_3$) δ 142.6, 128.9, 128.4, 125.6, 117.7, 117.4, 110.3, 108.8, 68.0, 39.8. **^{19}F NMR** (564 MHz, $CDCl_3$) δ -80.09 – -81.87 (m, 3F), -111.96 – -113.20 (m, 1F), -113.44 – -114.65 (m, 1F), -123.90 – -125.05 (m, 2F), -125.57 – -126.40 (m, 2F). **HRMS** (**ESI**): $[M-H]^-$ Calcd. for $C_{12}H_8OF_9$ 339.0436; Found: 339.0432. **FTIR** (neat): ν(cm^{-1}) 3388, 2924, 1496, 1456, 1355, 1217, 1130, 1073, 1026, 917, 881, 855, 793, 755, 728, 712, 699, 659, 587, 559, 531.

10. Reference

[1] M. Hartmann, Y. Li and A. Studer, *J. Am. Chem. Soc.,* 2012, **134**, 16516.

[2] Y. Chen, L. Su and H. Gong, *Org. Lett.,* 2019, **21**, 4689.

[3] A. K. Ravn, M. B. Johansen and T. Skrydstrup, *Angew. Chem. Int. Ed.,* 2022, **61**, e202112390.

[4] M. O. Ratnikov and M. P. Doyle, *J. Am. Chem. Soc.,* 2013, **135**, 1549.

[5] C. S. Lancefield, B. Fölker, R. C. Cioc, K. Stanciakova, R. E. Bulo, M. Lutz, M. Crockatt, and P. C. A. Bruijnincx, *Angew. Chem. Int. Ed.,* 2020, **59**, 23480.

[6] J. Q. Ng, H. Arima, T. Mochizuki, K. Toh, K. Matsui, M. Ratanasak, J.-Y.Hasegawa, M. Hatano and K. Ishihara, *ACS Catal.,* 2021, **11**, 199.

[7] A. Bugarin, K. D. Jones and B. T. Connell, *Chem. Commun.,* 2010, **46**, 1715.

[8] C. Wang and G. A. Russell, *J. Org. Chem.,* 1999, **64**, 2066.

[9] C. Chen, T. R. Dugan, W. W. Brennessel, D. J. Weix and P. L. Holland, *J. Am. Chem. Soc.,* 2014, **136**, 945.

[10] N. J. Adamson, H. Jeddi and S. J. Malcolmson, *J. Am. Chem. Soc.,* 2019, **141**, 8574.

[11] H. Fujioka, J. Shou, R. Kojima, Y. Urano, Y. Ozeki and M. Kamiya, *J. Am. Chem. Soc.,* 2020, **142**, 20701.

[12] A. Cai, W. Yan, C. Wang and W. Liu, *Angew. Chem. Int. Ed.,* 2021, **60**, 27070.

[13] S. Kim, J. R.-Martinab and F. D. Toste *Chem. Sci.,* 2016, **7**, 85.

11. NMR data

4a

^{1}H NMR (500 MHz, $CDCl_3$)

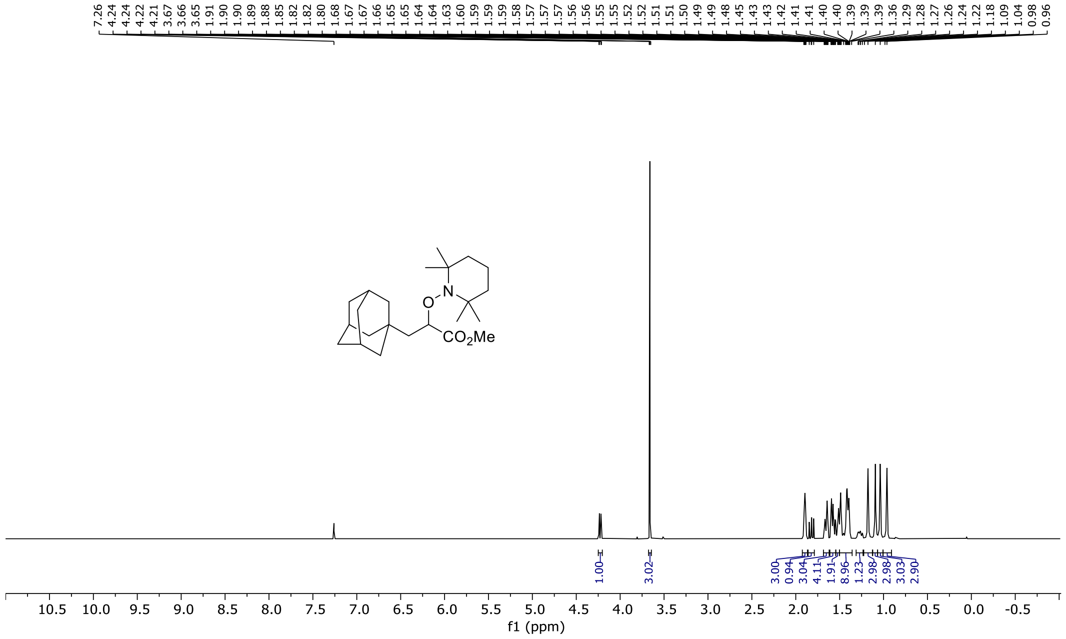

^{13}C NMR (126 MHz, $CDCl_3$)

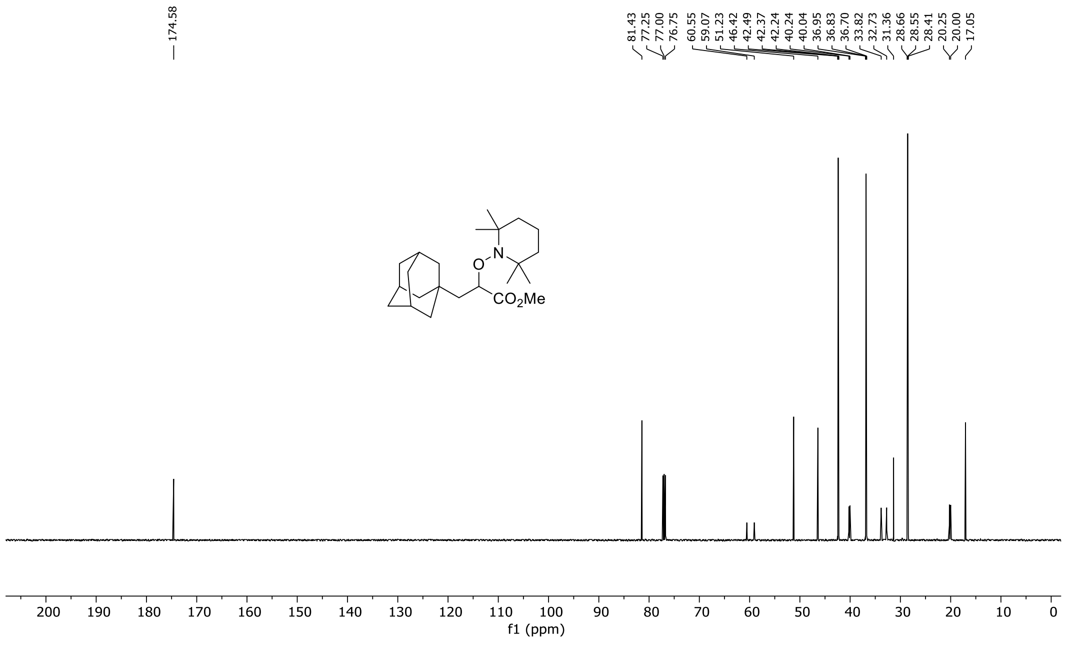

4b

^{1}H NMR (300 MHz, $CDCl_3$)

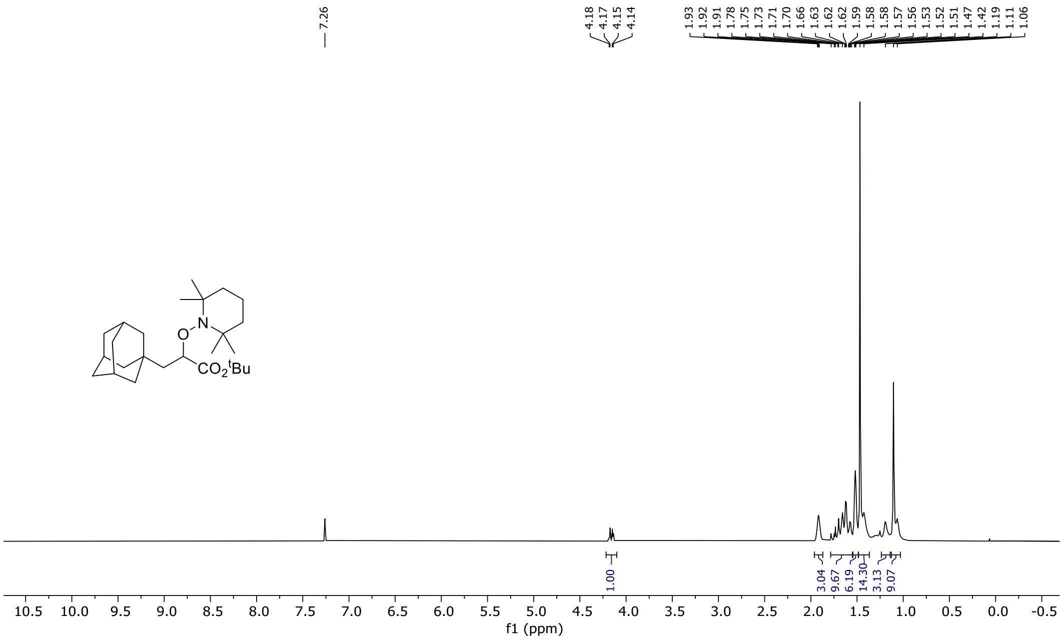

^{13}C NMR (151 MHz, $CDCl_3$)

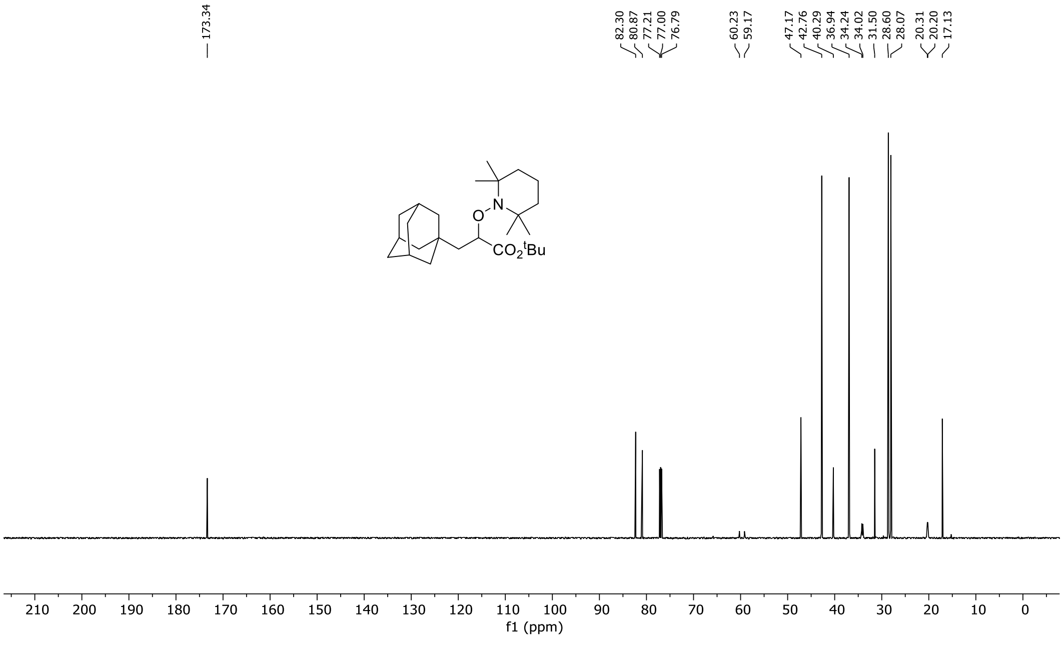

4c

^{1}H NMR (600 MHz, $CDCl_3$)

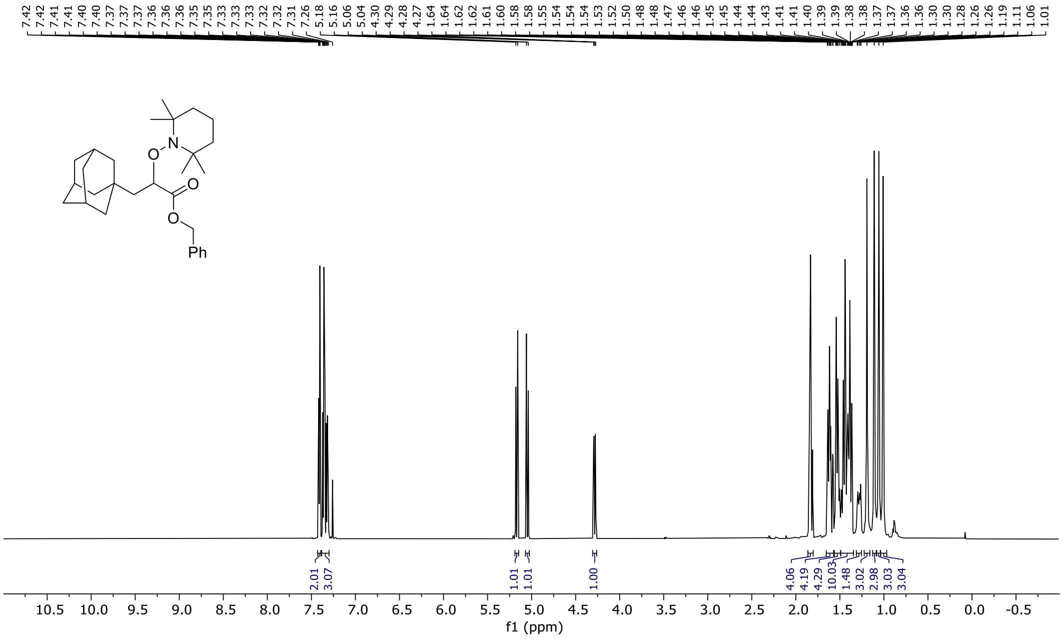

^{13}C NMR (151 MHz, $CDCl_3$)

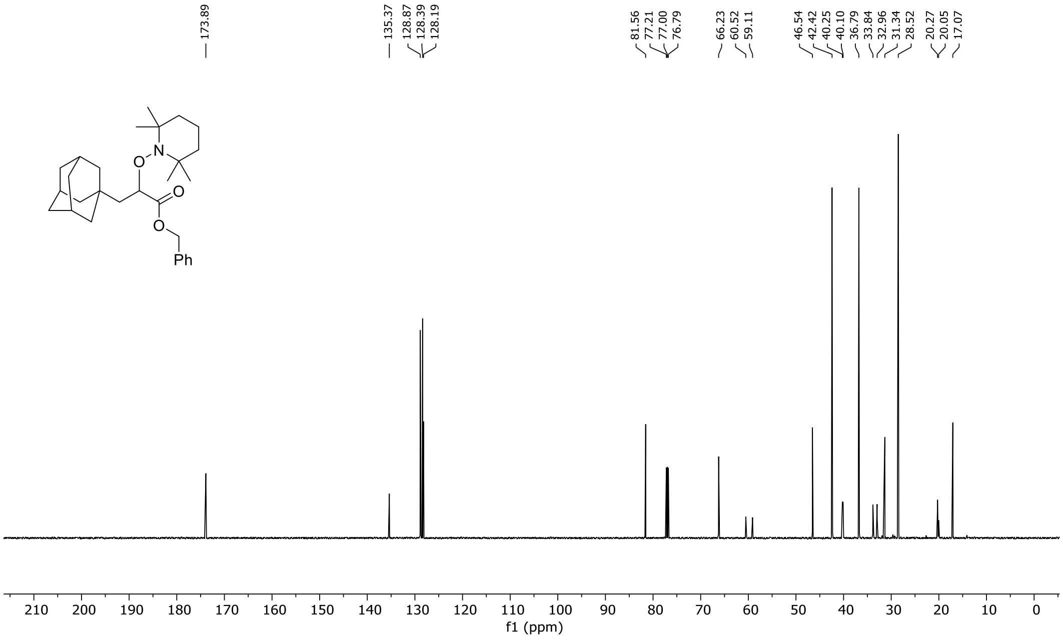

4d

^{1}H NMR (500 MHz, $CDCl_3$)

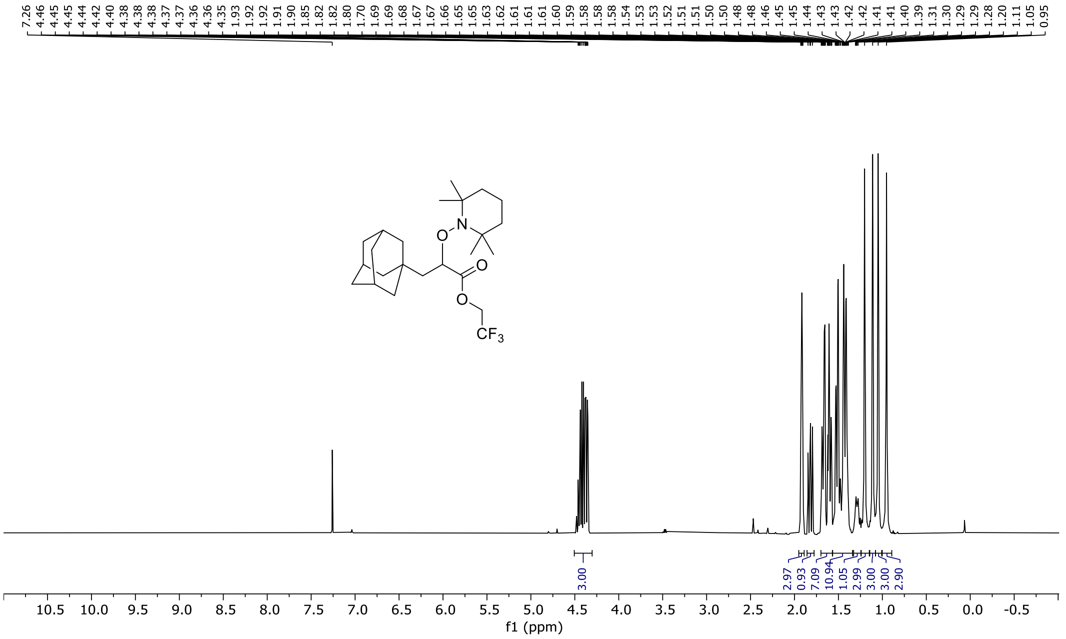

^{13}C NMR (76 MHz, $CDCl_3$)

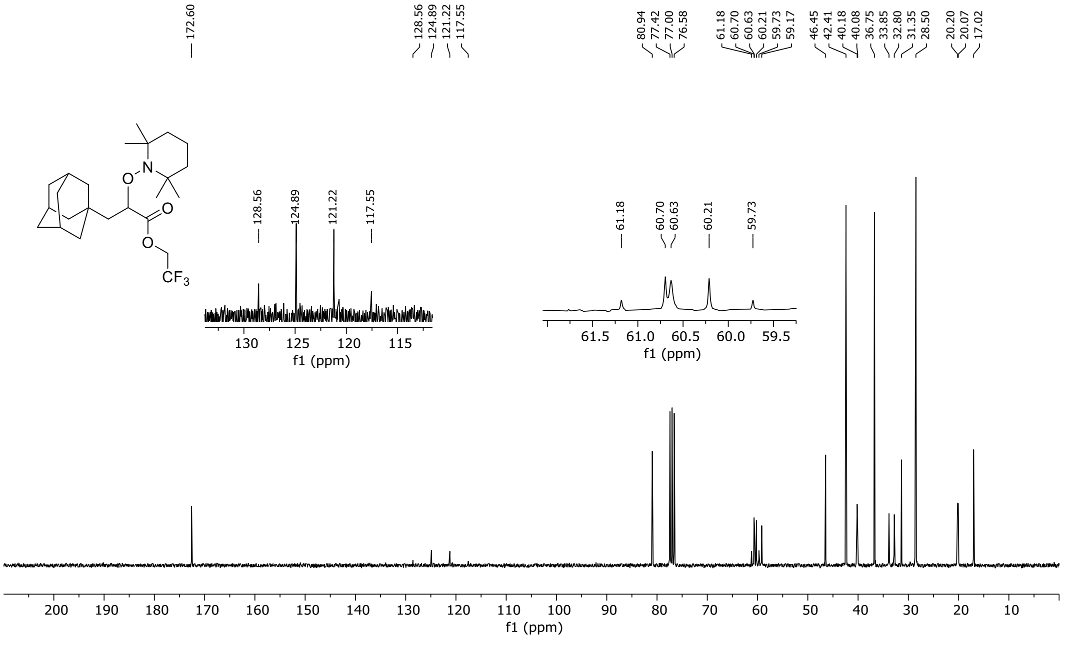

^{19}F NMR (282 MHz, $CDCl_3$)

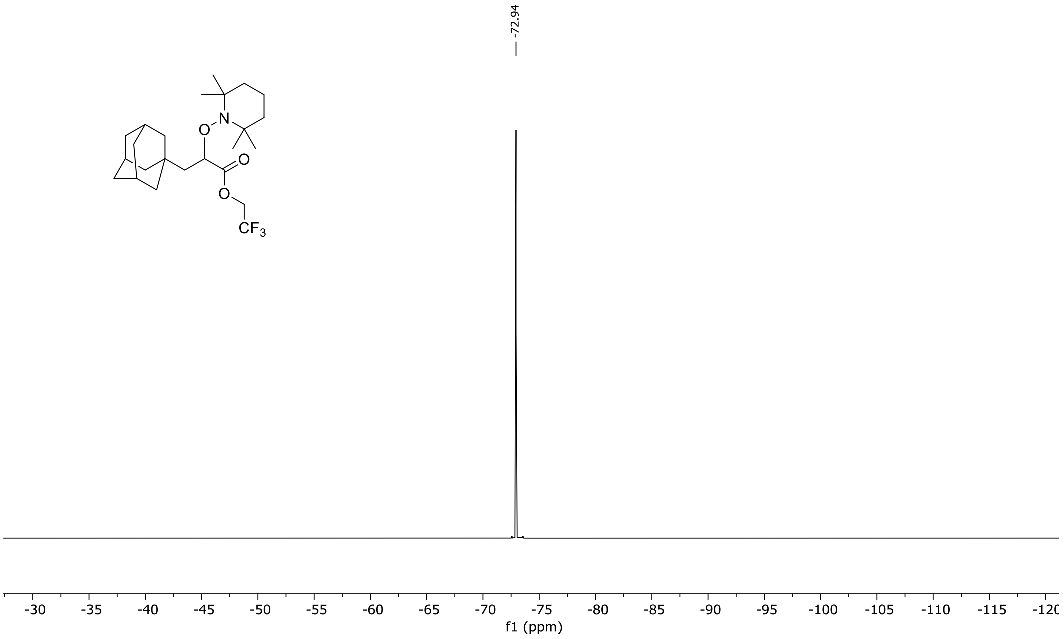

4e

^{1}H NMR (300 MHz, $CDCl_3$)

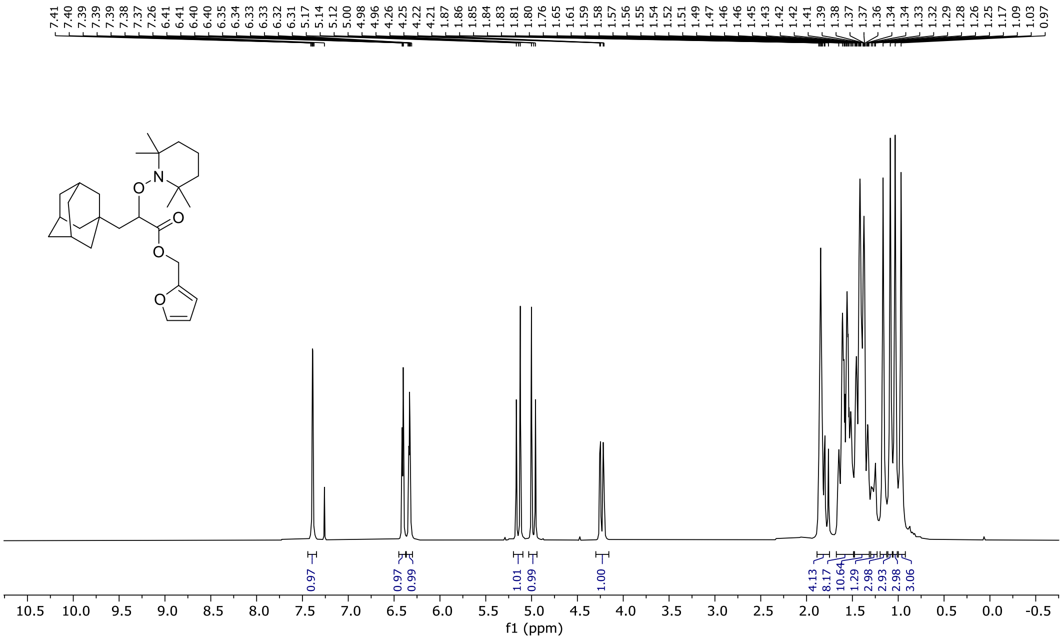

^{13}C NMR (126 MHz, $CDCl_3$)

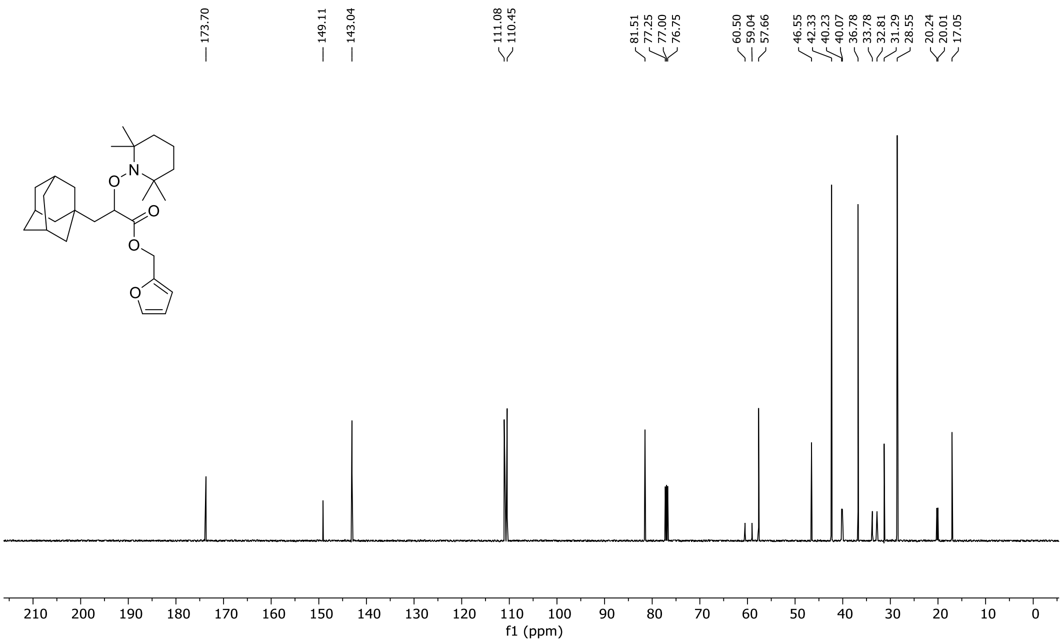

4f

^{1}H NMR (500 MHz, $CDCl_3$)

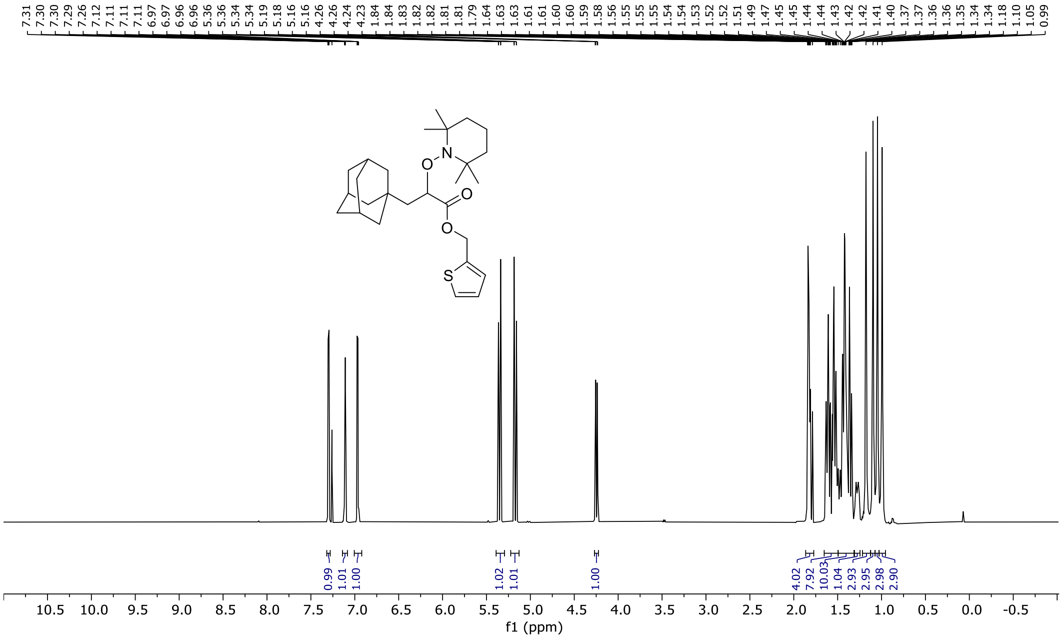

^{13}**C NMR** (126 MHz, $CDCl_3$)

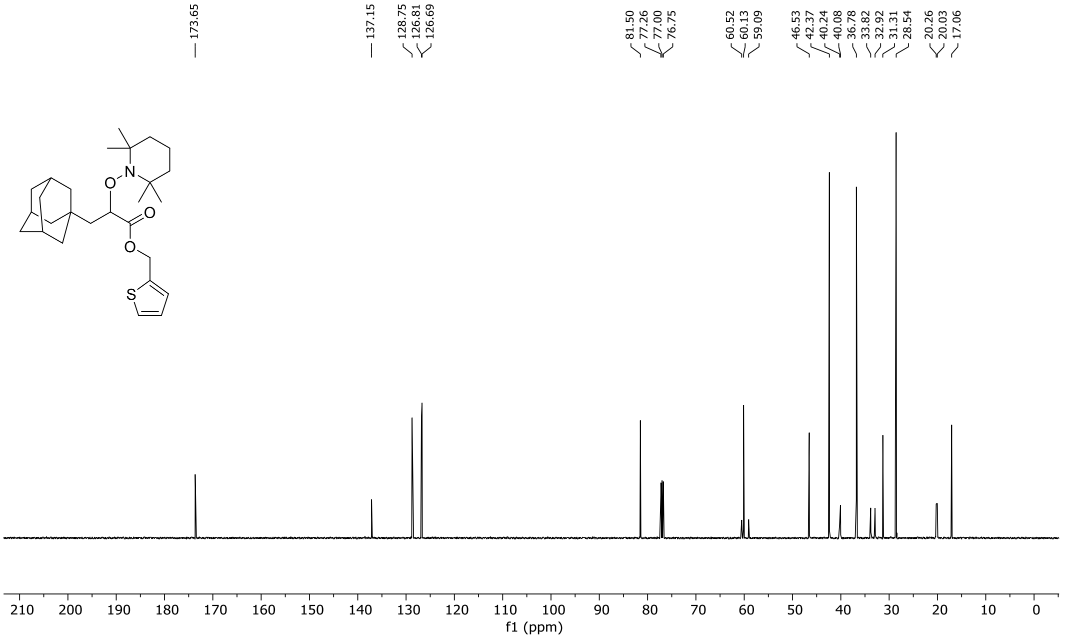

4g

^{1}**H NMR** (300 MHz, $CDCl_3$)

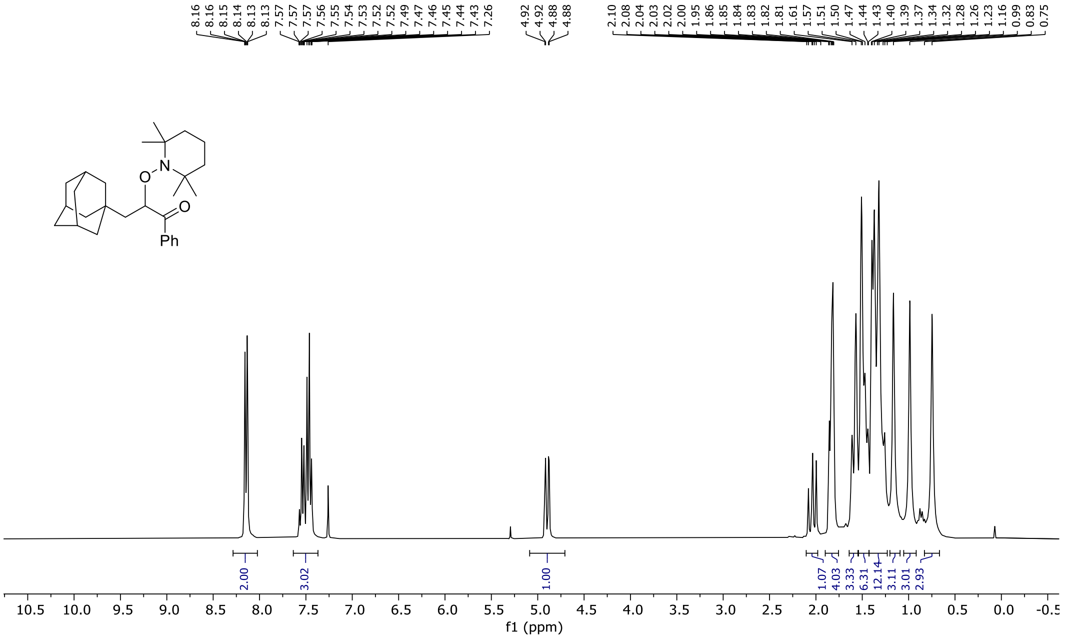

^{13}C NMR (126 MHz, $CDCl_3$)

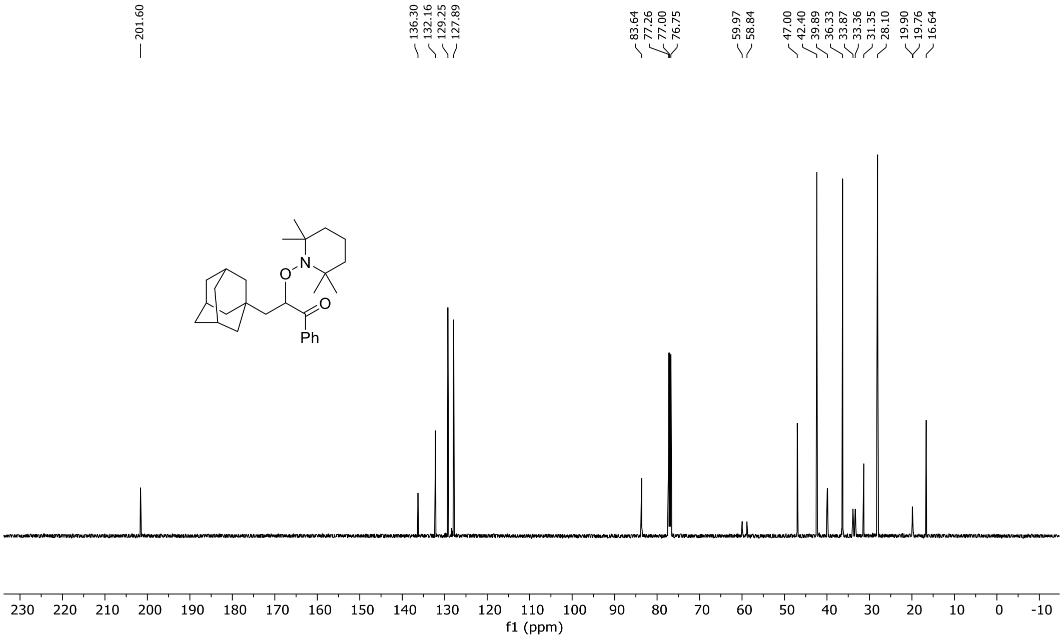

4h

^{1}H NMR (300 MHz, $CDCl_3$)

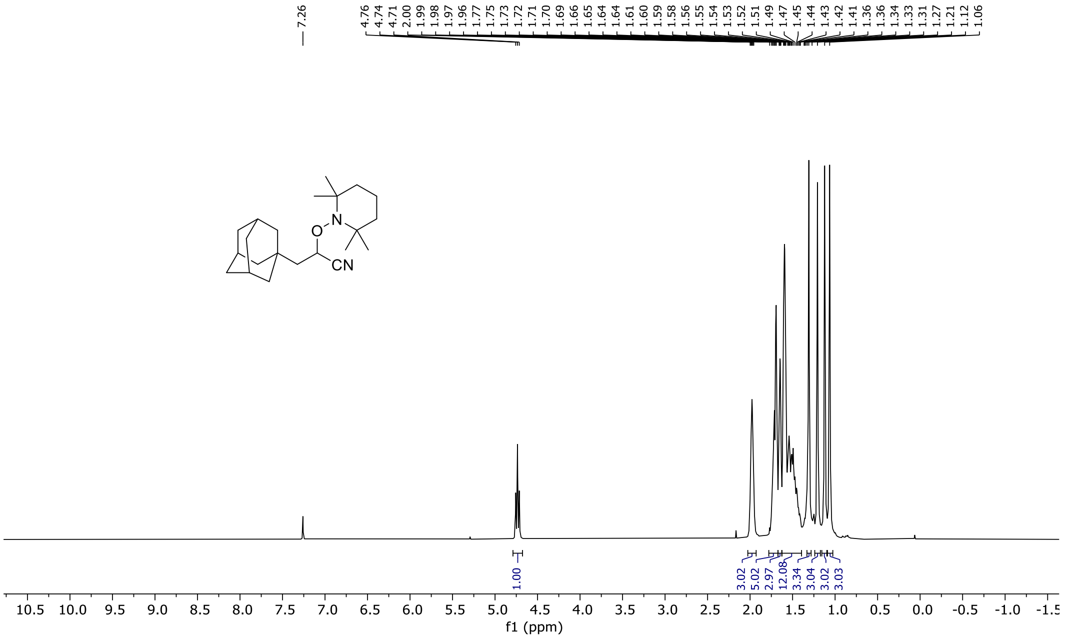

^{13}C NMR (126 MHz, $CDCl_3$)

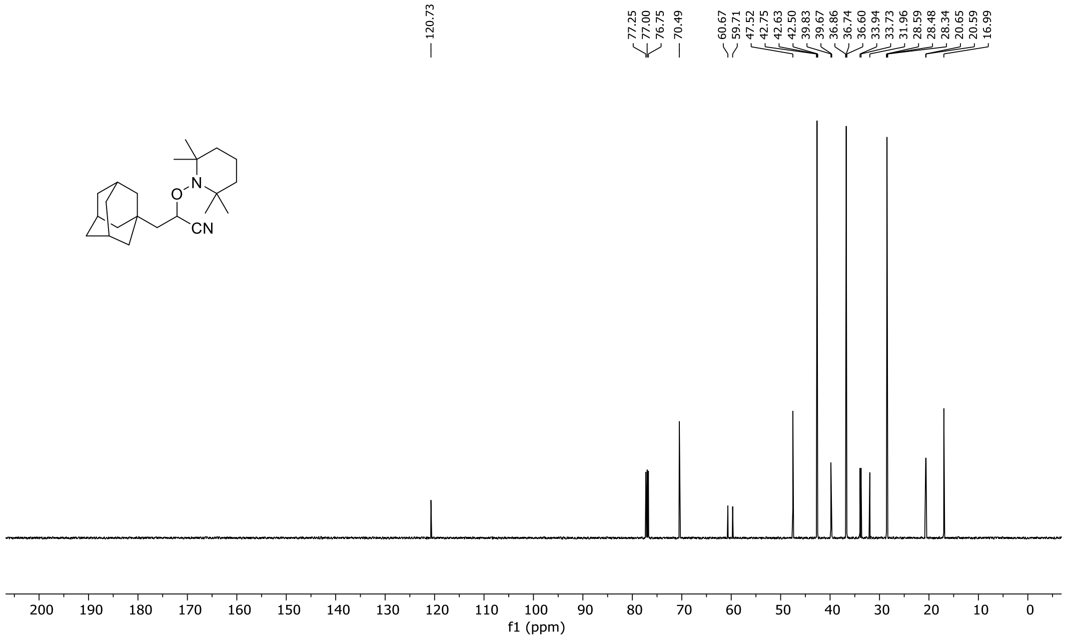

4i

^{1}H NMR (500 MHz, $CDCl_3$)

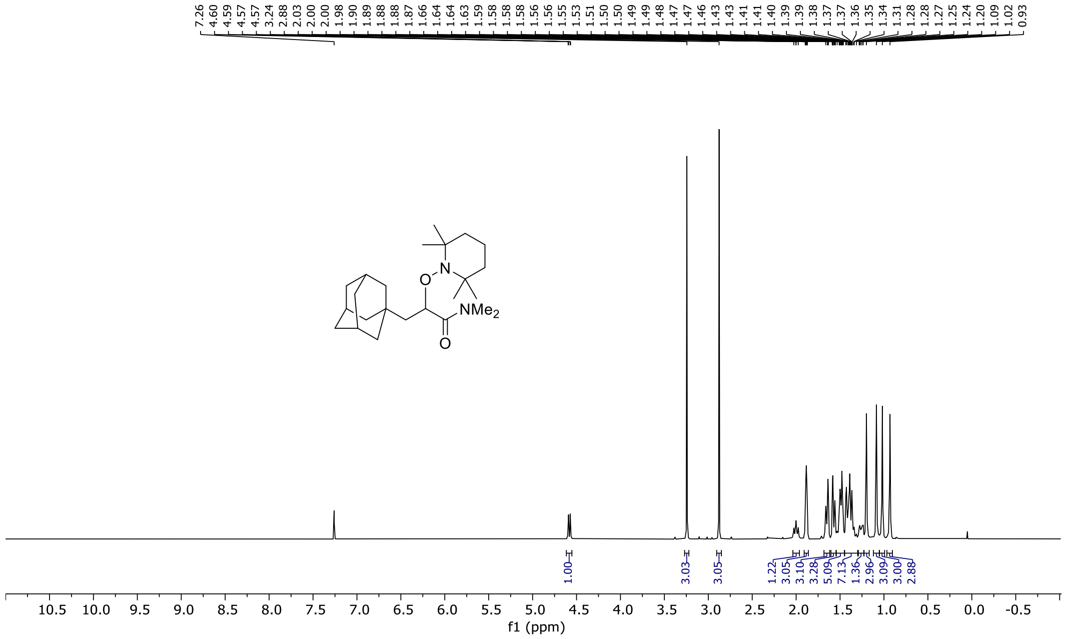

^{13}C NMR (126 MHz, $CDCl_3$)

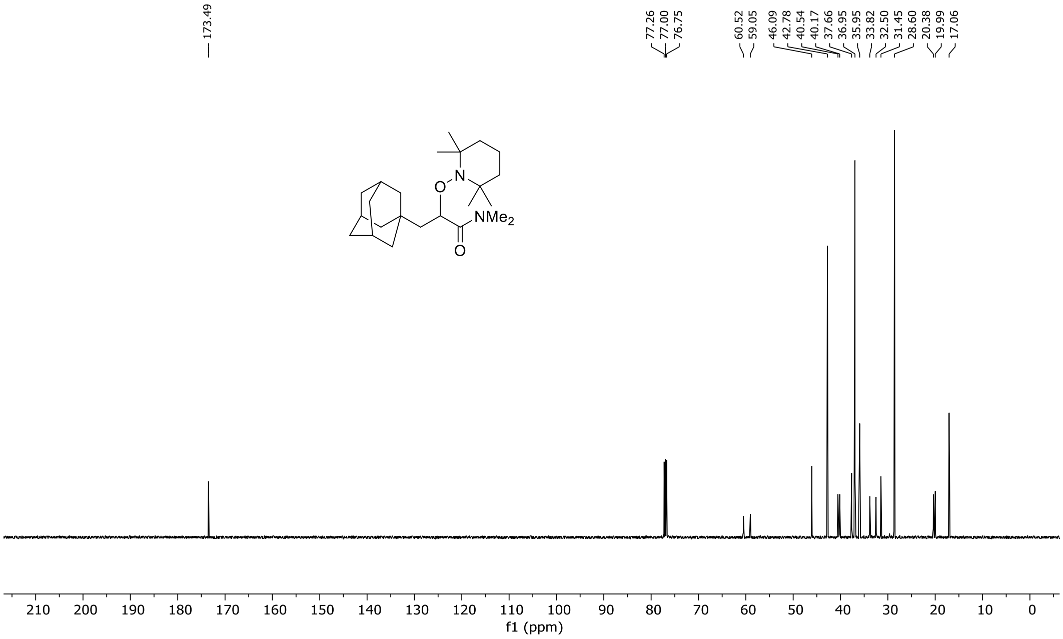

4j

^{1}H NMR (500 MHz, $CDCl_3$)

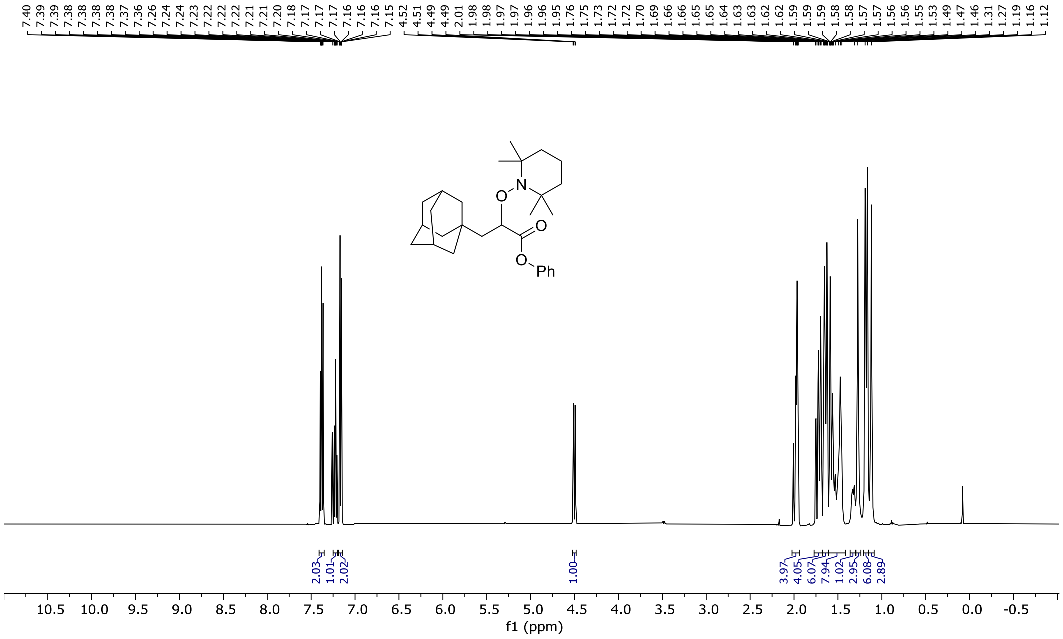

^{13}C NMR (126 MHz, CDCl$_3$)

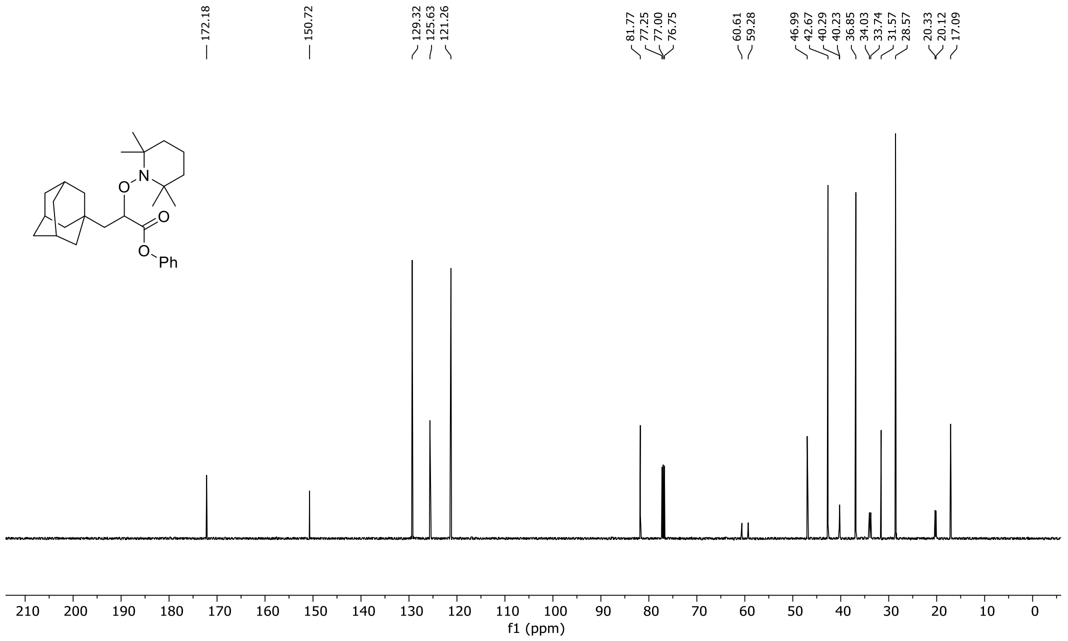

4k

^{1}H NMR (500 MHz, CDCl$_3$)

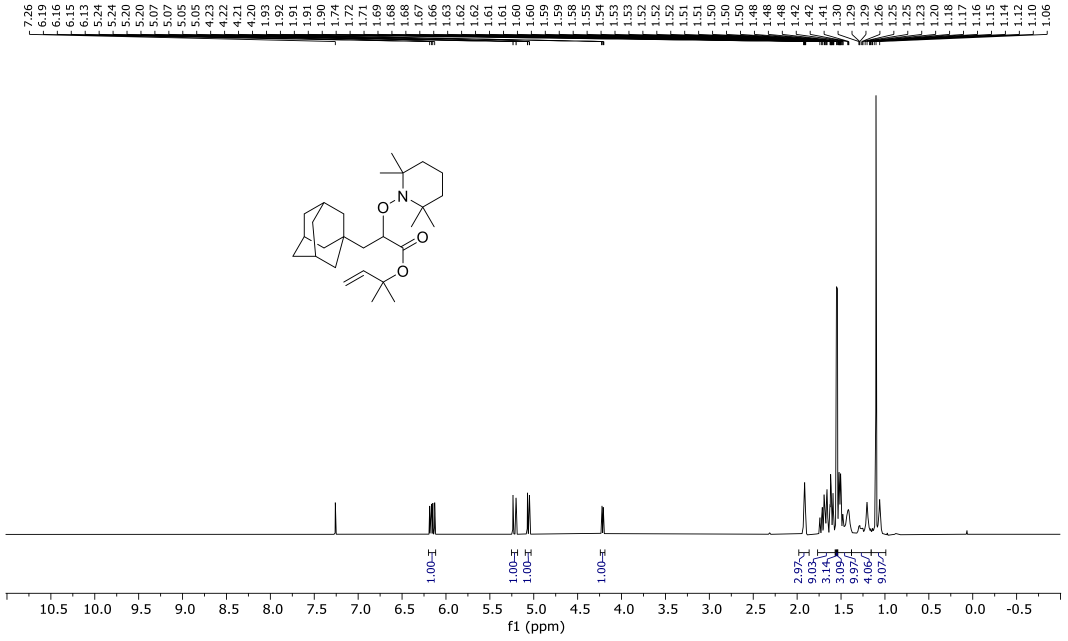

^{13}C NMR (126 MHz, $CDCl_3$)

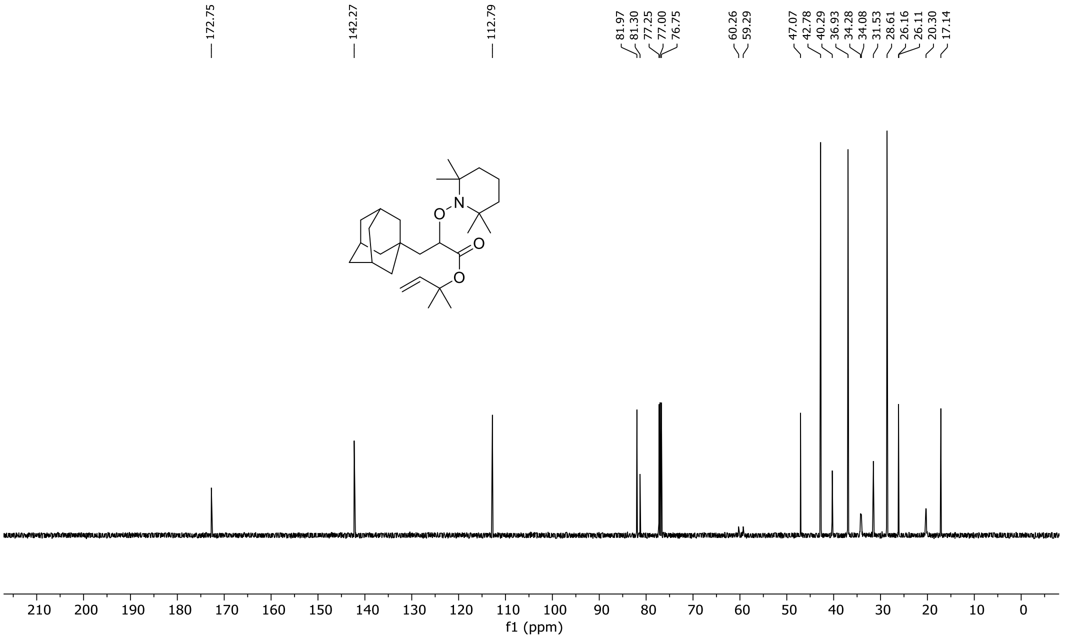

4l

^{1}H NMR (300 MHz, $CDCl_3$)

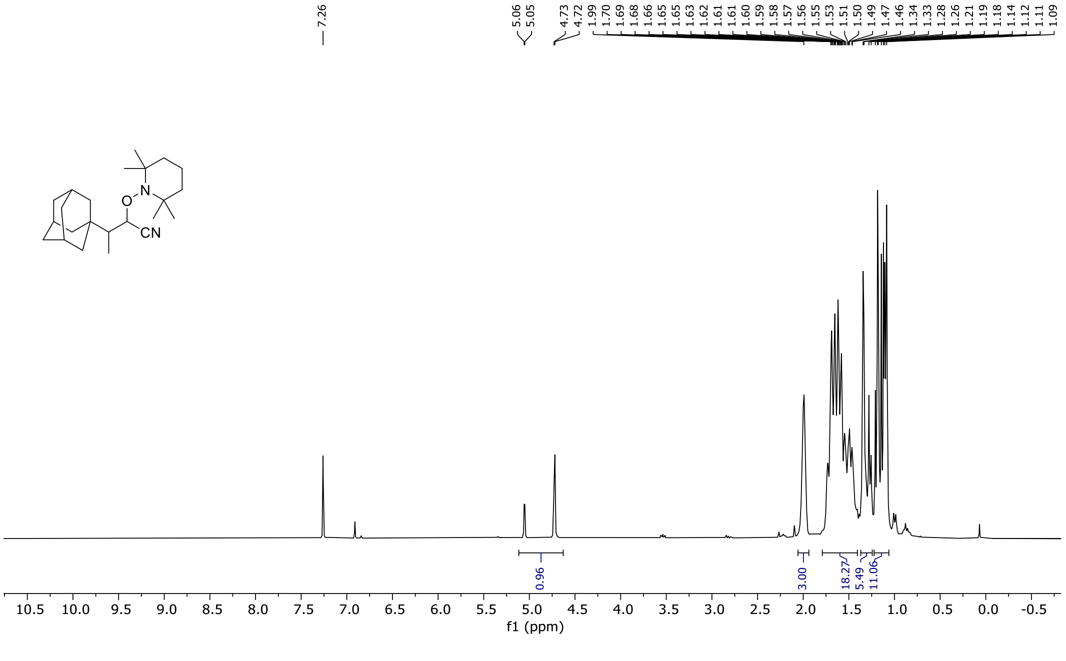

^{13}C NMR (76 MHz, $CDCl_3$)

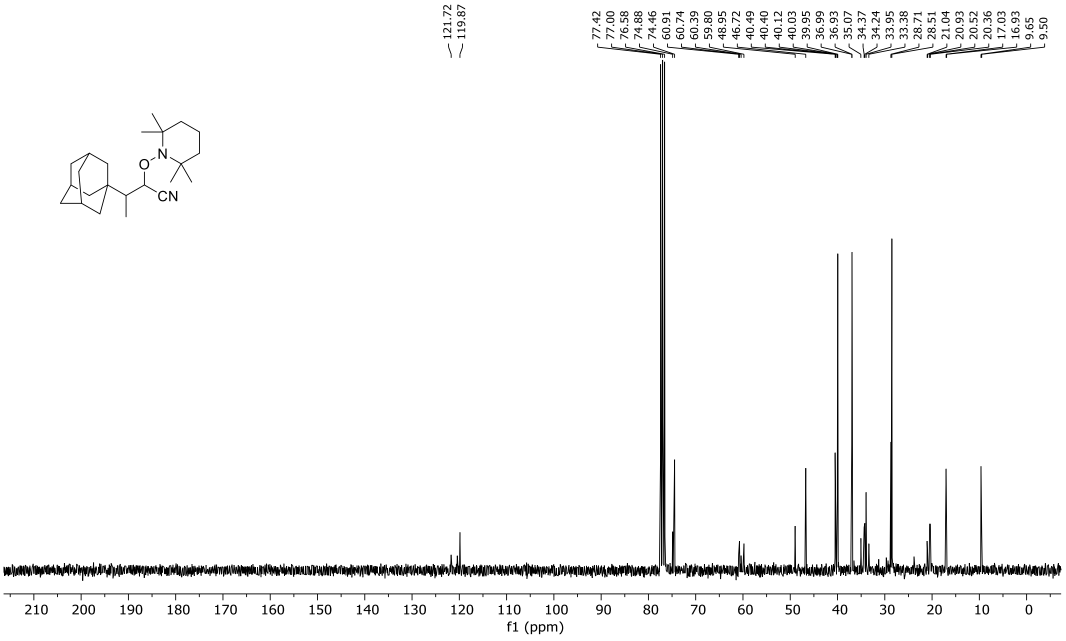

4m

^{1}H NMR (300 MHz, $CDCl_3$)

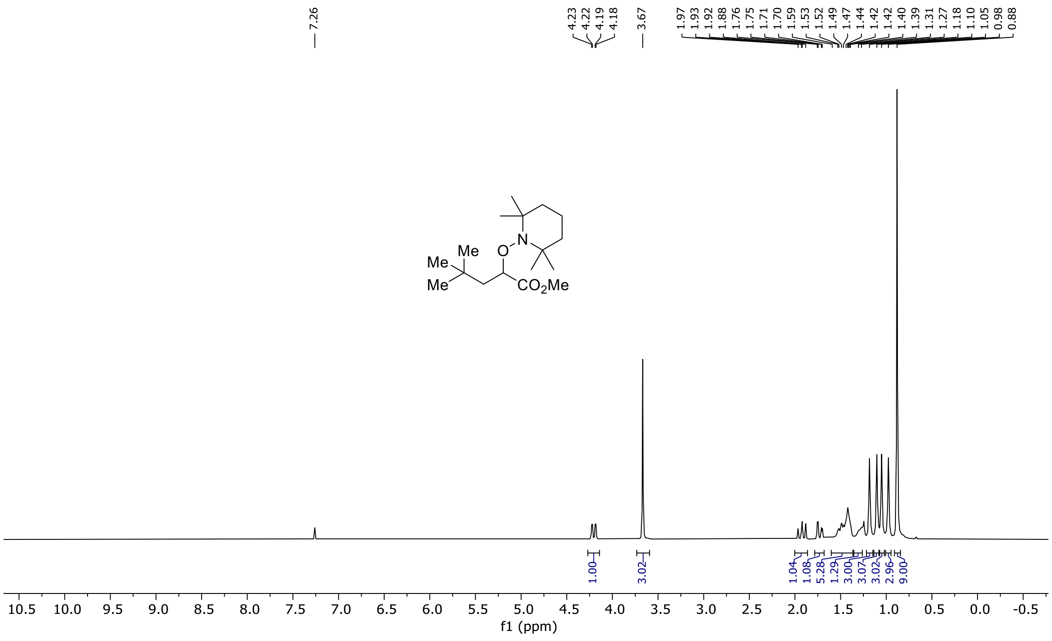

^{13}C NMR (151 MHz, CDCl$_3$)

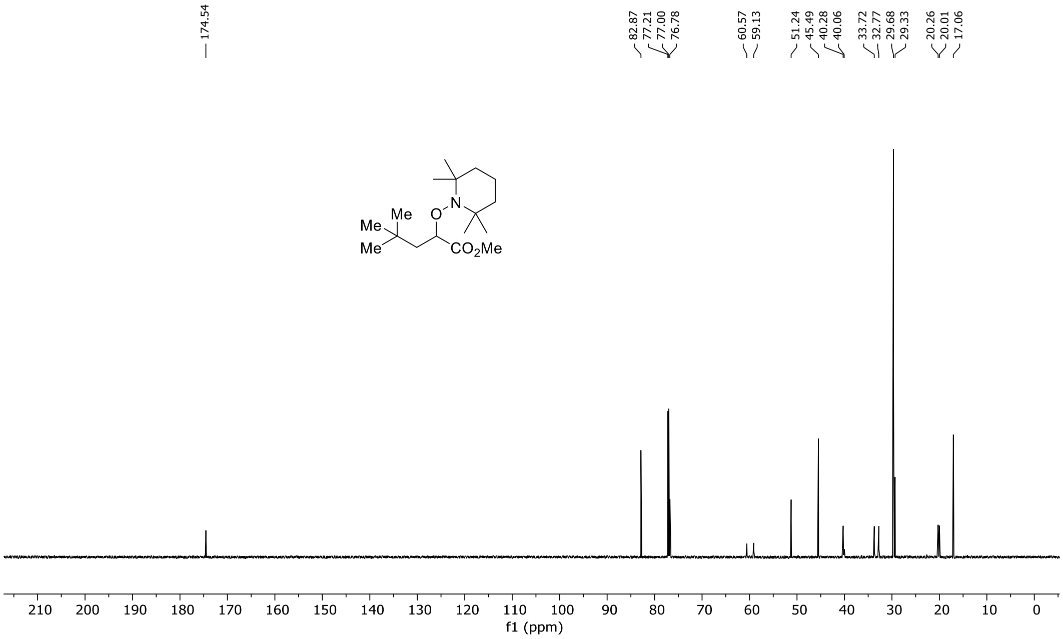

4n

^{1}H NMR (300 MHz, CDCl$_3$)

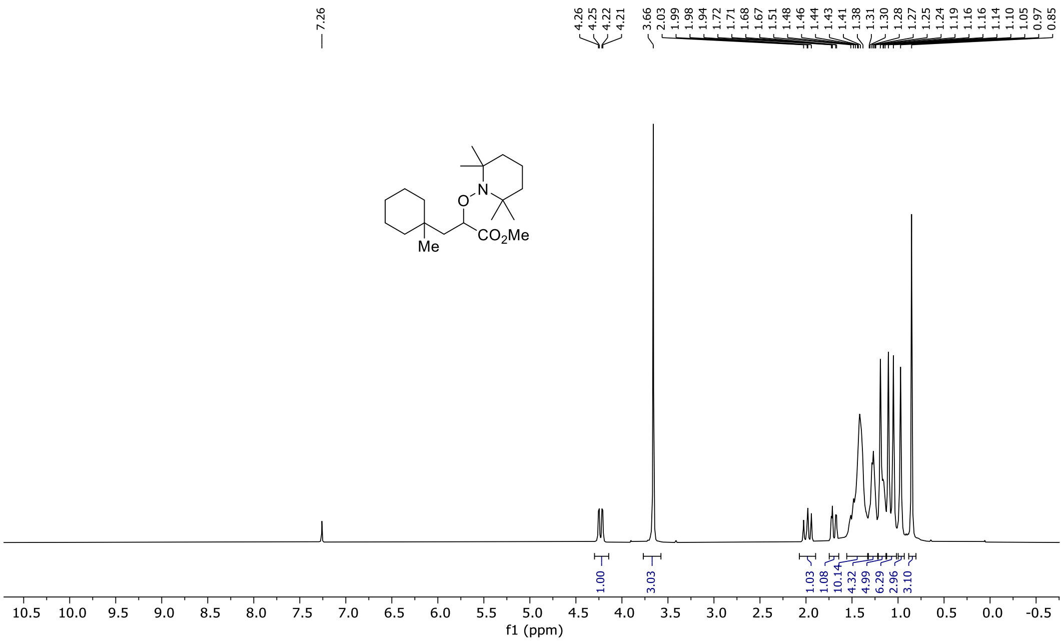

^{13}C NMR (151 MHz, $CDCl_3$)

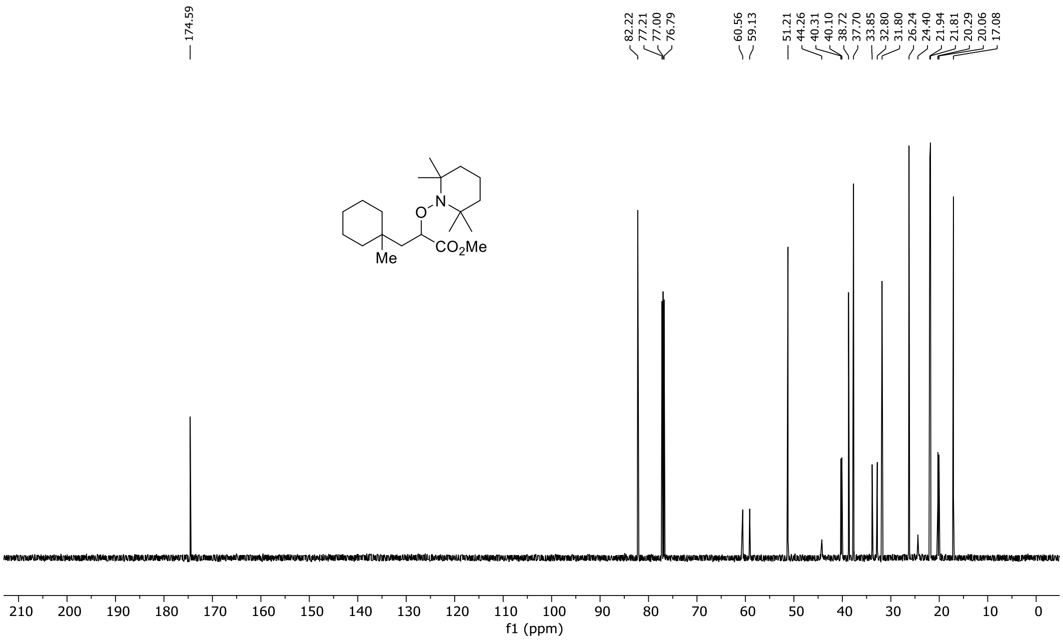

4o

^{1}H NMR (300 MHz, $CDCl_3$)

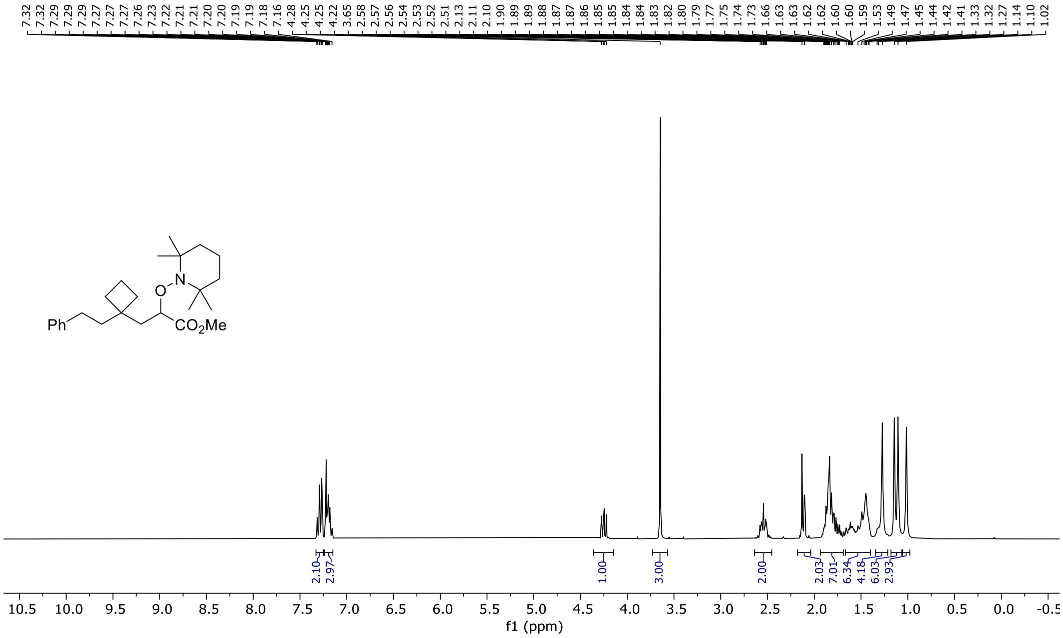

^{13}C NMR (126 MHz, $CDCl_3$)

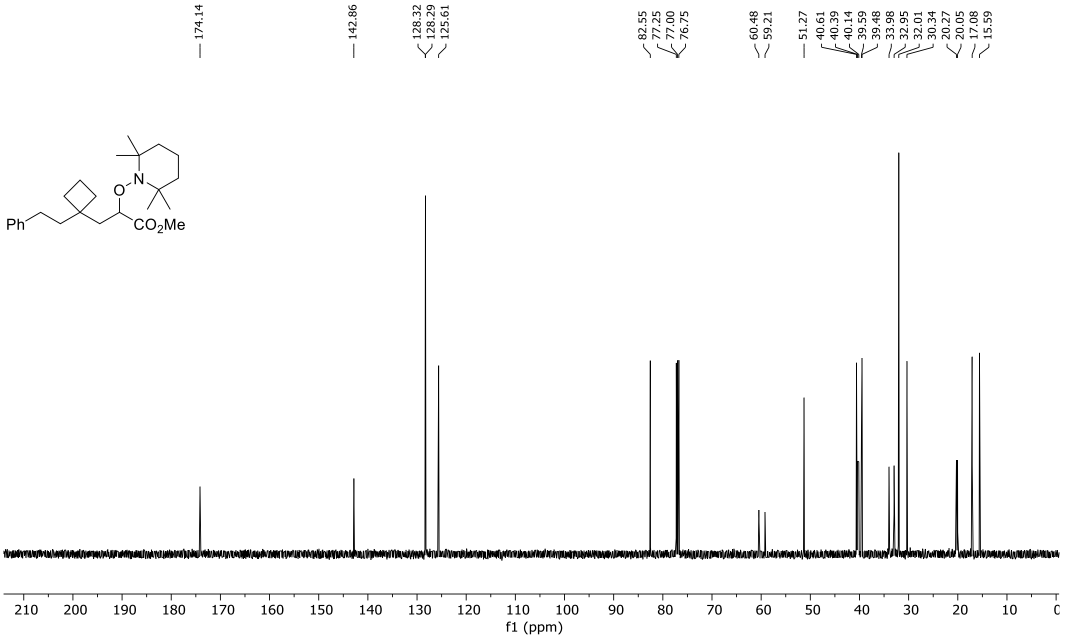

4p

^{1}H NMR (300 MHz, $CDCl_3$)

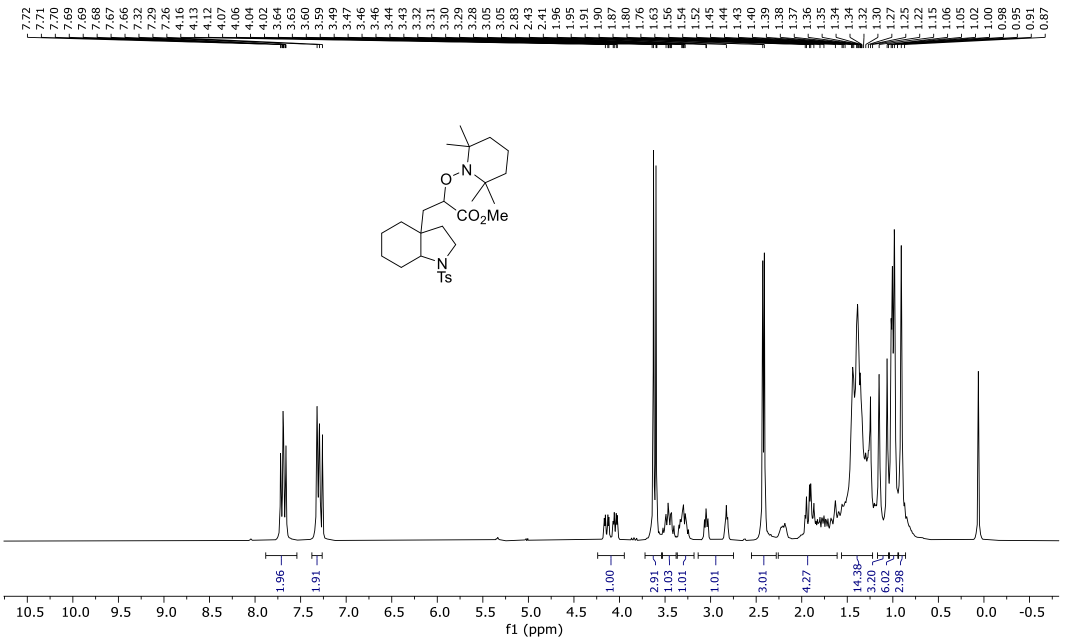

^{13}C NMR (126 MHz, CDCl$_3$)

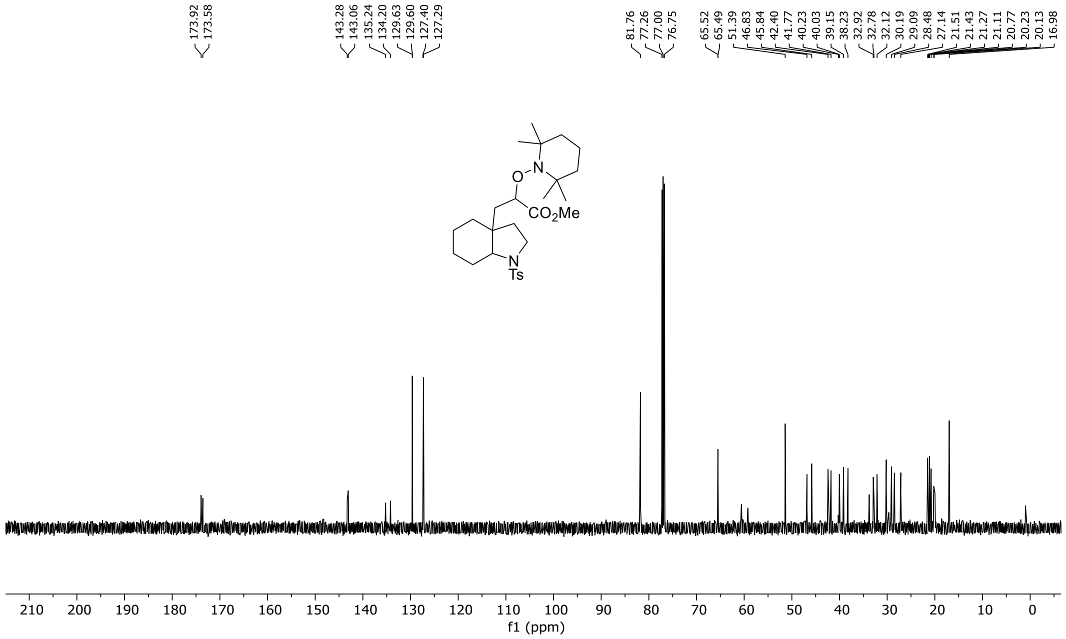

4q

^{1}H NMR (300 MHz, CDCl$_3$)

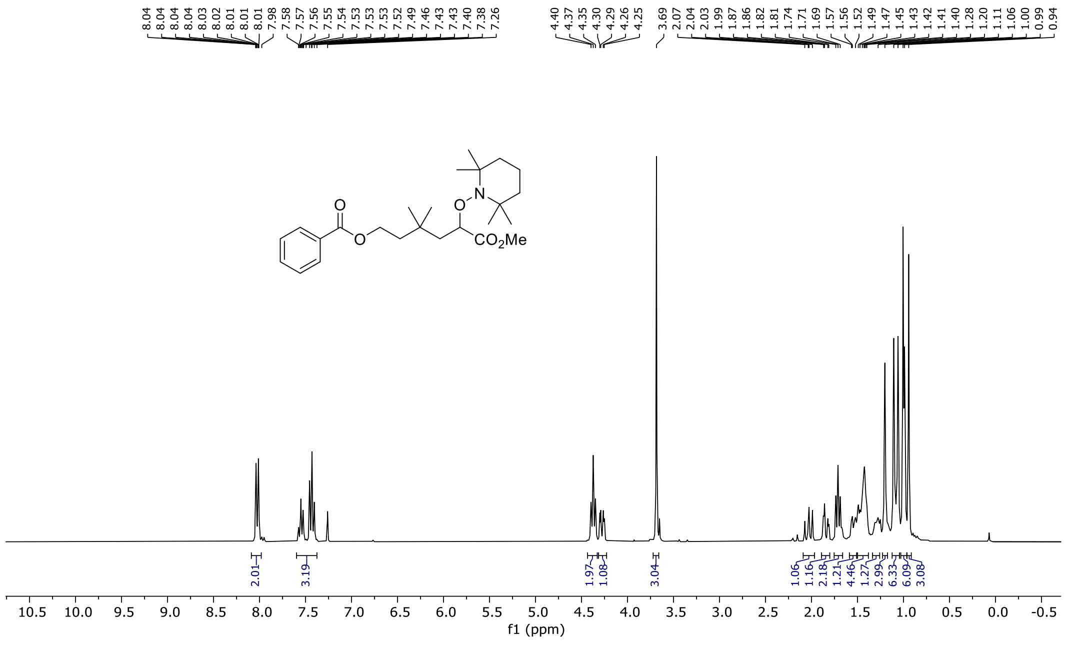

^{13}C NMR (126 MHz, $CDCl_3$)

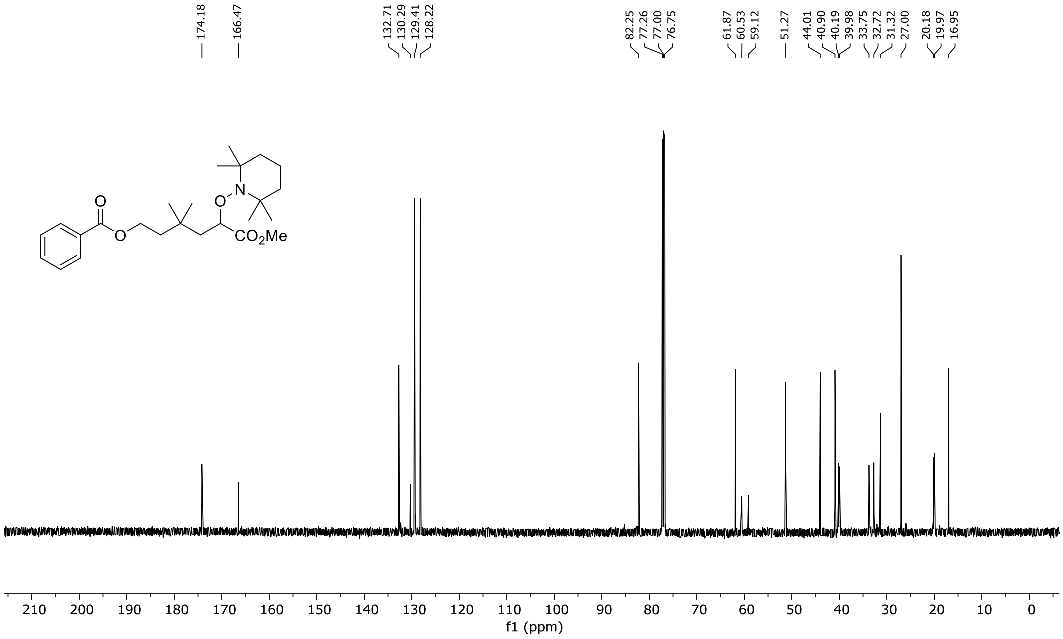

4r

^{1}H NMR (500 MHz, $CDCl_3$)

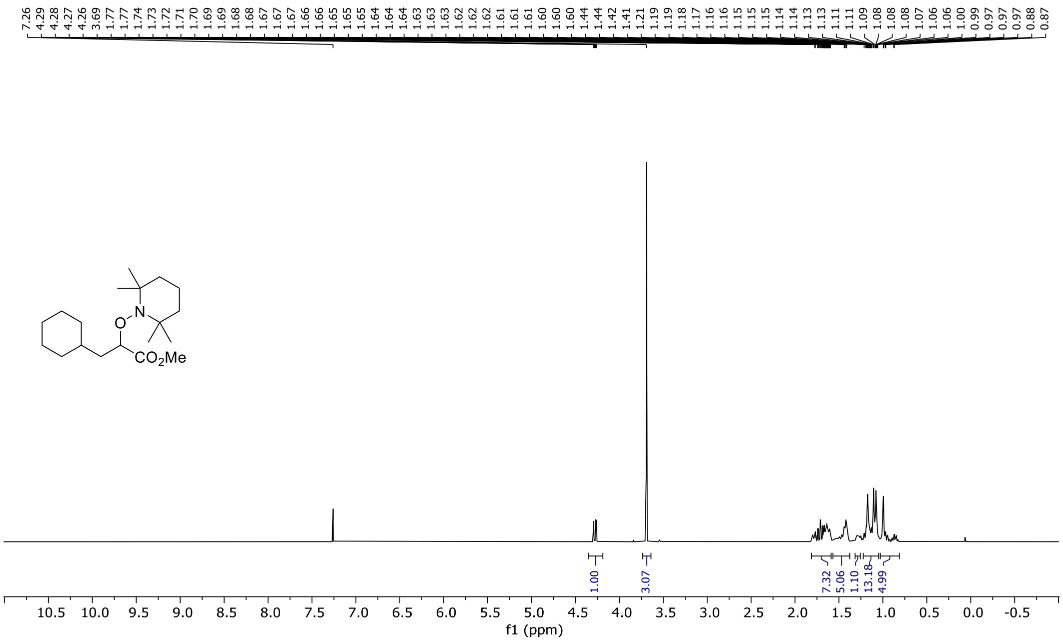

^{13}C NMR (126 MHz, $CDCl_3$)

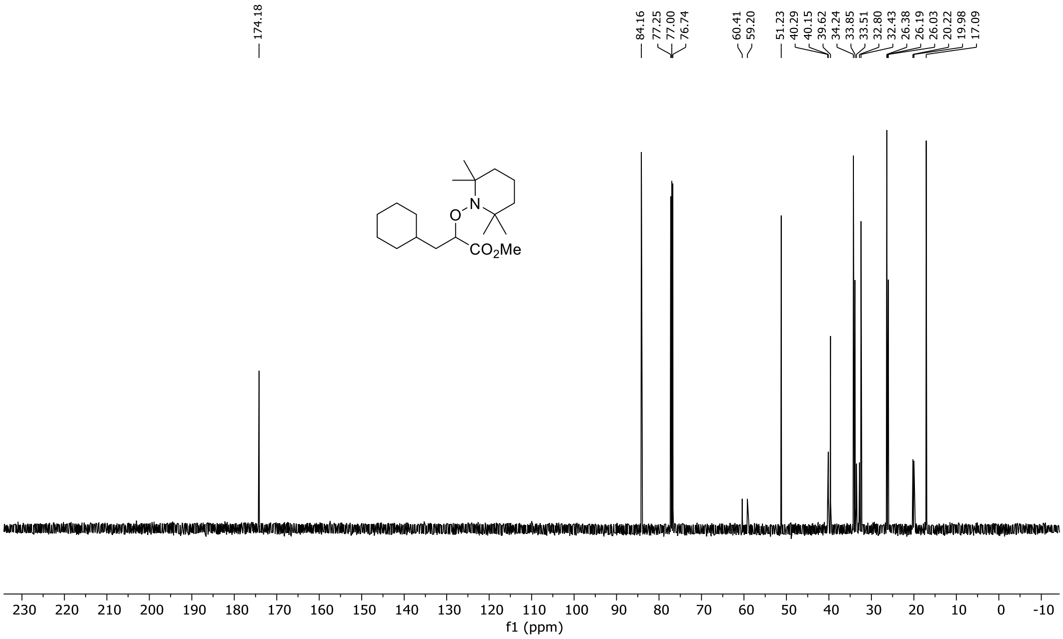

4s

^{1}H NMR (300 MHz, $CDCl_3$)

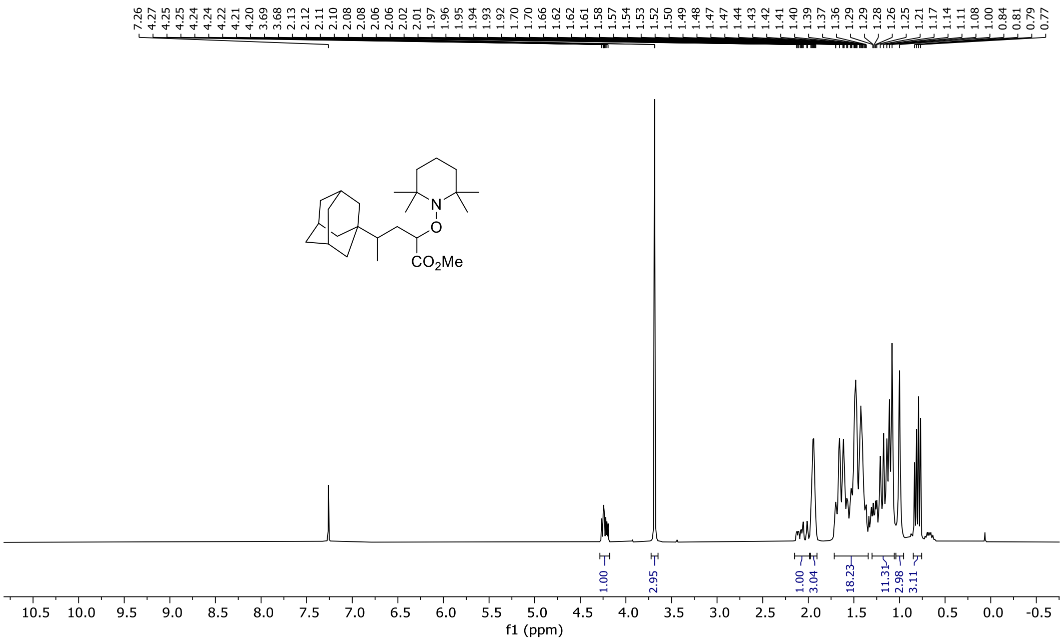

^{13}C NMR (151 MHz, $CDCl_3$)

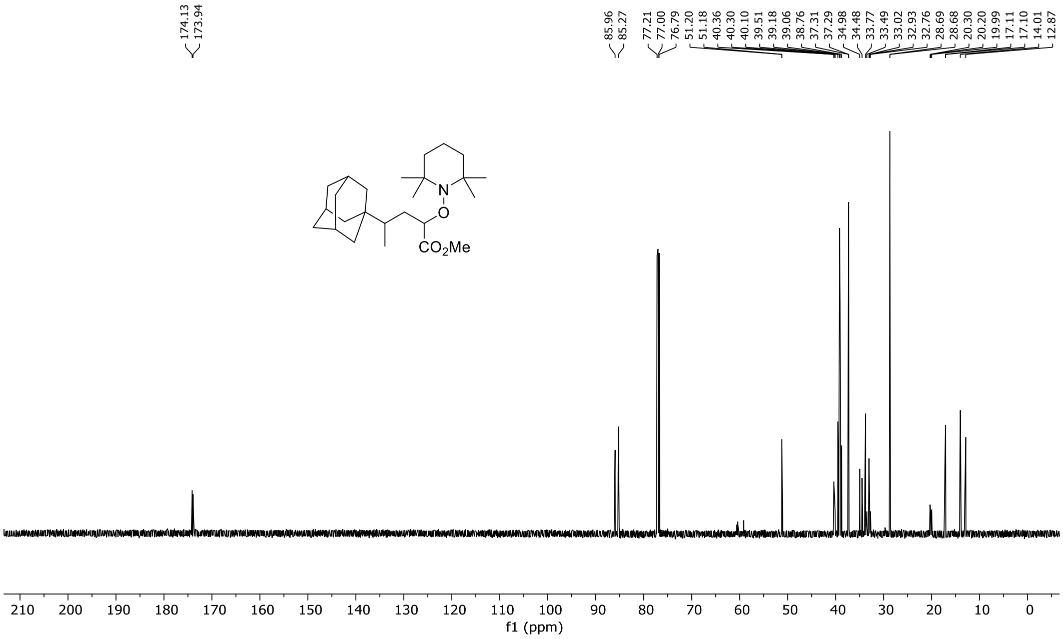

4t

^{1}H NMR (400 MHz, $CDCl_3$)

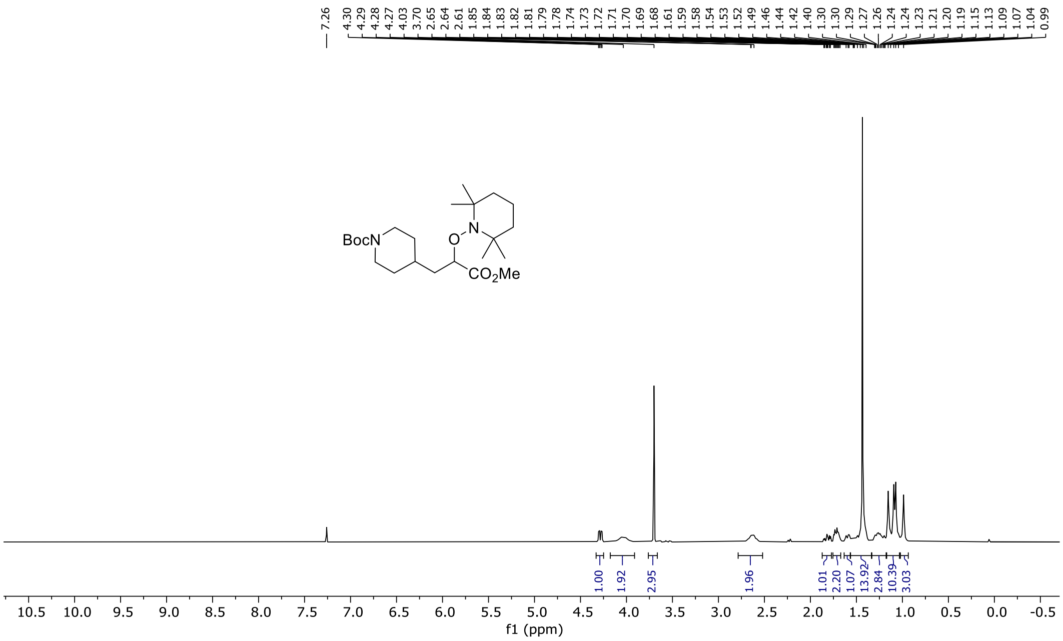

^{13}C NMR (126 MHz, $CDCl_3$)

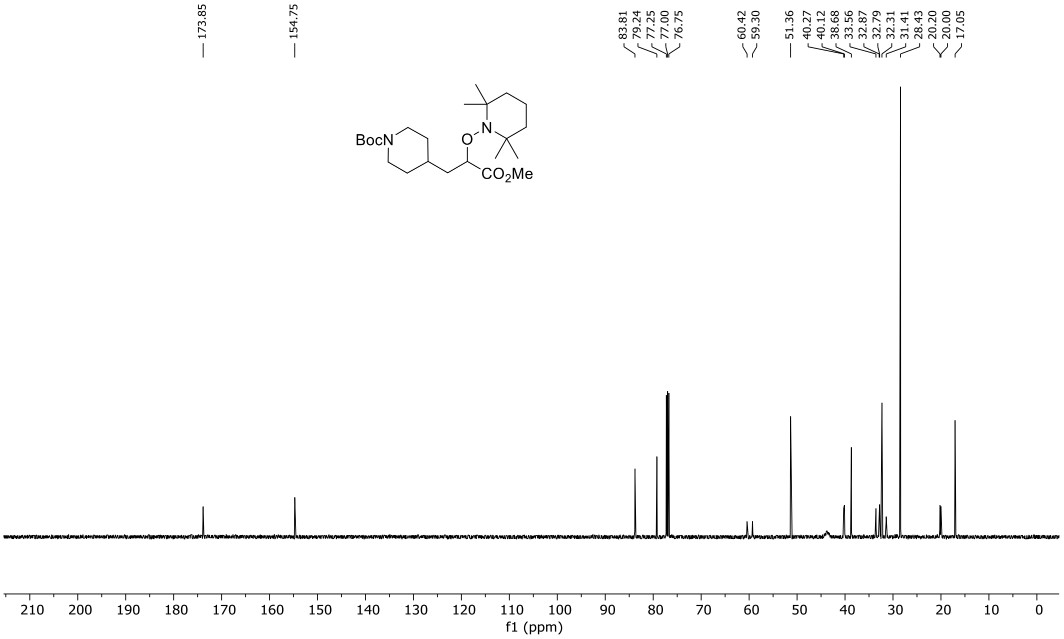

4u

^{1}H NMR (300 MHz, $CDCl_3$)

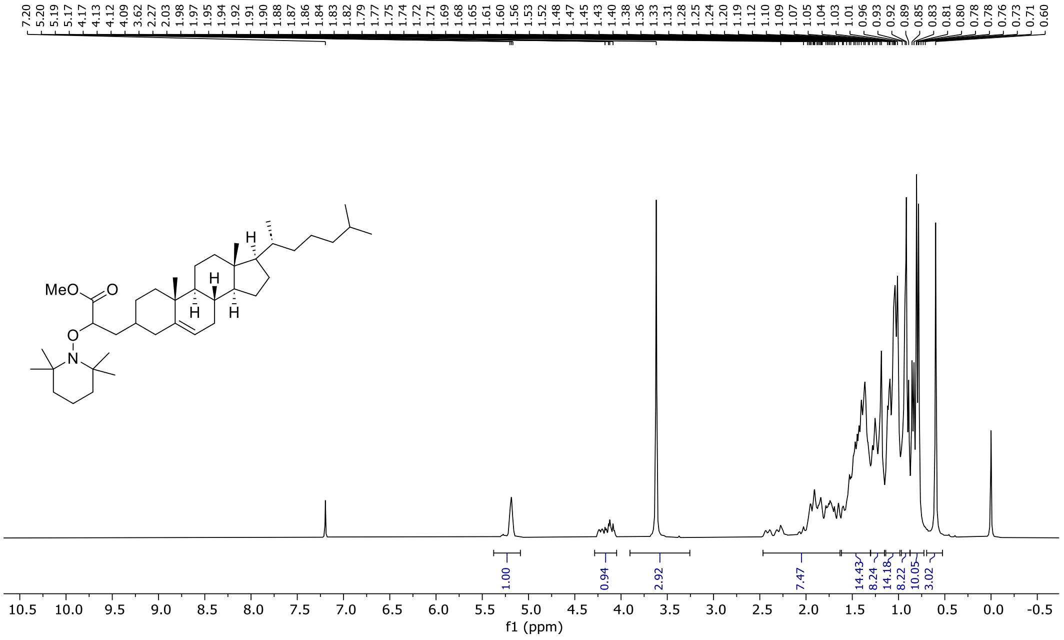

^{13}C NMR (126 MHz, $CDCl_3$)

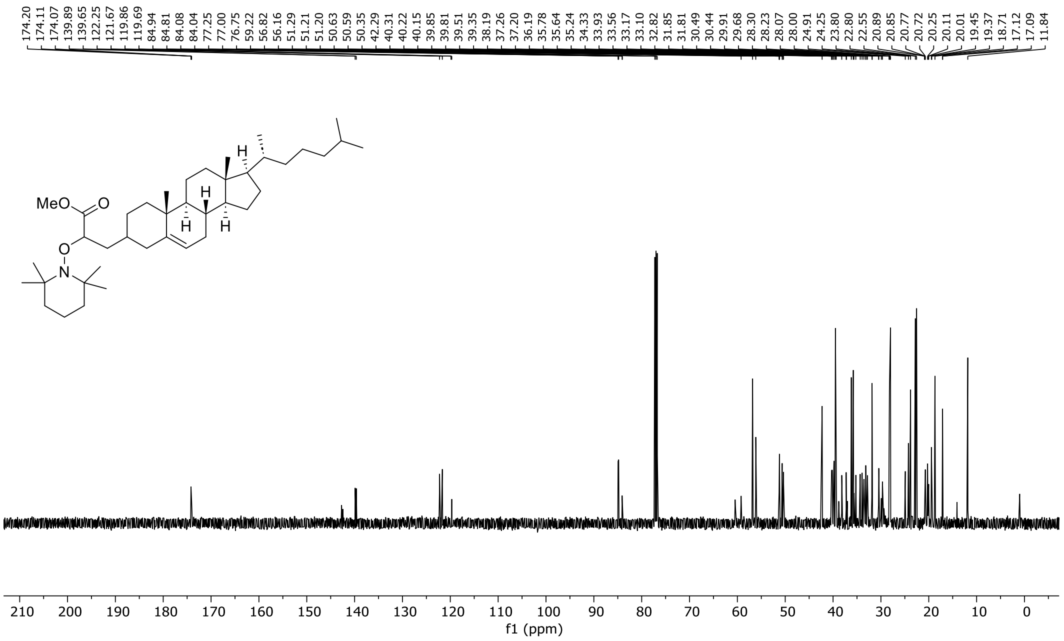

4v

^{1}H NMR (300 MHz, $CDCl_3$)

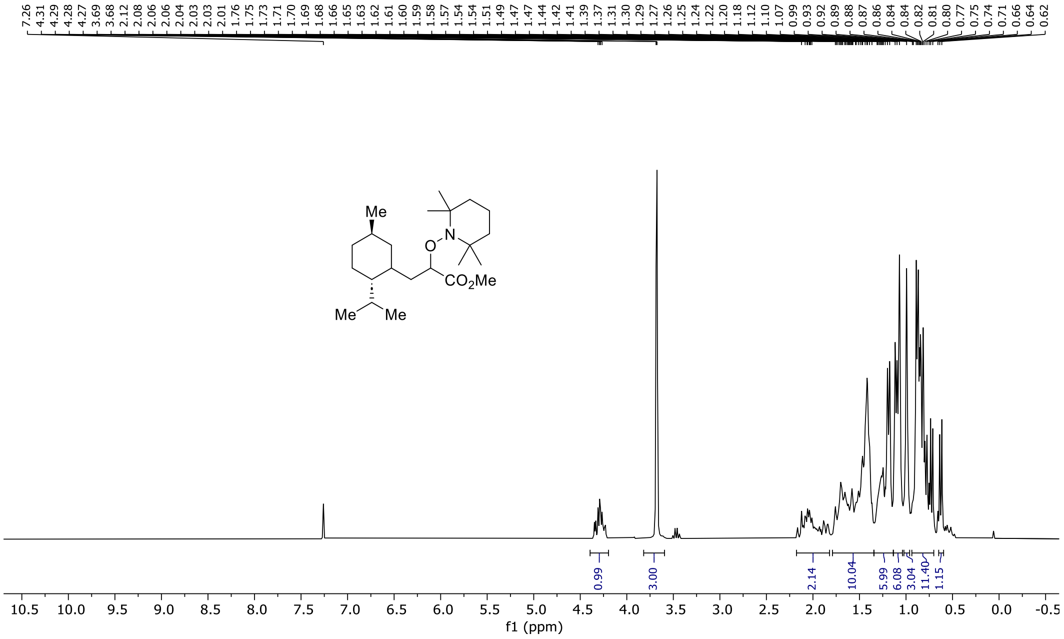

^{13}C NMR (126 MHz, $CDCl_3$)

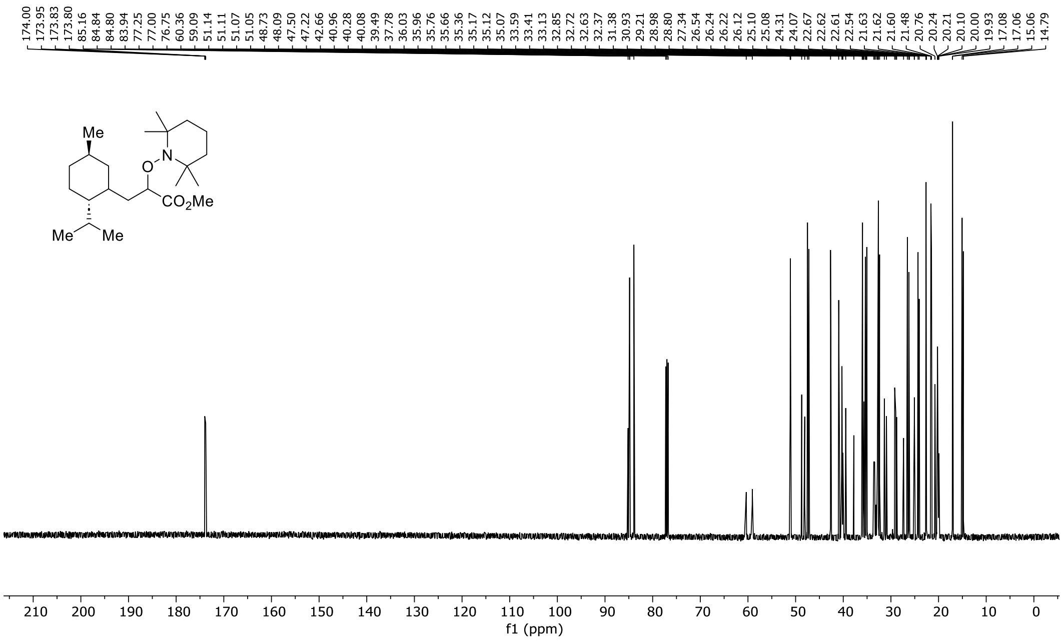

4w

^{1}H NMR (300 MHz, $CDCl_3$)

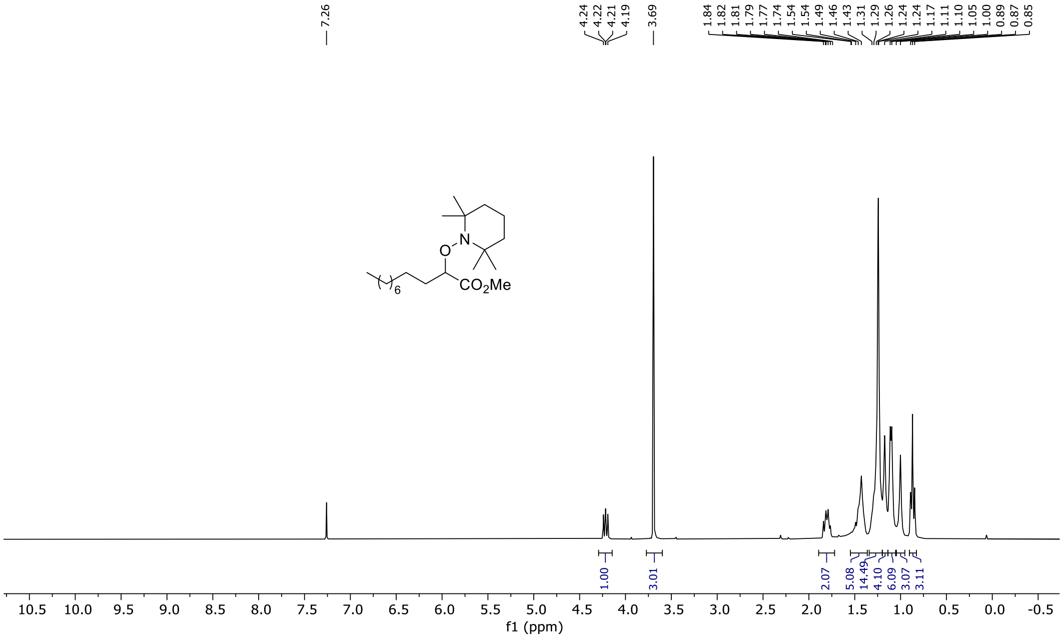

^{13}C NMR (151 MHz, $CDCl_3$)

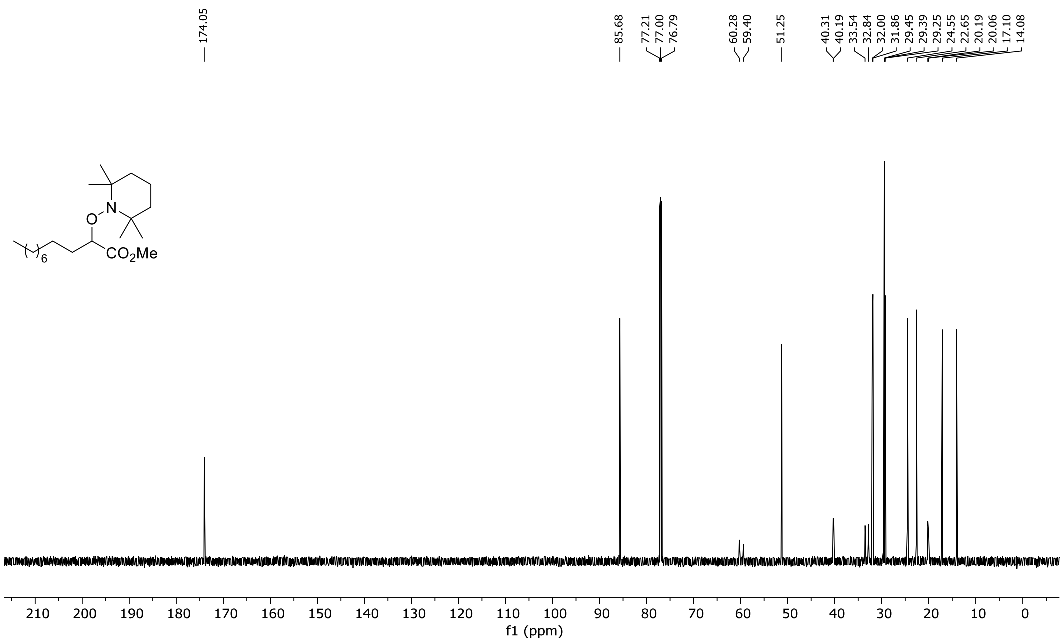

4x

^{1}H NMR (300 MHz, $CDCl_3$)

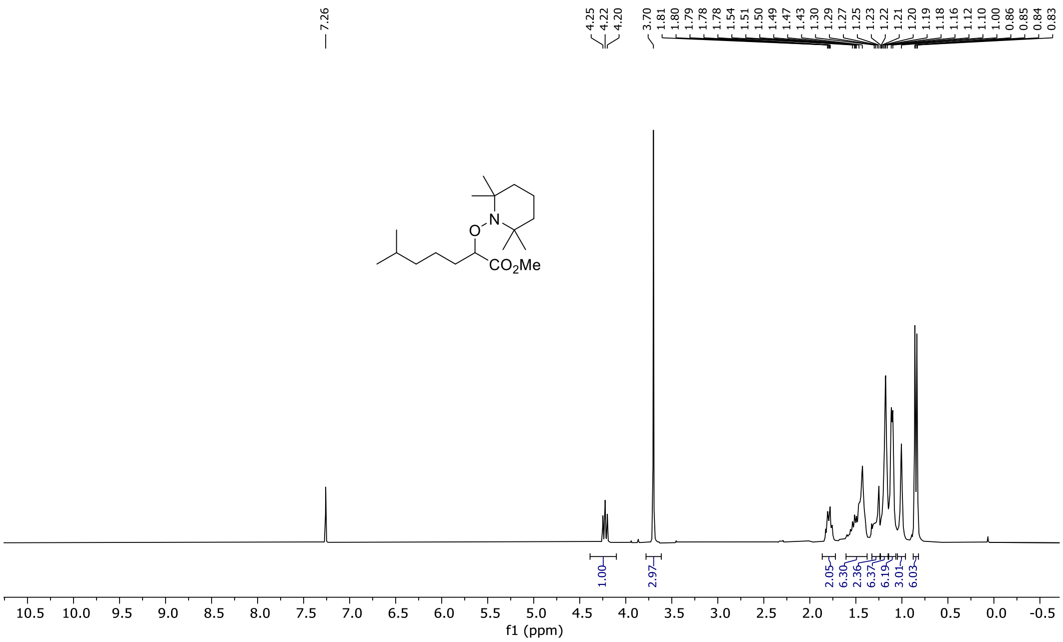

^{13}C NMR (126 MHz, $CDCl_3$)

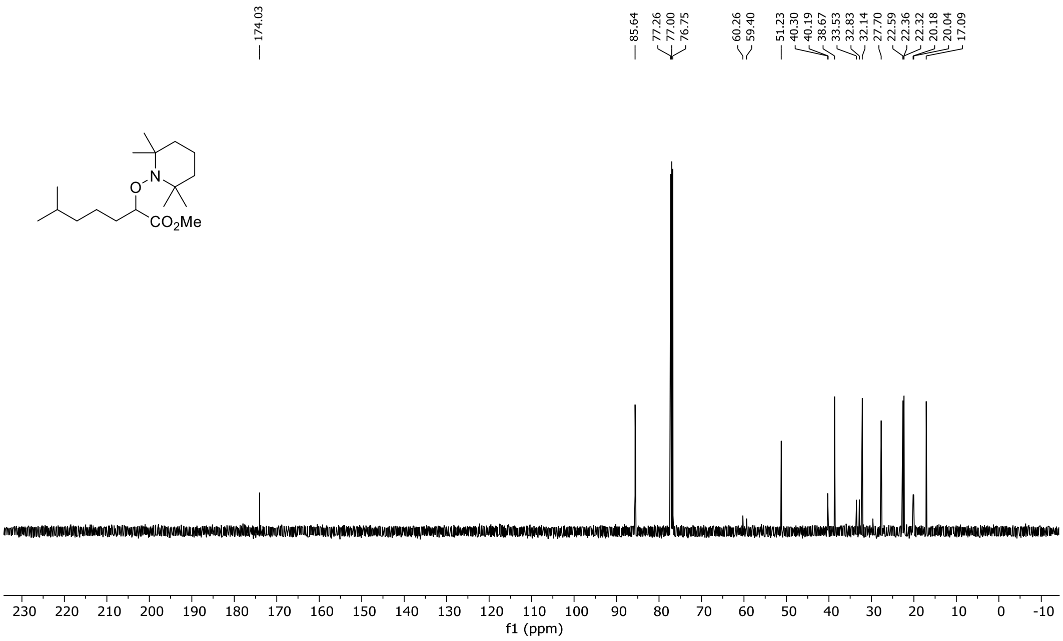

4y

^{1}H NMR (600 MHz, $CDCl_3$)

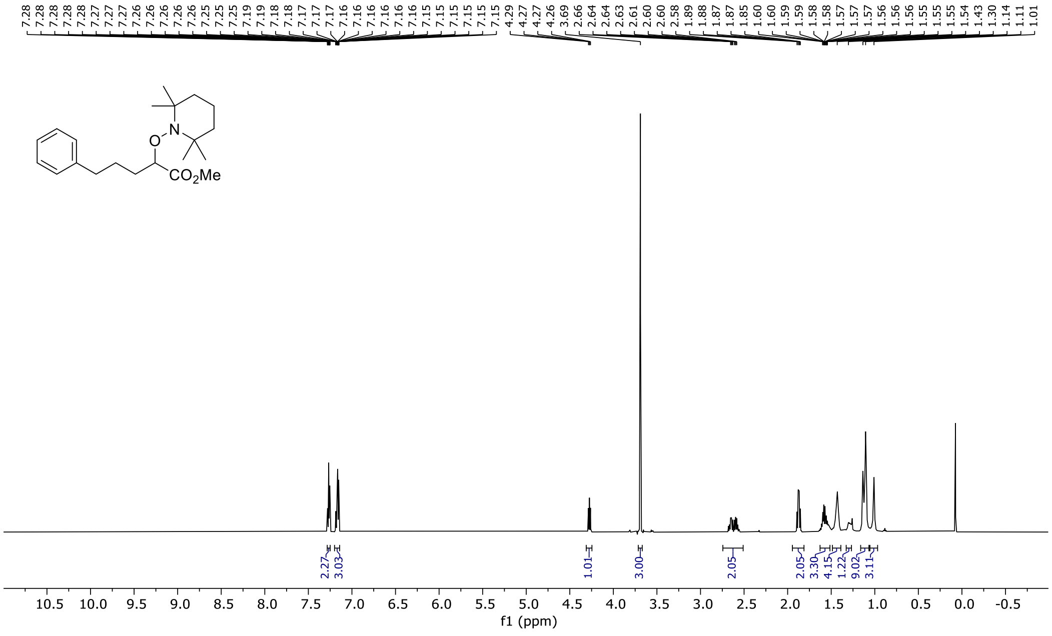

¹³C NMR (151 MHz, $CDCl_3$)

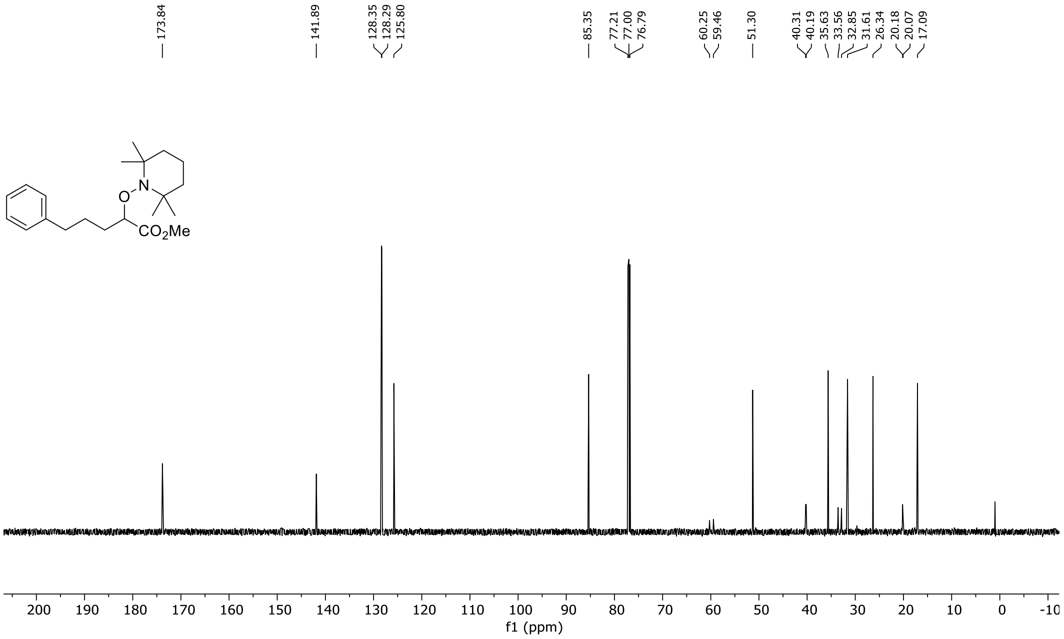

6a

¹H NMR (500 MHz, $CDCl_3$)

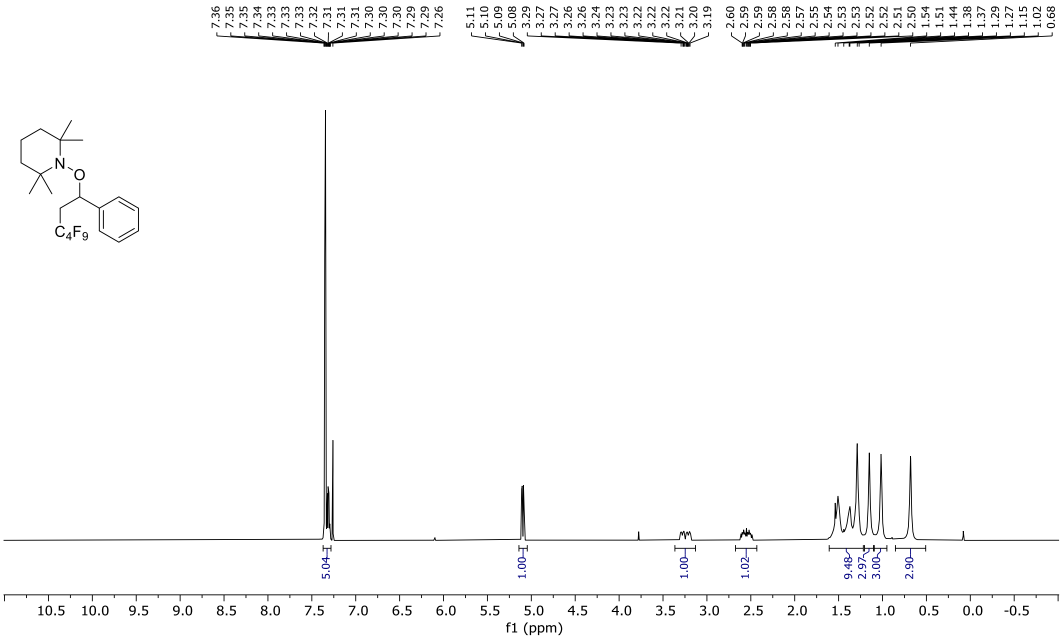

$^{13}C\{^{19}F\}$ NMR (126 MHz, $CDCl_3$)

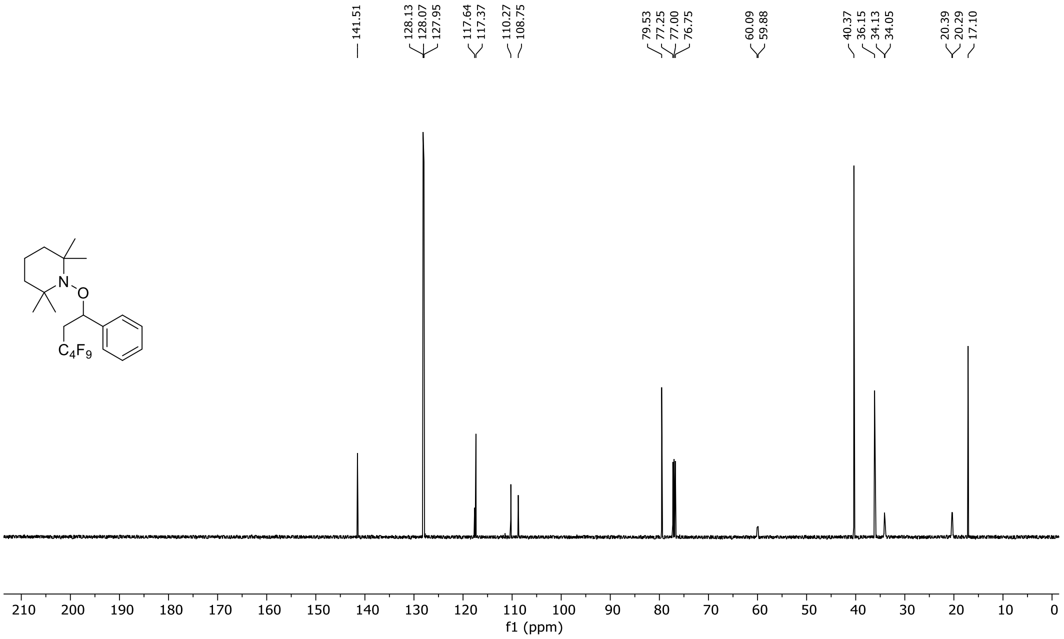

^{19}F NMR (470 MHz, $CDCl_3$)

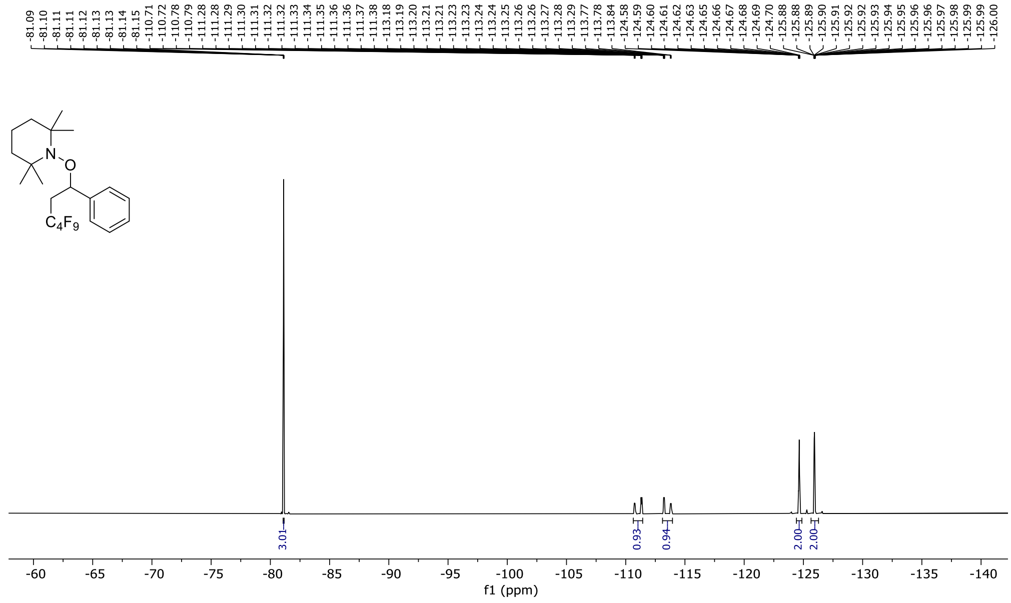

6b

^{1}H NMR (300 MHz, $CDCl_3$)

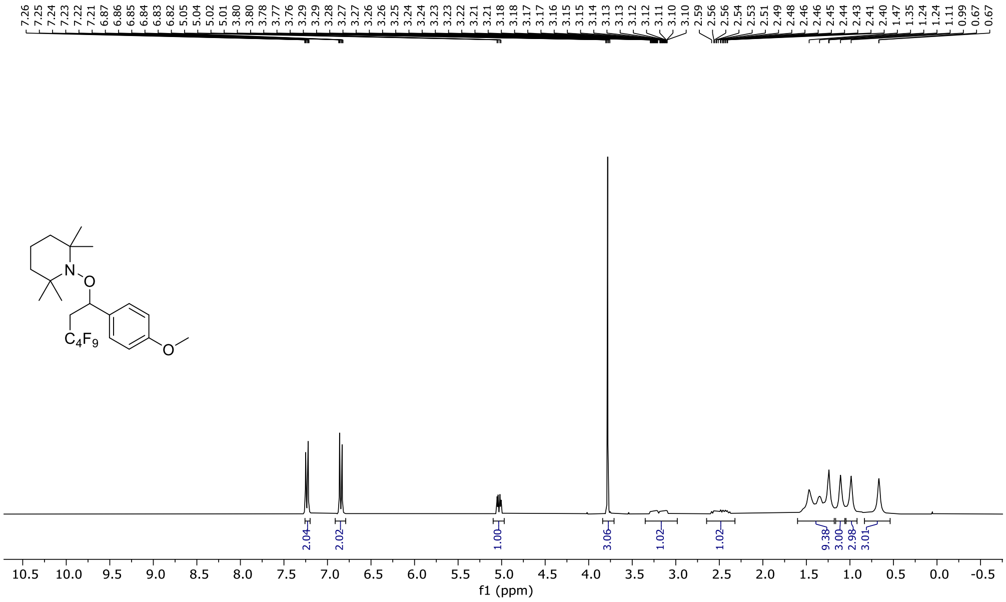

^{13}C{^{19}F} NMR (151 MHz, $CDCl_3$)

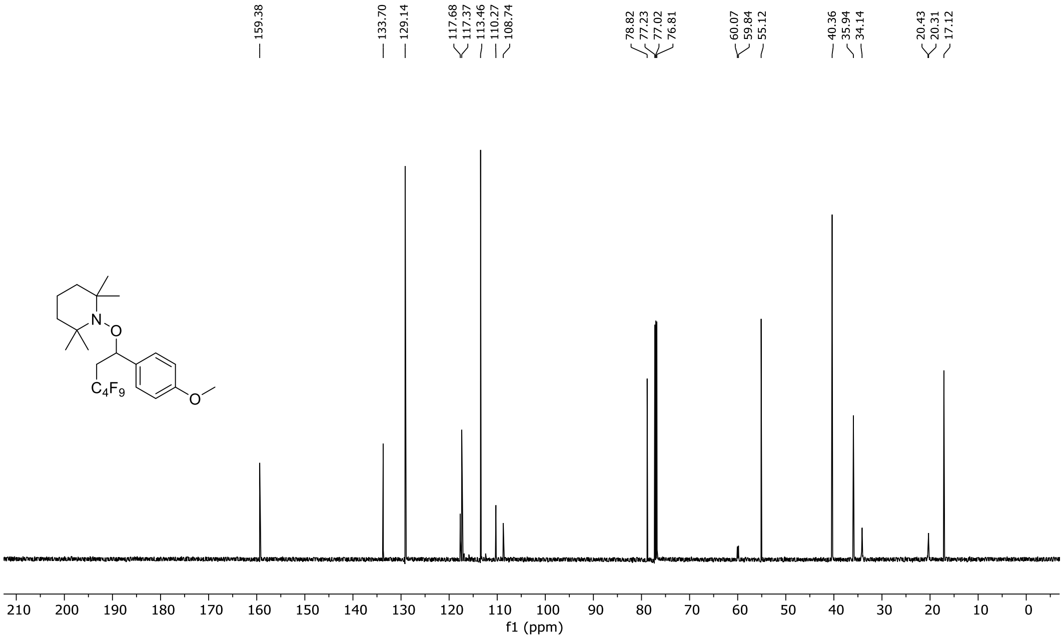

^{19}F NMR (564 MHz, $CDCl_3$)

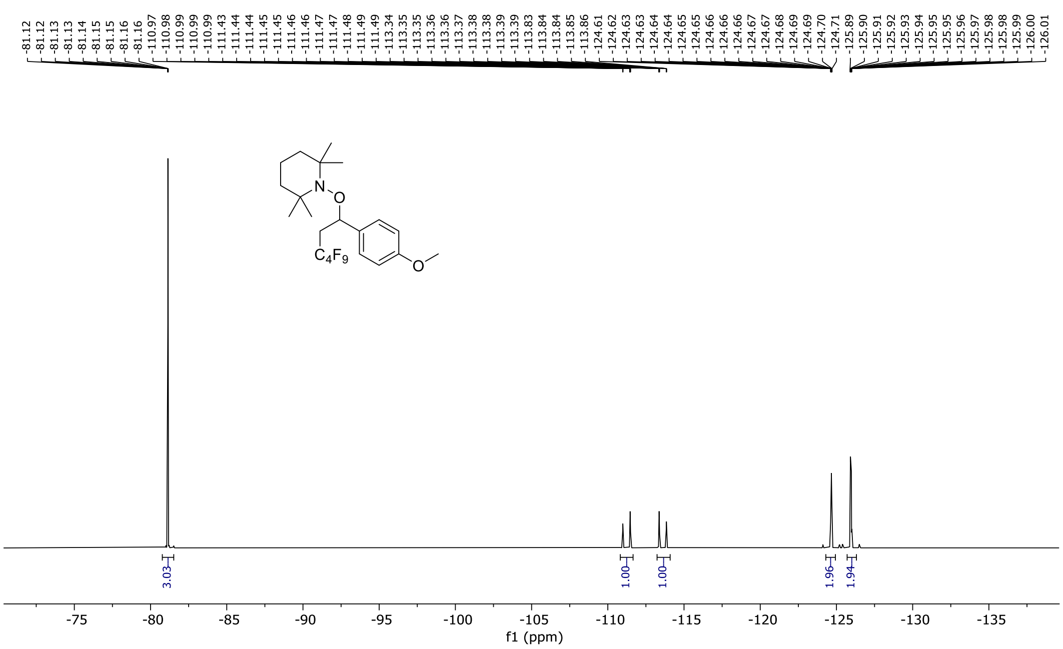

6c

^{1}H NMR (300 MHz, $CDCl_3$)

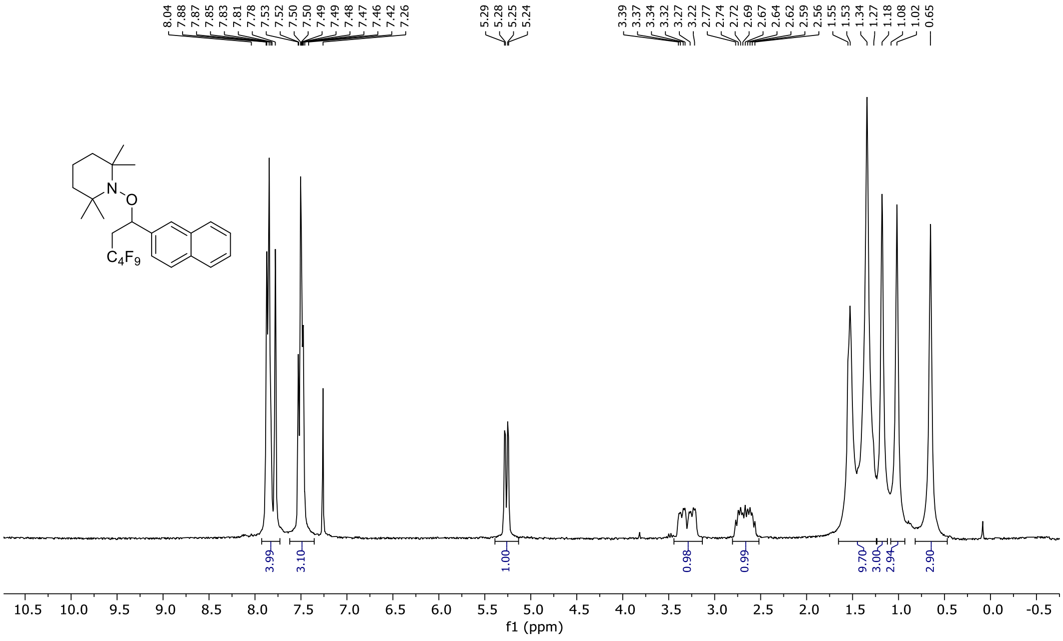

$^{13}C\{^{19}F\}$ NMR (126 MHz, $CDCl_3$)

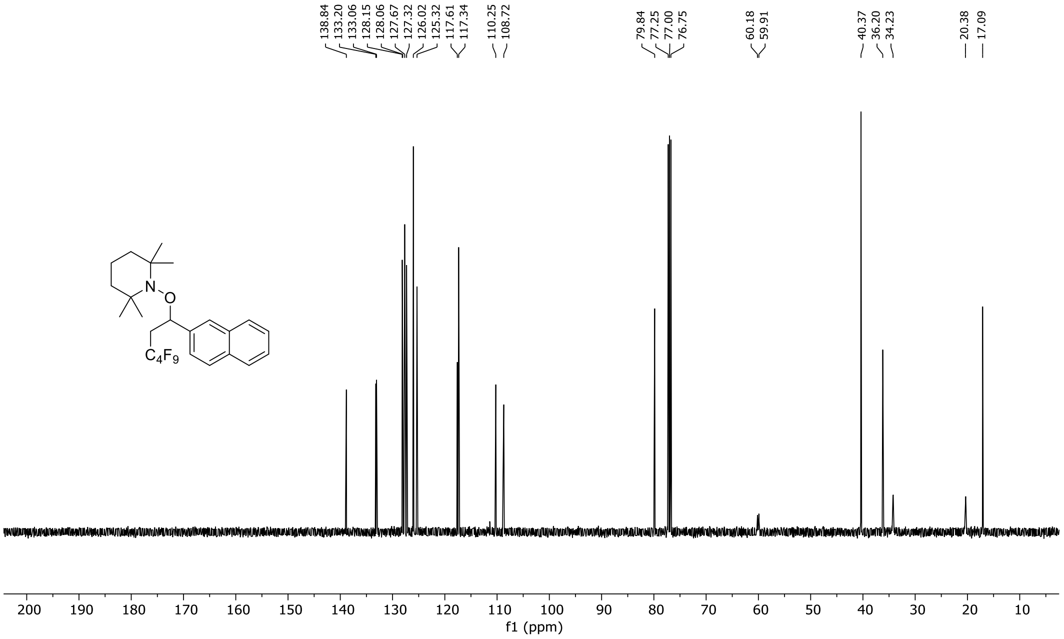

^{19}F NMR (470 MHz, $CDCl_3$)

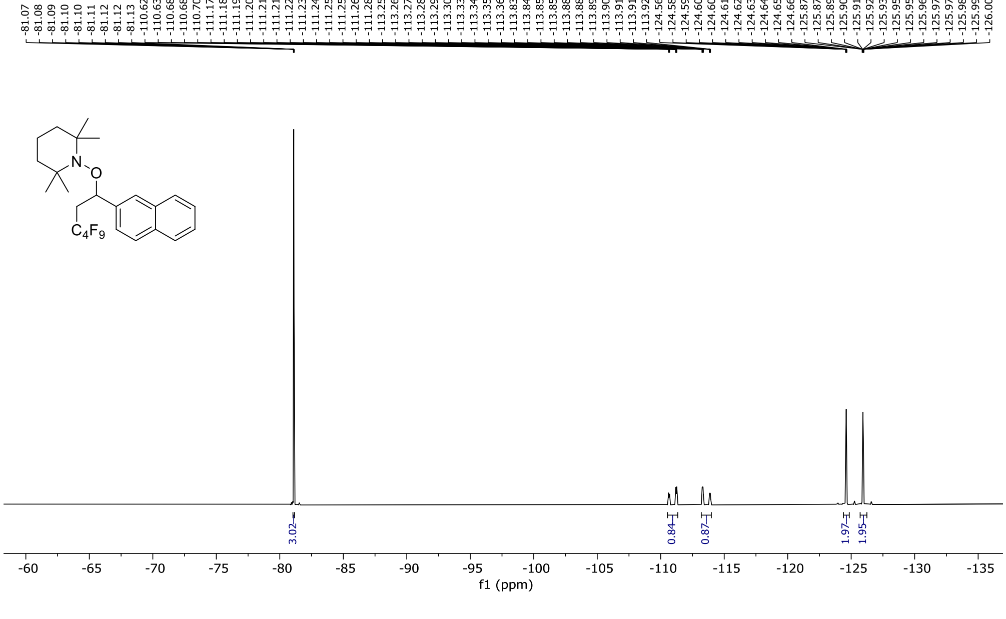

6d

^{1}H NMR (600 MHz, $CDCl_3$)

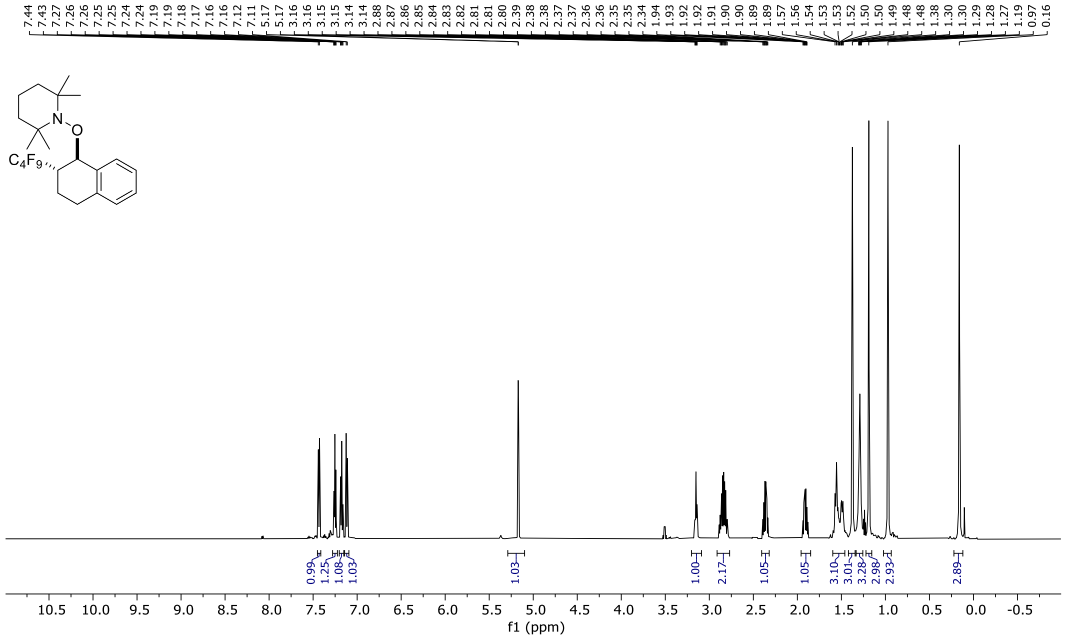

^{13}C{^{19}F} NMR (151 MHz, $CDCl_3$)

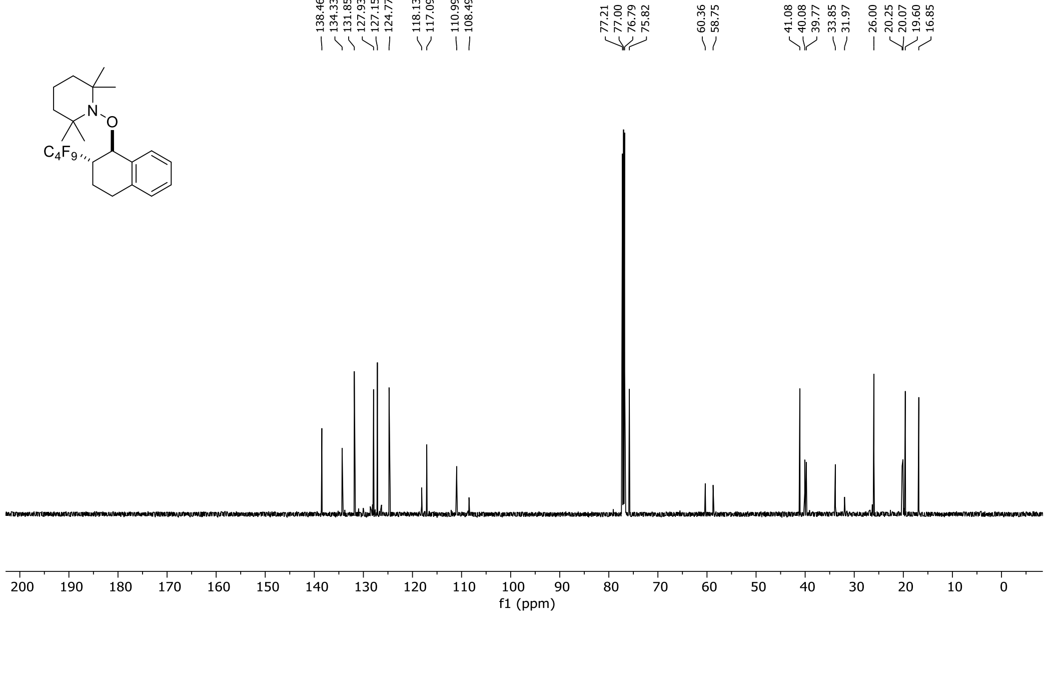

^{19}F NMR (564 MHz, $CDCl_3$)

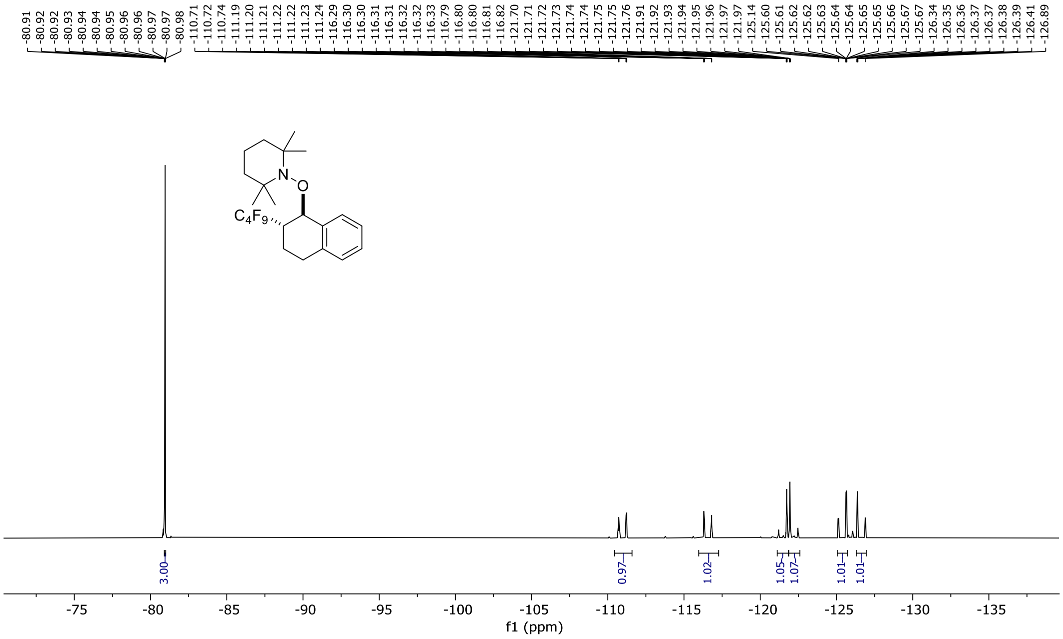

6e

^{1}H NMR (500 MHz, $CDCl_3$)

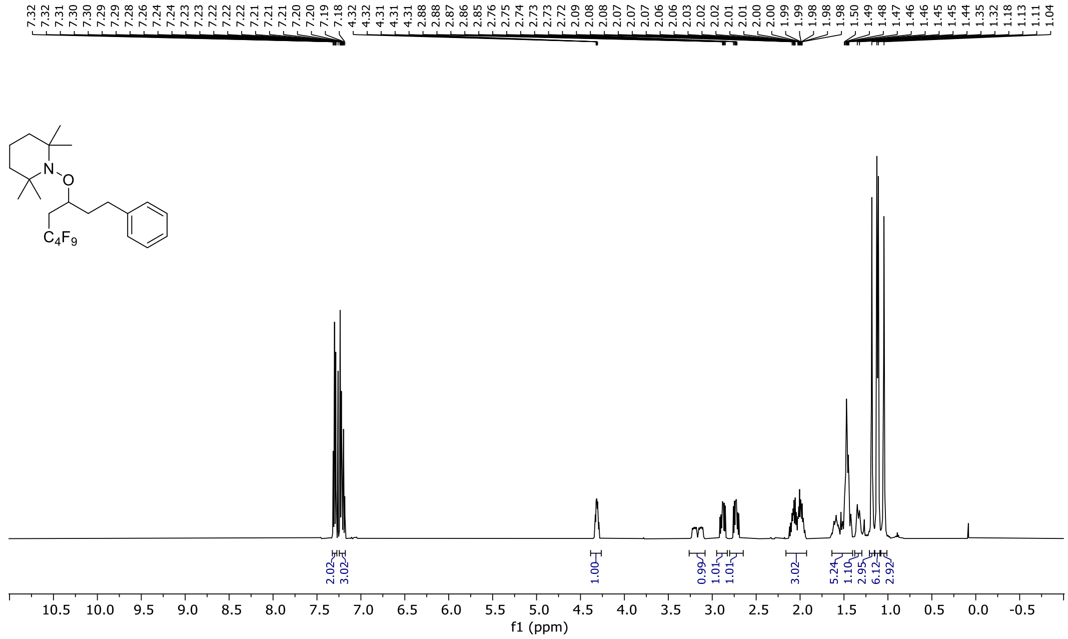

$^{13}C\{^{19}F\}$ NMR (126 MHz, $CDCl_3$)

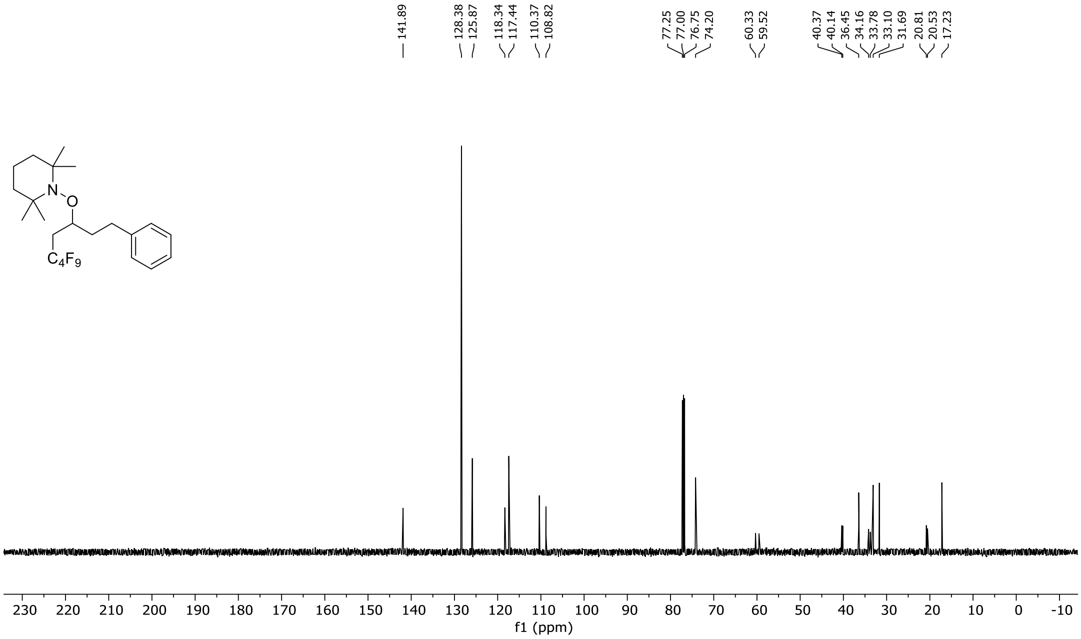

^{19}F NMR (470 MHz, $CDCl_3$)

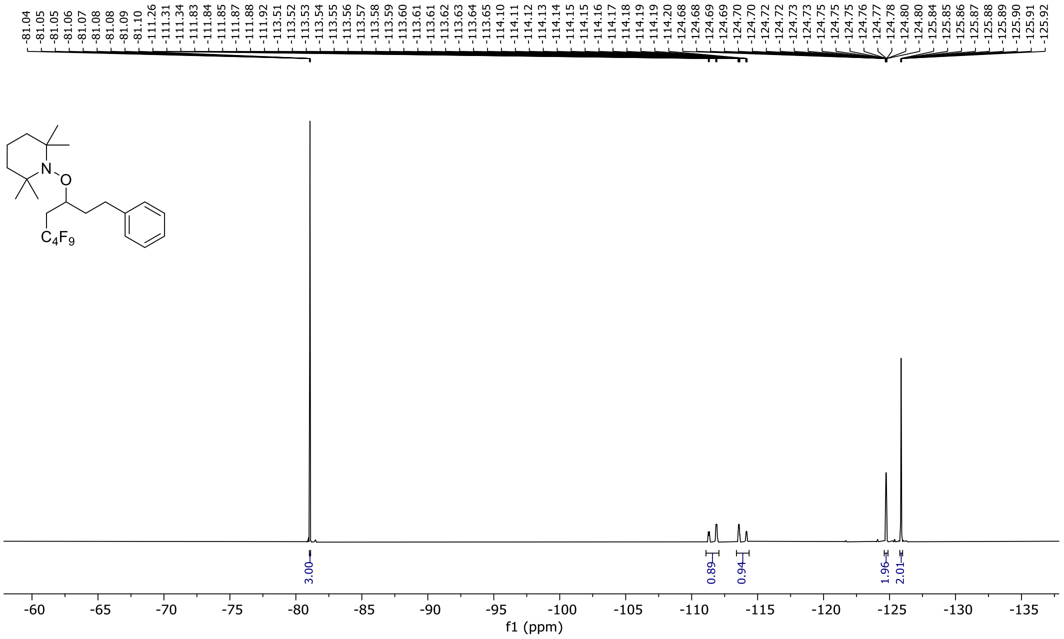

6f

^{1}H NMR (500 MHz, CDCl$_3$)

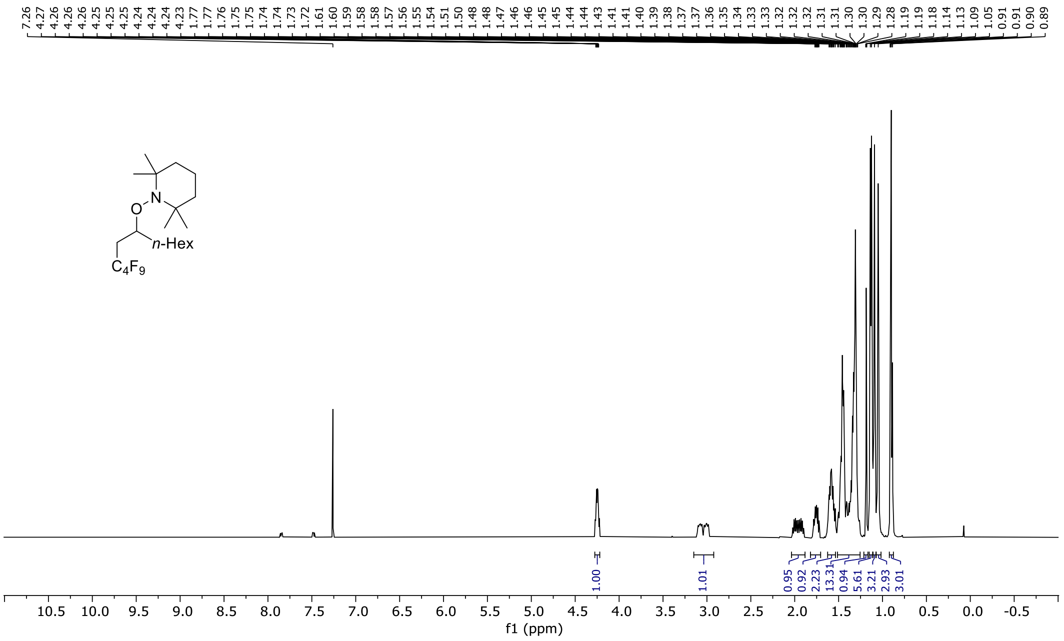

^{13}C{^{19}F} NMR (126 MHz, CDCl$_3$)

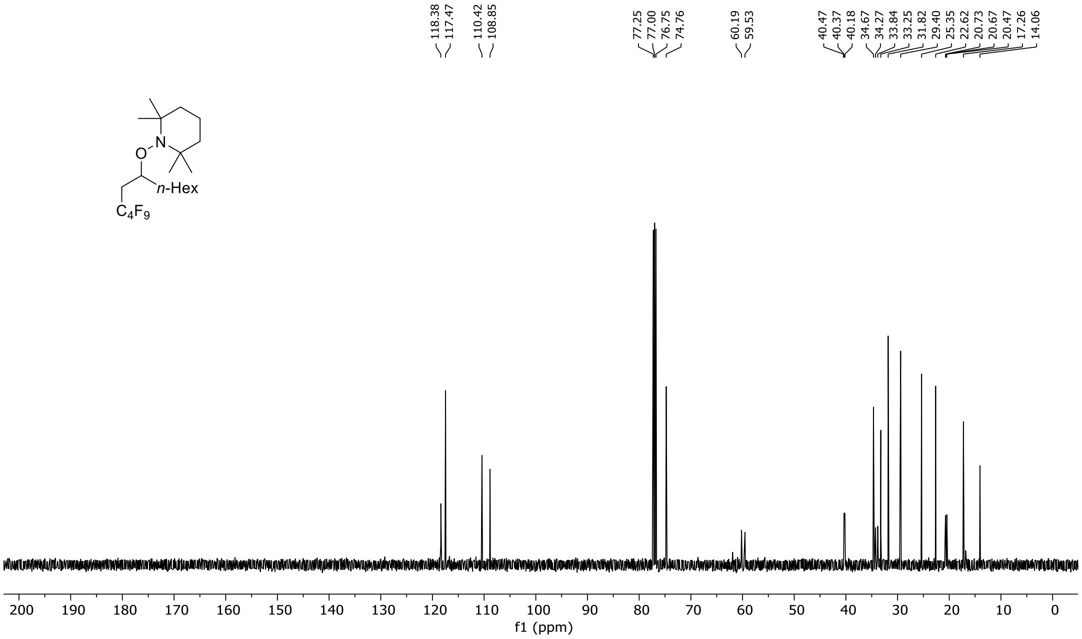

^{19}F NMR (470 MHz, $CDCl_3$)

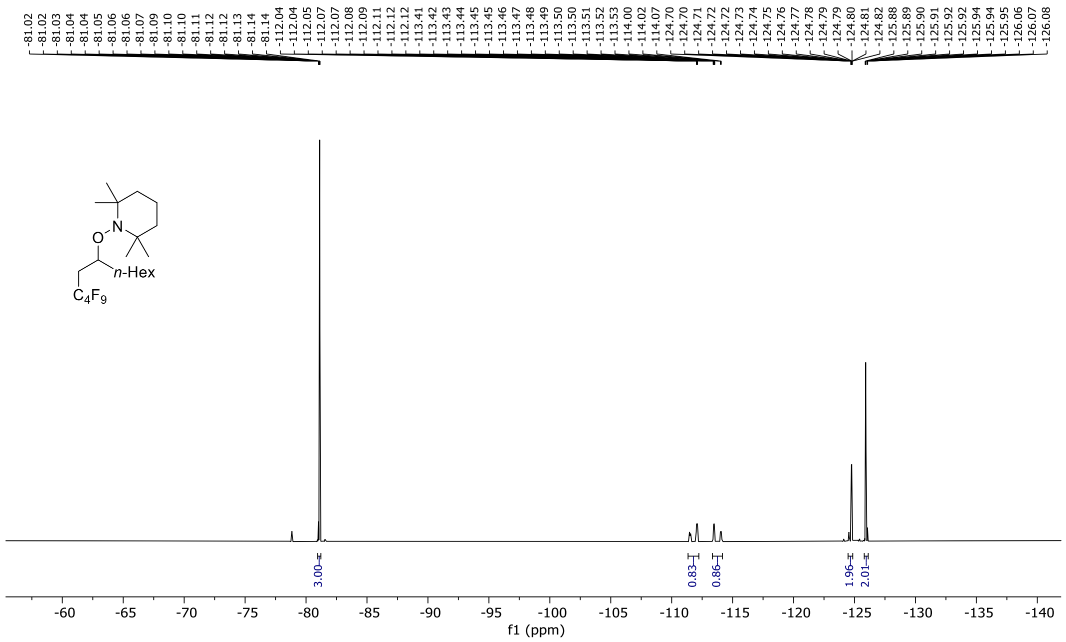

6g

^{1}H NMR (500 MHz, $CDCl_3$)

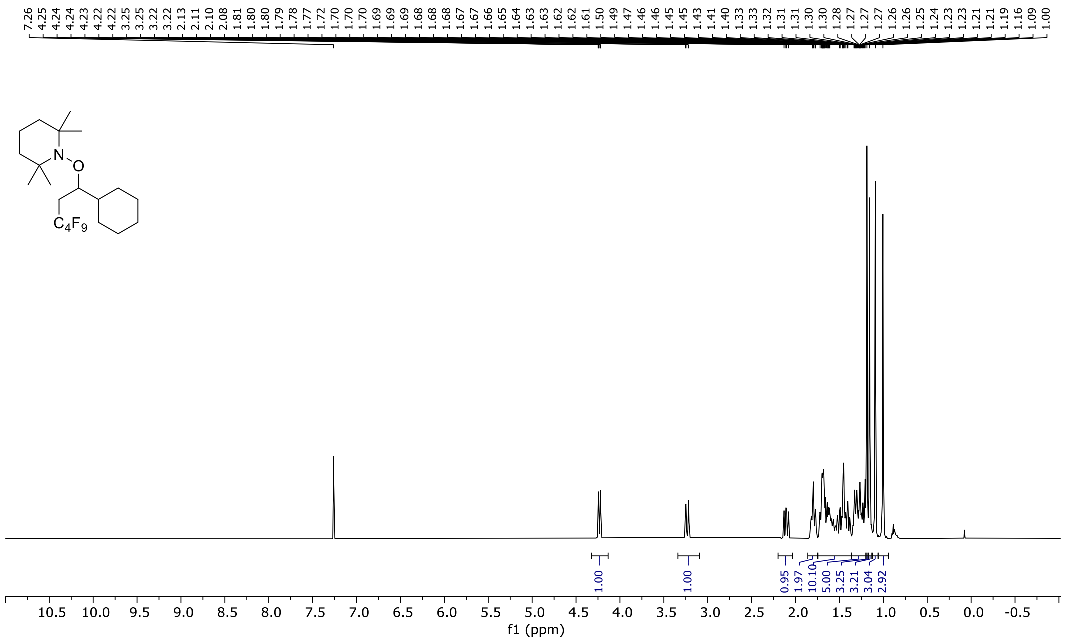

$^{13}C\{^{19}F\}$ NMR (126 MHz, $CDCl_3$)

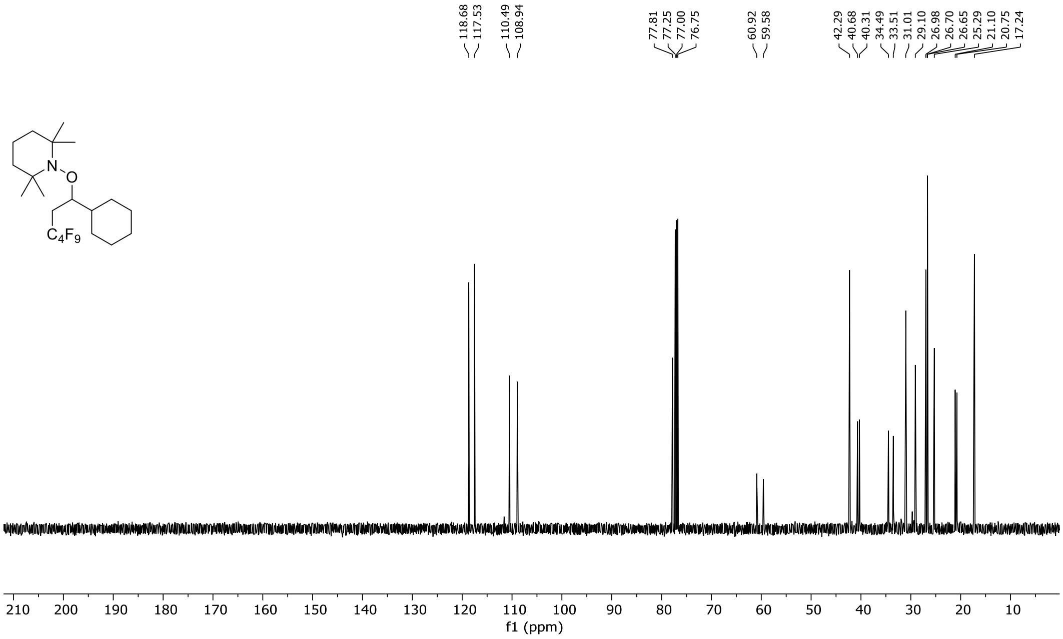

^{19}F NMR (470 MHz, $CDCl_3$)

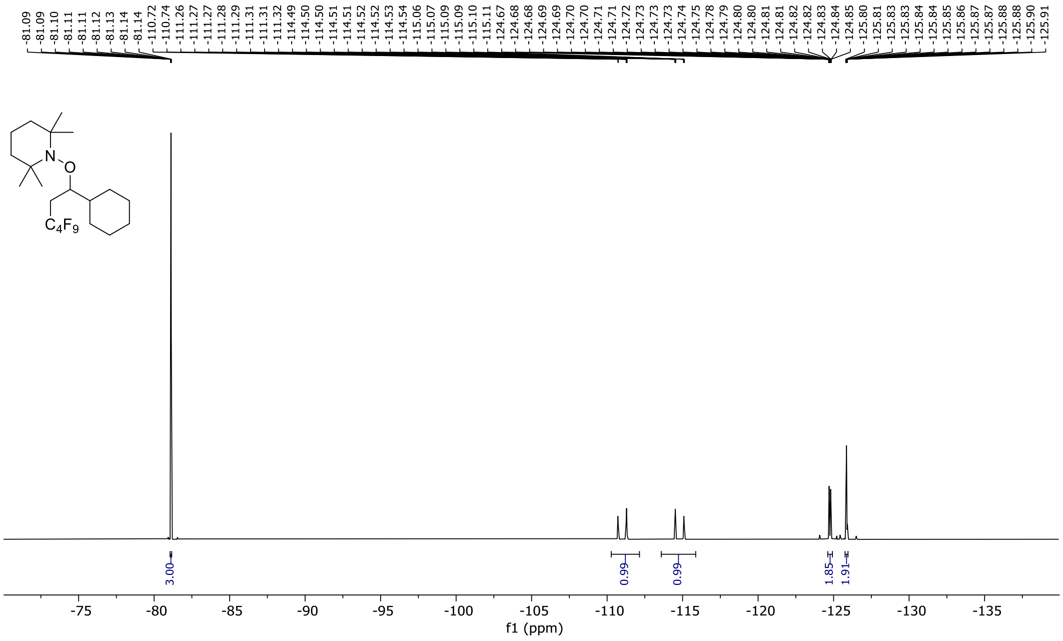

6h

^{1}H NMR (500 MHz, $CDCl_3$)

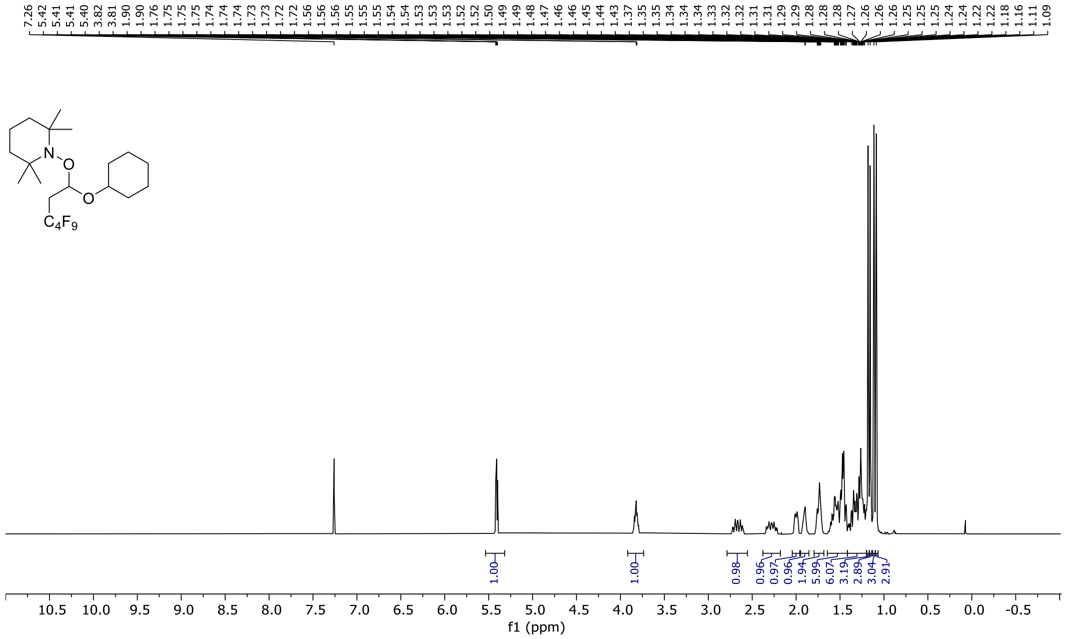

^{13}C{^{19}F} NMR (126 MHz, $CDCl_3$)

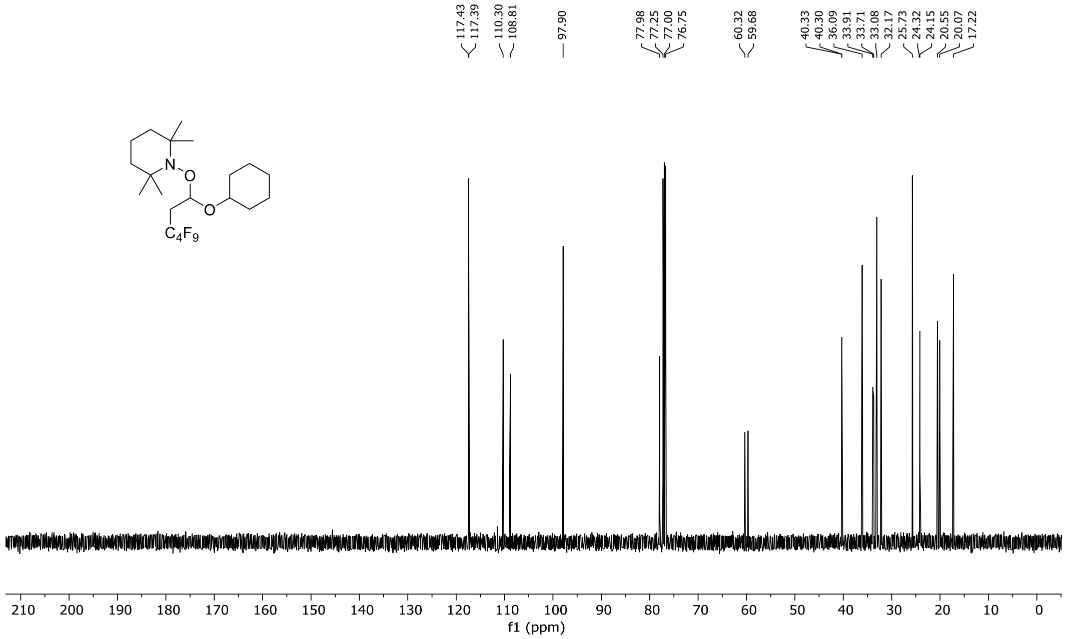

^{19}F NMR (470 MHz, $CDCl_3$)

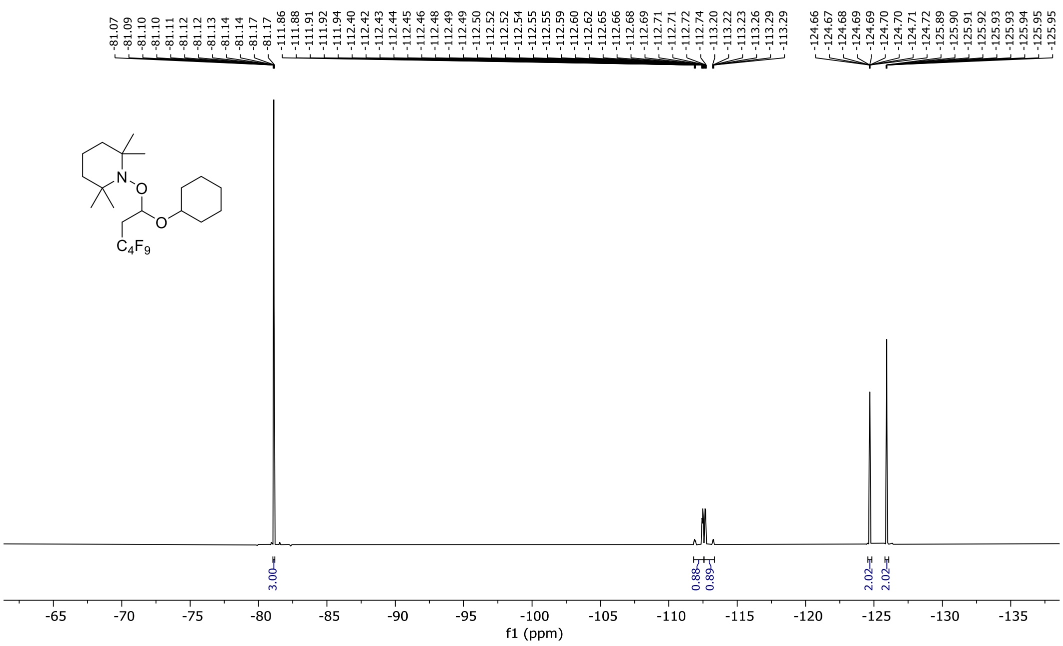

6i

^{1}H NMR (300 MHz, $CDCl_3$)

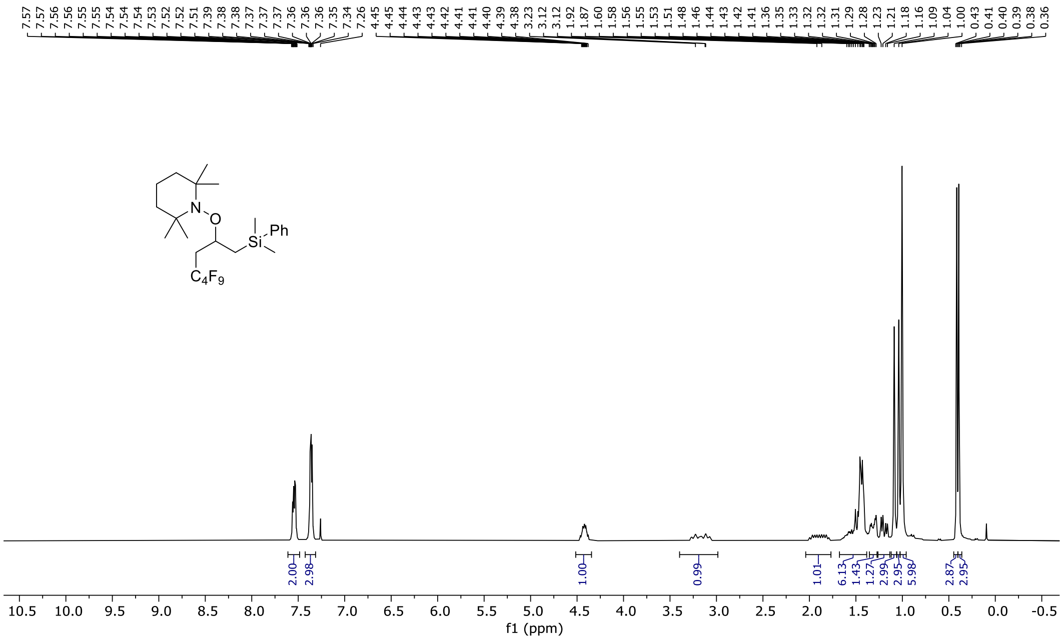

$^{13}C\{^{19}F\}$ NMR (126 MHz, $CDCl_3$)

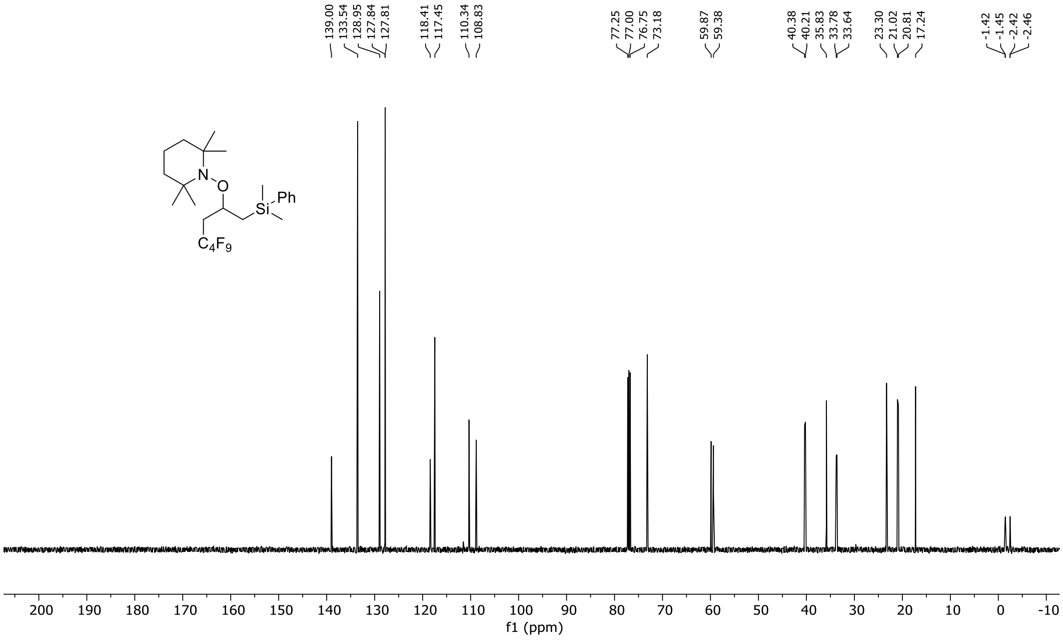

^{19}F NMR (470 MHz, $CDCl_3$)

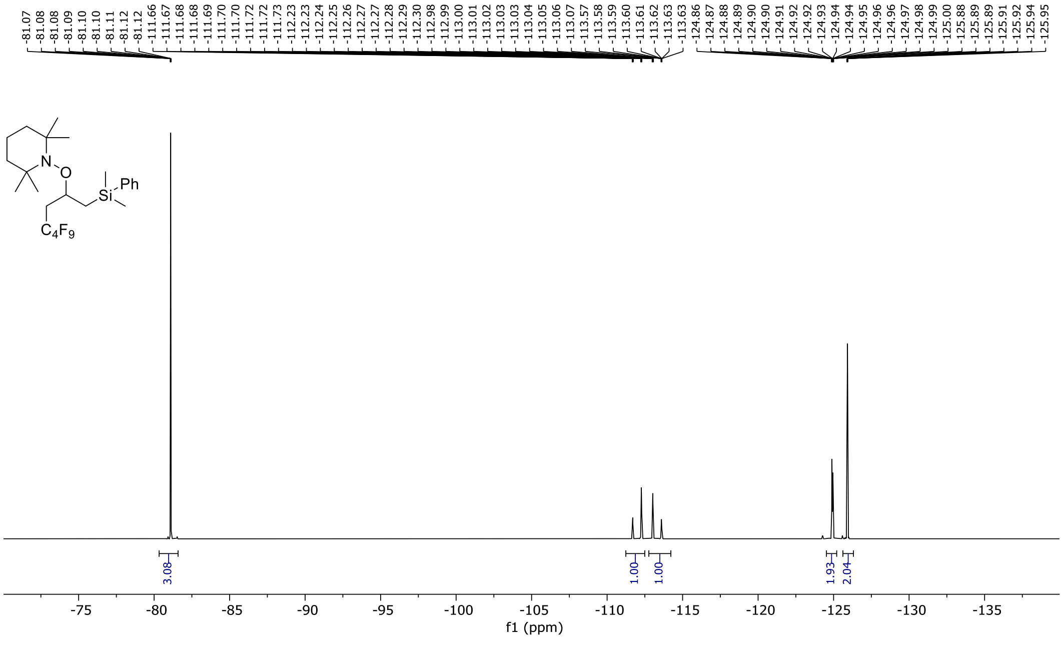

6j

^{1}H NMR (500 MHz, $CDCl_3$)

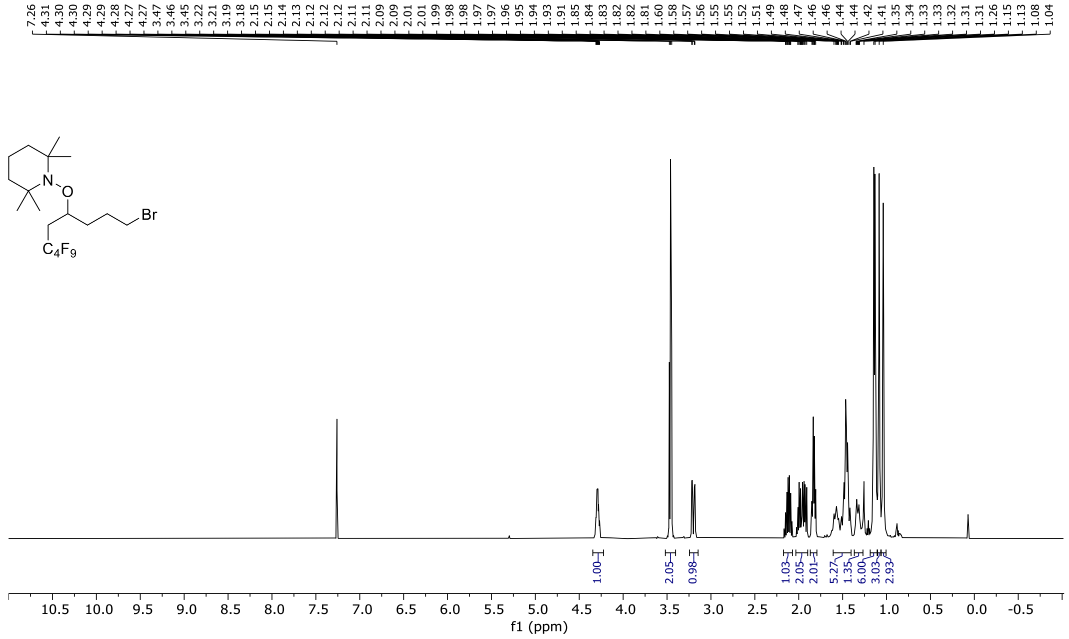

^{13}C{^{19}F} NMR (126 MHz, $CDCl_3$)

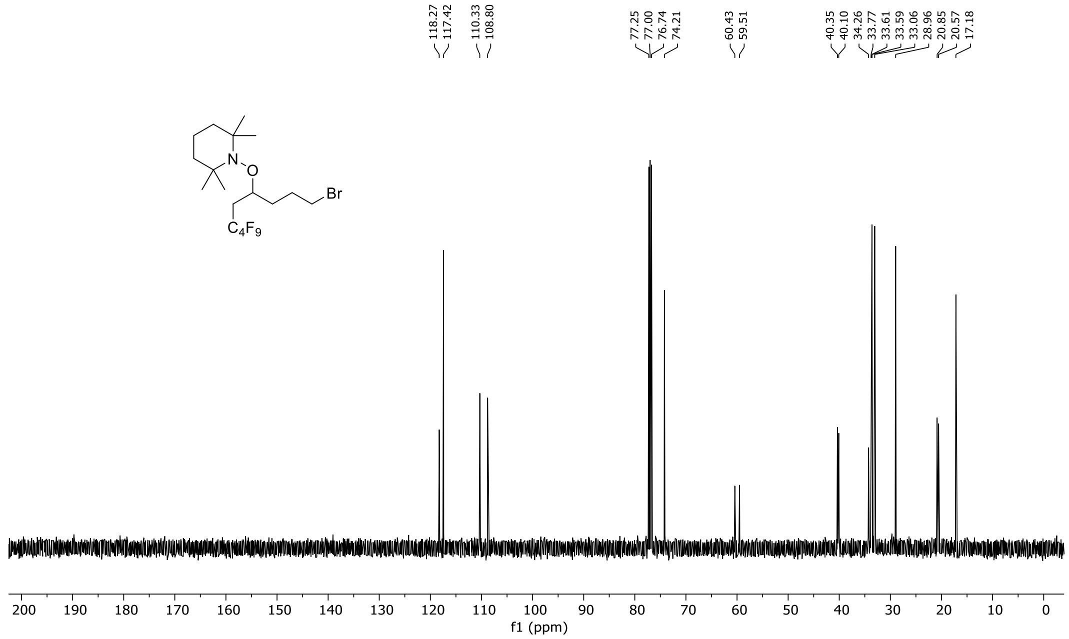

^{19}F **NMR** (470 MHz, $CDCl_3$)

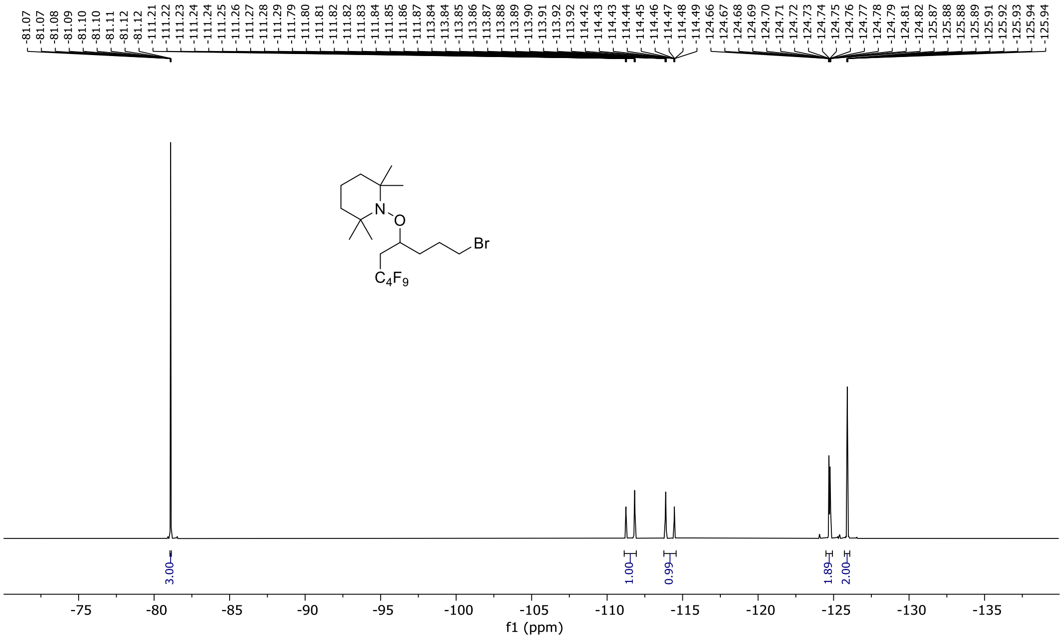

6k

^{1}H **NMR** (500 MHz, $CDCl_3$)

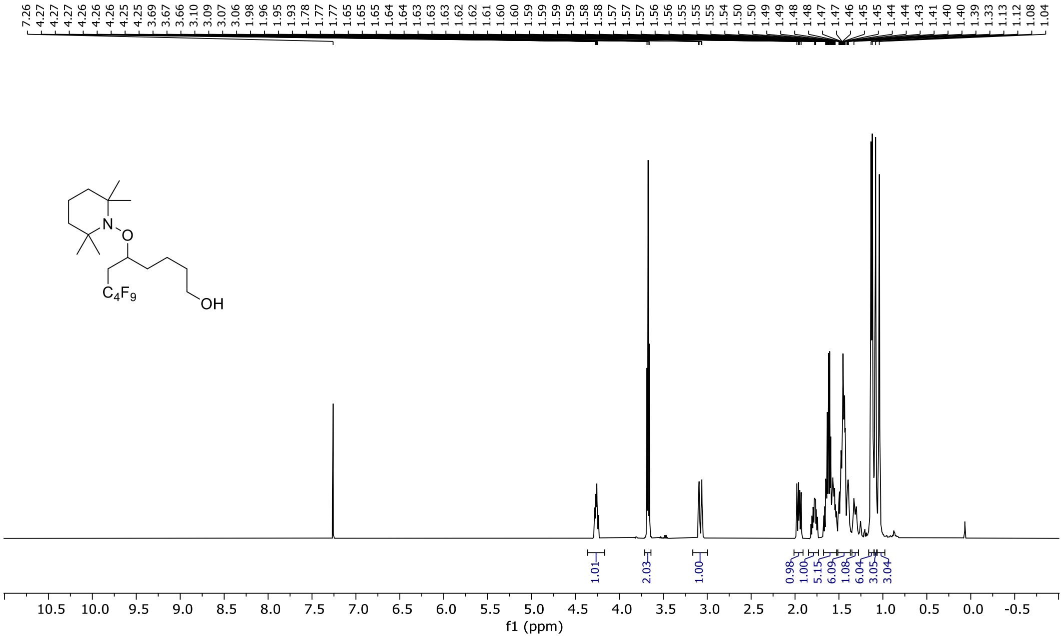

$^{13}C\{^{19}F\}$ NMR (126 MHz, $CDCl_3$)

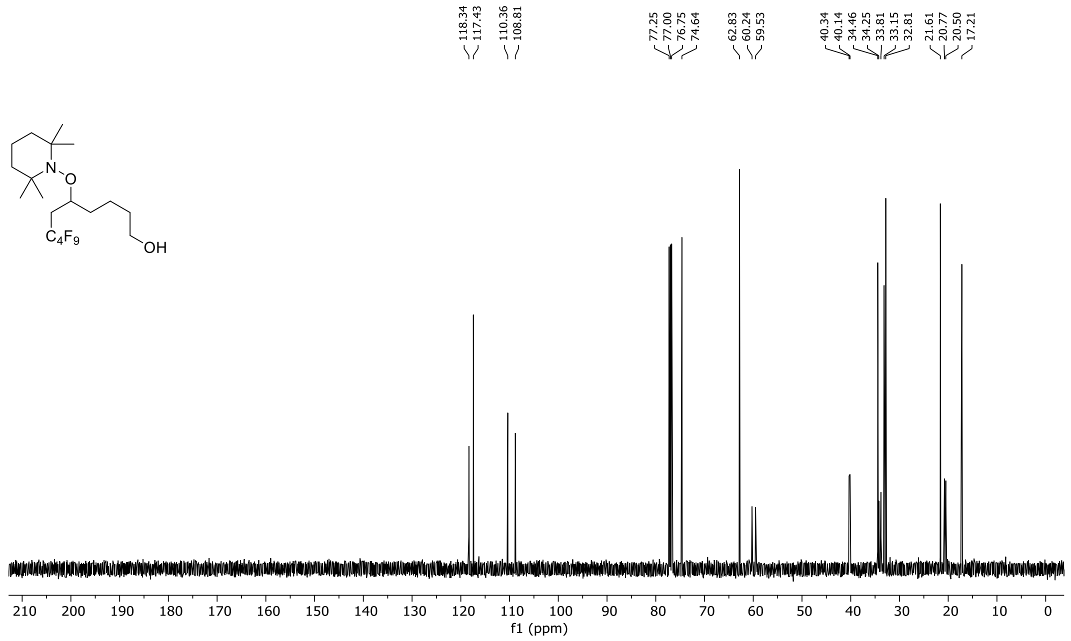

^{19}F NMR (470 MHz, $CDCl_3$)

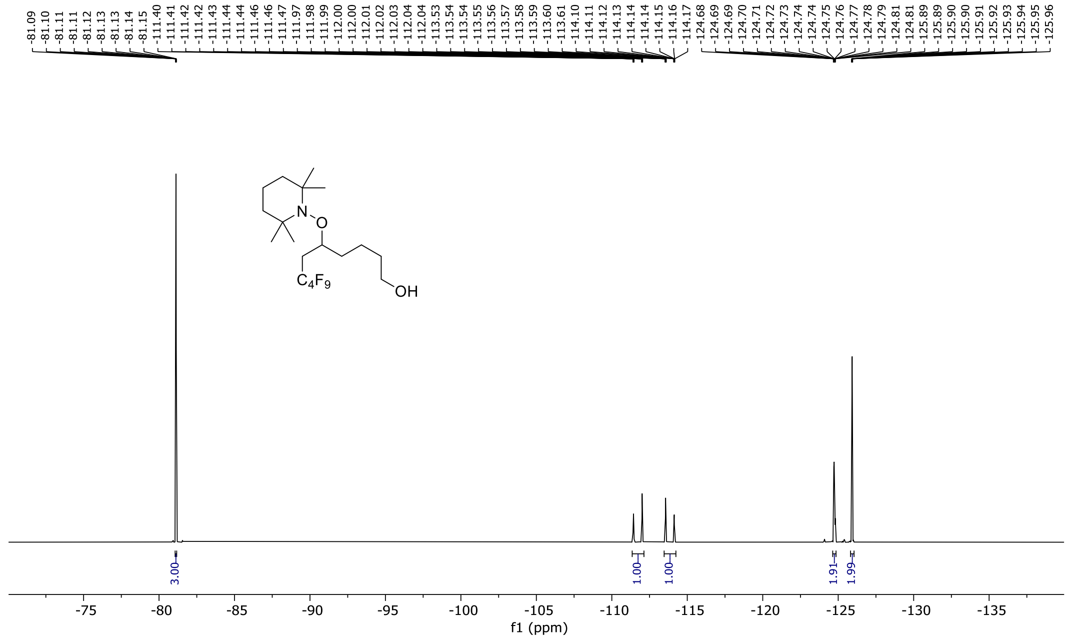

6l

^{1}H NMR (300 MHz, $CDCl_3$)

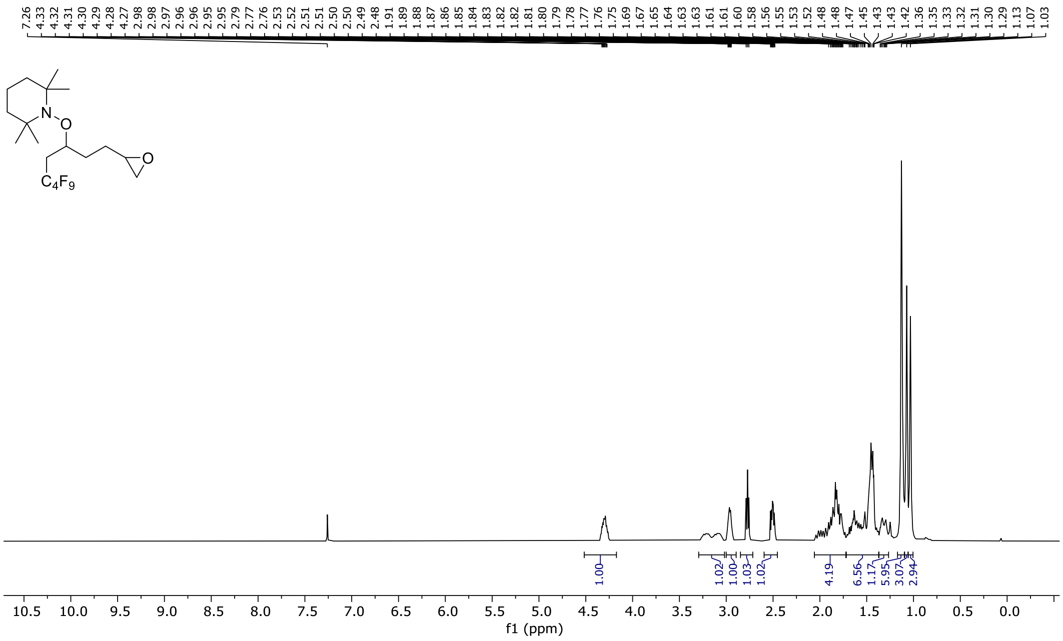

^{13}C{^{19}F} NMR (126 MHz, $CDCl_3$)

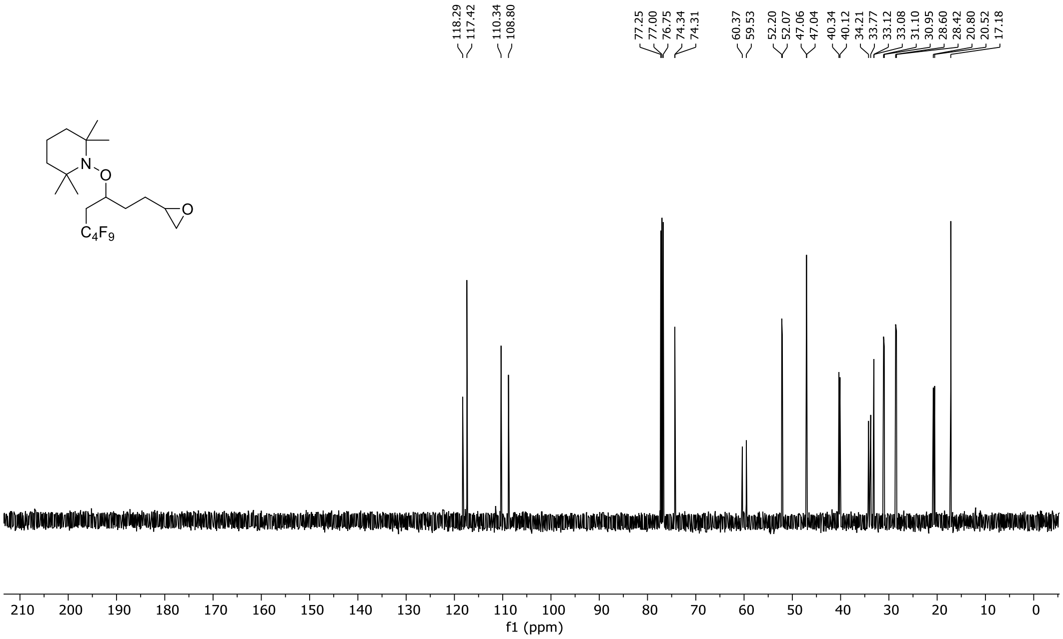

^{19}F NMR (470 MHz, $CDCl_3$)

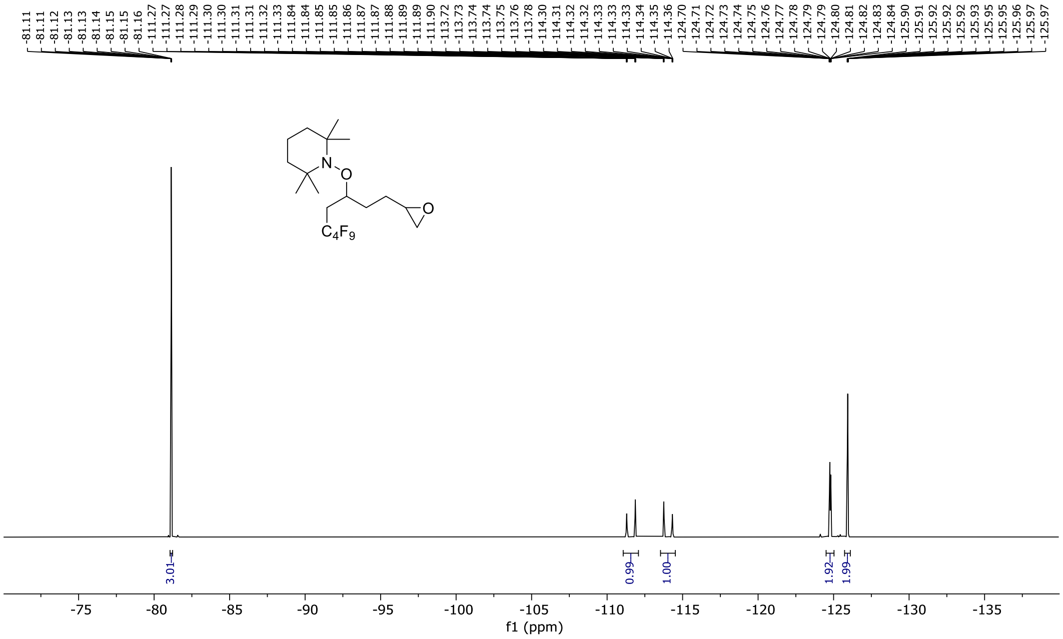

6m

^{1}H NMR (500 MHz, $CDCl_3$)

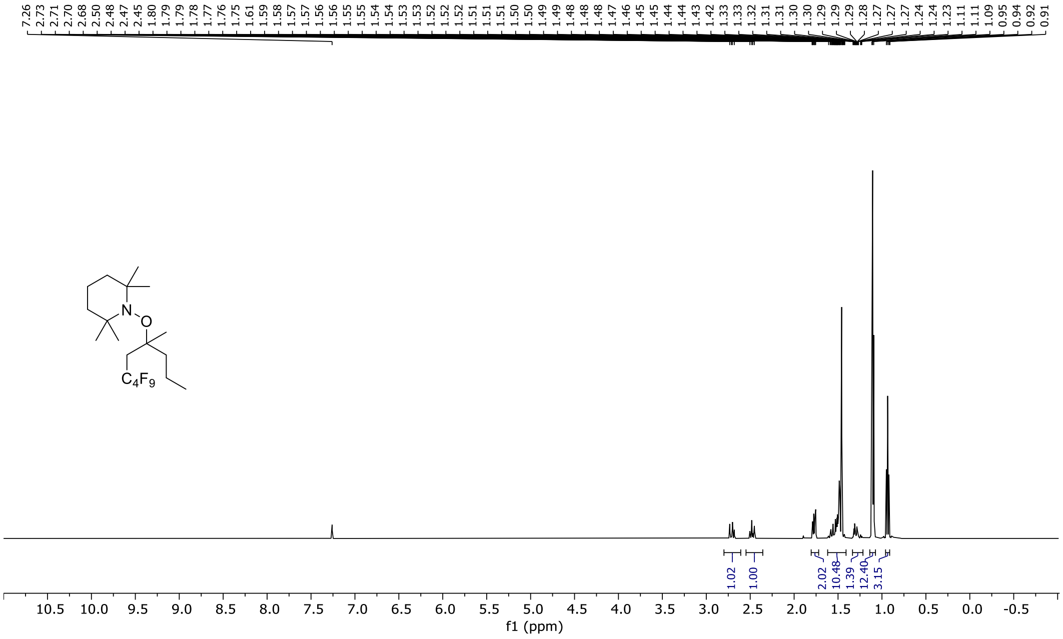

$^{13}C\{^{19}F\}$ NMR (126 MHz, $CDCl_3$)

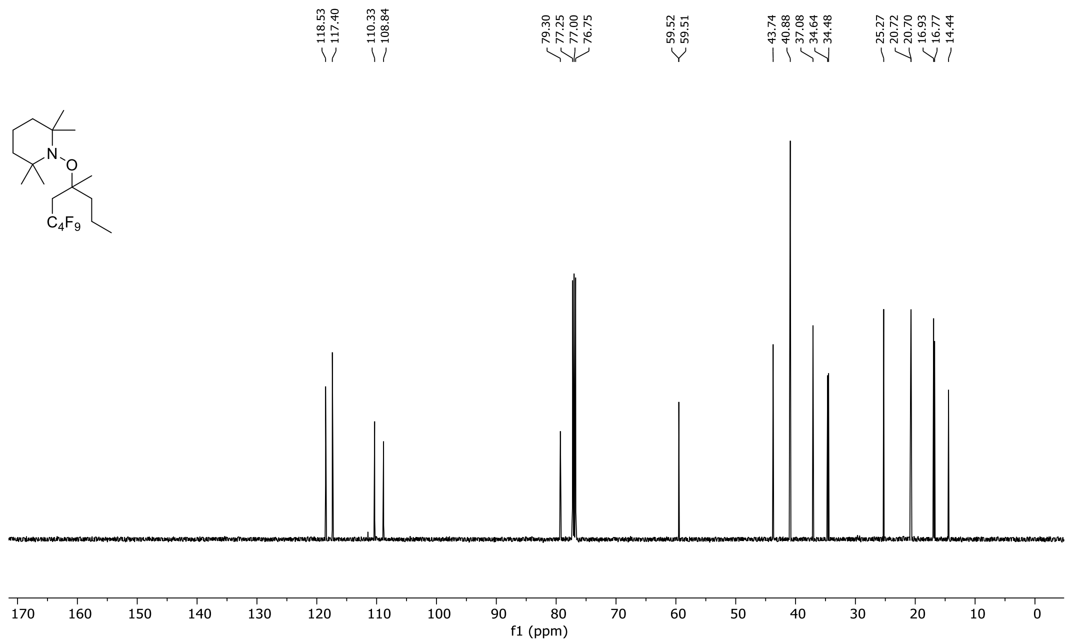

^{19}F NMR (470 MHz, $CDCl_3$)

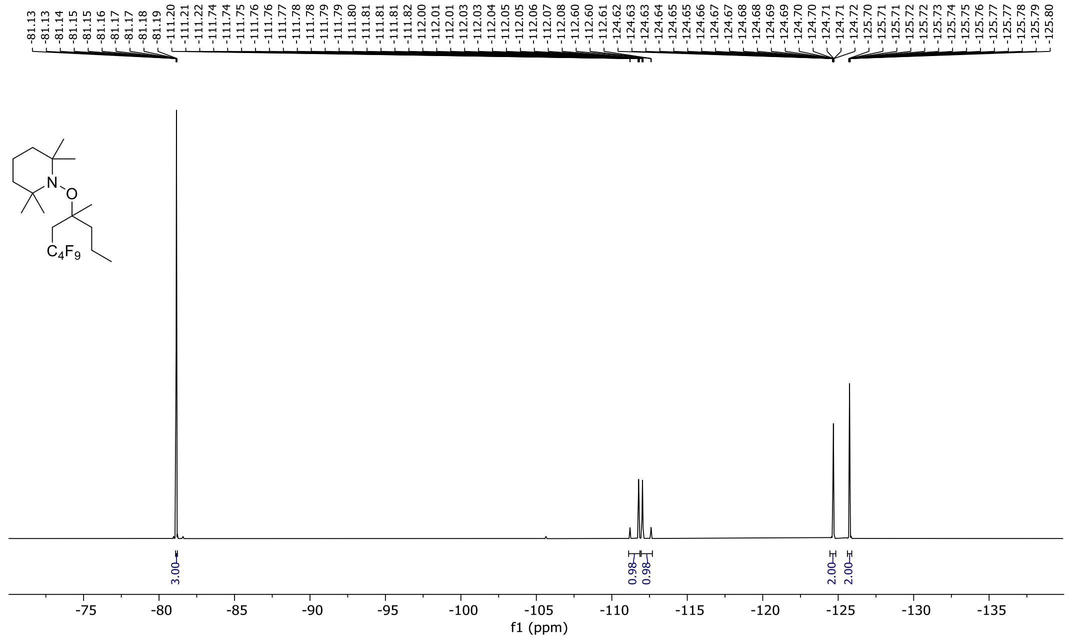

6n

^{1}H NMR (500 MHz, CDCl$_3$)

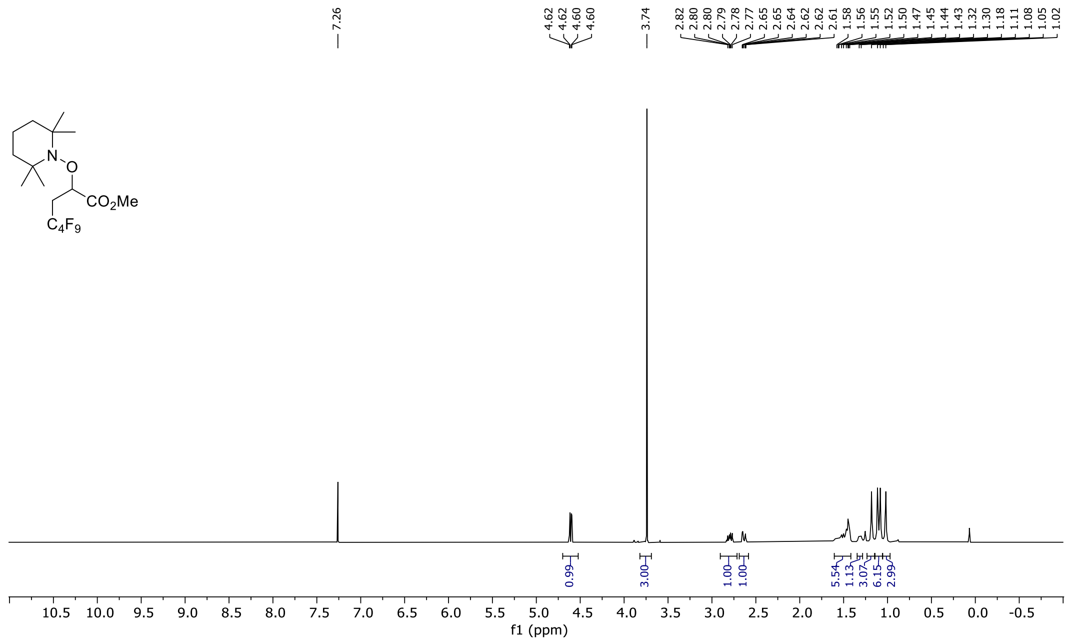

^{13}C{^{19}F} NMR (126 MHz, CDCl$_3$)

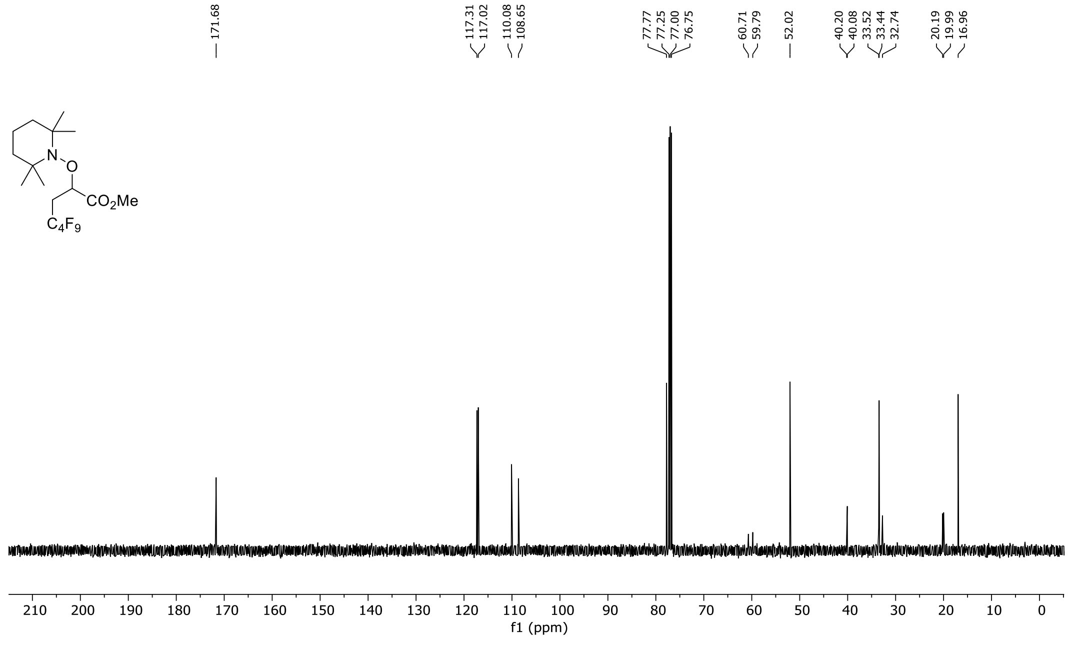

^{19}F NMR (470 MHz, $CDCl_3$)

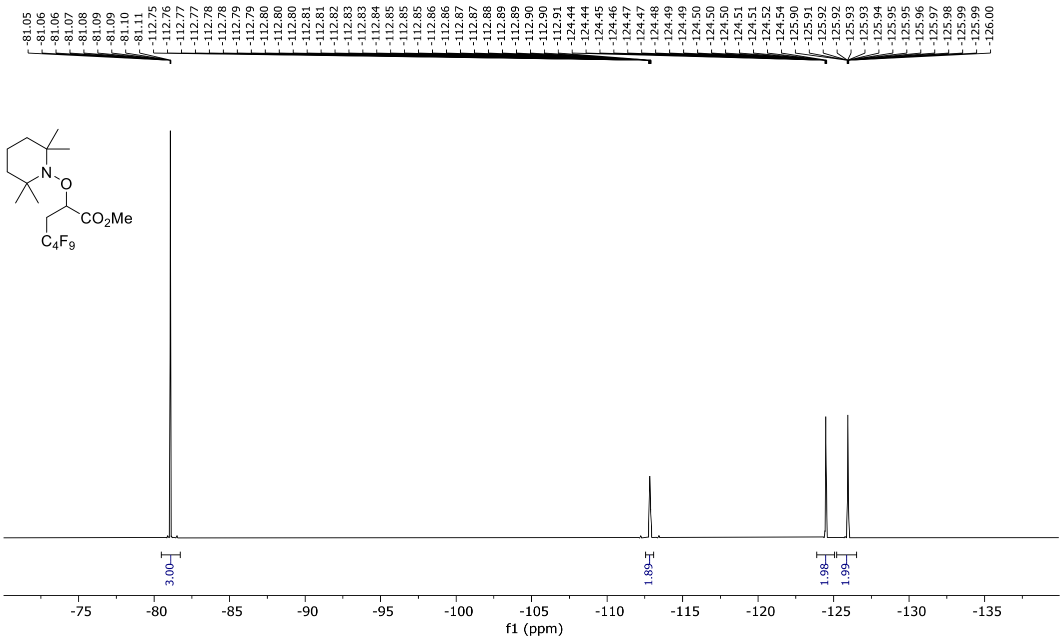

6o

^{1}H NMR (300 MHz, $CDCl_3$)

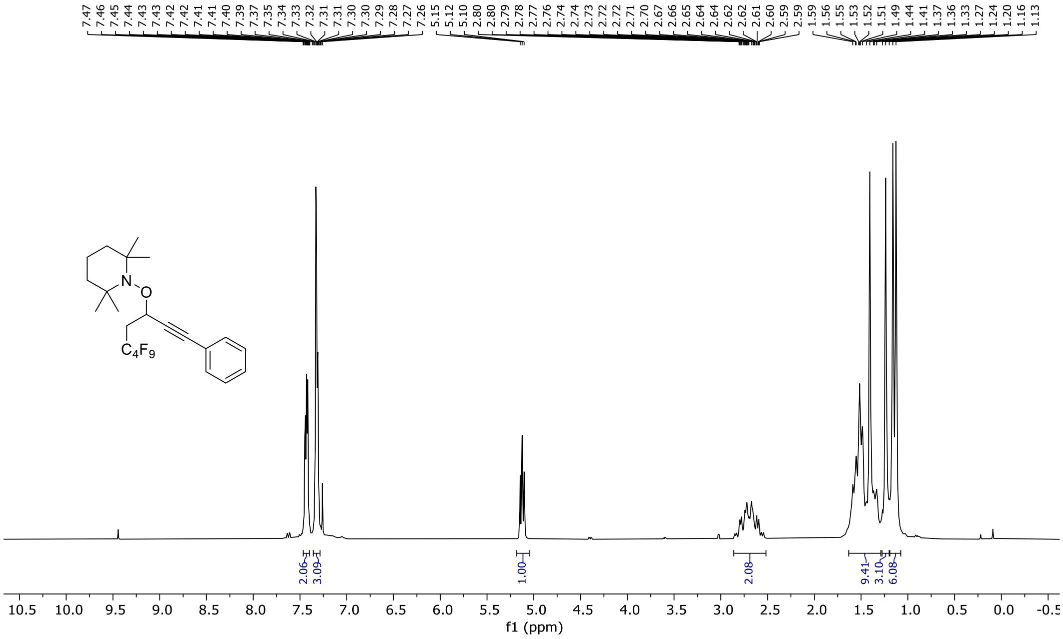

$^{13}C\{^{19}F\}$ NMR (151 MHz, $CDCl_3$)

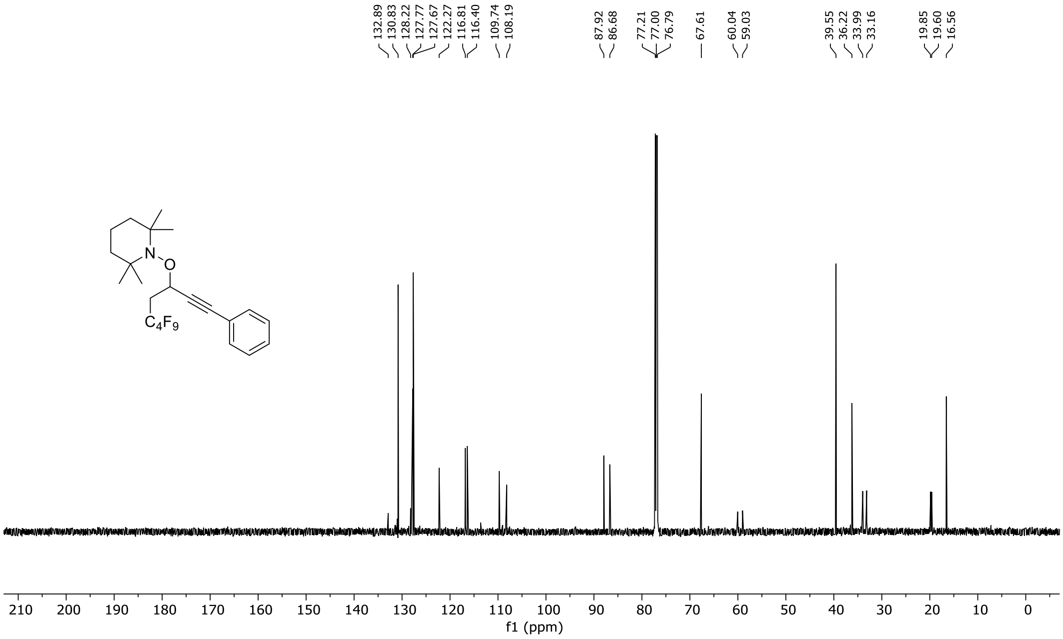

^{19}F NMR (564 MHz, $CDCl_3$)

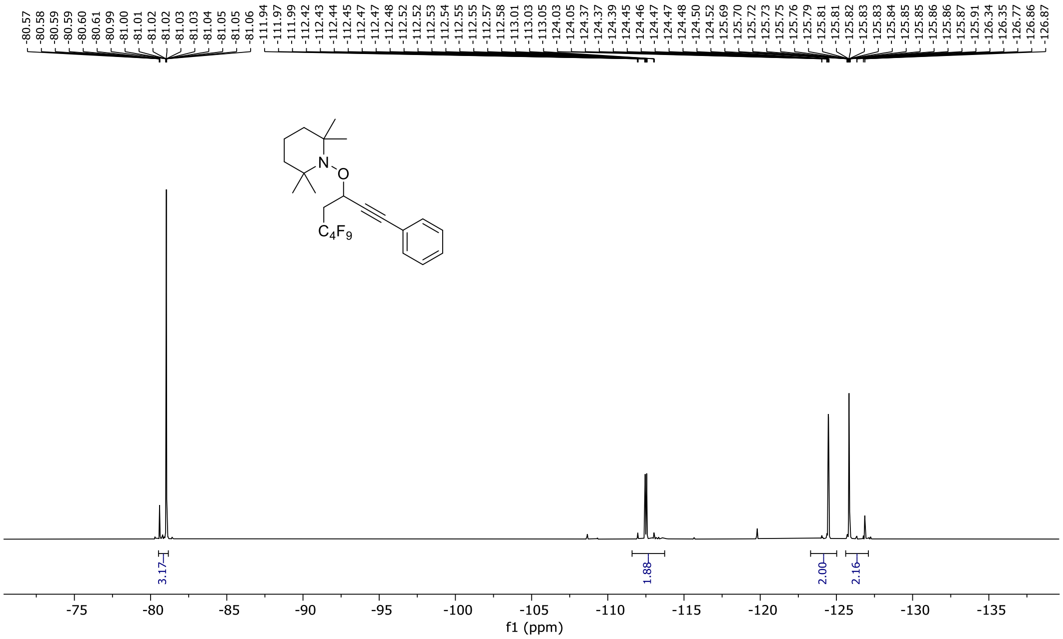

6P

^{1}H NMR (500 MHz, $CDCl_3$)

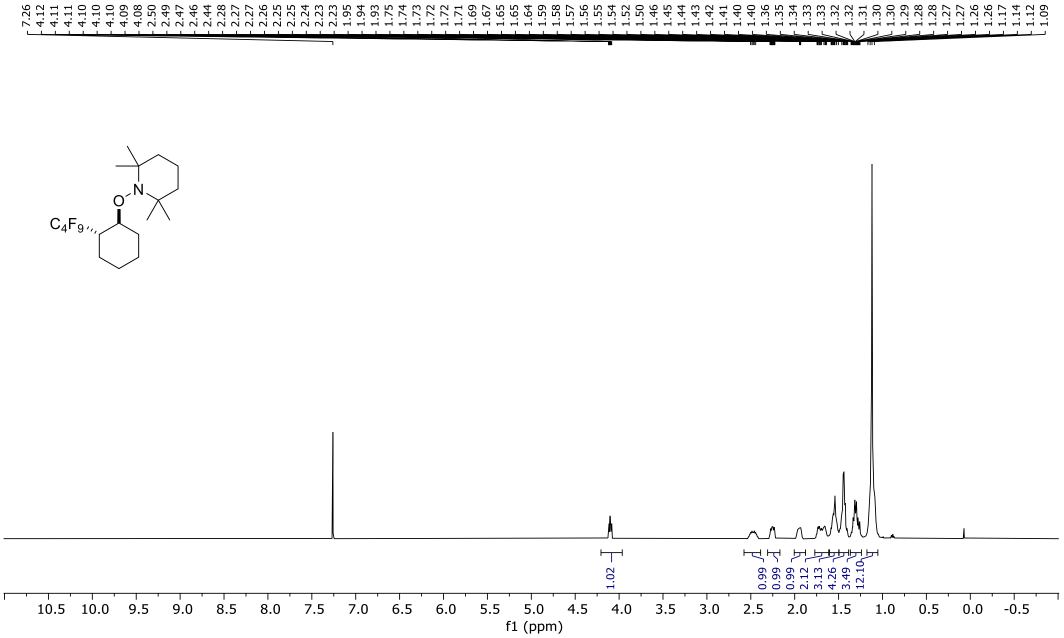

^{13}C{^{19}F} NMR (126 MHz, $CDCl_3$)

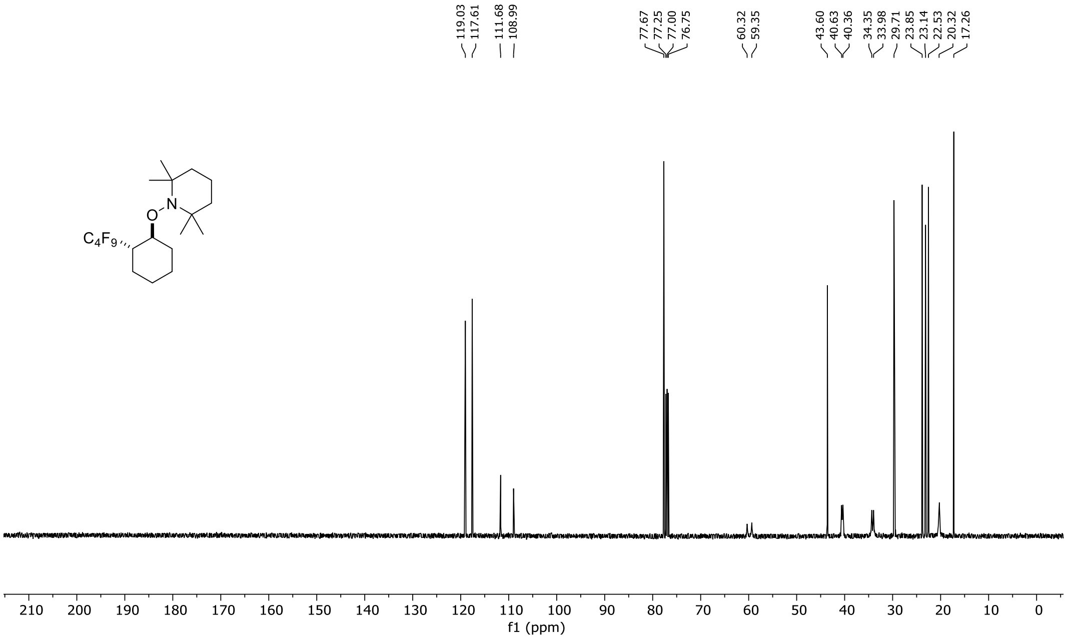

^{19}F NMR (470 MHz, CDCl$_3$)

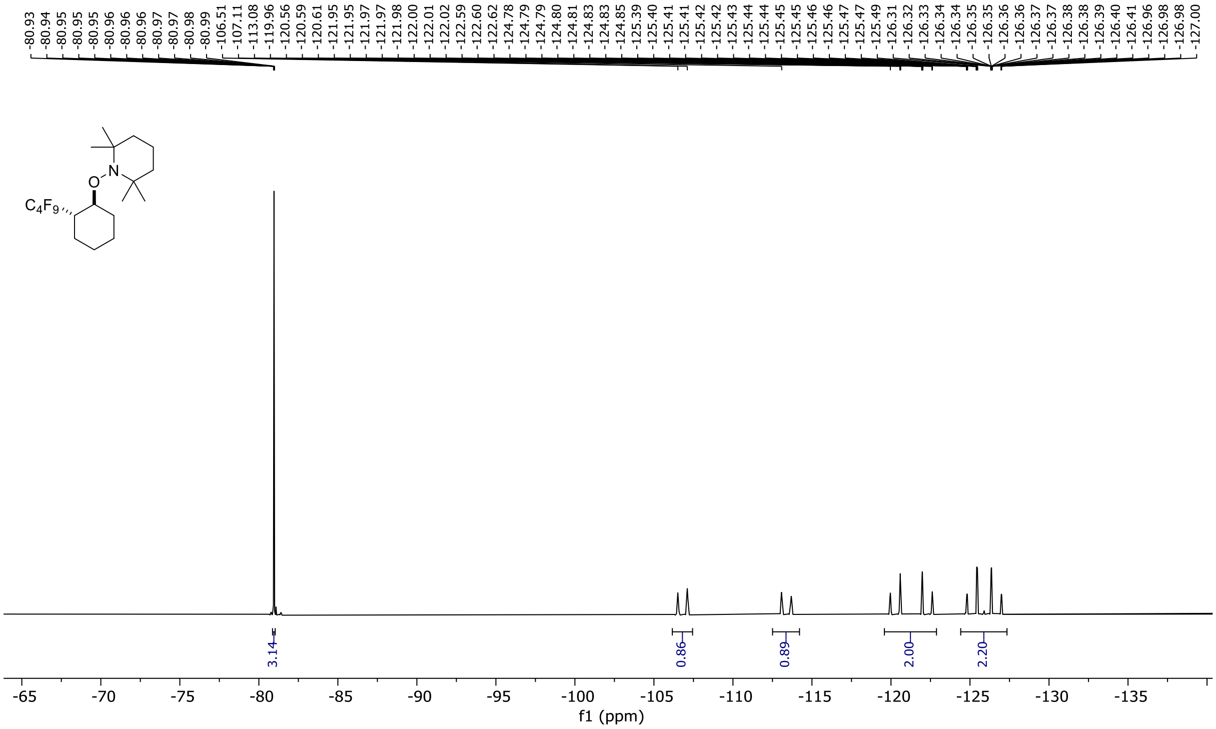

6q

^{1}H NMR (500 MHz, CDCl$_3$)

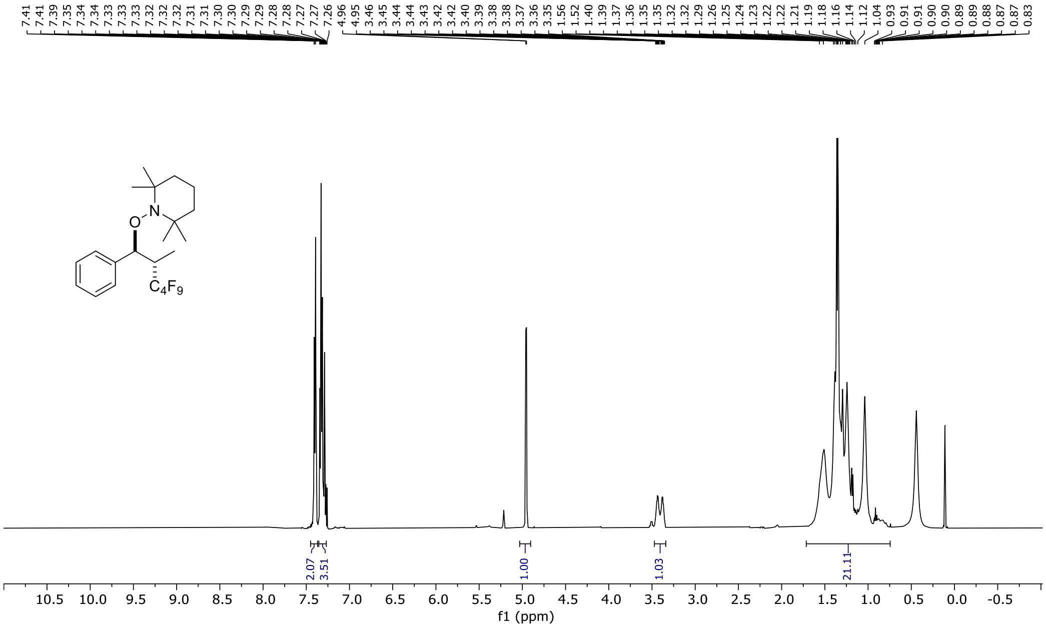

$^{13}C\{^{19}F\}$ NMR (126 MHz, $CDCl_3$)

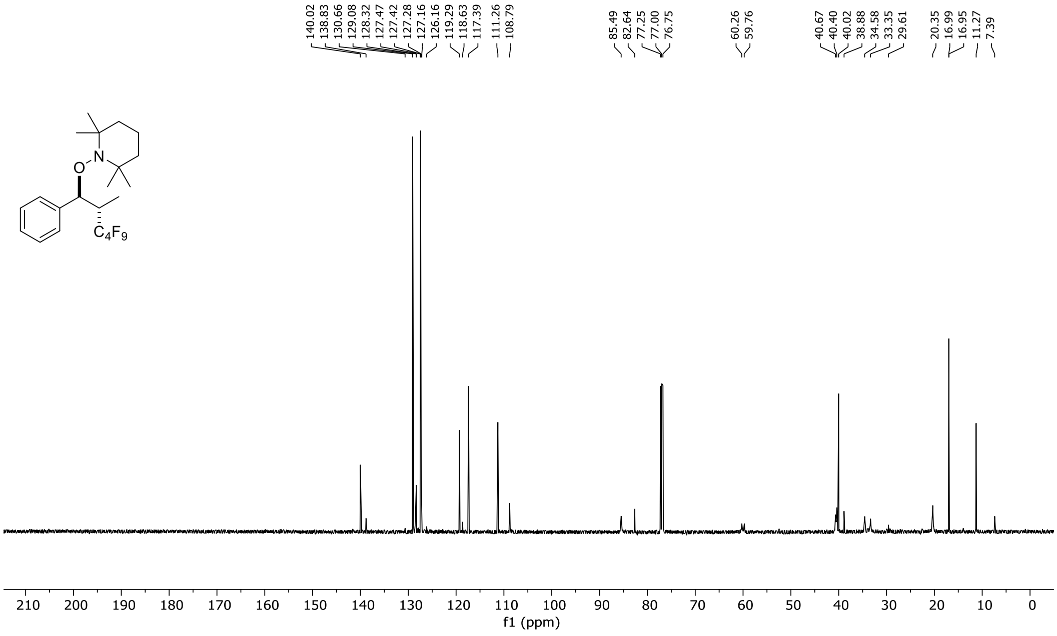

^{19}F NMR (470 MHz, $CDCl_3$)

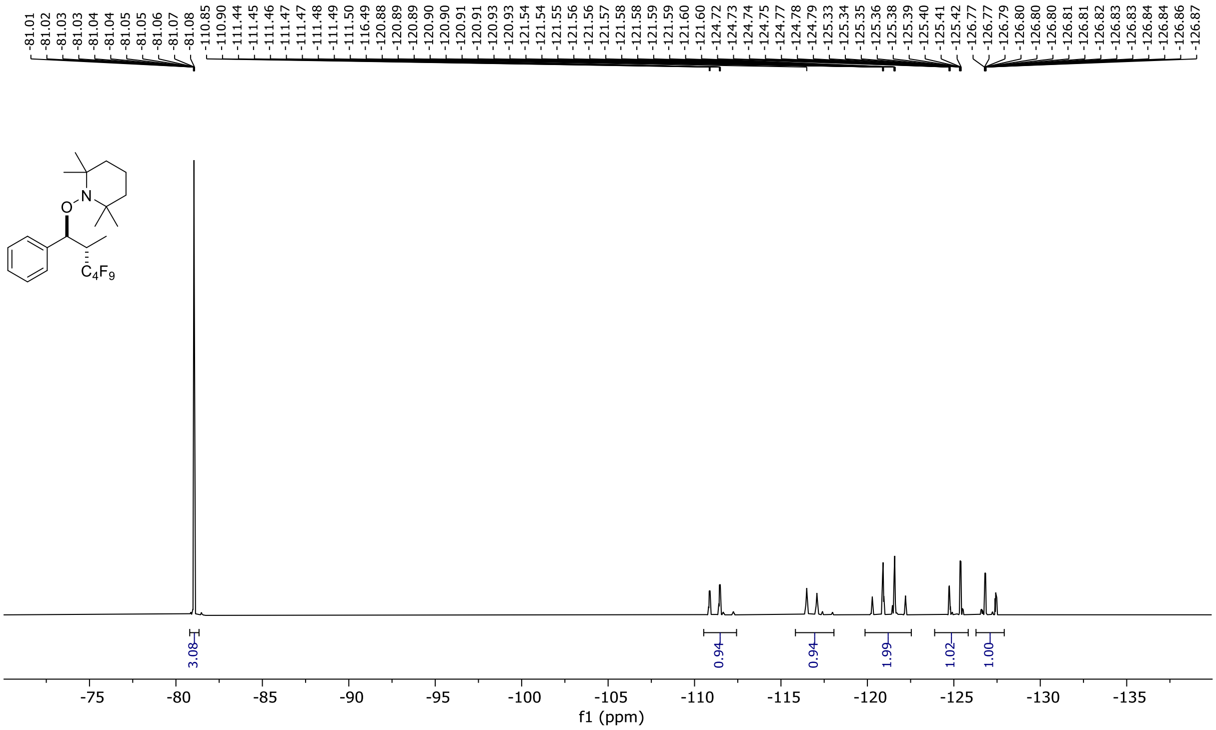

6r

^{1}H NMR (500 MHz, $CDCl_3$)

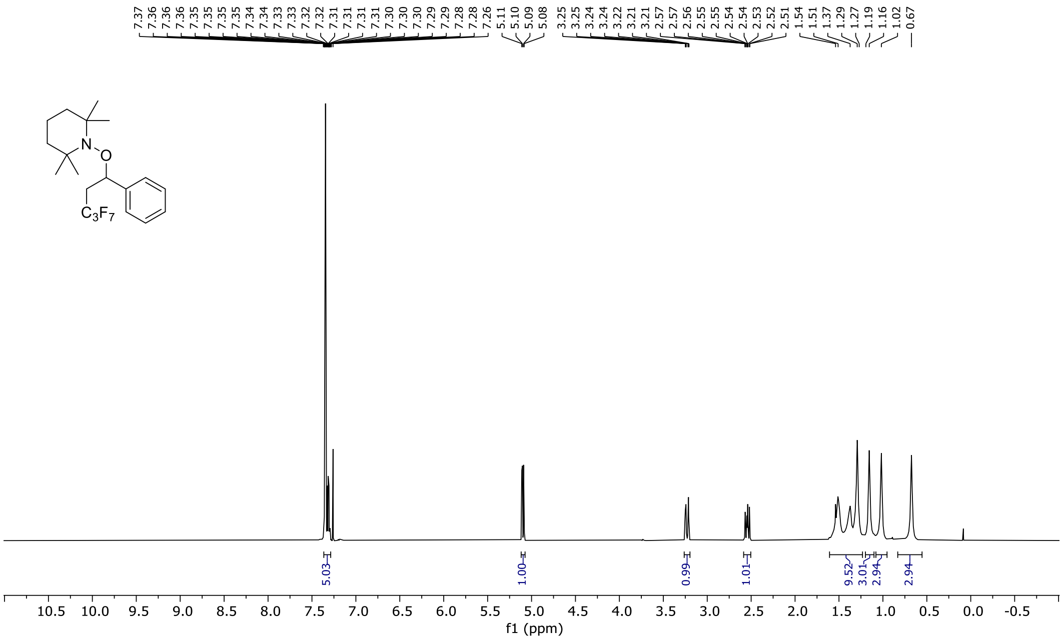

$^{13}C\{^{19}F\}$ NMR (151 MHz, $CDCl_3$)

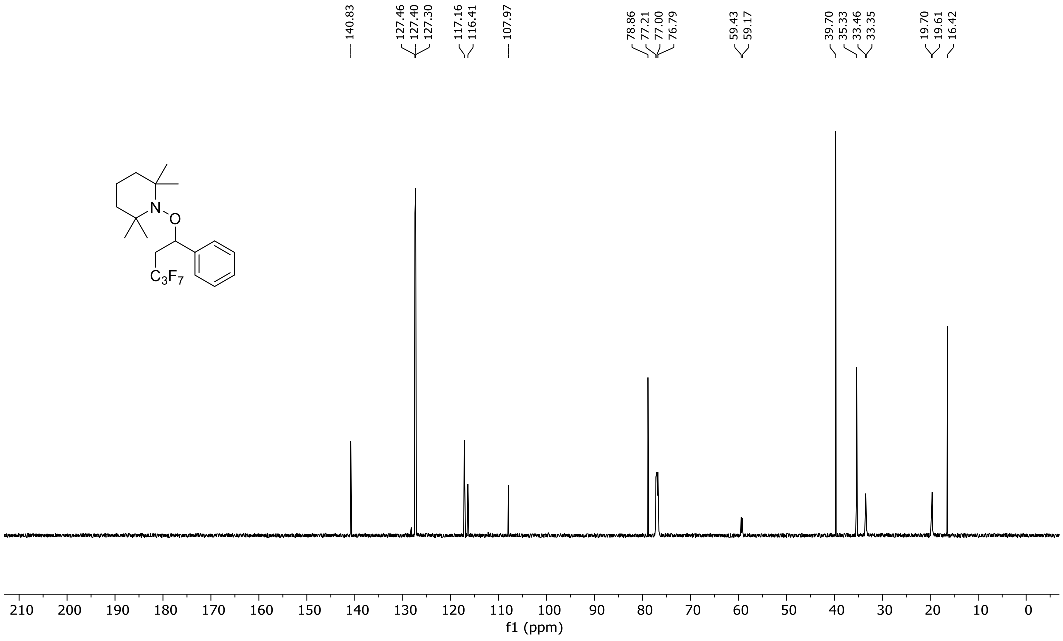

^{19}F NMR (470 MHz, $CDCl_3$)

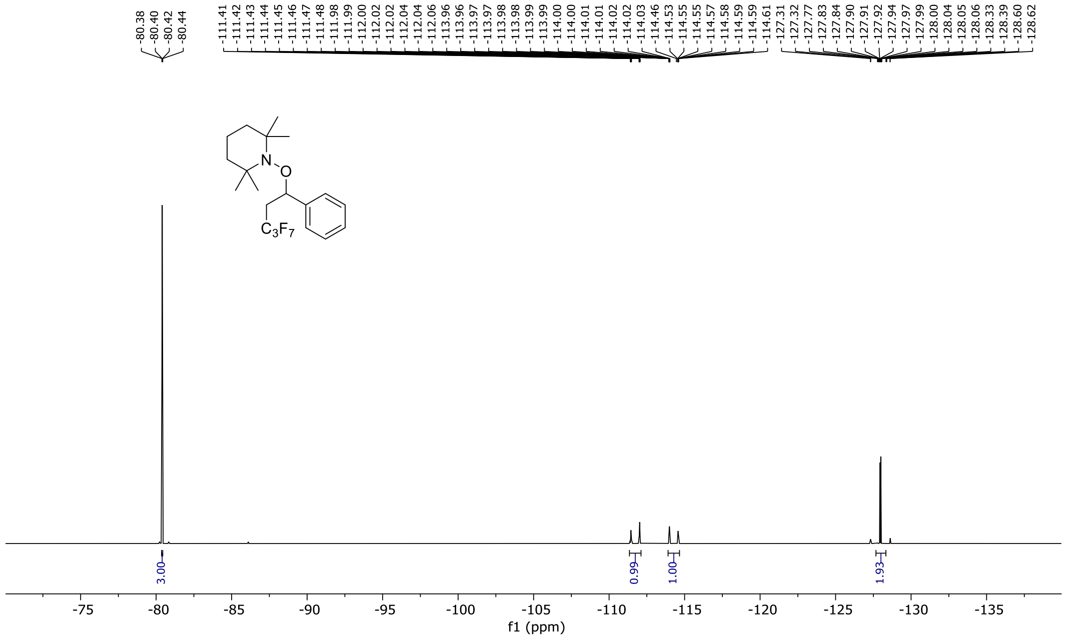

6s

^{1}H NMR (500 MHz, $CDCl_3$)

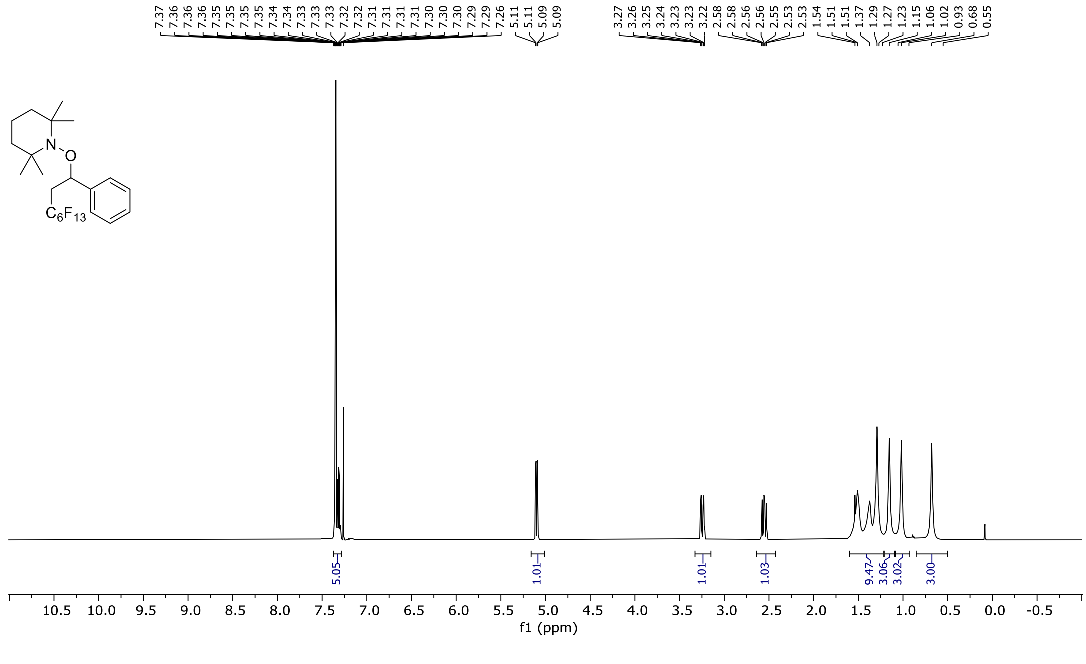

$^{13}C\{^{19}F\}$ NMR (126 MHz, $CDCl_3$)

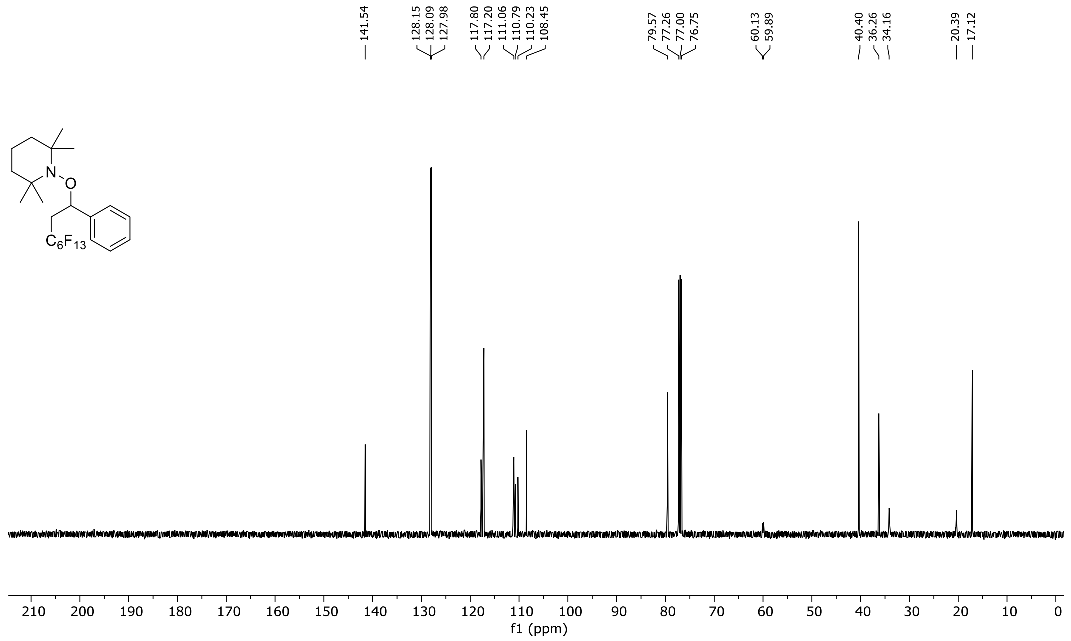

^{19}F NMR (470 MHz, $CDCl_3$)

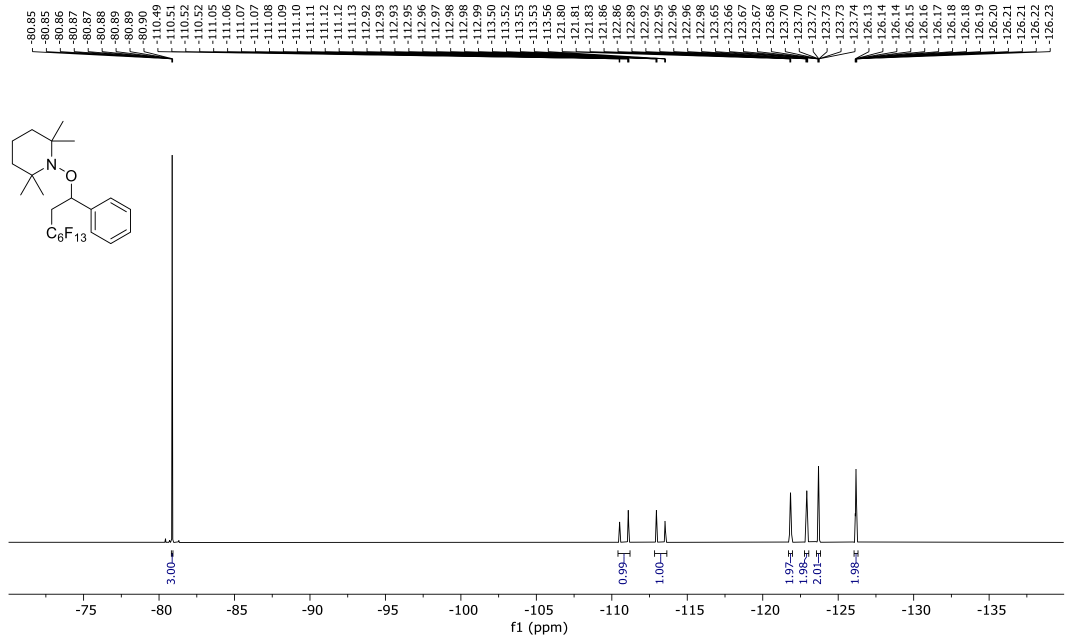

6t

^{1}H NMR (500 MHz, $CDCl_3$)

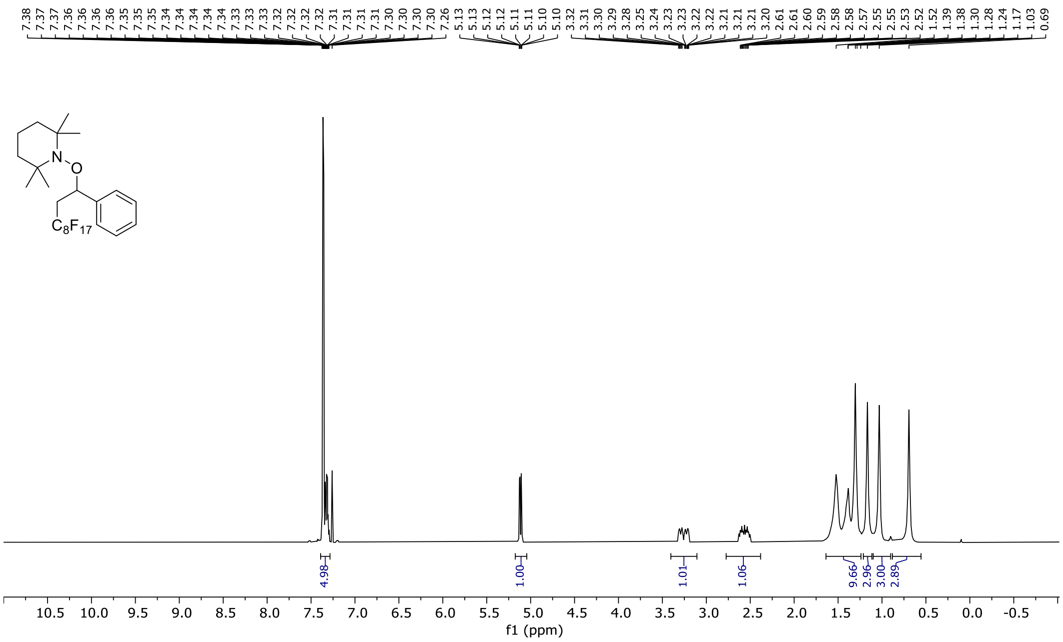

$^{13}C\{^{19}F\}$ NMR (126 MHz, $CDCl_3$)

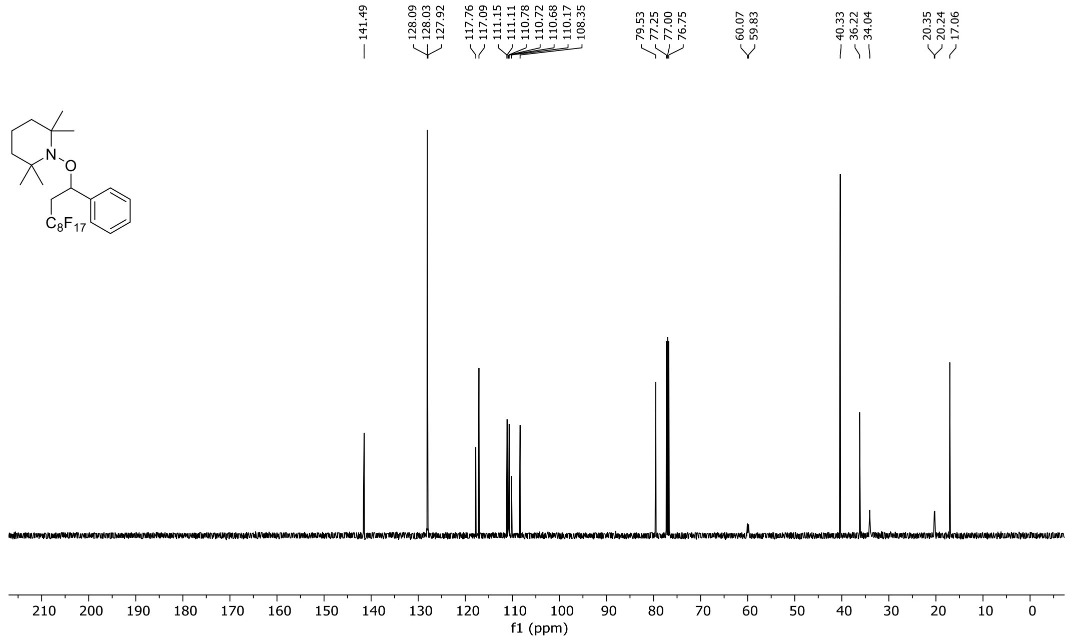

^{19}F NMR (470 MHz, $CDCl_3$)

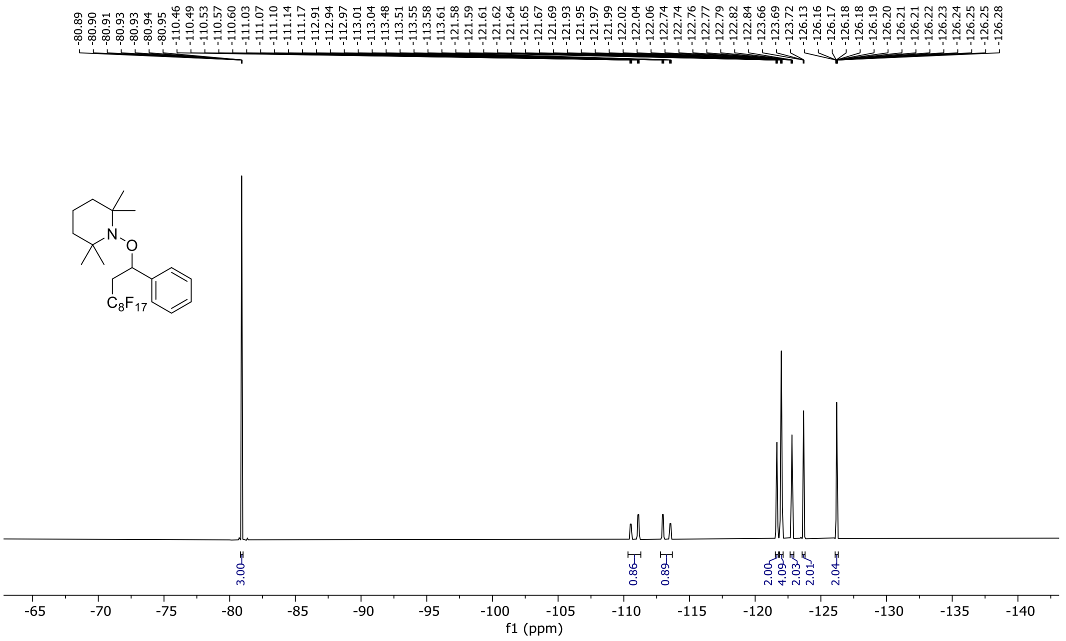

6u

^{1}H NMR (300 MHz, $CDCl_3$)

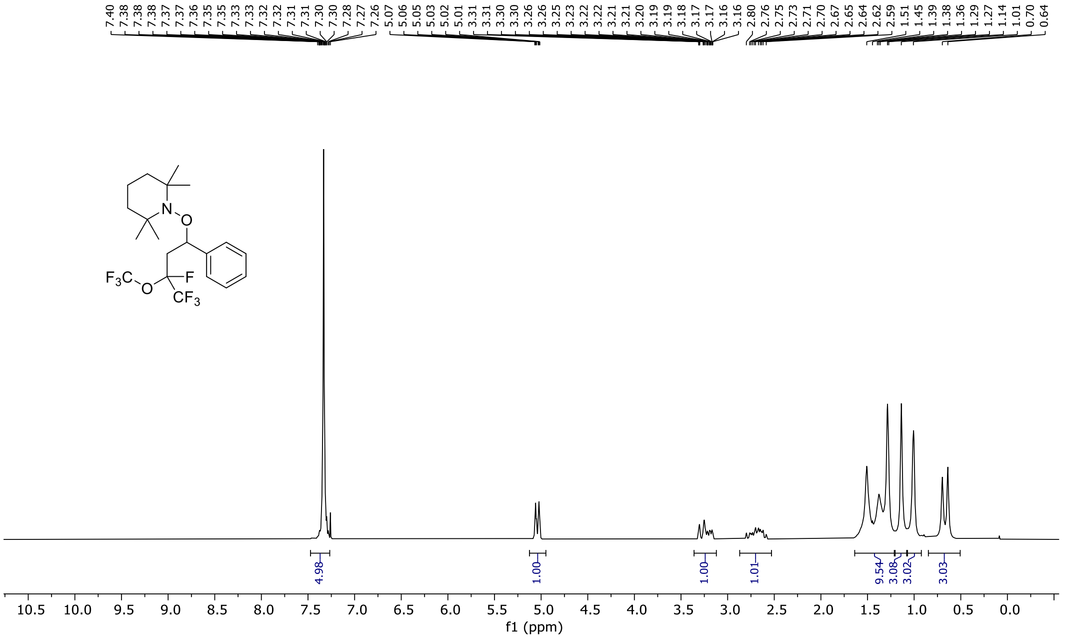

$^{13}C\{^{19}F\}$ NMR (126 MHz, $CDCl_3$)

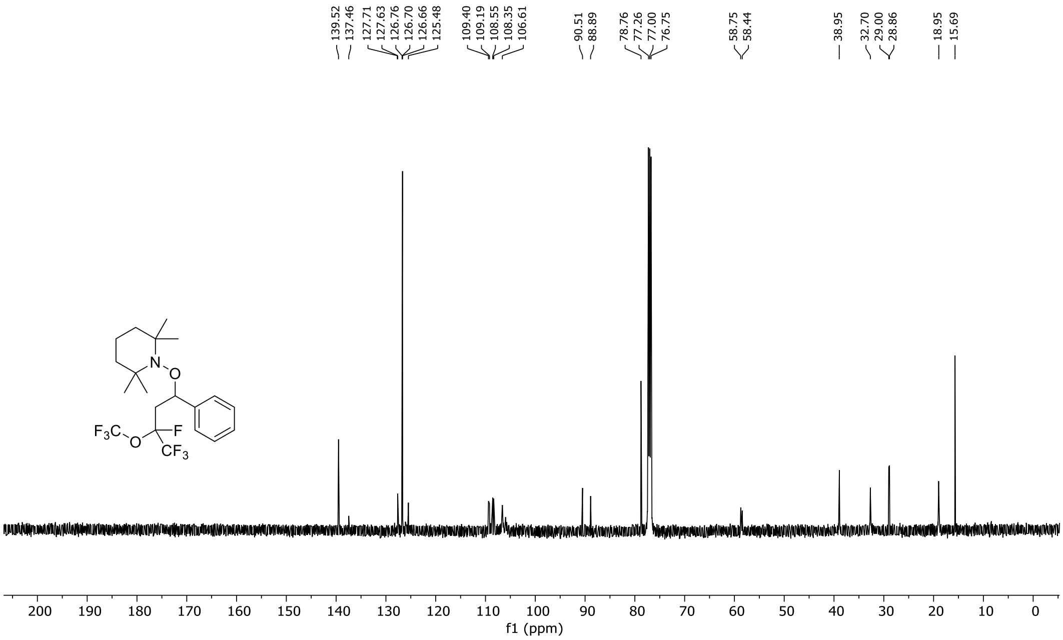

^{19}F NMR (564 MHz, $CDCl_3$)

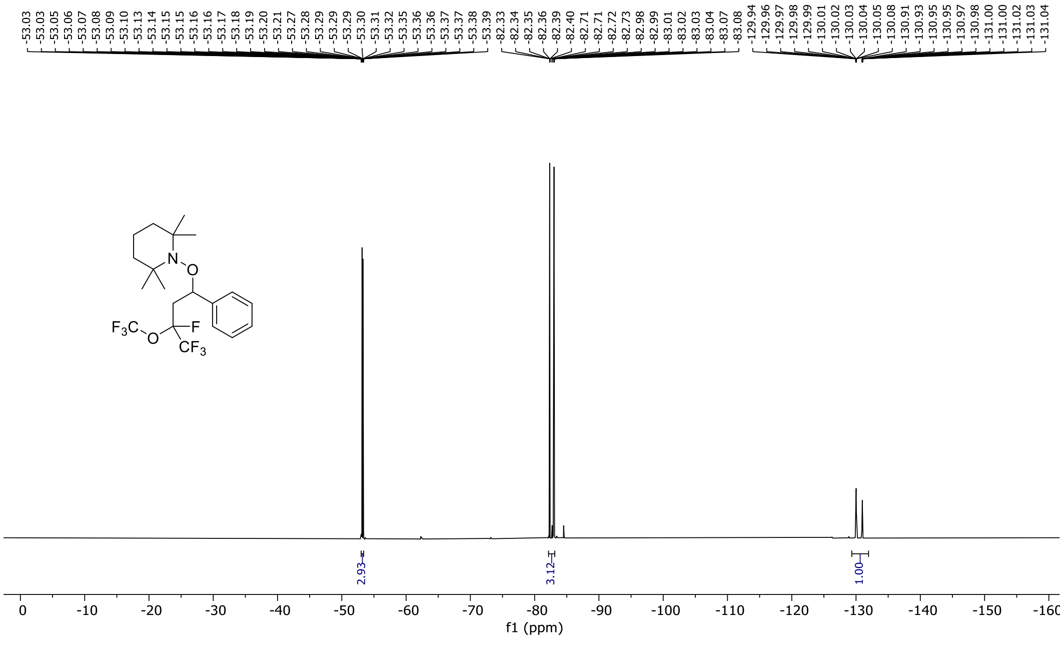

6v

^{1}H NMR (300 MHz, $CDCl_3$)

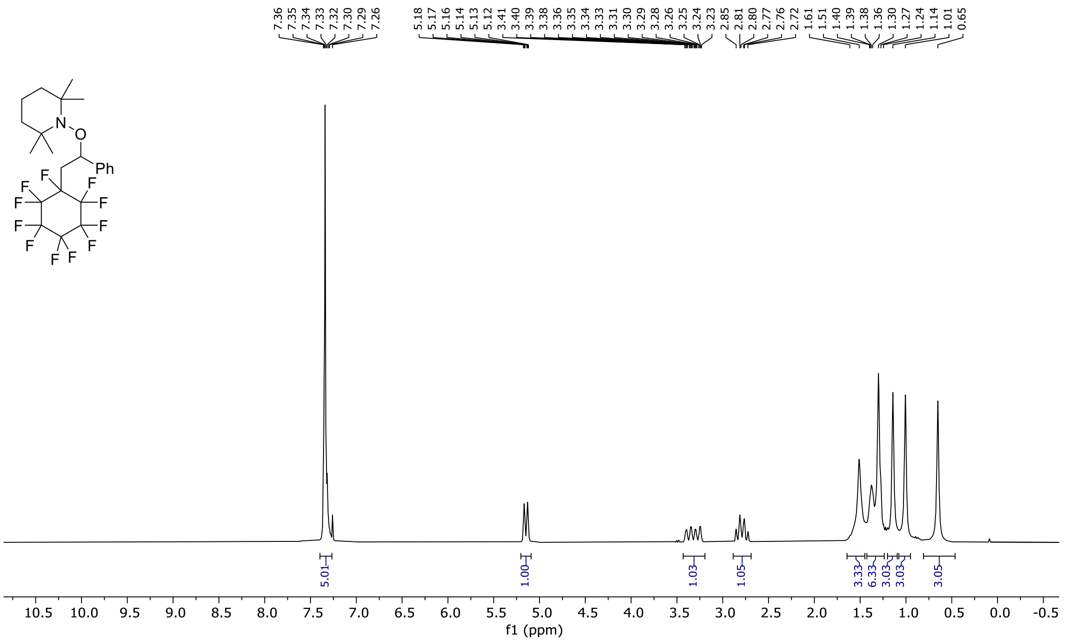

^{13}C{^{19}F} NMR (126 MHz, $CDCl_3$)

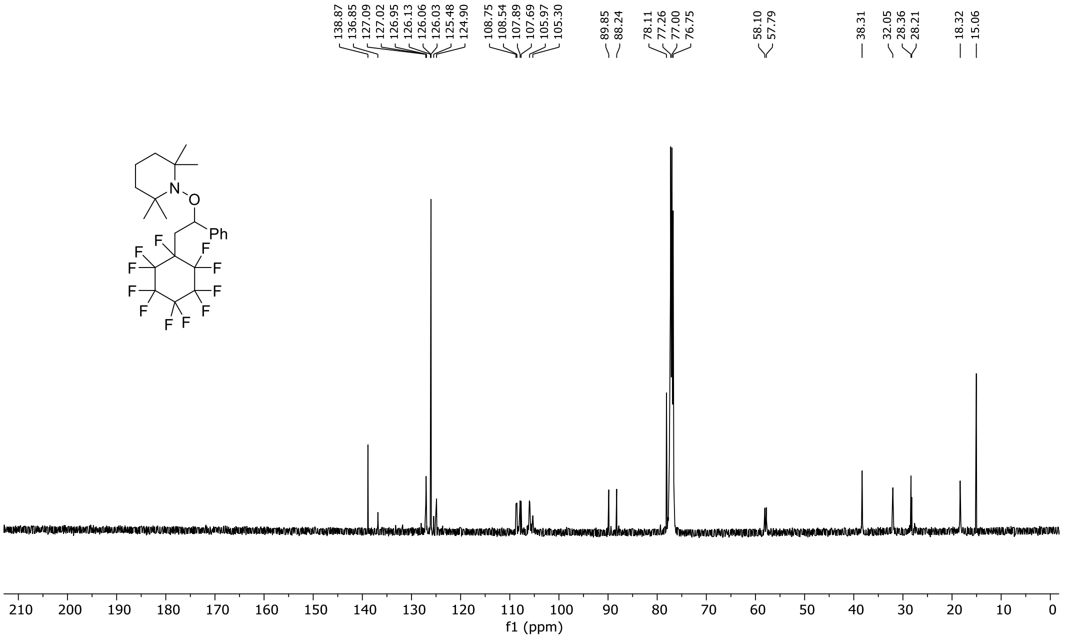

^{19}F NMR (470 MHz, $CDCl_3$)

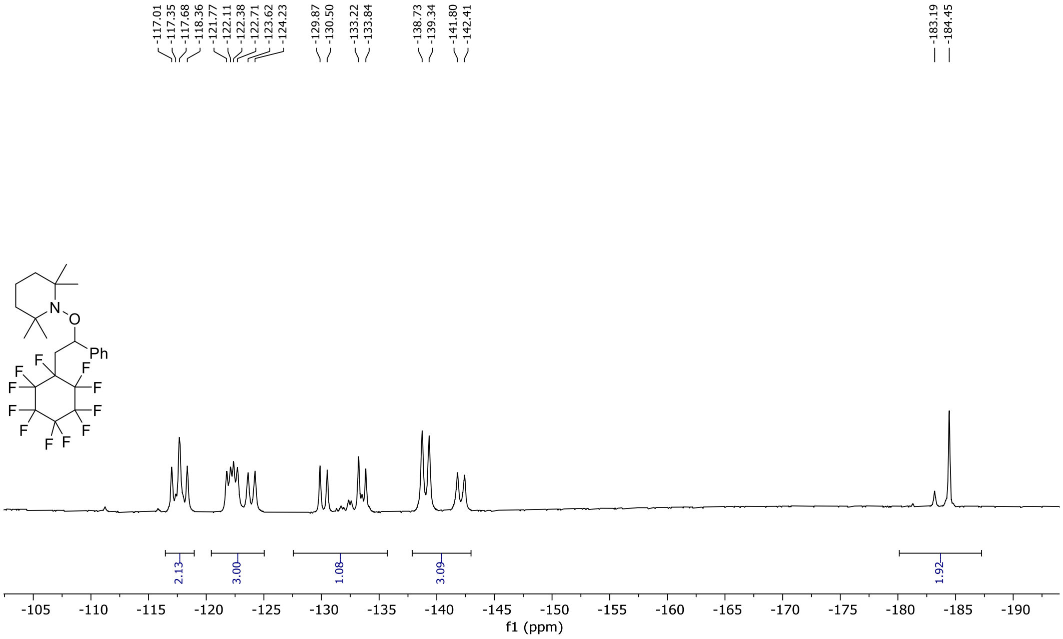

6w

^{1}H NMR (500 MHz, $CDCl_3$)

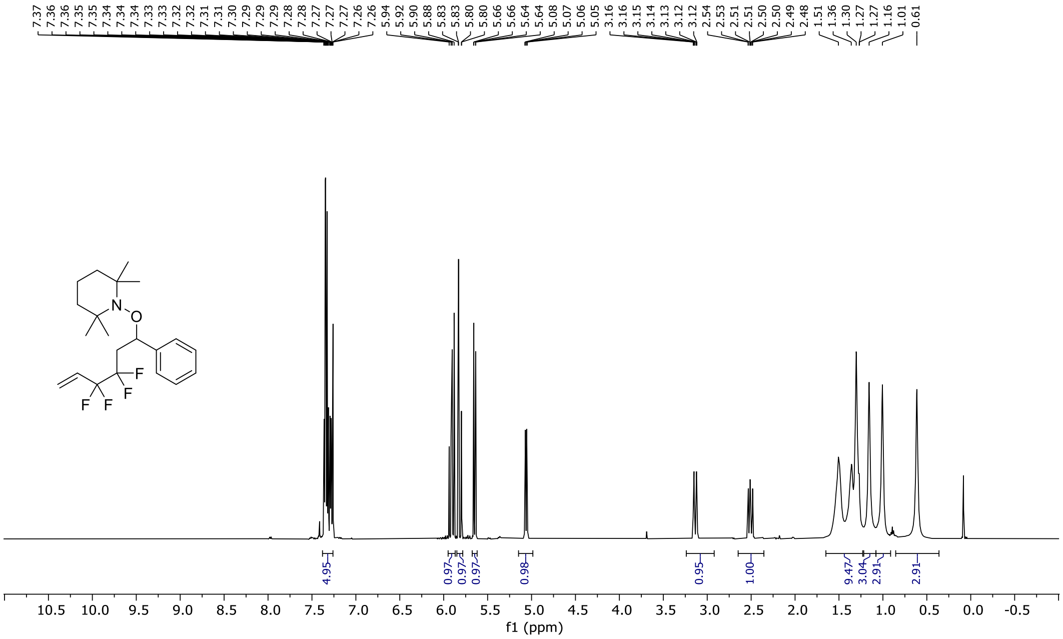

$^{13}C\{^{19}F\}$ NMR (126 MHz, $CDCl_3$)

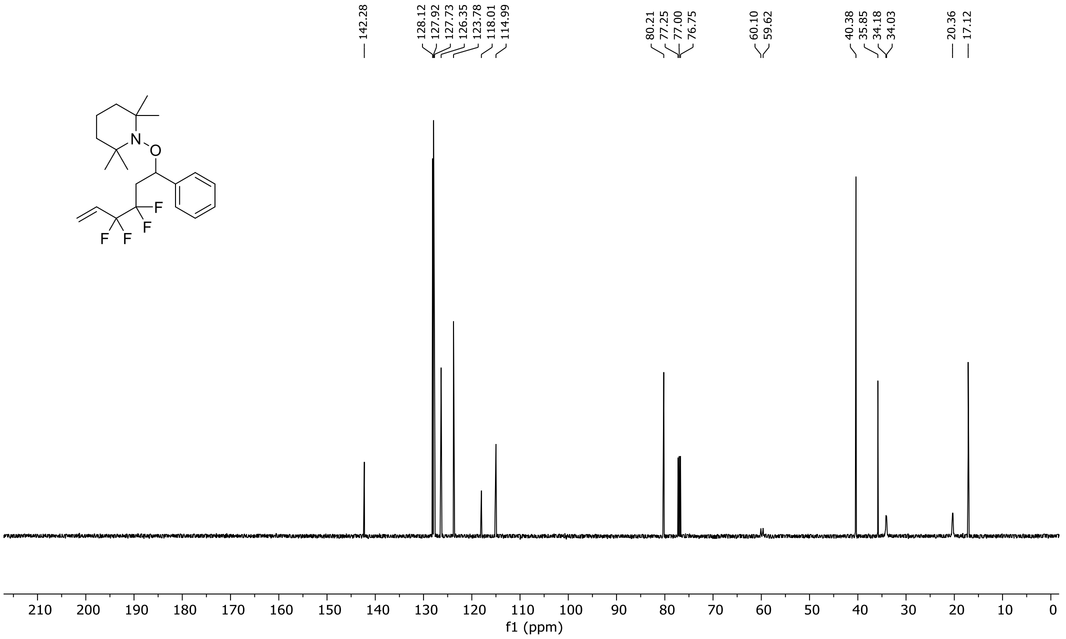

^{19}F NMR (470 MHz, $CDCl_3$)

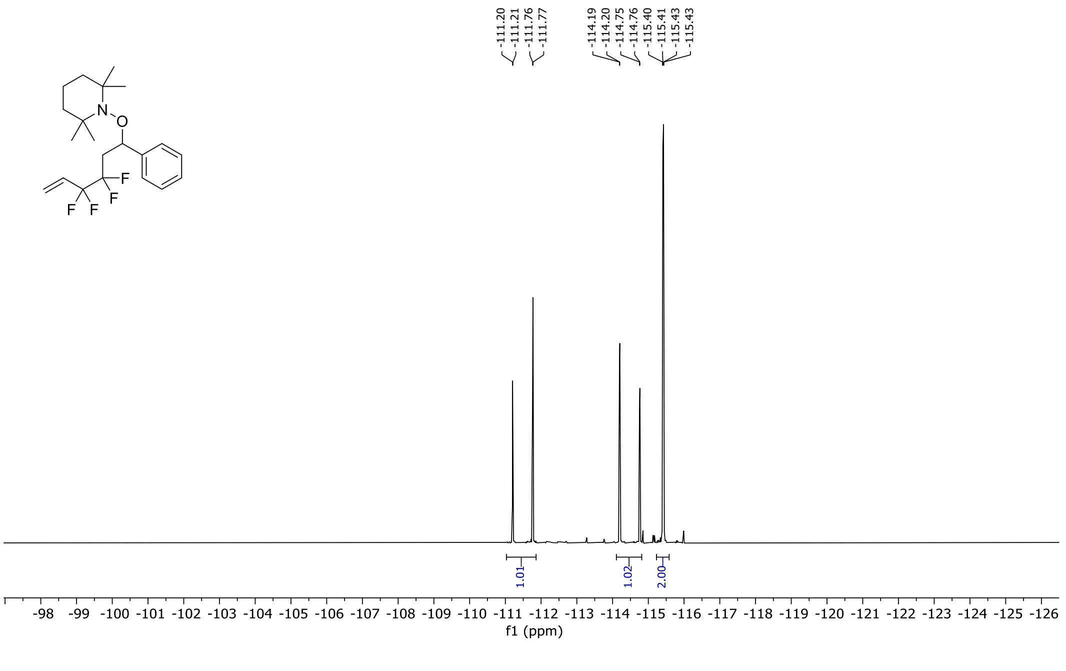

7

^{1}H NMR (300 MHz, $CDCl_3$)

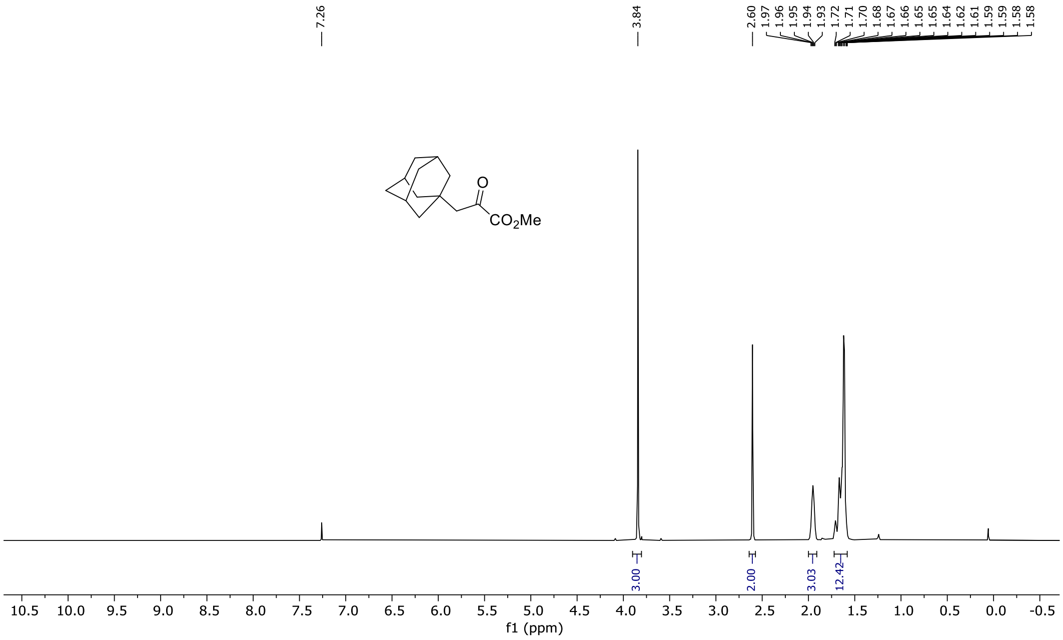

^{13}C NMR (76 MHz, $CDCl_3$)

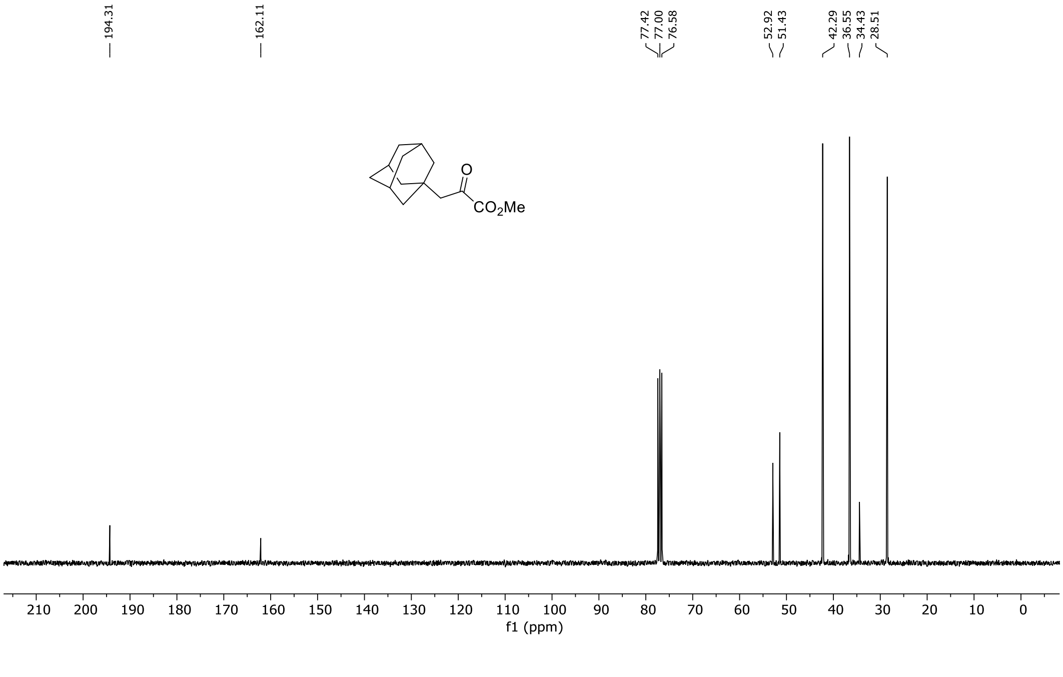

8

^{1}H NMR (300 MHz, $CDCl_3$)

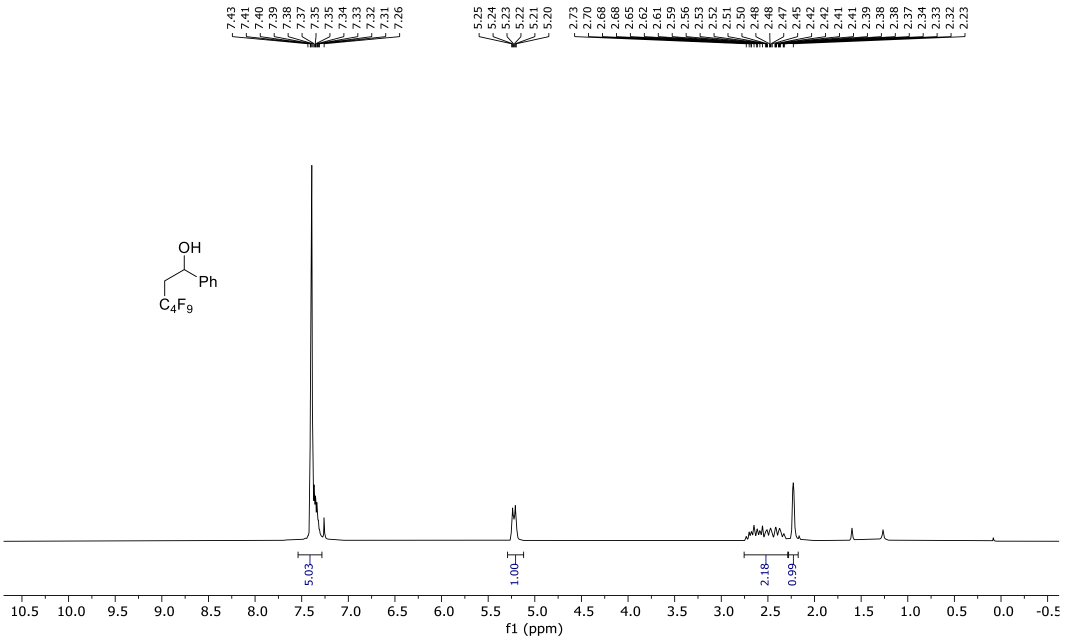

^{13}C{^{19}F} NMR (126 MHz, $CDCl_3$)

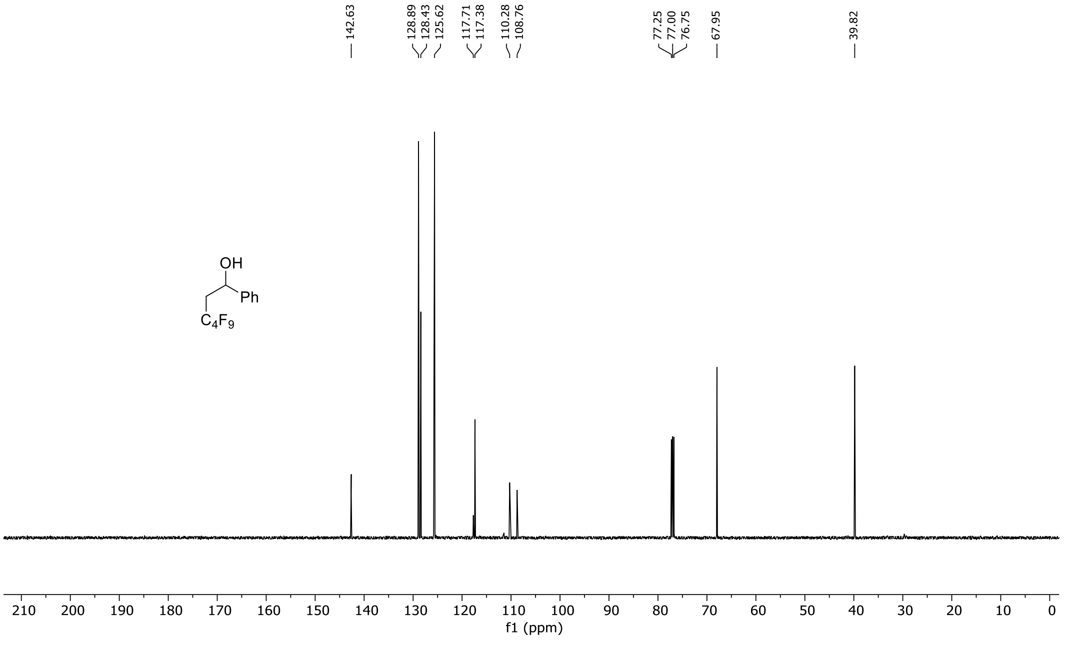

^{19}F NMR (564 MHz, $CDCl_3$)

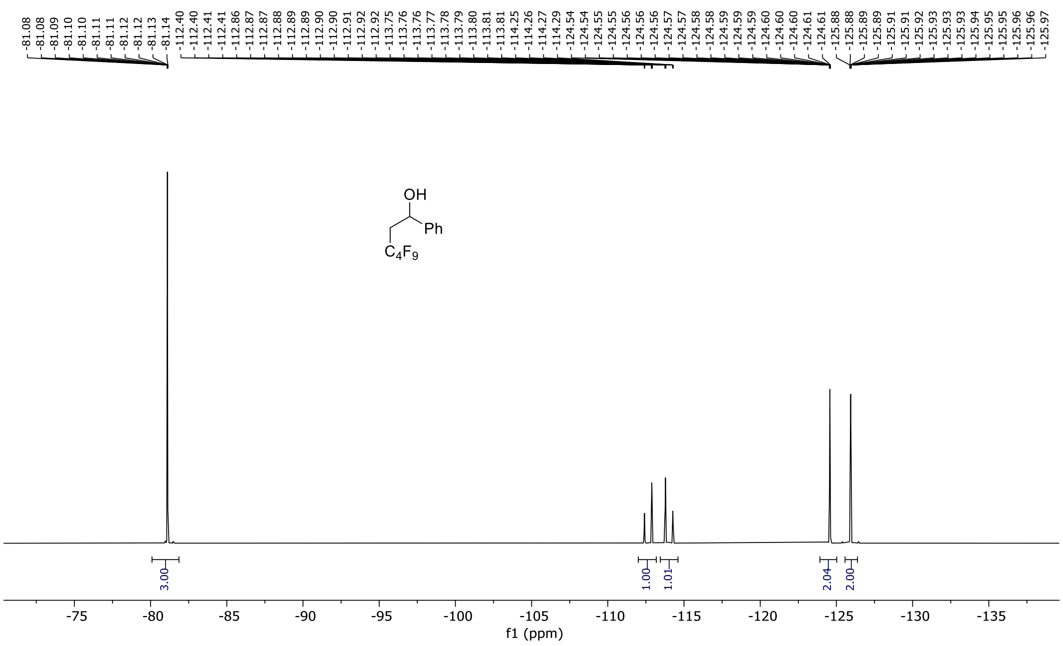

10

^{1}H NMR (300 MHz, $CDCl_3$)

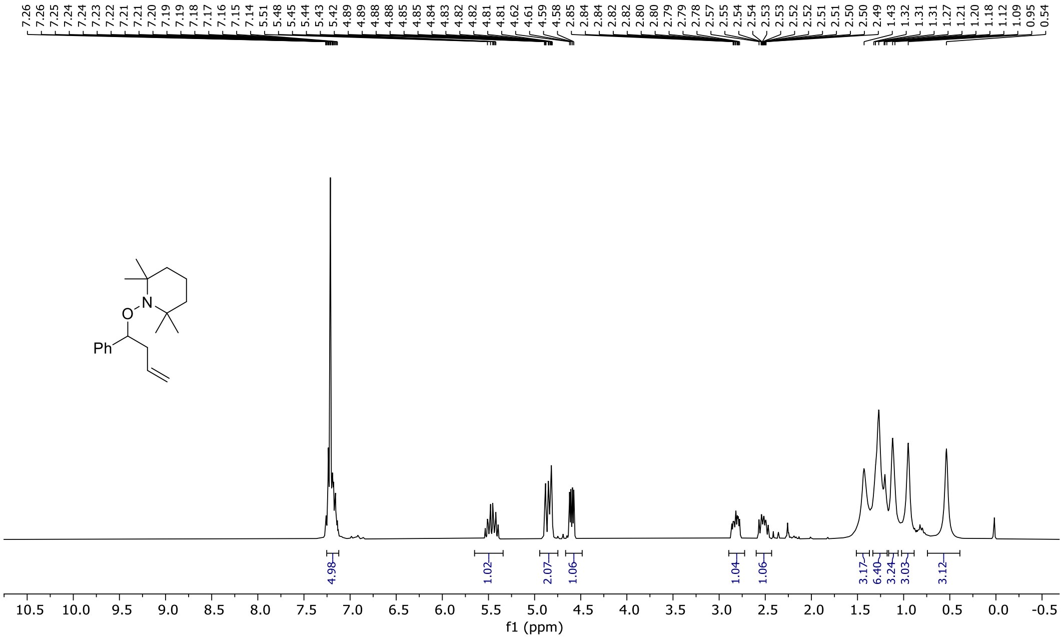

^{13}C NMR (151 MHz, $CDCl_3$)

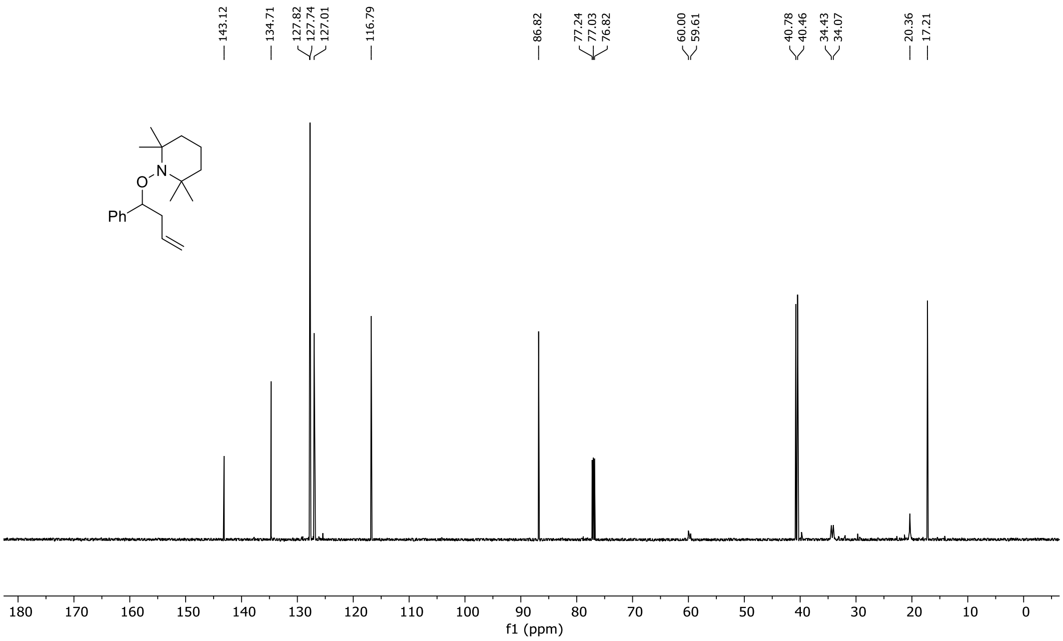

13

^{1}H NMR (300 MHz, CD_3COCD_3)

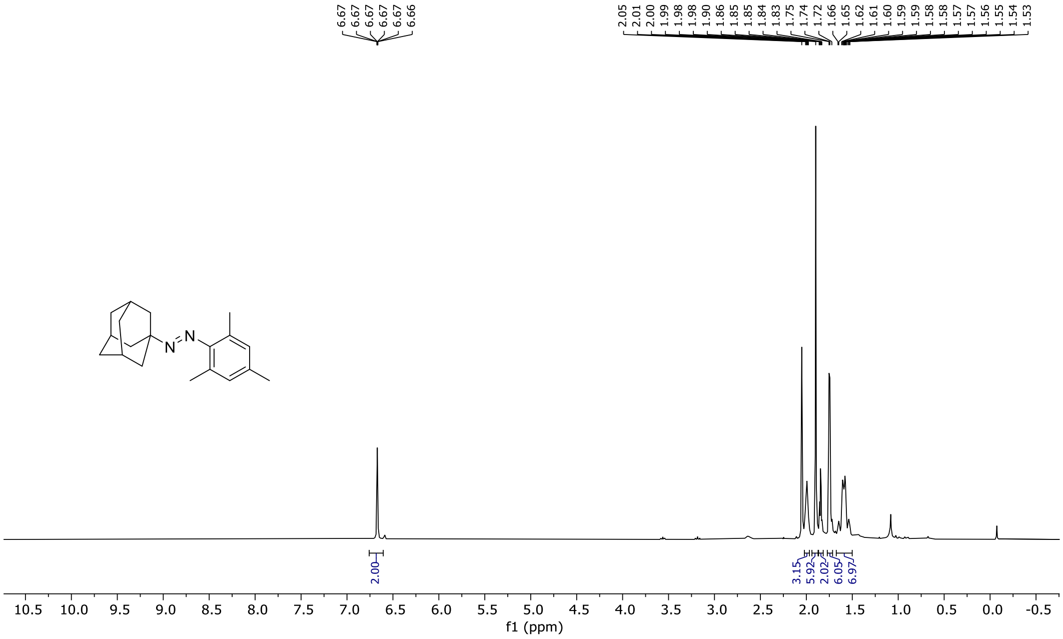

^{13}C NMR (126 MHz, CD_3COCD_3)

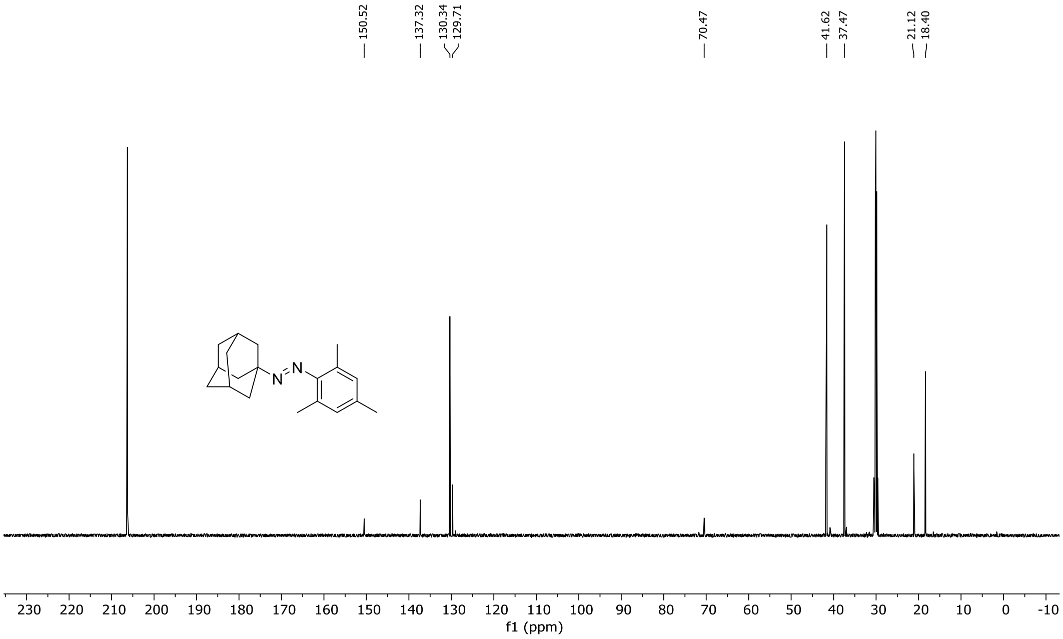

12a

^{1}H NMR (500 MHz, $CDCl_3$)

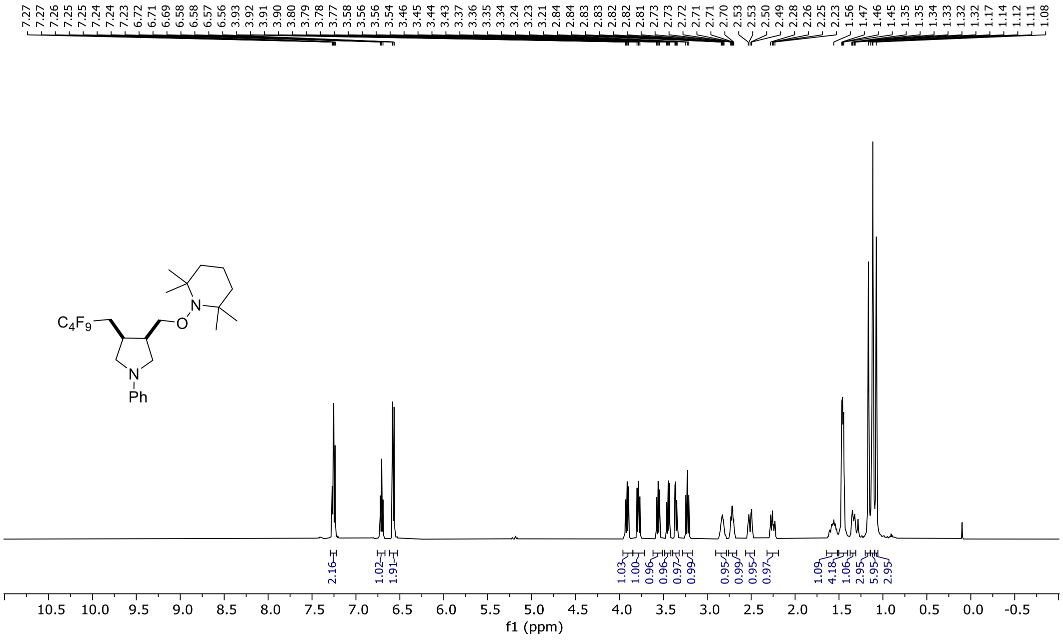

$^{13}C\{^{19}F\}$ NMR (126 MHz, $CDCl_3$)

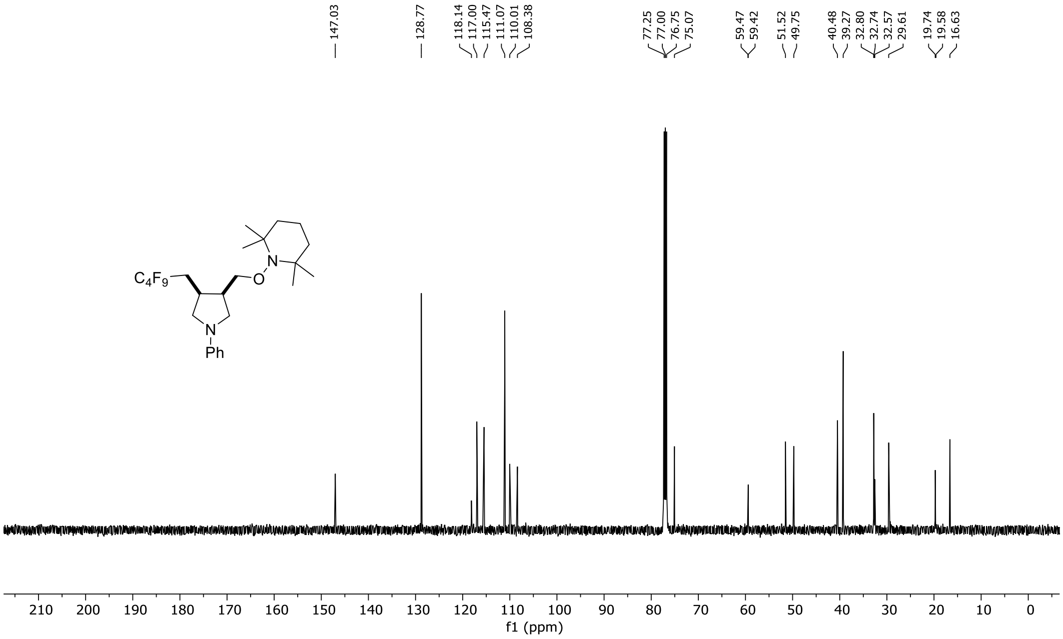

^{19}F NMR (470 MHz, $CDCl_3$)

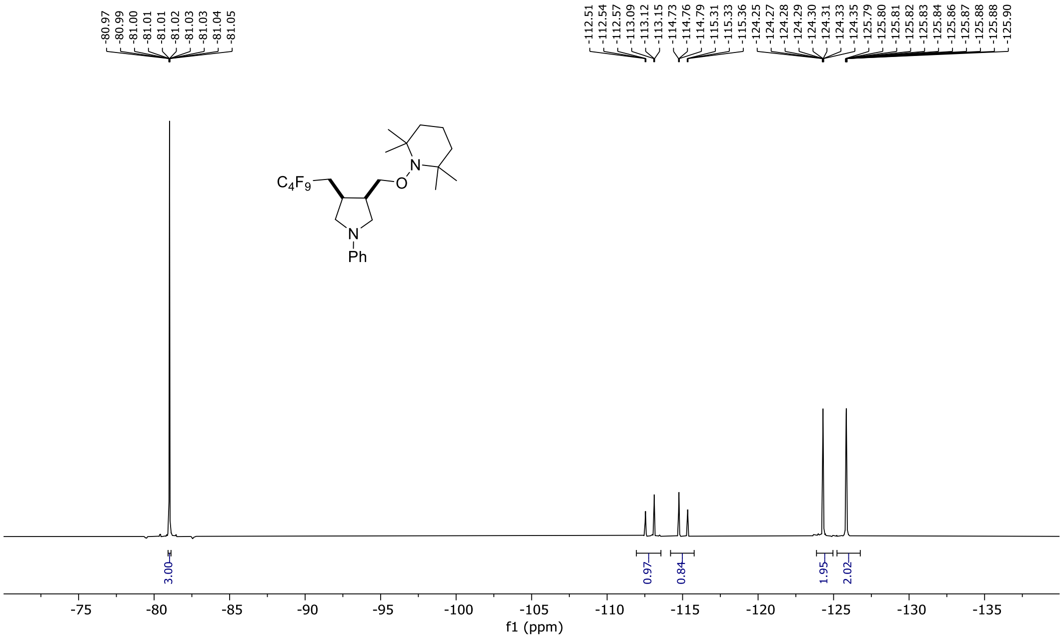

12b

^{1}H NMR (300 MHz, CDCl$_3$)

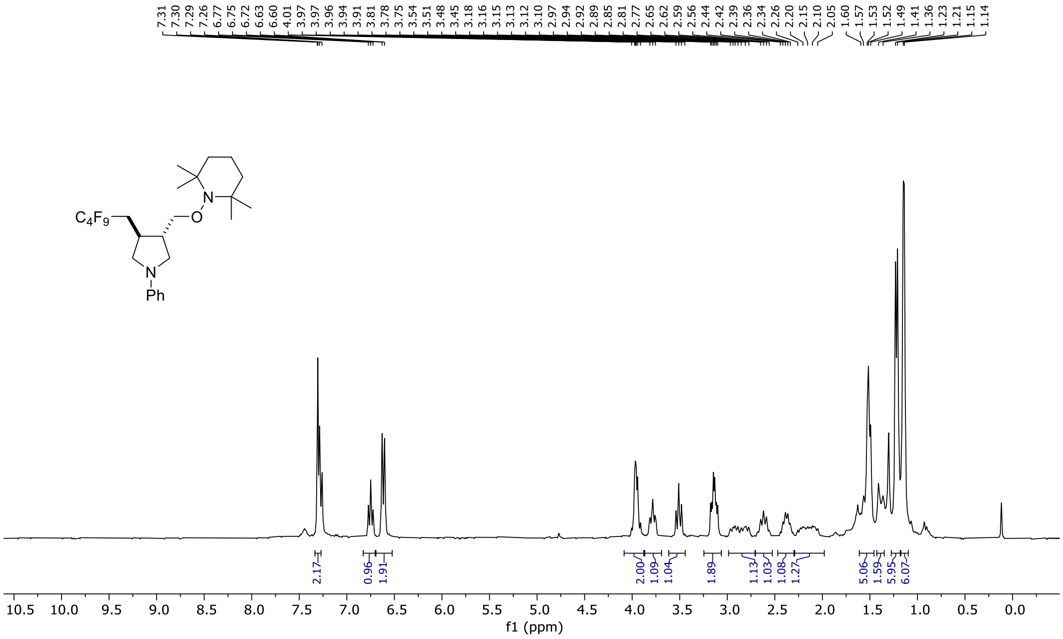

^{13}C{^{19}F} NMR (126 MHz, CDCl$_3$)

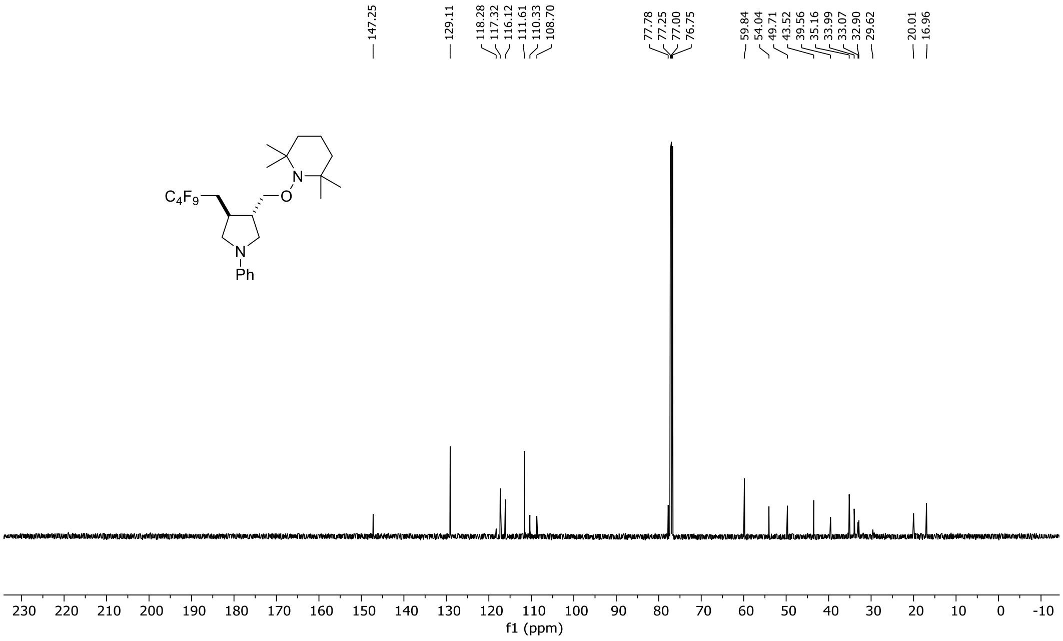

^{19}F NMR (470 MHz, $CDCl_3$)

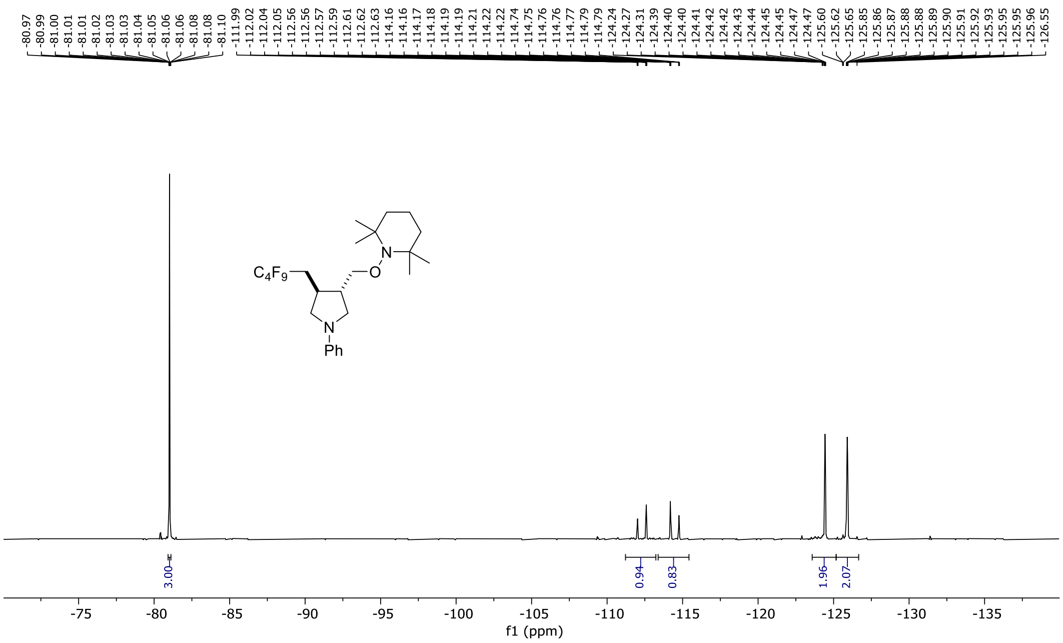

Supplement: SC-014-D3SC02544F-s001 [file SC-014-D3SC02544F-s001.pdf]
